# Supplementary material for: Proteomics research and related functional classification of liquid sclerotial exudates of Sclerotinia ginseng
Source: PeerJ. 2017 Oct 31;5:e3979. doi: 10.7717/peerj.3979 (PMC5669253; doi:10.7717/peerj.3979)
Supplement: Supplemental Information 2 [file peerj-05-3979-s002.doc]

**Supplement table 1 List of proteins identified by SDS-PAGE with LC-MS/MS in the sclerotial exudates**

| **No.** | **GI^a^** | **MS/MS Peptide sequence** |
| --- | --- | --- |
| 1 | gi|1095449307|gb|APA06271.1| | R.ATPYWYEQISHQGK.S K.SAFGPSGYK.V K.GDGVTDDTAAINAAISSGGR.C R.NLVFNNCVTAISMFWDWGWLFQGISINNCQK.G K.YYTQSKPQYNTLSVSSFTSAR.T K.VTSTITIPPGSR.I R.IGGFTGSNLQVAQCEK.N R.QLDIYAGR.G |
| 2 | gi|238477235|gb|ACR43470.1| | M.SLLTTELACTSAPDQVLHLFFQDGQNILEAR.S K.DKQLTEK.V R.MKGDNPIWEDVK.I K.IPDEVR.K K.MGIVEIR.R K.DKVQATTPLACTMTK.D K.DGSVHLFYVSK.T K.KEEELINFFPGSK.L K.LGATSVENK.I K.ITLFFR.N R.NLNPVNEVGTLENDNGSWK.H |
| 3 | gi|154693286|gb|EDN93024.1| | R.STFLMTVAGK.V K.AVNGMSFQSGQDVVVR.G K.WGVWDHK.F K.FAVDSLAAGGQDYLTITSLSAR.Q R.QAFGAVQLCGTPDKPYYFLK.E K.GIIAIEAMSR.I K.LAYQDSTGHGLLYNLYADTLLK.L K.LDFVPK.R R.IYDMQSEYYPTIANAYGVQLDSR.N K.SMFISK.L K.LATWIGVTPTDR.A |
| 4 | gi|347830055|emb|CCD45752.1| | R.AVPDQGSVGYYR.A R.VGISWISK.E K.SVWNDEVLSR.I R.SLIDIWR.F R.TQGGSNADNVLADAYVK.G K.GVRGQINWDDGYAAMVK.D K.TLTITSTGGDGNGDSNYYVQSLK.V |
| 5 | gi|154699986|gb|EDN99724.1| | R.SSTDASLTGKDWSEPGNK.L K.ELPELAGDYR.T R.IQPLSANPTWELDSMPEGR.G K.APTLEALLYDPTK.A R.FSTLATSTIPR.L R.VENYVPPYLSGDNANK.R K.LSSGSFK.A K.ADGSTLDVTFNCPAGAK.A R.MLHLDNTGFVAGATQQK.L |
| 6 | gi|154697664|gb|EDN97402.1| | R.ACWGNGFSIATDFDTK.N K.YTTNTITSGK.K R.AEVQNGCGINNNNGNIK.S K.LDYNNPSR.R |
| 7 | gi|154705171|gb|EDO04910.1| | K.DFGAVGDGVTDDTAAINAAISAGNR.C K.STILAGNTK.I K.IASWAQGK.V R.TKPQYETLPASSFLSVK.S K.GDGVTDDTAAIQAIFDK.A K.GFNDQSNPK.A K.EASQGSVGMWDVHTR.I |
| 8 | gi|154703817|gb|EDO03556.1| | R.KGPITWTQGDTTK.Q K.QTLAAIQTLAYR.Y R.YAPATDVVTGIELLNEPANWALDMGAVK.Q K.WTIVGEFSGAQTDCAK.W K.WLNGFGVGSR.Y R.YDGSYPGSPAVYGSCQTK.D K.DVGTVDGLLAIDK.V R.AGLIPQPLTSR.K |
| 9 | gi|154695005|gb|EDN94743.1| | R.DSALVFK.N R.ATAMIAYSK.W K.VVTDSFR.S K.QGSITVTSTSLAFFR.D |
| 10 | gi|1095456302|gb|APA13257.1| | K.VSGAGNYVVASPSK.A K.ANPDYFYTWTR.D R.ALQPQIEK.Y R.ALSNFR.A R.AVVNSFR.G |
| 11 | gi|154694741|gb|EDN94479.1| | K.GNAFFIGDNR.F R.GVDYQPGGSSK.I K.FKELGINTVR.V K.AVTRDMR.Q R.QYIGSR.G R.SIPVGYSAADVDSNR.L K.TGAGAGAGLTGK.G |
| 12 | gi|1095455875|gb|APA12830.1| | M.AQSLFR.L R.QINIGGWSIPSTIGIR.F R.ILDEVTLQTQK.L K.LLPNIPAAVNNFLGGR.T R.VLVSGSDPEDPR.Y R.LGEWPNFPDFTLPGVGPTVGAGGAVK.L |
| 13 | gi|154693130|gb|EDN92868.1| | K.AGFAGDDAPR.A R.AVFPSIVGRPR.H K.DSYVGDEAQSKR.G R.VAPEEHPVLLTEAPINPK.S K.LCYVALDFEQEIQTASQSSSLEK.S K.SYELPDGQVITIGNER.F K.IIAPPER.K K.QEYDESGPSIVHR.K |
| 14 | gi|154696190|gb|EDN95928.1| | K.KRPDLISR.L K.DWYFQR.V K.KEEVQPPYQNPTPLACTMTK.N R.KQPENDVGTMVYSDGK.W |
| 15 | gi|154705040|gb|EDO04779.1| | R.FGGLTPGEFLAK.W R.VDPSVLISNR.Q |
| 16 | gi|154694427|gb|EDN94165.1| | K.IFYQGLTPWK.F K.LAPVTIYDAPK.F R.NWYPVEDIKR.T R.GAAAAEVLWSGAK.D K.DPVTGQNRSQIDAGSR.L R.LPEFNEHLR.T |
| 17 | gi|1095450409|gb|APA07372.1| | R.LAEAGLK.T K.TLLLESGGPSYGVTGGDLNAR.R R.LYDTQPSTSLTSQDGIR.Y R.YLQSGYNAAR.K R.KWLVEGLGYK.D R.GQVNHWSSSCR.L |
| 18 | gi|154696912|gb|EDN96650.1| | R.ADTFAITGVQDGGIQPR.L R.QGAATWTISR.G K.RQAATPSRPR.D |
| 19 | gi|154702253|gb|EDO01992.1| | R.YVGGLLAGYDLLK.G K.TYGELSQK.G K.MYVYDSTR.F K.WVFNTEAHPLK.V |
| 20 | gi|154701174|gb|EDO00913.1| | K.AEGFNSVR.I K.FSALWSQIGTR.M |
| 21 | gi|154696764|gb|EDN96502.1| | K.IFLTPQGTYFQVK.C K.ASAVVIDPPTSAK.K |
| 22 | gi|347832626|emb|CCD48323.1| | K.AFASSFAGDQLDIAALR.A K.QQFFLQR.G |
| 23 | gi|154704206|gb|EDO03945.1| | R.AVGDSSWTSVDSAAER.A K.NKYTLTPSSSPWGRVR.V K.QAISPNAAEITK.L K.TAANAVIAAMK.T |
| 24 | gi|1095455126|gb|APA12083.1| | M.PSLHLSR.V R.GLEHIHTK.Y K.LGDLITR.K K.ANGTMTLSDLK.N R.TLLGDPK.F K.ATAAEVR.S |
| 25 | gi|154702326|gb|EDO02065.1| | R.GGQVTTLTR.A R.LAFSER.L R.DPYVGDIAVAGK.T K.TLFLSHNKPEASR.N R.NVLADLADHQR.A K.SQGQSADASRWLAR.A R.STSILNYLNSANR.R |
| 26 | gi|154698322|gb|EDN98060.1| | K.SVLGFNEPDLTYEQSSNMLPEVAAQGYK.S K.SWIQPFAGQVR.I |
| 27 | gi|347840672|emb|CCD55244.1| | R.DVILVNDVFPGPLIEANWGDTIEVTVHNEIR.G R.VLVSSDSNLIQGK.G R.LINAGTEGMQK.F R.STIAGCSLAKNPEAK.A |
| 28 | gi|154691589|gb|EDN91327.1| | R.NFMVYQR.G R.ESSETSFLR.A R.VNAPTISK.L R.VLGVSGLR.V |
| 29 | gi|154704145|gb|EDO03884.1| | R.SYDFTVQR.G R.LINAGTEGTQK.F K.SGSYWMR.S |
| 30 | gi|154697112|gb|EDN96850.1| | R.SQSIYQVITDR.F R.FIASVNQIR.N |
| 31 | gi|347831150|emb|CCD46847.1| | K.GGFNMQSTTSIDCSGFK.S |
| 32 | gi|154698335|gb|EDN98073.1| | K.TQADWENDFR.M K.QALLAATR.T |
| 33 | gi|347841076|emb|CCD55648.1| | R.GGPVTTYLQSALQR.S |
| 34 | gi|154700524|gb|EDO00263.1| | R.ILDTGTTFVEEHNGWWQLIDATGDGRPDLAYIKNK.N K.SSNSVLPDLVYIK.T K.LDLTYIK.Y |
| 35 | gi|347836311|emb|CCD50883.1| | K.ALSSNIDDR.T R.TLHEFYMWPFAEGIKAGVGAVMTSYNDVNGSAASQNSYLINNLIK.D R.LNDMVTR.I K.AITSVPPAR.A K.QVVSLAVTR.K |
| 36 | gi|154696502|gb|EDN96240.1| | M.PLPLSPR.S R.TSYYDLHYQIAR.T |
| 37 | gi|154691568|gb|EDN91306.1| | K.IASTWEGIQAAR.I |
| 38 | gi|347838742|emb|CCD53314.1| | K.WTITIPSNPTVR.F K.IIELEGR.G |
| 39 | gi|154698875|gb|EDN98613.1| | R.SIYQIITDR.Y K.KLSAELHK.R |
| 40 | gi|347827005|emb|CCD42702.1| | K.ITAFQPGHR.M R.MVVGNPALR.T |
| 41 | gi|507414638|emb|CCD56877.2| | R.SGPCGYNAR.V R.ICQDQSIVDK.L |
| 42 | gi|154702896|gb|EDO02635.1| | R.YYDLTVSR.S K.VPNGPVFPNVDNNLINGK.G R.LINAGSSGTQK.F R.SDLDVPCMR.L |
| 43 | gi|347827354|emb|CCD43051.1| | R.EVDDLDTAVR.N K.NVNKPLTPLSDWLVR.F R.VFEAEDPKNWK.T K.EKVPNFDNKTTYGELFQEGIWTHSFLNTFDYHR.Q K.KAGGHNFLAPR.R |
| 44 | gi|1095454060|gb|APA11018.1| | R.EYAPMDVPR.H |
| 45 | gi|1095457170|gb|APA14123.1| | M.VSPNLLNWR.V K.TIQIIVQAVMQIFVKTLTGKTITLEVESSDTIDNVK.A K.ESTLHLVLR.L |
| 46 | gi|347838722|emb|CCD53294.1| | K.FWDFELK.Q |
| 47 | gi|154693234|gb|EDN92972.1| | K.NNQAAITAIR.K |
| 48 | gi|154703307|gb|EDO03046.1| | K.KSPAAVAAQR.G R.GIQEKAVLGNASQQGIVNANPSDALAGK.E R.LELLQQAKDHSSNGSK.E R.VNDANGQTR.F R.LRPDQR.A |
| 49 | gi|154691708|gb|EDN91446.1| | R.TFWVVR.Q |
| 50 | gi|154693514|gb|EDN93252.1| | R.LSSNQAWSK.G |
| 51 | gi|347830059|emb|CCD45756.1| | R.TCHVSSLGSGR.D |
| 52 | gi|347837081|emb|CCD51653.1| | R.IFPDRADEIR.M R.AALEILR.Y K.RKIMAAVGPR.I |
| 53 | gi|154693707|gb|EDN93445.1| | R.SSSPRRDK.R R.DNTKNEELVSK.A |
| 54 | gi|154691632|gb|EDN91370.1| | K.LSALQTR.D R.SISLEKR.A R.TQALSCPIGDDIQTR.M R.DQLRAGR.G |
| 55 | gi|154703678|gb|EDO03417.1| | K.QLAAMIPK.Y |
| 56 | gi|347830591|emb|CCD46288.1| | R.DRANSKSR.H R.TPAWPPSVRDRPEAEALMAGSGSDADSSMAEAVSQK.T R.MARHNAIVR.K R.ILRIGNIANNAR.L R.TGVSKSIR.R K.KLGMPIAEPR.A |
| 57 | gi|347836319|emb|CCD50891.1| | K.NTNNALPLSKPK.F R.TPFTWGPTR.E |
| 58 | gi|154703202|gb|EDO02941.1| | R.EVSEDLAGLSDK.E |
| 59 | gi|154693400|gb|EDN93138.1| | M.TSSLEMGGLR.S |
