# Supplementary material for: Proteomics research and related functional classification of liquid sclerotial exudates of Sclerotinia ginseng
Source: PeerJ. 2017 Oct 31;5:e3979. doi: 10.7717/peerj.3979 (PMC5669253; doi:10.7717/peerj.3979)
Supplement: Supplemental Information 3 — Unknown protein sequences from the excised gel slices resulted in peptide mass fingerprints that were used to search the NCBInr database using Mascot software (www.matrixscience.com) to obtain basic information on those proteins such as the nominal mass and calculated isoelectric point. [file peerj-05-3979-s003.html]

Submitted from 11743-renshenhepanjun by Mascot Daemon on DELL-PC (Mascot Search Results)

 

# MASCOT Search Results

Search metadata

|  |  |  |
| --- | --- | --- |
| User | : | protein2 |
| E-mail | : | protein2@sangon.com |
| Search title | : | Submitted from 11743-renshenhepanjun by Mascot Daemon on DELL-PC |
| MS data file | : | C:\Users\DELL\Desktop\20161130-LC-MS\shenggong\_11743\_90min.mgf |
| Database | : | complexdatabase (59,551 sequences; 22,351,232 residues) |
| Timestamp | : | 5 Dec 2016 at 07:02:24 GMT |

Repeat search

All

Non-significant

Unassigned


[help]

Export


As
XML
CSV
pepXML
mzIdentML
DTASelect
Mascot DAT File
MGF Peak List

Not what you expected? Try the select summary.

Search parameters

|  |  |  |
| --- | --- | --- |
| Type of search | : | MS/MS Ion Search |
| Enzyme | : | Trypsin |
| Fixed modifications | : | Carbamidomethyl (C) |
| Variable modifications | : | Acetyl (Protein N-term), Deamidated (NQ), Dioxidation (W), Oxidation (M) |
| Mass values | : | Monoisotopic |
| Protein mass | : | Unrestricted |
| Peptide mass tolerance | : | ± 30 ppm |
| Fragment mass tolerance | : | ± 0.15 Da |
| Max missed cleavages | : | 2 |
| Instrument type | : | ESI-QUAD-TOF |
| Number of queries | : | 24,686 |

### Score distribution

Peptide score distribution. Ions score is −10log(*P*), where *P* is the probability that the observed match is a random event. Individual ions scores **> 33** indicate **identity** or **extensive homology** (p<0.05).

**[Deprecated]** Score distribution for family members in the first 50 proteins.
Protein scores are derived from ions scores as a non-probabilistic basis for ranking protein families.

Legend

| Dupes | Expect | Rank | U | 1 | 2 | Peptide |  |
| --- | --- | --- | --- | --- | --- | --- | --- |
|  | 0.037 | 2 |  |  |  | GAYSLSLR | significant |
|  | 9 | 1 |  |  |  | GFFLFVEGGR | top ranking |
|  | 6.4e-005 | 1 |  |  |  | GSSIFGLAPGK | significant and top ranking |
|  | 1.3e-006 | 1 |  |  |  | SSGTSYPDVLK | peptide is found in all proteins in family member 1 |
|  | 6.2e-007 | 1 |  |  |  | VCNYVSWIK | peptide is found in some but not all proteins in family member 2 |
|  | 6.4e-005 | 1 | U |  |  | GSSIFGLAPGK | unique |
| 2 | 5.7e-005 | 1 |  |  |  | LNTLETEEWFFK | peptide has two duplicates |
|  | 0.18 | 1 |  |  |  | LNTLETEEWFFK | duplicate peptide |

Right-facing triangle () in the Dupes or Rank column indicates content that can be expanded by clicking on it. Down-facing triangle () indicates the content is expanded and can be collapsed. For more details about particular columns, see results format help.

## Protein Family Summary

Filters

Significance threshold p<

Ions score or expect cut-off

Max. number of families

Dendrograms cut at

[help]

Show

- Proteins (122)
- Quantitation (128)
- Unassigned (23924)

§ link to

## Protein families 1–122 (out of 122)

---

15102050100200300400500600700800900100015002000allper page

Page: 1

AccessionDescriptionQueryObservedMr(expt)Mr(calc)SequenceFamily numberPage numberMatch case

### 1

|  |  | Accession | Score | Description |
| --- | --- | --- | --- | --- |
|  | 1 | gi|1095449307|gb|APA06271.1| | 1206 | hypothetical protein sscle\_01g010410 [Sclerotinia sclerotiorum 1980 UF-70] |

|  |  | Score | Mass | Matches | Sequences | emPAI |  |
| --- | --- | --- | --- | --- | --- | --- | --- |
| 1.1 | gi|1095449307|gb|APA06271.1| | 1206 | 87452 | 74 (51) | 8 (7) | 0.55 |  |
|  | hypothetical protein sscle\_01g010410 [Sclerotinia sclerotiorum 1980 UF-70] | | | | | | |

|  |  | Score | Mass | Matches | Sequences | emPAI |  |
| --- | --- | --- | --- | --- | --- | --- | --- |
| 1.1 | gi|1095449307|gb|APA06271.1| | 1206 | 87452 | 74 (51) | 8 (7) | 0.55 | hypothetical protein sscle\_01g010410 [Sclerotinia sclerotiorum 1980 UF-70] |
|  |  | | | | | | |

#### 74 peptide matches (16 non-duplicate, 58 duplicate)

Auto-fit to window

| Query | Dupes | Observed | Mr(expt) | Mr(calc) | ppm | M | Score | Expect | Rank | U | Peptide |
| --- | --- | --- | --- | --- | --- | --- | --- | --- | --- | --- | --- |

| Query | Dupes | Observed | Mr(expt) | Mr(calc) | ppm | M | Score | Expect | Rank | U | Peptide |
| --- | --- | --- | --- | --- | --- | --- | --- | --- | --- | --- | --- |
| Query | Dupes | Observed | Mr(expt) | Mr(calc) | ppm | M | Score | Expect | Rank | U | Peptide |
| --- | --- | --- | --- | --- | --- | --- | --- | --- | --- | --- | --- |
| 1051 | 3 | 457.2244 | 912.4342 | 912.4341 | 0.13 | 0 | 26 | 0.015 | 1Score **> 29** indicates **identity** Score **> 20** indicates **homology** | U | K.SAFGPSGYK.V |
| 1227 | 4 | 468.2508 | 934.4870 | 934.4872 | -0.20 | 0 | 46 | 0.0029 | 1Score **> 33** indicates **identity** | U | R.QLDIYAGR.G |
| 1234 |  | 468.7429 | 935.4713 | 935.4712 | 0.071 | 0 | 23 | 0.02 | 1Score **> 33** indicates **identity** Score **> 18** indicates **homology** | U | R.QLDIYAGR.G + Deamidated (NQ) |
| 4146 | 29 | 614.8483 | 1227.6821 | 1227.6823 | -0.17 | 0 | 57 | 9.5e-005 | 1Score **> 33** indicates **identity** Score **> 29** indicates **homology** | U | K.VTSTITIPPGSR.I |
| 8619 | 2 | 854.4103 | 1706.8061 | 1706.8053 | 0.51 | 0 | 62 | 0.00011 | 1Score **> 35** indicates **identity** | U | R.ATPYWYEQISHQGK.S |
| 8632 | 6 | 854.9204 | 1707.8263 | 1707.8250 | 0.73 | 0 | 77 | 1.1e-006 | 1Score **> 35** indicates **identity** Score **> 30** indicates **homology** | U | R.IGGFTGSNLQVAQCEK.N |
| 8637 | 1 | 855.4112 | 1708.8078 | 1708.8090 | -0.71 | 0 | 57 | 6.2e-005 | 1Score **> 34** indicates **identity** Score **> 28** indicates **homology** | U | R.IGGFTGSNLQVAQCEK.N + Deamidated (NQ) |
| 8640 |  | 855.4172 | 1708.8199 | 1708.8090 | 6.37 | 0 | 29 | 0.22 | 1Score **> 35** indicates **identity** | U | R.IGGFTGSNLQVAQCEK.N + Deamidated (NQ) |
| 9765 |  | 616.6299 | 1846.8680 | 1846.8657 | 1.24 | 0 | 17 | 1 | 1Score **> 34** indicates **identity** Score **> 30** indicates **homology** | U | K.GDGVTDDTAAINAAISSGGR.C |
| 9766 | 6 | 924.4413 | 1846.8680 | 1846.8657 | 1.25 | 0 | 133 | 2.6e-012 | 1Score **> 34** indicates **identity** Score **> 29** indicates **homology** | U | K.GDGVTDDTAAINAAISSGGR.C |
| 9784 | 2 | 924.9309 | 1847.8473 | 1847.8497 | -1.32 | 0 | 65 | 1.1e-005 | 1Score **> 34** indicates **identity** Score **> 28** indicates **homology** | U | K.GDGVTDDTAAINAAISSGGR.C + Deamidated (NQ) |
| 13704 | 2 | 1214.5914 | 2427.1683 | 2427.1706 | -0.96 | 0 | 75 | 5.4e-006 | 1Score **> 35** indicates **identity** | U | K.YYTQSKPQYNTLSVSSFTSAR.T |
| 13709 | 2 | 810.0646 | 2427.1721 | 2427.1706 | 0.60 | 0 | 52 | 0.0011 | 1Score **> 35** indicates **identity** | U | K.YYTQSKPQYNTLSVSSFTSAR.T |
| 13711 | 1 | 810.3917 | 2428.1532 | 2428.1546 | -0.62 | 0 | 30 | 0.16 | 1Score **> 35** indicates **identity** | U | K.YYTQSKPQYNTLSVSSFTSAR.T + Deamidated (NQ) |
| 13713 |  | 1215.0858 | 2428.1571 | 2428.1546 | 1.00 | 0 | 50 | 0.0018 | 1Score **> 35** indicates **identity** | U | K.YYTQSKPQYNTLSVSSFTSAR.T + Deamidated (NQ) |
| 22301 |  | 1293.2094 | 3876.6062 | 3876.6746 | -17.6 | 0 | 0 | 1 | 2Score **> 29** indicates **identity** Score **> 13** indicates **homology** | U | R.NLVFNNCVTAISMFWDWGWLFQGISINNCQK.G + 3 Deamidated (NQ); 3 Dioxidation (W); Oxidation (M) |

#### 2 subsets and intersections (3 subset proteins in total)

|  |  | Score | Mass | Subset of |  |
| --- | --- | --- | --- | --- | --- |
|  | gi|154696565|gb|EDN96303.1| | 631 | 77399 | 1.1 |  |
|  | hypothetical protein SS1G\_01229 [Sclerotinia sclerotiorum 1980 UF-70] | | | | |
|  | 1 sameset of gi|154696565|gb|EDN96303.1| | | | | |
|  | gi|156062226|ref|XP\_001597035.1| | 631 | 77399 |  |  |
|  | hypothetical protein SS1G\_01229 [Sclerotinia sclerotiorum 1980 UF-70] | | | | |
|  | gi|507414518|emb|CCD47609.2| | 515 | 84454 | 1.1 |  |
|  | glycoside hydrolase family 55 protein [Botrytis cinerea T4] | | | | |

|  |  | Score | Mass | Subset of |  |
| --- | --- | --- | --- | --- | --- |
|  | gi|154696565|gb|EDN96303.1| | 631 | 77399 | 1.1 | hypothetical protein SS1G\_01229 [Sclerotinia sclerotiorum 1980 UF-70] |
|  |  | | | | |
|  | 1 sameset of gi|154696565|gb|EDN96303.1| | | | | |
|  | gi|156062226|ref|XP\_001597035.1| | 631 | 77399 |  | hypothetical protein SS1G\_01229 [Sclerotinia sclerotiorum 1980 UF-70] |
|  |  | | | | |
|  | gi|507414518|emb|CCD47609.2| | 515 | 84454 | 1.1 | glycoside hydrolase family 55 protein [Botrytis cinerea T4] |
|  |  | | | | |

---

### 2

|  |  | Accession | Score | Description |
| --- | --- | --- | --- | --- |
|  | 1 | gi|238477235|gb|ACR43470.1| | 915 | developmental-specific protein Ssp1 [Sclerotinia minor] |
|  | 2 | gi\|238477233\|gb\|ACR43469.1\| | 624 | developmental-specific protein Ssp1 [Sclerotinia trifoliorum] |
|  | 3 | gi\|1095456207\|gb\|APA13162.1\| | 477 | hypothetical protein sscle\_10g079320 [Sclerotinia sclerotiorum 1980 UF-70] |
|  |  |  |  |  |
| Cut threshold Threshold (**0**): | | | | |

|  |  | Score | Mass | Matches | Sequences | emPAI |  |
| --- | --- | --- | --- | --- | --- | --- | --- |
| 2.1 | gi|238477235|gb|ACR43470.1| | 915 | 35067 | 86 (45) | 17 (13) | 5.09 |  |
|  | developmental-specific protein Ssp1 [Sclerotinia minor] | | | | | | |
| 2.2 | gi|238477233|gb|ACR43469.1| | 624 | 35151 | 66 (31) | 12 (9) | 2.86 |  |
|  | developmental-specific protein Ssp1 [Sclerotinia trifoliorum] | | | | | | |
| 2.3 | gi|1095456207|gb|APA13162.1| | 477 | 35188 | 64 (26) | 13 (9) | 2.53 |  |
|  | hypothetical protein sscle\_10g079320 [Sclerotinia sclerotiorum 1980 UF-70] | | | | | | |
|  | 3 samesets of gi|1095456207|gb|APA13162.1| | | | | | | |
|  | gi|154699467|gb|EDN99205.1| | 477 | 35188 | 64 (26) | 13 (9) | 2.53 |  |
|  | predicted protein [Sclerotinia sclerotiorum 1980 UF-70] | | | | | | |
|  | gi|156031287|ref|XP\_001584968.1| | 477 | 35188 | 64 (26) | 13 (9) | 2.53 |  |
|  | predicted protein [Sclerotinia sclerotiorum 1980 UF-70] | | | | | | |
|  | gi|238477227|gb|ACR43466.1| | 477 | 35188 | 64 (26) | 13 (9) | 2.53 |  |
|  | developmental-specific protein Ssp1 [Sclerotinia sclerotiorum] | | | | | | |
|  | | | | | | |

|  |  | Score | Mass | Matches | Sequences | emPAI |  |
| --- | --- | --- | --- | --- | --- | --- | --- |
| 2.1 | gi|238477235|gb|ACR43470.1| | 915 | 35067 | 86 (45) | 17 (13) | 5.09 | developmental-specific protein Ssp1 [Sclerotinia minor] |
|  |  | | | | | | |
| 2.2 | gi|238477233|gb|ACR43469.1| | 624 | 35151 | 66 (31) | 12 (9) | 2.86 | developmental-specific protein Ssp1 [Sclerotinia trifoliorum] |
|  |  | | | | | | |
| 2.3 | gi|1095456207|gb|APA13162.1| | 477 | 35188 | 64 (26) | 13 (9) | 2.53 | hypothetical protein sscle\_10g079320 [Sclerotinia sclerotiorum 1980 UF-70] |
|  |  | | | | | | |
|  | 3 samesets of gi|1095456207|gb|APA13162.1| | | | | | | |
|  | gi|154699467|gb|EDN99205.1| | 477 | 35188 | 64 (26) | 13 (9) | 2.53 | predicted protein [Sclerotinia sclerotiorum 1980 UF-70] |
|  |  | | | | | | |
|  | gi|156031287|ref|XP\_001584968.1| | 477 | 35188 | 64 (26) | 13 (9) | 2.53 | predicted protein [Sclerotinia sclerotiorum 1980 UF-70] |
|  |  | | | | | | |
|  | gi|238477227|gb|ACR43466.1| | 477 | 35188 | 64 (26) | 13 (9) | 2.53 | developmental-specific protein Ssp1 [Sclerotinia sclerotiorum] |
|  |  | | | | | | |
|  | | | | | | |

#### 99 peptide matches (47 non-duplicate, 52 duplicate)

Auto-fit to window

| Query | Dupes | Observed | Mr(expt) | Mr(calc) | ppm | M | Score | Expect | Rank | U | 1 | 2 | 3 | Peptide |
| --- | --- | --- | --- | --- | --- | --- | --- | --- | --- | --- | --- | --- | --- | --- |

| Query | Dupes | Observed | Mr(expt) | Mr(calc) | ppm | M | Score | Expect | Rank | U | 1 | 2 | 3 | Peptide |
| --- | --- | --- | --- | --- | --- | --- | --- | --- | --- | --- | --- | --- | --- | --- |
| Query | Dupes | Observed | Mr(expt) | Mr(calc) | ppm | M | Score | Expect | Rank | U | 1 | 2 | 3 | Peptide |
| --- | --- | --- | --- | --- | --- | --- | --- | --- | --- | --- | --- | --- | --- | --- |
| 67 | 2 | 364.7006 | 727.3867 | 727.3864 | 0.34 | 0 | 44 | 0.0012 | 1Score **> 27** indicates **identity** | U |  |  |  | K.IPDEVR.K |
| 283 |  | 396.2241 | 790.4336 | 790.4337 | -0.23 | 0 | 7 | 0.51 | 1Score **> 32** indicates **identity** Score **> 17** indicates **homology** | U |  |  |  | K.HGTPVIPA.- |
| 311 | 1 | 398.7397 | 795.4648 | 795.4643 | 0.65 | 0 | 26 | 0.037 | 1Score **> 27** indicates **identity** Score **> 24** indicates **homology** | U |  |  |  | K.ITLFFR.N |
| 408 | 1 | 409.2338 | 816.4530 | 816.4527 | 0.27 | 0 | 51 | 0.00037 | 1Score **> 34** indicates **identity** Score **> 30** indicates **homology** | U |  |  |  | K.MGIVEIR.R |
| 484 | 2 | 417.2311 | 832.4477 | 832.4477 | 0.079 | 0 | 41 | 0.0035 | 1Score **> 33** indicates **identity** Score **> 29** indicates **homology** | U |  |  |  | K.MGIVEIR.R + Oxidation (M) |
| 649 |  | 428.7478 | 855.4810 | 855.4814 | -0.41 | 1 | 12 | 1.3 | 2Score **> 29** indicates **identity** Score **> 26** indicates **homology** | U |  |  |  | K.IPDEVRK.G |
| 688 |  | 431.7372 | 861.4599 | 861.4443 | 18.0 | 1 | 11 | 10 | 8Score **> 34** indicates **identity** | U |  |  |  | K.DKQLTEK.V + Deamidated (NQ) |
| 1110 | 5 | 459.7482 | 917.4818 | 917.4818 | 0.057 | 0 | 53 | 0.00065 | 1Score **> 34** indicates **identity** | U |  |  |  | K.LGATSVENK.I |
| 4444 | 3 | 626.3202 | 1250.6258 | 1250.6295 | -2.97 | 0 | 29 | 0.0054 | 1Score **> 34** indicates **identity** Score **> 19** indicates **homology** | U |  |  |  | K.DGSVHLFYVSK |
| 4453 | 3 | 417.8854 | 1250.6342 | 1250.6295 | 3.74 | 0 | 11 | 1 | 1Score **> 34** indicates **identity** Score **> 23** indicates **homology** | U |  |  |  | K.DGSVHLFYVSK |
| 6035 | 3 | 705.8466 | 1409.6786 | 1409.6714 | 5.06 | 0 | 63 | 6e-005 | 1Score **> 34** indicates **identity** | U |  |  |  | K.EEELINFFPGSK.L + Deamidated (NQ) |
| 6312 | 2 | 719.3576 | 1436.7006 | 1436.7003 | 0.21 | 0 | 43 | 9.5e-005 | 1Score **> 34** indicates **identity** Score **> 15** indicates **homology** | U |  |  |  | K.VQATTPLACTMTK.D + Oxidation (M) |
| 6410 | 3 | 724.3474 | 1446.6803 | 1446.6813 | -0.72 | 1 | 62 | 3.9e-005 | 1Score **> 33** indicates **identity** Score **> 30** indicates **homology** | U |  |  |  | R.MKGDNPIWEDVK.I + Oxidation (M) |
| 6413 |  | 483.2345 | 1446.6817 | 1446.6813 | 0.25 | 1 | 8 | 0.31 | 1Score **> 34** indicates **identity** Score **> 16** indicates **homology** | U |  |  |  | R.MKGDNPIWEDVK.I + Oxidation (M) |
| 7224 | 1 | 513.5968 | 1537.7686 | 1537.7664 | 1.42 | 1 | 16 | 0.048 | 1Score **> 34** indicates **identity** Score **> 15** indicates **homology** | U |  |  |  | K.KEEELINFFPGSK.L + Deamidated (NQ) |
| 7225 | 1 | 769.8916 | 1537.7686 | 1537.7664 | 1.47 | 1 | 80 | 1.3e-006 | 1Score **> 34** indicates **identity** | U |  |  |  | K.KEEELINFFPGSK.L + Deamidated (NQ) |
| 8253 |  | 832.9207 | 1663.8269 | 1663.8273 | -0.27 | 1 | 25 | 0.52 | 1Score **> 35** indicates **identity** | U |  |  |  | K.DKVQATTPLACTMTK.D |
| 8444 | 2 | 560.9475 | 1679.8207 | 1679.8222 | -0.92 | 1 | 16 | 0.047 | 1Score **> 35** indicates **identity** Score **> 15** indicates **homology** | U |  |  |  | K.DKVQATTPLACTMTK.D + Oxidation (M) |
| 8445 | 1 | 840.9178 | 1679.8210 | 1679.8222 | -0.73 | 1 | 55 | 9.3e-006 | 1Score **> 35** indicates **identity** Score **> 17** indicates **homology** | U |  |  |  | K.DKVQATTPLACTMTK.D + Oxidation (M) |
| 8452 |  | 841.4093 | 1680.8040 | 1680.8063 | -1.32 | 1 | 39 | 0.0017 | 1Score **> 34** indicates **identity** Score **> 24** indicates **homology** | U |  |  |  | K.DKVQATTPLACTMTK.D + Deamidated (NQ); Oxidation (M) |
| 9878 |  | 929.4593 | 1856.9040 | 1856.9091 | -2.72 | 0 | 1 | 1 | 2Score **> 34** indicates **identity** Score **> 14** indicates **homology** | U |  |  |  | K.WGDELPGTALACVIGNGK.I |
| 10075 | 1 | 627.9845 | 1880.9317 | 1880.9268 | 2.57 | 1 | 5 | 0.32 | 1Score **> 35** indicates **identity** Score **> 13** indicates **homology** | U |  |  |  | K.GDNPIWEDVKIPDEVR.K |
| 10076 | 1 | 941.4784 | 1880.9422 | 1880.9268 | 8.18 | 1 | 67 | 6.2e-006 | 1Score **> 35** indicates **identity** Score **> 27** indicates **homology** | U |  |  |  | K.GDNPIWEDVKIPDEVR.K |
| 11703 | 3 | 1050.5046 | 2098.9947 | 2098.9919 | 1.33 | 0 | 76 | 2.5e-007 | 1Score **> 35** indicates **identity** Score **> 22** indicates **homology** | U |  |  |  | R.NLNPVNEVGTLENDNGSWK.H |
| 11704 |  | 700.6726 | 2098.9958 | 2098.9919 | 1.85 | 0 | 26 | 0.37 | 1Score **> 35** indicates **identity** | U |  |  |  | R.NLNPVNEVGTLENDNGSWK.H |
| 11716 | 1 | 701.0009 | 2099.9807 | 2099.9760 | 2.27 | 0 | 17 | 0.027 | 1Score **> 34** indicates **identity** Score **> 14** indicates **homology** | U |  |  |  | R.NLNPVNEVGTLENDNGSWK.H + Deamidated (NQ) |
| 11718 | 3 | 1050.9995 | 2099.9845 | 2099.9760 | 4.06 | 0 | 85 | 1.9e-007 | 1Score **> 34** indicates **identity** Score **> 30** indicates **homology** | U |  |  |  | R.NLNPVNEVGTLENDNGSWK.H + Deamidated (NQ) |
| 11719 |  | 1050.9998 | 2099.9850 | 2099.9760 | 4.29 | 0 | 60 | 0.00013 | 1Score **> 34** indicates **identity** | U |  |  |  | R.NLNPVNEVGTLENDNGSWK.H + Deamidated (NQ) |
| 12126 |  | 540.0220 | 2156.0590 | 2156.0572 | 0.85 | 2 | 11 | 1 | 1Score **> 35** indicates **identity** Score **> 23** indicates **homology** | U |  |  |  | R.MKGDNPIWEDVKIPDEVR.K + Oxidation (M) |
| 12128 | 1 | 719.6939 | 2156.0599 | 2156.0572 | 1.26 | 2 | 37 | 0.00064 | 1Score **> 35** indicates **identity** Score **> 18** indicates **homology** | U |  |  |  | R.MKGDNPIWEDVKIPDEVR.K + Oxidation (M) |
| 12130 | 1 | 1079.0376 | 2156.0606 | 2156.0572 | 1.60 | 2 | 62 | 1.9e-006 | 1Score **> 35** indicates **identity** Score **> 17** indicates **homology** | U |  |  |  | R.MKGDNPIWEDVKIPDEVR.K + Oxidation (M) |
| 13781 | 1 | 817.0704 | 2448.1895 | 2448.1995 | -4.10 | 2 | 40 | 0.021 | 1Score **> 35** indicates **identity** | U |  |  |  | K.IVHCTDGKKEEELINFFPGSK.L + Deamidated (NQ) |
| 13783 | 1 | 613.0568 | 2448.1982 | 2448.1995 | -0.54 | 2 | 5 | 1 | 1Score **> 35** indicates **identity** Score **> 18** indicates **homology** | U |  |  |  | K.IVHCTDGKKEEELINFFPGSK.L + Deamidated (NQ) |
| 15858 |  | 1396.6388 | 2791.2630 | 2791.2627 | 0.13 | 0 | 88 | 5.8e-009 | 1Score **> 34** indicates **identity** Score **> 19** indicates **homology** | U |  |  |  | K.LTSGSFNQGTGWNPNGSQWAYFSASK.D |
| 15881 | 1 | 1397.1307 | 2792.2469 | 2792.2467 | 0.091 | 0 | 64 | 4.5e-005 | 1Score **> 33** indicates **identity** | U |  |  |  | K.LTSGSFNQGTGWNPNGSQWAYFSASK.D + Deamidated (NQ) |
| 15883 | 1 | 1397.1326 | 2792.2506 | 2792.2467 | 1.40 | 0 | 31 | 0.11 | 1Score **> 34** indicates **identity** | U |  |  |  | K.LTSGSFNQGTGWNPNGSQWAYFSASK.D + Deamidated (NQ) |
| 15885 |  | 931.7579 | 2792.2520 | 2792.2467 | 1.89 | 0 | 10 | 1 | 1Score **> 34** indicates **identity** Score **> 22** indicates **homology** | U |  |  |  | K.LTSGSFNQGTGWNPNGSQWAYFSASK.D + Deamidated (NQ) |
| 15896 |  | 1398.1409 | 2794.2672 | 2794.2147 | 18.8 | 0 | 15 | 1 | 1Score **> 34** indicates **identity** Score **> 27** indicates **homology** | U |  |  |  | K.LTSGSFNQGTGWNPNGSQWAYFSASK.D + 3 Deamidated (NQ) |
| 20756 |  | 1765.3870 | 3528.7594 | 3528.7559 | 0.99 | 0 | 6 | 1 | 1Score **> 35** indicates **identity** Score **> 19** indicates **homology** | U |  |  |  | M.SLLTTELACTSAPDQVLHLFFQDGQNILEAR.S + Acetyl (Protein N-term) |
| 20757 | 5 | 1177.2605 | 3528.7597 | 3528.7559 | 1.08 | 0 | 11 | 1 | 1Score **> 35** indicates **identity** Score **> 24** indicates **homology** | U |  |  |  | M.SLLTTELACTSAPDQVLHLFFQDGQNILEAR.S + Acetyl (Protein N-term) |
| 20761 |  | 883.1984 | 3528.7646 | 3528.7559 | 2.48 | 0 | 2 | 1 | 1Score **> 35** indicates **identity** Score **> 15** indicates **homology** | U |  |  |  | M.SLLTTELACTSAPDQVLHLFFQDGQNILEAR.S + Acetyl (Protein N-term) |
| 20775 |  | 1765.8892 | 3529.7638 | 3529.7399 | 6.77 | 0 | 12 | 1 | 1Score **> 35** indicates **identity** Score **> 24** indicates **homology** | U |  |  |  | M.SLLTTELACTSAPDQVLHLFFQDGQNILEAR.S + Acetyl (Protein N-term); Deamidated (NQ) |
| 20776 |  | 1177.5991 | 3529.7755 | 3529.7399 | 10.1 | 0 | 4 | 1 | 1Score **> 35** indicates **identity** Score **> 16** indicates **homology** | U |  |  |  | M.SLLTTELACTSAPDQVLHLFFQDGQNILEAR.S + Acetyl (Protein N-term); Deamidated (NQ) |
| 20777 | 1 | 1765.9131 | 3529.8116 | 3529.7399 | 20.3 | 0 | 32 | 0.088 | 1Score **> 34** indicates **identity** | U |  |  |  | M.SLLTTELACTSAPDQVLHLFFQDGQNILEAR.S + Acetyl (Protein N-term); Deamidated (NQ) |
| 20782 | 1 | 1177.9279 | 3530.7618 | 3530.7239 | 10.7 | 0 | 17 | 1 | 1Score **> 35** indicates **identity** Score **> 29** indicates **homology** | U |  |  |  | M.SLLTTELACTSAPDQVLHLFFQDGQNILEAR.S + Acetyl (Protein N-term); 2 Deamidated (NQ) |
| 20784 |  | 883.6993 | 3530.7683 | 3530.7239 | 12.6 | 0 | 1 | 1 | 1Score **> 35** indicates **identity** Score **> 14** indicates **homology** | U |  |  |  | M.SLLTTELACTSAPDQVLHLFFQDGQNILEAR.S + Acetyl (Protein N-term); 2 Deamidated (NQ) |
| 21371 |  | 1212.5996 | 3634.7770 | 3634.7647 | 3.38 | 0 | 0 | 1 | 1Score **> 35** indicates **identity** Score **> 13** indicates **homology** | U |  |  |  | -.MSLLTTELACTSAPDQVLHLFFQDGQNILEAR.S + Deamidated (NQ); Oxidation (M) |

#### 2 subsets and intersections (3 subset proteins in total)

|  |  | Score | Mass | Subset of |  |
| --- | --- | --- | --- | --- | --- |
|  | gi|238477229|gb|ACR43467.1| | 210 | 35565 | 2.1, 2.2 |  |
|  | developmental-specific protein Ssp1 [Botrytis cinerea] | | | | |
|  | 1 sameset of gi|238477229|gb|ACR43467.1| | | | | |
|  | gi|347837504|emb|CCD52076.1| | 210 | 35565 |  |  |
|  | hypothetical protein BofuT4\_P082350.1 [Botrytis cinerea T4] | | | | |
|  | gi|347837689|emb|CCD52261.1| | 26 | 71106 | 2.1 |  |
|  | similar to ferric reductase like transmembrane component [Botrytis cinerea T4] | | | | |

|  |  | Score | Mass | Subset of |  |
| --- | --- | --- | --- | --- | --- |
|  | gi|238477229|gb|ACR43467.1| | 210 | 35565 | 2.1, 2.2 | developmental-specific protein Ssp1 [Botrytis cinerea] |
|  |  | | | | |
|  | 1 sameset of gi|238477229|gb|ACR43467.1| | | | | |
|  | gi|347837504|emb|CCD52076.1| | 210 | 35565 |  | hypothetical protein BofuT4\_P082350.1 [Botrytis cinerea T4] |
|  |  | | | | |
|  | gi|347837689|emb|CCD52261.1| | 26 | 71106 | 2.1 | similar to ferric reductase like transmembrane component [Botrytis cinerea T4] |
|  |  | | | | |

---

### 3

|  |  | Accession | Score | Description |
| --- | --- | --- | --- | --- |
|  | 1 | gi|154693286|gb|EDN93024.1| | 525 | hypothetical protein SS1G\_08889 [Sclerotinia sclerotiorum 1980 UF-70] |

|  |  | Score | Mass | Matches | Sequences | emPAI |  |
| --- | --- | --- | --- | --- | --- | --- | --- |
| 3.1 | gi|154693286|gb|EDN93024.1| | 525 | 81000 | 37 (23) | 11 (9) | 0.74 |  |
|  | hypothetical protein SS1G\_08889 [Sclerotinia sclerotiorum 1980 UF-70] | | | | | | |
|  | 2 samesets of gi|154693286|gb|EDN93024.1| | | | | | | |
|  | gi|156048316|ref|XP\_001590125.1| | 525 | 81000 | 37 (23) | 11 (9) | 0.74 |  |
|  | hypothetical protein SS1G\_08889 [Sclerotinia sclerotiorum 1980 UF-70] | | | | | | |
|  | gi|1095458308|gb|APA15259.1| | 525 | 81000 | 37 (23) | 11 (9) | 0.74 |  |
|  | hypothetical protein sscle\_14g100290 [Sclerotinia sclerotiorum 1980 UF-70] | | | | | | |

|  |  | Score | Mass | Matches | Sequences | emPAI |  |
| --- | --- | --- | --- | --- | --- | --- | --- |
| 3.1 | gi|154693286|gb|EDN93024.1| | 525 | 81000 | 37 (23) | 11 (9) | 0.74 | hypothetical protein SS1G\_08889 [Sclerotinia sclerotiorum 1980 UF-70] |
|  |  | | | | | | |
|  | 2 samesets of gi|154693286|gb|EDN93024.1| | | | | | | |
|  | gi|156048316|ref|XP\_001590125.1| | 525 | 81000 | 37 (23) | 11 (9) | 0.74 | hypothetical protein SS1G\_08889 [Sclerotinia sclerotiorum 1980 UF-70] |
|  |  | | | | | | |
|  | gi|1095458308|gb|APA15259.1| | 525 | 81000 | 37 (23) | 11 (9) | 0.74 | hypothetical protein sscle\_14g100290 [Sclerotinia sclerotiorum 1980 UF-70] |
|  |  | | | | | | |

#### 37 peptide matches (18 non-duplicate, 19 duplicate)

Auto-fit to window

| Query | Dupes | Observed | Mr(expt) | Mr(calc) | ppm | M | Score | Expect | Rank | U | Peptide |
| --- | --- | --- | --- | --- | --- | --- | --- | --- | --- | --- | --- |

| Query | Dupes | Observed | Mr(expt) | Mr(calc) | ppm | M | Score | Expect | Rank | U | Peptide |
| --- | --- | --- | --- | --- | --- | --- | --- | --- | --- | --- | --- |
| Query | Dupes | Observed | Mr(expt) | Mr(calc) | ppm | M | Score | Expect | Rank | U | Peptide |
| --- | --- | --- | --- | --- | --- | --- | --- | --- | --- | --- | --- |
| 37 | 1 | 359.7103 | 717.4060 | 717.4061 | -0.20 | 0 | 31 | 0.085 | 1Score **> 33** indicates **identity** | U | K.LDFVPK.R |
| 65 | 1 | 364.6859 | 727.3573 | 727.3575 | -0.17 | 0 | 22 | 0.095 | 1Score **> 27** indicates **identity** Score **> 25** indicates **homology** | U | K.SMFISK.L + Oxidation (M) |
| 1164 | 1 | 464.2274 | 926.4403 | 926.4399 | 0.42 | 0 | 35 | 0.0058 | 1Score **> 30** indicates **identity** Score **> 25** indicates **homology** | U | K.WGVWDHK.F |
| 2204 | 1 | 527.7831 | 1053.5517 | 1053.5529 | -1.09 | 0 | 57 | 5.3e-006 | 1Score **> 31** indicates **identity** Score **> 16** indicates **homology** | U | R.STFLMTVAGK.V |
| 2353 | 2 | 535.7813 | 1069.5479 | 1069.5478 | 0.15 | 0 | 48 | 0.00047 | 1Score **> 31** indicates **identity** Score **> 27** indicates **homology** | U | R.STFLMTVAGK.V + Oxidation (M) |
| 2427 | 1 | 538.7925 | 1075.5705 | 1075.5695 | 0.91 | 0 | 75 | 4.7e-006 | 1Score **> 34** indicates **identity** | U | K.GIIAIEAMSR.I + Oxidation (M) |
| 5285 | 3 | 665.3627 | 1328.7109 | 1328.7089 | 1.54 | 0 | 51 | 0.0011 | 1Score **> 34** indicates **identity** | U | K.LATWIGVTPTDR.A |
| 8516 |  | 847.4213 | 1692.8280 | 1692.8254 | 1.53 | 0 | 41 | 0.014 | 1Score **> 35** indicates **identity** | U | K.AVNGMSFQSGQDVVVR.G |
| 8520 |  | 847.9128 | 1693.8110 | 1693.8094 | 0.96 | 0 | 25 | 0.56 | 1Score **> 35** indicates **identity** | U | K.AVNGMSFQSGQDVVVR.G + Deamidated (NQ) |
| 8639 | 3 | 855.4171 | 1708.8195 | 1708.8203 | -0.44 | 0 | 92 | 2.6e-009 | 1Score **> 35** indicates **identity** Score **> 19** indicates **homology** | U | K.AVNGMSFQSGQDVVVR.G + Oxidation (M) |
| 8657 | 2 | 855.9091 | 1709.8036 | 1709.8043 | -0.43 | 0 | 67 | 2.4e-006 | 1Score **> 34** indicates **identity** Score **> 23** indicates **homology** | U | K.AVNGMSFQSGQDVVVR.G + Deamidated (NQ); Oxidation (M) |
| 8662 | 1 | 856.4025 | 1710.7904 | 1710.7883 | 1.21 | 0 | 37 | 0.025 | 1Score **> 33** indicates **identity** | U | K.AVNGMSFQSGQDVVVR.G + 2 Deamidated (NQ); Oxidation (M) |
| 12783 | 1 | 752.7229 | 2255.1469 | 2255.1434 | 1.55 | 0 | 37 | 0.00049 | 1Score **> 35** indicates **identity** Score **> 16** indicates **homology** | U | K.FAVDSLAAGGQDYLTITSLSAR.Q |
| 12784 |  | 1128.5809 | 2255.1473 | 2255.1434 | 1.75 | 0 | 49 | 0.0019 | 1Score **> 34** indicates **identity** | U | K.FAVDSLAAGGQDYLTITSLSAR.Q |
| 13063 | 1 | 1152.0801 | 2302.1456 | 2302.1456 | -0.0096 | 0 | 41 | 0.013 | 1Score **> 35** indicates **identity** | U | R.QAFGAVQLCGTPDKPYYFLK.E |
| 13916 |  | 823.7636 | 2468.2688 | 2468.2587 | 4.10 | 0 | 22 | 0.012 | 1Score **> 34** indicates **identity** Score **> 15** indicates **homology** | U | K.LAYQDSTGHGLLYNLYADTLLK.L |
| 15265 | 1 | 1357.1290 | 2712.2435 | 2712.2377 | 2.14 | 0 | 38 | 0.029 | 1Score **> 35** indicates **identity** | U | R.IYDMQSEYYPTIANAYGVQLDSR.N + Oxidation (M) |
| 15268 |  | 905.0889 | 2712.2448 | 2712.2377 | 2.60 | 0 | 10 | 1 | 1Score **> 35** indicates **identity** Score **> 22** indicates **homology** | U | R.IYDMQSEYYPTIANAYGVQLDSR.N + Oxidation (M) |

#### 1 subset or intersection (1 subset protein in total)

|  |  | Score | Mass | Subset of |  |
| --- | --- | --- | --- | --- | --- |
|  | gi|347832971|emb|CCD48668.1| | 163 | 81430 | 3.1 |  |
|  | similar to glutaminase [Botrytis cinerea T4] | | | | |

|  |  | Score | Mass | Subset of |  |
| --- | --- | --- | --- | --- | --- |
|  | gi|347832971|emb|CCD48668.1| | 163 | 81430 | 3.1 | similar to glutaminase [Botrytis cinerea T4] |
|  |  | | | | |

---

### 4

|  |  | Accession | Score | Description |
| --- | --- | --- | --- | --- |
|  | 1 | gi|347830055|emb|CCD45752.1| | 513 | glycoside hydrolase family 92 protein [Botrytis cinerea T4] |
|  | 2 | gi\|1095450133\|gb\|APA07096.1\| | 338 | hypothetical protein sscle\_02g018660 [Sclerotinia sclerotiorum 1980 UF-70] |
|  |  |  |  |  |
| Cut threshold Threshold (**0**): | | | | |

|  |  | Score | Mass | Matches | Sequences | emPAI |  |
| --- | --- | --- | --- | --- | --- | --- | --- |
| 4.1 | gi|347830055|emb|CCD45752.1| | 513 | 86256 | 16 (11) | 7 (4) | 0.16 |  |
|  | glycoside hydrolase family 92 protein [Botrytis cinerea T4] | | | | | | |
| 4.2 | gi|1095450133|gb|APA07096.1| | 338 | 85504 | 17 (11) | 9 (6) | 0.25 |  |
|  | hypothetical protein sscle\_02g018660 [Sclerotinia sclerotiorum 1980 UF-70] | | | | | | |
|  | 2 samesets of gi|1095450133|gb|APA07096.1| | | | | | | |
|  | gi|154701986|gb|EDO01725.1| | 338 | 85030 | 17 (11) | 9 (6) | 0.25 |  |
|  | hypothetical protein SS1G\_04200 [Sclerotinia sclerotiorum 1980 UF-70] | | | | | | |
|  | gi|156056939|ref|XP\_001594393.1| | 338 | 85030 | 17 (11) | 9 (6) | 0.25 |  |
|  | hypothetical protein SS1G\_04200 [Sclerotinia sclerotiorum 1980 UF-70] | | | | | | |
|  | | | | | | |

|  |  | Score | Mass | Matches | Sequences | emPAI |  |
| --- | --- | --- | --- | --- | --- | --- | --- |
| 4.1 | gi|347830055|emb|CCD45752.1| | 513 | 86256 | 16 (11) | 7 (4) | 0.16 | glycoside hydrolase family 92 protein [Botrytis cinerea T4] |
|  |  | | | | | | |
| 4.2 | gi|1095450133|gb|APA07096.1| | 338 | 85504 | 17 (11) | 9 (6) | 0.25 | hypothetical protein sscle\_02g018660 [Sclerotinia sclerotiorum 1980 UF-70] |
|  |  | | | | | | |
|  | 2 samesets of gi|1095450133|gb|APA07096.1| | | | | | | |
|  | gi|154701986|gb|EDO01725.1| | 338 | 85030 | 17 (11) | 9 (6) | 0.25 | hypothetical protein SS1G\_04200 [Sclerotinia sclerotiorum 1980 UF-70] |
|  |  | | | | | | |
|  | gi|156056939|ref|XP\_001594393.1| | 338 | 85030 | 17 (11) | 9 (6) | 0.25 | hypothetical protein SS1G\_04200 [Sclerotinia sclerotiorum 1980 UF-70] |
|  |  | | | | | | |
|  | | | | | | |

#### 23 peptide matches (13 non-duplicate, 10 duplicate)

Auto-fit to window

| Query | Dupes | Observed | Mr(expt) | Mr(calc) | ppm | M | Score | Expect | Rank | U | 1 | 2 | Peptide |
| --- | --- | --- | --- | --- | --- | --- | --- | --- | --- | --- | --- | --- | --- |

| Query | Dupes | Observed | Mr(expt) | Mr(calc) | ppm | M | Score | Expect | Rank | U | 1 | 2 | Peptide |
| --- | --- | --- | --- | --- | --- | --- | --- | --- | --- | --- | --- | --- | --- |
| Query | Dupes | Observed | Mr(expt) | Mr(calc) | ppm | M | Score | Expect | Rank | U | 1 | 2 | Peptide |
| --- | --- | --- | --- | --- | --- | --- | --- | --- | --- | --- | --- | --- | --- |
| 884 | 1 | 445.2625 | 888.5104 | 888.5069 | 3.95 | 0 | 42 | 0.0019 | 1Score **> 32** indicates **identity** Score **> 27** indicates **homology** | U |  |  | R.VGISWISK.E |
| 988 |  | 451.7602 | 901.5058 | 901.5021 | 4.10 | 0 | 33 | 0.07 | 1Score **> 34** indicates **identity** | U |  |  | R.SLIDIWR |
| 2431 |  | 539.2246 | 1076.4345 | 1076.4345 | 0.0074 | 0 | 23 | 0.0072 | 1Score **> 26** indicates **identity** Score **> 15** indicates **homology** | U |  |  | R.HDGYMPDAR.S + Oxidation (M) |
| 3842 |  | 601.8176 | 1201.6206 | 1201.6204 | 0.19 | 0 | 42 | 0.0026 | 1Score **> 34** indicates **identity** Score **> 28** indicates **homology** | U |  |  | K.SVWNNQVLSR.I |
| 3866 |  | 602.8071 | 1203.5996 | 1203.5884 | 9.31 | 0 | 4 | 1.5 | 4Score **> 34** indicates **identity** Score **> 18** indicates **homology** | U |  |  | K.SVWNNQVLSR.I + 2 Deamidated (NQ) |
| 3866 |  | 602.8071 | 1203.5996 | 1203.5884 | 9.30 | 0 | 1 | 2.7 | 7Score **> 34** indicates **identity** Score **> 18** indicates **homology** | U |  |  | K.SVWNdeVLSR.I |
| 3878 |  | 603.3080 | 1204.6015 | 1204.5724 | 24.2 | 0 | 2 | 1 | 3Score **> 34** indicates **identity** Score **> 14** indicates **homology** | U |  |  | K.SVWNdeVLSR.I + Deamidated (NQ) |
| 5117 |  | 656.3196 | 1310.6247 | 1310.6255 | -0.60 | 0 | 27 | 0.007 | 1Score **> 32** indicates **identity** Score **> 18** indicates **homology** | U |  |  | R.AVPDQGSVGYYR.A |
| 7609 | 1 | 791.8676 | 1581.7205 | 1581.7246 | -2.54 | 0 | 65 | 3.4e-005 | 1Score **> 33** indicates **identity** | U |  |  | R.GQINWNDGYAAMVK.D + Oxidation (M) |
| 8745 | 3 | 861.9179 | 1721.8213 | 1721.8220 | -0.45 | 0 | 101 | 7.8e-010 | 1Score **> 35** indicates **identity** Score **> 22** indicates **homology** | U |  |  | R.TQGGSNADNVLADAYVK.G |
| 10258 | 1 | 949.4363 | 1896.8581 | 1896.8676 | -4.99 | 1 | 1 | 1.1 | 5Score **> 33** indicates **identity** Score **> 14** indicates **homology** | U |  |  | K.GVRGQINWdDGYAAMVK.D + 2 Deamidated (NQ); Oxidation (M) |
| 13510 | 3 | 1196.5623 | 2391.1100 | 2391.1078 | 0.91 | 0 | 110 | 1.9e-009 | 1Score **> 35** indicates **identity** | U |  |  | K.TLTITSTGGDGNGDSNYYVQSLK.V + Deamidated (NQ) |
| 24303 |  | 1651.7690 | 4952.2853 | 4952.3736 | -17.8 | 1 | 3 | 0.99 | 1Score **> 34** indicates **identity** Score **> 16** indicates **homology** | U |  |  | K.SVWNNQVLSRITTTSTNSTNLGLLYTSMYFMNLLPTNQTGENPK.W + 4 Deamidated (NQ) |

---

### 5

|  |  | Accession | Score | Description |
| --- | --- | --- | --- | --- |
|  | 1 | gi|154699986|gb|EDN99724.1| | 463 | hypothetical protein SS1G\_02582 [Sclerotinia sclerotiorum 1980 UF-70] |

|  |  | Score | Mass | Matches | Sequences | emPAI |  |
| --- | --- | --- | --- | --- | --- | --- | --- |
| 5.1 | gi|154699986|gb|EDN99724.1| | 463 | 82732 | 17 (14) | 9 (9) | 0.42 |  |
|  | hypothetical protein SS1G\_02582 [Sclerotinia sclerotiorum 1980 UF-70] | | | | | | |
|  | 2 samesets of gi|154699986|gb|EDN99724.1| | | | | | | |
|  | gi|156060879|ref|XP\_001596362.1| | 463 | 82732 | 17 (14) | 9 (9) | 0.42 |  |
|  | hypothetical protein SS1G\_02582 [Sclerotinia sclerotiorum 1980 UF-70] | | | | | | |
|  | gi|1095451833|gb|APA08794.1| | 463 | 86754 | 17 (14) | 9 (9) | 0.40 |  |
|  | hypothetical protein sscle\_04g035640 [Sclerotinia sclerotiorum 1980 UF-70] | | | | | | |

|  |  | Score | Mass | Matches | Sequences | emPAI |  |
| --- | --- | --- | --- | --- | --- | --- | --- |
| 5.1 | gi|154699986|gb|EDN99724.1| | 463 | 82732 | 17 (14) | 9 (9) | 0.42 | hypothetical protein SS1G\_02582 [Sclerotinia sclerotiorum 1980 UF-70] |
|  |  | | | | | | |
|  | 2 samesets of gi|154699986|gb|EDN99724.1| | | | | | | |
|  | gi|156060879|ref|XP\_001596362.1| | 463 | 82732 | 17 (14) | 9 (9) | 0.42 | hypothetical protein SS1G\_02582 [Sclerotinia sclerotiorum 1980 UF-70] |
|  |  | | | | | | |
|  | gi|1095451833|gb|APA08794.1| | 463 | 86754 | 17 (14) | 9 (9) | 0.40 | hypothetical protein sscle\_04g035640 [Sclerotinia sclerotiorum 1980 UF-70] |
|  |  | | | | | | |

#### 17 peptide matches (11 non-duplicate, 6 duplicate)

Auto-fit to window

| Query | Dupes | Observed | Mr(expt) | Mr(calc) | ppm | M | Score | Expect | Rank | U | Peptide |
| --- | --- | --- | --- | --- | --- | --- | --- | --- | --- | --- | --- |

| Query | Dupes | Observed | Mr(expt) | Mr(calc) | ppm | M | Score | Expect | Rank | U | Peptide |
| --- | --- | --- | --- | --- | --- | --- | --- | --- | --- | --- | --- |
| Query | Dupes | Observed | Mr(expt) | Mr(calc) | ppm | M | Score | Expect | Rank | U | Peptide |
| --- | --- | --- | --- | --- | --- | --- | --- | --- | --- | --- | --- |
| 54 |  | 363.1950 | 724.3755 | 724.3755 | -0.11 | 0 | 28 | 0.025 | 1Score **> 28** indicates **identity** Score **> 24** indicates **homology** | U | K.LSSGSFK.A |
| 3362 | 1 | 581.7907 | 1161.5669 | 1161.5666 | 0.25 | 0 | 42 | 0.0026 | 1Score **> 35** indicates **identity** Score **> 29** indicates **homology** | U | K.ELPELAGDYR.T |
| 3743 | 1 | 597.3301 | 1192.6456 | 1192.6452 | 0.35 | 0 | 84 | 3.2e-007 | 1Score **> 32** indicates **identity** Score **> 32** indicates **homology** | U | R.FSTLATSTIPR.L |
| 6256 |  | 716.3920 | 1430.7694 | 1430.7657 | 2.60 | 0 | 117 | 1.7e-010 | 1Score **> 33** indicates **identity** Score **> 32** indicates **homology** | U | K.APTLEALLYDPTK.A |
| 8763 | 1 | 862.8932 | 1723.7718 | 1723.7723 | -0.29 | 0 | 81 | 4.5e-008 | 1Score **> 33** indicates **identity** Score **> 20** indicates **homology** | U | K.ADGSTLDVTFNCPAGAK.A + Deamidated (NQ) |
| 9207 | 1 | 890.4316 | 1778.8486 | 1778.8475 | 0.62 | 0 | 22 | 0.028 | 1Score **> 35** indicates **identity** Score **> 19** indicates **homology** | U | R.VENYVPPYLSGDNANK.R |
| 9742 |  | 923.9591 | 1845.9035 | 1845.9043 | -0.43 | 0 | 61 | 0.00014 | 1Score **> 35** indicates **identity** | U | R.MLHLDNTGFVAGATQQK.L + Oxidation (M) |
| 9784 |  | 924.9309 | 1847.8473 | 1847.8724 | -13.6 | 0 | 12 | 2.1 | 2Score **> 34** indicates **identity** Score **> 28** indicates **homology** | U | R.MLHLDNTGFVAGATQQK.L + 2 Deamidated (NQ); Oxidation (M) |
| 10054 | 1 | 940.4176 | 1878.8206 | 1878.8595 | -20.7 | 1 | 63 | 1.2e-006 | 1Score **> 32** indicates **identity** Score **> 17** indicates **homology** | U | R.SSTDASLTGKDWSEPGNK.L |
| 12117 | 1 | 1079.0186 | 2156.0225 | 2156.0208 | 0.81 | 0 | 59 | 0.0001 | 1Score **> 35** indicates **identity** Score **> 31** indicates **homology** | U | R.IQPLSANPTWELDSMPEGR.G + Oxidation (M) |
| 12123 |  | 719.6828 | 2156.0266 | 2156.0208 | 2.68 | 0 | 3 | 1 | 1Score **> 35** indicates **identity** Score **> 16** indicates **homology** | U | R.IQPLSANPTWELDSMPEGR.G + Oxidation (M) |

#### 1 subset or intersection (1 subset protein in total)

|  |  | Score | Mass | Subset of |  |
| --- | --- | --- | --- | --- | --- |
|  | gi|347833387|emb|CCD49084.1| | 254 | 86628 | 5.1 |  |
|  | carbohydrate-Binding Module family 18 protein [Botrytis cinerea T4] | | | | |

|  |  | Score | Mass | Subset of |  |
| --- | --- | --- | --- | --- | --- |
|  | gi|347833387|emb|CCD49084.1| | 254 | 86628 | 5.1 | carbohydrate-Binding Module family 18 protein [Botrytis cinerea T4] |
|  |  | | | | |

---

### 6

|  |  | Accession | Score | Description |
| --- | --- | --- | --- | --- |
|  | 1 | gi|154697664|gb|EDN97402.1| | 448 | hypothetical protein SS1G\_11927 [Sclerotinia sclerotiorum 1980 UF-70] |

|  |  | Score | Mass | Matches | Sequences | emPAI |  |
| --- | --- | --- | --- | --- | --- | --- | --- |
| 6.1 | gi|154697664|gb|EDN97402.1| | 448 | 70689 | 14 (11) | 4 (4) | 0.37 |  |
|  | hypothetical protein SS1G\_11927 [Sclerotinia sclerotiorum 1980 UF-70] | | | | | | |
|  | 2 samesets of gi|154697664|gb|EDN97402.1| | | | | | | |
|  | gi|156039581|ref|XP\_001586898.1| | 448 | 70689 | 14 (11) | 4 (4) | 0.37 |  |
|  | hypothetical protein SS1G\_11927 [Sclerotinia sclerotiorum 1980 UF-70] | | | | | | |
|  | gi|1095457316|gb|APA14269.1| | 448 | 70689 | 14 (11) | 4 (4) | 0.37 |  |
|  | hypothetical protein sscle\_12g090390 [Sclerotinia sclerotiorum 1980 UF-70] | | | | | | |

|  |  | Score | Mass | Matches | Sequences | emPAI |  |
| --- | --- | --- | --- | --- | --- | --- | --- |
| 6.1 | gi|154697664|gb|EDN97402.1| | 448 | 70689 | 14 (11) | 4 (4) | 0.37 | hypothetical protein SS1G\_11927 [Sclerotinia sclerotiorum 1980 UF-70] |
|  |  | | | | | | |
|  | 2 samesets of gi|154697664|gb|EDN97402.1| | | | | | | |
|  | gi|156039581|ref|XP\_001586898.1| | 448 | 70689 | 14 (11) | 4 (4) | 0.37 | hypothetical protein SS1G\_11927 [Sclerotinia sclerotiorum 1980 UF-70] |
|  |  | | | | | | |
|  | gi|1095457316|gb|APA14269.1| | 448 | 70689 | 14 (11) | 4 (4) | 0.37 | hypothetical protein sscle\_12g090390 [Sclerotinia sclerotiorum 1980 UF-70] |
|  |  | | | | | | |

#### 14 peptide matches (10 non-duplicate, 4 duplicate)

Auto-fit to window

| Query | Dupes | Observed | Mr(expt) | Mr(calc) | ppm | M | Score | Expect | Rank | U | Peptide |
| --- | --- | --- | --- | --- | --- | --- | --- | --- | --- | --- | --- |

| Query | Dupes | Observed | Mr(expt) | Mr(calc) | ppm | M | Score | Expect | Rank | U | Peptide |
| --- | --- | --- | --- | --- | --- | --- | --- | --- | --- | --- | --- |
| 1567 |  | 490.2279 | 978.4412 | 978.4406 | 0.56 | 0 | 38 | 0.0048 | 1Score **> 32** indicates **identity** Score **> 28** indicates **homology** | U | K.LDYNNPSR.R + Deamidated (NQ) |
| 2512 |  | 543.2772 | 1084.5399 | 1084.5401 | -0.15 | 0 | 24 | 0.021 | 1Score **> 31** indicates **identity** Score **> 19** indicates **homology** | U | K.YTTNTITSGK.K |
| 9299 |  | 895.8873 | 1789.7600 | 1789.7618 | -0.99 | 0 | 82 | 3.4e-007 | 1Score **> 30** indicates **identity** | U | R.ACWGNGFSIATDFDTK.N + Deamidated (NQ) |
| 9516 | 1 | 908.4237 | 1814.8329 | 1814.8329 | -0.035 | 0 | 100 | 6.2e-010 | 1Score **> 33** indicates **identity** Score **> 20** indicates **homology** | U | R.AEVQNGCGINNNNGNIK.S |
| 9522 | 2 | 908.9150 | 1815.8154 | 1815.8169 | -0.84 | 0 | 90 | 1.1e-007 | 1Score **> 33** indicates **identity** | U | R.AEVQNGCGINNNNGNIK.S + Deamidated (NQ) |
| 9523 |  | 908.9153 | 1815.8161 | 1815.8169 | -0.45 | 0 | 72 | 9.4e-007 | 1Score **> 33** indicates **identity** Score **> 24** indicates **homology** | U | R.AEVQNGCGINNNNGNIK.S + Deamidated (NQ) |
| 9525 | 1 | 909.4073 | 1816.8000 | 1816.8009 | -0.51 | 0 | 65 | 2.7e-005 | 1Score **> 32** indicates **identity** | U | R.AEVQNGCGINNNNGNIK.S + 2 Deamidated (NQ) |
| 9526 |  | 909.4073 | 1816.8001 | 1816.8009 | -0.44 | 0 | 43 | 0.00018 | 1Score **> 32** indicates **identity** Score **> 18** indicates **homology** | U | R.AEVQNGCGINNNNGNIK.S + 2 Deamidated (NQ) |
| 9528 |  | 909.4078 | 1816.8010 | 1816.8009 | 0.032 | 0 | 60 | 2.6e-006 | 1Score **> 32** indicates **identity** Score **> 17** indicates **homology** | U | R.AEVQNGCGINNNNGNIK.S + 2 Deamidated (NQ) |
| 9534 |  | 909.8947 | 1817.7749 | 1817.7850 | -5.55 | 0 | 59 | 1.1e-005 | 1Score **> 30** indicates **identity** Score **> 22** indicates **homology** | U | R.AEVQNGCGINNNNGNIK.S + 3 Deamidated (NQ) |

#### 1 subset or intersection (1 subset protein in total)

|  |  | Score | Mass | Subset of |  |
| --- | --- | --- | --- | --- | --- |
|  | gi|347835694|emb|CCD50266.1| | 82 | 71674 | 6.1 |  |
|  | hypothetical protein BofuT4\_P092250.1 [Botrytis cinerea T4] | | | | |

|  |  | Score | Mass | Subset of |  |
| --- | --- | --- | --- | --- | --- |
|  | gi|347835694|emb|CCD50266.1| | 82 | 71674 | 6.1 | hypothetical protein BofuT4\_P092250.1 [Botrytis cinerea T4] |
|  |  | | | | |

---

### 7

|  |  | Accession | Score | Description |
| --- | --- | --- | --- | --- |
|  | 1 | gi|154705171|gb|EDO04910.1| | 260 | hypothetical protein SS1G\_07393 [Sclerotinia sclerotiorum 1980 UF-70] |

|  |  | Score | Mass | Matches | Sequences | emPAI |  |
| --- | --- | --- | --- | --- | --- | --- | --- |
| 7.1 | gi|154705171|gb|EDO04910.1| | 260 | 83872 | 12 (8) | 7 (3) | 0.12 |  |
|  | hypothetical protein SS1G\_07393 [Sclerotinia sclerotiorum 1980 UF-70] | | | | | | |
|  | 2 samesets of gi|154705171|gb|EDO04910.1| | | | | | | |
|  | gi|156051972|ref|XP\_001591947.1| | 260 | 83872 | 12 (8) | 7 (3) | 0.12 |  |
|  | hypothetical protein SS1G\_07393 [Sclerotinia sclerotiorum 1980 UF-70] | | | | | | |
|  | gi|1095453223|gb|APA10182.1| | 260 | 96180 | 12 (8) | 7 (3) | 0.11 |  |
|  | hypothetical protein sscle\_06g049520 [Sclerotinia sclerotiorum 1980 UF-70] | | | | | | |

|  |  | Score | Mass | Matches | Sequences | emPAI |  |
| --- | --- | --- | --- | --- | --- | --- | --- |
| 7.1 | gi|154705171|gb|EDO04910.1| | 260 | 83872 | 12 (8) | 7 (3) | 0.12 | hypothetical protein SS1G\_07393 [Sclerotinia sclerotiorum 1980 UF-70] |
|  |  | | | | | | |
|  | 2 samesets of gi|154705171|gb|EDO04910.1| | | | | | | |
|  | gi|156051972|ref|XP\_001591947.1| | 260 | 83872 | 12 (8) | 7 (3) | 0.12 | hypothetical protein SS1G\_07393 [Sclerotinia sclerotiorum 1980 UF-70] |
|  |  | | | | | | |
|  | gi|1095453223|gb|APA10182.1| | 260 | 96180 | 12 (8) | 7 (3) | 0.11 | hypothetical protein sscle\_06g049520 [Sclerotinia sclerotiorum 1980 UF-70] |
|  |  | | | | | | |

#### 12 peptide matches (7 non-duplicate, 5 duplicate)

Auto-fit to window

| Query | Dupes | Observed | Mr(expt) | Mr(calc) | ppm | M | Score | Expect | Rank | U | Peptide |
| --- | --- | --- | --- | --- | --- | --- | --- | --- | --- | --- | --- |

| Query | Dupes | Observed | Mr(expt) | Mr(calc) | ppm | M | Score | Expect | Rank | U | Peptide |
| --- | --- | --- | --- | --- | --- | --- | --- | --- | --- | --- | --- |
| 685 |  | 430.7351 | 859.4555 | 859.4552 | 0.42 | 0 | 18 | 0.75 | 1Score **> 35** indicates **identity** Score **> 29** indicates **homology** | U | K.IASWAQGK.V |
| 995 |  | 452.7587 | 903.5028 | 903.5025 | 0.28 | 0 | 20 | 0.32 | 1Score **> 33** indicates **identity** Score **> 28** indicates **homology** | U | K.STILAGNTK.I |
| 1766 |  | 503.7338 | 1005.4531 | 1005.4516 | 1.53 | 0 | 25 | 0.036 | 1Score **> 31** indicates **identity** Score **> 23** indicates **homology** | U | K.GFNDQSNPK.A |
| 8390 | 2 | 838.3751 | 1674.7357 | 1674.7420 | -3.79 | 0 | 55 | 0.00025 | 1Score **> 31** indicates **identity** | U | K.EASQGSVGMWDVHTR.I + Oxidation (M) |
| 8867 |  | 868.9224 | 1735.8303 | 1735.8265 | 2.20 | 0 | 5 | 1 | 1Score **> 35** indicates **identity** Score **> 18** indicates **homology** | U | K.GDGVTDDTAAIQAIFDK.A |
| 10250 |  | 632.6757 | 1895.0052 | 1895.0040 | 0.60 | 0 | 5 | 1 | 1Score **> 33** indicates **identity** Score **> 17** indicates **homology** |  | R.TKPQYETLPASSFLSVK.S |
| 13459 | 3 | 1189.5618 | 2377.1090 | 2377.1146 | -2.36 | 0 | 85 | 1.4e-007 | 1Score **> 35** indicates **identity** Score **> 29** indicates **homology** | U | K.DFGAVGDGVTDDTAAINAAISAGNR.C |

---

### 8

|  |  | Accession | Score | Description |
| --- | --- | --- | --- | --- |
|  | 1 | gi|154703817|gb|EDO03556.1| | 231 | glucan 1,3-beta-glucosidase [Sclerotinia sclerotiorum 1980 UF-70] |
|  | 2 | gi\|347830866\|emb\|CCD46563.1\| | 115 | glycoside hydrolase family 5 protein, partial sequence [Botrytis cinerea T4] |
|  |  |  |  |  |
| Cut threshold Threshold (**0**): | | | | |

|  |  | Score | Mass | Matches | Sequences | emPAI |  |
| --- | --- | --- | --- | --- | --- | --- | --- |
| 8.1 | gi|154703817|gb|EDO03556.1| | 231 | 46611 | 20 (10) | 9 (6) | 0.61 |  |
|  | glucan 1,3-beta-glucosidase [Sclerotinia sclerotiorum 1980 UF-70] | | | | | | |
|  | 2 samesets of gi|154703817|gb|EDO03556.1| | | | | | | |
|  | gi|156054378|ref|XP\_001593115.1| | 231 | 46611 | 20 (10) | 9 (6) | 0.61 |  |
|  | glucan 1,3-beta-glucosidase [Sclerotinia sclerotiorum 1980 UF-70] | | | | | | |
|  | gi|1095452802|gb|APA09762.1| | 231 | 46611 | 20 (10) | 9 (6) | 0.61 |  |
|  | hypothetical protein sscle\_05g045320 [Sclerotinia sclerotiorum 1980 UF-70] | | | | | | |
| 8.2 | gi|347830866|emb|CCD46563.1| | 115 | 27955 | 14 (5) | 5 (2) | 0.40 |  |
|  | glycoside hydrolase family 5 protein, partial sequence [Botrytis cinerea T4] | | | | | | |
|  | | | | | | |

|  |  | Score | Mass | Matches | Sequences | emPAI |  |
| --- | --- | --- | --- | --- | --- | --- | --- |
| 8.1 | gi|154703817|gb|EDO03556.1| | 231 | 46611 | 20 (10) | 9 (6) | 0.61 | glucan 1,3-beta-glucosidase [Sclerotinia sclerotiorum 1980 UF-70] |
|  |  | | | | | | |
|  | 2 samesets of gi|154703817|gb|EDO03556.1| | | | | | | |
|  | gi|156054378|ref|XP\_001593115.1| | 231 | 46611 | 20 (10) | 9 (6) | 0.61 | glucan 1,3-beta-glucosidase [Sclerotinia sclerotiorum 1980 UF-70] |
|  |  | | | | | | |
|  | gi|1095452802|gb|APA09762.1| | 231 | 46611 | 20 (10) | 9 (6) | 0.61 | hypothetical protein sscle\_05g045320 [Sclerotinia sclerotiorum 1980 UF-70] |
|  |  | | | | | | |
| 8.2 | gi|347830866|emb|CCD46563.1| | 115 | 27955 | 14 (5) | 5 (2) | 0.40 | glycoside hydrolase family 5 protein, partial sequence [Botrytis cinerea T4] |
|  |  | | | | | | |
|  | | | | | | |

#### 31 peptide matches (18 non-duplicate, 13 duplicate)

Auto-fit to window

| Query | Dupes | Observed | Mr(expt) | Mr(calc) | ppm | M | Score | Expect | Rank | U | 1 | 2 | Peptide |
| --- | --- | --- | --- | --- | --- | --- | --- | --- | --- | --- | --- | --- | --- |

| Query | Dupes | Observed | Mr(expt) | Mr(calc) | ppm | M | Score | Expect | Rank | U | 1 | 2 | Peptide |
| --- | --- | --- | --- | --- | --- | --- | --- | --- | --- | --- | --- | --- | --- |
| Query | Dupes | Observed | Mr(expt) | Mr(calc) | ppm | M | Score | Expect | Rank | U | 1 | 2 | Peptide |
| --- | --- | --- | --- | --- | --- | --- | --- | --- | --- | --- | --- | --- | --- |
| 2533 |  | 544.2562 | 1086.4979 | 1086.4982 | -0.25 | 0 | 2 | 1.3 | 3Score **> 30** indicates **identity** Score **> 16** indicates **homology** | U |  |  | K.GLSFDYNGSK.V |
| 2580 |  | 546.7848 | 1091.5550 | 1091.5512 | 3.47 | 0 | 28 | 0.0031 | 1Score **> 34** indicates **identity** Score **> 16** indicates **homology** | U |  |  | K.WLNGFGVGSR.Y |
| 2592 | 3 | 547.2763 | 1092.5381 | 1092.5352 | 2.58 | 0 | 40 | 0.00064 | 1Score **> 34** indicates **identity** Score **> 20** indicates **homology** | U |  |  | K.WLNGFGVGSR.Y + Deamidated (NQ) |
| 3248 | 2 | 576.8408 | 1151.6671 | 1151.6662 | 0.73 | 0 | 24 | 0.14 | 1Score **> 28** indicates **identity** | U |  |  | R.AGLIPQPLTSR.K |
| 5033 | 1 | 652.8274 | 1303.6402 | 1303.6409 | -0.48 | 0 | 38 | 0.021 | 1Score **> 34** indicates **identity** | U |  |  | K.GPITWTQGDTTK.Q |
| 5163 | 1 | 658.3604 | 1314.7061 | 1314.7031 | 2.31 | 0 | 75 | 3.9e-006 | 1Score **> 34** indicates **identity** | U |  |  | K.DVGTVDGLLAIDK.V |
| 5501 |  | 674.8837 | 1347.7528 | 1347.7510 | 1.31 | 0 | 56 | 7.2e-006 | 1Score **> 31** indicates **identity** Score **> 17** indicates **homology** | U |  |  | K.QTLAAIQTLAYR.Y |
| 6258 |  | 716.8746 | 1431.7346 | 1431.7358 | -0.85 | 1 | 55 | 0.00049 | 1Score **> 34** indicates **identity** | U |  |  | R.KGPITWTQGDTTK.Q |
| 6259 |  | 478.2523 | 1431.7351 | 1431.7358 | -0.47 | 1 | 14 | 1 | 1Score **> 34** indicates **identity** Score **> 26** indicates **homology** | U |  |  | R.KGPITWTQGDTTK.Q |
| 6447 |  | 726.3646 | 1450.7147 | 1450.6940 | 14.3 | 1 | 0 | 2.1 | 4Score **> 34** indicates **identity** Score **> 16** indicates **homology** | U |  |  | R.KGPVTWTQGDTTK.Q + Deamidated (NQ); Dioxidation (W) |
| 9146 | 1 | 885.4138 | 1768.8130 | 1768.8091 | 2.21 | 0 | 24 | 0.46 | 1Score **> 33** indicates **identity** | U |  |  | K.WTIVGEFSGAQTDCAK.W |
| 10485 |  | 968.9196 | 1935.8246 | 1935.8309 | -3.27 | 0 | 3 | 1 | 1Score **> 30** indicates **identity** Score **> 15** indicates **homology** | U |  |  | R.YDGSYPGSPAVYGSCQTK.D |
| 11306 |  | 680.6679 | 2038.9819 | 2038.9821 | -0.090 | 0 | 4 | 1 | 1Score **> 35** indicates **identity** Score **> 16** indicates **homology** | U |  |  | K.VILDVHGAPGSQNGFDNSGR.K |
| 11307 | 1 | 1020.4982 | 2038.9819 | 2038.9821 | -0.087 | 0 | 32 | 0.001 | 1Score **> 35** indicates **identity** Score **> 15** indicates **homology** | U |  |  | K.VILDVHGAPGSQNGFDNSGR.K |
| 11310 | 2 | 1020.9899 | 2039.9652 | 2039.9661 | -0.44 | 0 | 47 | 0.0035 | 1Score **> 35** indicates **identity** | U |  |  | K.VILDVHGAPGSQNGFDNSGR.K + Deamidated (NQ) |
| 11313 | 2 | 680.9982 | 2039.9727 | 2039.9661 | 3.23 | 0 | 15 | 1 | 1Score **> 35** indicates **identity** Score **> 28** indicates **homology** | U |  |  | K.VILDVHGAPGSQNGFDNSGR.K + Deamidated (NQ) |
| 17639 |  | 1488.2529 | 2974.4913 | 2974.4634 | 9.39 | 0 | 0 | 1 | 2Score **> 35** indicates **identity** Score **> 13** indicates **homology** | U |  |  | R.YAPATDVVTGIELLNEPANWALDMGAVK.Q + Deamidated (NQ); Oxidation (M) |
| 17640 |  | 992.5055 | 2974.4946 | 2974.4634 | 10.5 | 0 | 25 | 0.64 | 1Score **> 35** indicates **identity** | U |  |  | R.YAPATDVVTGIELLNEPANWALDMGAVK.Q + Deamidated (NQ); Oxidation (M) |

#### 1 subset or intersection (1 subset protein in total)

|  |  | Score | Mass | Subset of |  |
| --- | --- | --- | --- | --- | --- |
|  | gi|347830867|emb|CCD46564.1| | 84 | 16377 | 8.1 |  |
|  | glycoside hydrolase family 5 protein, partial sequence [Botrytis cinerea T4] | | | | |

|  |  | Score | Mass | Subset of |  |
| --- | --- | --- | --- | --- | --- |
|  | gi|347830867|emb|CCD46564.1| | 84 | 16377 | 8.1 | glycoside hydrolase family 5 protein, partial sequence [Botrytis cinerea T4] |
|  |  | | | | |

---

### 9

|  |  | Accession | Score | Description |
| --- | --- | --- | --- | --- |
|  | 1 | gi|154695005|gb|EDN94743.1| | 225 | hypothetical protein SS1G\_10617 [Sclerotinia sclerotiorum 1980 UF-70] |
|  | 2 | gi\|507414541\|emb\|CCD49819.2\| | 55 | glycoside hydrolase family 15 protein [Botrytis cinerea T4] |
|  |  |  |  |  |
| Cut threshold Threshold (**0**): | | | | |

|  |  | Score | Mass | Matches | Sequences | emPAI |  |
| --- | --- | --- | --- | --- | --- | --- | --- |
| 9.1 | gi|154695005|gb|EDN94743.1| | 225 | 72488 | 11 (8) | 4 (2) | 0.09 |  |
|  | hypothetical protein SS1G\_10617 [Sclerotinia sclerotiorum 1980 UF-70] | | | | | | |
|  | 2 samesets of gi|154695005|gb|EDN94743.1| | | | | | | |
|  | gi|156043229|ref|XP\_001588171.1| | 225 | 72488 | 11 (8) | 4 (2) | 0.09 |  |
|  | hypothetical protein SS1G\_10617 [Sclerotinia sclerotiorum 1980 UF-70] | | | | | | |
|  | gi|1095455401|gb|APA12357.1| | 225 | 72488 | 11 (8) | 4 (2) | 0.09 |  |
|  | hypothetical protein sscle\_09g071270 [Sclerotinia sclerotiorum 1980 UF-70] | | | | | | |
| 9.2 | gi|507414541|emb|CCD49819.2| | 55 | 68339 | 7 (5) | 3 (2) | 0.10 |  |
|  | glycoside hydrolase family 15 protein [Botrytis cinerea T4] | | | | | | |
|  | | | | | | |

|  |  | Score | Mass | Matches | Sequences | emPAI |  |
| --- | --- | --- | --- | --- | --- | --- | --- |
| 9.1 | gi|154695005|gb|EDN94743.1| | 225 | 72488 | 11 (8) | 4 (2) | 0.09 | hypothetical protein SS1G\_10617 [Sclerotinia sclerotiorum 1980 UF-70] |
|  |  | | | | | | |
|  | 2 samesets of gi|154695005|gb|EDN94743.1| | | | | | | |
|  | gi|156043229|ref|XP\_001588171.1| | 225 | 72488 | 11 (8) | 4 (2) | 0.09 | hypothetical protein SS1G\_10617 [Sclerotinia sclerotiorum 1980 UF-70] |
|  |  | | | | | | |
|  | gi|1095455401|gb|APA12357.1| | 225 | 72488 | 11 (8) | 4 (2) | 0.09 | hypothetical protein sscle\_09g071270 [Sclerotinia sclerotiorum 1980 UF-70] |
|  |  | | | | | | |
| 9.2 | gi|507414541|emb|CCD49819.2| | 55 | 68339 | 7 (5) | 3 (2) | 0.10 | glycoside hydrolase family 15 protein [Botrytis cinerea T4] |
|  |  | | | | | | |
|  | | | | | | |

#### 13 peptide matches (5 non-duplicate, 8 duplicate)

Auto-fit to window

| Query | Dupes | Observed | Mr(expt) | Mr(calc) | ppm | M | Score | Expect | Rank | U | 1 | 2 | Peptide |
| --- | --- | --- | --- | --- | --- | --- | --- | --- | --- | --- | --- | --- | --- |

| Query | Dupes | Observed | Mr(expt) | Mr(calc) | ppm | M | Score | Expect | Rank | U | 1 | 2 | Peptide |
| --- | --- | --- | --- | --- | --- | --- | --- | --- | --- | --- | --- | --- | --- |
| 220 | 2 | 390.2196 | 778.4246 | 778.4225 | 2.76 | 0 | 39 | 0.008 | 1Score **> 31** indicates **identity** | U |  |  | R.DSALVFK |
| 428 | 1 | 412.2192 | 822.4238 | 822.4236 | 0.27 | 0 | 26 | 0.099 | 1Score **> 31** indicates **identity** Score **> 29** indicates **homology** | U |  |  | K.VVTDSFR.S |
| 1371 |  | 478.2511 | 954.4876 | 954.4844 | 3.38 | 0 | 0 | 3.2 | 6Score **> 31** indicates **identity** Score **> 18** indicates **homology** | U |  |  | R.ATAMIAYSK.W |
| 1737 | 1 | 501.2524 | 1000.4903 | 1000.4899 | 0.41 | 0 | 31 | 0.017 | 1Score **> 32** indicates **identity** Score **> 26** indicates **homology** | U |  |  | R.ATAMITYSK.W + Oxidation (M) |
| 7898 | 4 | 807.9286 | 1613.8427 | 1613.8413 | 0.87 | 0 | 92 | 6.2e-009 | 1Score **> 34** indicates **identity** Score **> 22** indicates **homology** | U |  |  | K.QGSITVTSTSLAFFR.D |

---

### 10

|  |  | Accession | Score | Description |
| --- | --- | --- | --- | --- |
|  | 1 | gi|1095456302|gb|APA13257.1| | 212 | hypothetical protein sscle\_10g080270 [Sclerotinia sclerotiorum 1980 UF-70] |

|  |  | Score | Mass | Matches | Sequences | emPAI |  |
| --- | --- | --- | --- | --- | --- | --- | --- |
| 10.1 | gi|1095456302|gb|APA13257.1| | 212 | 67612 | 8 (4) | 5 (2) | 0.10 |  |
|  | hypothetical protein sscle\_10g080270 [Sclerotinia sclerotiorum 1980 UF-70] | | | | | | |

|  |  | Score | Mass | Matches | Sequences | emPAI |  |
| --- | --- | --- | --- | --- | --- | --- | --- |
| 10.1 | gi|1095456302|gb|APA13257.1| | 212 | 67612 | 8 (4) | 5 (2) | 0.10 | hypothetical protein sscle\_10g080270 [Sclerotinia sclerotiorum 1980 UF-70] |
|  |  | | | | | | |

#### 8 peptide matches (6 non-duplicate, 2 duplicate)

Auto-fit to window

| Query | Dupes | Observed | Mr(expt) | Mr(calc) | ppm | M | Score | Expect | Rank | U | Peptide |
| --- | --- | --- | --- | --- | --- | --- | --- | --- | --- | --- | --- |

| Query | Dupes | Observed | Mr(expt) | Mr(calc) | ppm | M | Score | Expect | Rank | U | Peptide |
| --- | --- | --- | --- | --- | --- | --- | --- | --- | --- | --- | --- |
| 23 |  | 354.1953 | 706.3761 | 706.3762 | -0.11 | 0 | 12 | 1.2 | 2Score **> 31** indicates **identity** Score **> 25** indicates **homology** |  | R.ALSNFR.A |
| 293 |  | 396.7221 | 791.4297 | 791.4290 | 0.94 | 0 | 14 | 0.52 | 2Score **> 32** indicates **identity** Score **> 23** indicates **homology** |  | R.AVVNSFR.G |
| 1159 |  | 463.7690 | 925.5235 | 925.5233 | 0.30 | 0 | 26 | 0.12 | 1Score **> 29** indicates **identity** |  | R.ALQPQIEK.Y |
| 5349 | 2 | 668.3492 | 1334.6838 | 1334.6830 | 0.58 | 0 | 96 | 1.2e-008 | 1Score **> 34** indicates **identity** Score **> 30** indicates **homology** | U | K.VSGAGNYVVASPSK.A |
| 5357 |  | 668.8581 | 1335.7016 | 1335.6670 | 25.9 | 0 | 11 | 1.2 | 2Score **> 33** indicates **identity** Score **> 24** indicates **homology** | U | K.VSGAGNYVVASPSK.A + Deamidated (NQ) |
| 6267 |  | 717.3283 | 1432.6421 | 1432.6412 | 0.63 | 0 | 36 | 0.00047 | 1Score **> 32** indicates **identity** Score **> 15** indicates **homology** | U | K.ANPDYFYTWTR.D |

#### 1 subset or intersection (2 subset proteins in total)

|  |  | Score | Mass | Subset of |  |
| --- | --- | --- | --- | --- | --- |
|  | gi|154699211|gb|EDN98949.1| | 195 | 68281 | 10.1 |  |
|  | hypothetical protein SS1G\_13809 [Sclerotinia sclerotiorum 1980 UF-70] | | | | |
|  | 1 sameset of gi|154699211|gb|EDN98949.1| | | | | |
|  | gi|156032806|ref|XP\_001585240.1| | 195 | 68281 |  |  |
|  | hypothetical protein SS1G\_13809 [Sclerotinia sclerotiorum 1980 UF-70] | | | | |

|  |  | Score | Mass | Subset of |  |
| --- | --- | --- | --- | --- | --- |
|  | gi|154699211|gb|EDN98949.1| | 195 | 68281 | 10.1 | hypothetical protein SS1G\_13809 [Sclerotinia sclerotiorum 1980 UF-70] |
|  |  | | | | |
|  | 1 sameset of gi|154699211|gb|EDN98949.1| | | | | |
|  | gi|156032806|ref|XP\_001585240.1| | 195 | 68281 |  | hypothetical protein SS1G\_13809 [Sclerotinia sclerotiorum 1980 UF-70] |
|  |  | | | | |

---

### 11

|  |  | Accession | Score | Description |
| --- | --- | --- | --- | --- |
|  | 1 | gi|154694741|gb|EDN94479.1| | 198 | hypothetical protein SS1G\_10353 [Sclerotinia sclerotiorum 1980 UF-70] |

|  |  | Score | Mass | Matches | Sequences | emPAI |  |
| --- | --- | --- | --- | --- | --- | --- | --- |
| 11.1 | gi|154694741|gb|EDN94479.1| | 198 | 47929 | 12 (7) | 7 (4) | 0.30 |  |
|  | hypothetical protein SS1G\_10353 [Sclerotinia sclerotiorum 1980 UF-70] | | | | | | |
|  | 2 samesets of gi|154694741|gb|EDN94479.1| | | | | | | |
|  | gi|156044498|ref|XP\_001588805.1| | 198 | 47929 | 12 (7) | 7 (4) | 0.30 |  |
|  | hypothetical protein SS1G\_10353 [Sclerotinia sclerotiorum 1980 UF-70] | | | | | | |
|  | gi|1095459233|gb|APA16182.1| | 198 | 47929 | 12 (7) | 7 (4) | 0.30 |  |
|  | hypothetical protein sscle\_16g109520 [Sclerotinia sclerotiorum 1980 UF-70] | | | | | | |

|  |  | Score | Mass | Matches | Sequences | emPAI |  |
| --- | --- | --- | --- | --- | --- | --- | --- |
| 11.1 | gi|154694741|gb|EDN94479.1| | 198 | 47929 | 12 (7) | 7 (4) | 0.30 | hypothetical protein SS1G\_10353 [Sclerotinia sclerotiorum 1980 UF-70] |
|  |  | | | | | | |
|  | 2 samesets of gi|154694741|gb|EDN94479.1| | | | | | | |
|  | gi|156044498|ref|XP\_001588805.1| | 198 | 47929 | 12 (7) | 7 (4) | 0.30 | hypothetical protein SS1G\_10353 [Sclerotinia sclerotiorum 1980 UF-70] |
|  |  | | | | | | |
|  | gi|1095459233|gb|APA16182.1| | 198 | 47929 | 12 (7) | 7 (4) | 0.30 | hypothetical protein sscle\_16g109520 [Sclerotinia sclerotiorum 1980 UF-70] |
|  |  | | | | | | |

#### 12 peptide matches (7 non-duplicate, 5 duplicate)

Auto-fit to window

| Query | Dupes | Observed | Mr(expt) | Mr(calc) | ppm | M | Score | Expect | Rank | U | Peptide |
| --- | --- | --- | --- | --- | --- | --- | --- | --- | --- | --- | --- |

| Query | Dupes | Observed | Mr(expt) | Mr(calc) | ppm | M | Score | Expect | Rank | U | Peptide |
| --- | --- | --- | --- | --- | --- | --- | --- | --- | --- | --- | --- |
| 52 |  | 362.1932 | 722.3719 | 722.3711 | 1.03 | 0 | 18 | 0.17 | 1Score **> 30** indicates **identity** Score **> 23** indicates **homology** | U | R.QYIGSR.G |
| 695 | 1 | 432.7120 | 863.4095 | 863.4283 | -21.8 | 1 | 17 | 0.56 | 1Score **> 33** indicates **identity** Score **> 27** indicates **homology** | U | K.AVTRDMR.Q + Oxidation (M) |
| 1423 | 1 | 480.7598 | 959.5050 | 959.5036 | 1.43 | 0 | 78 | 2.8e-006 | 1Score **> 35** indicates **identity** | U | K.TGAGAGAGLTGK.G |
| 2602 |  | 547.7593 | 1093.5041 | 1093.5040 | 0.11 | 0 | 39 | 0.00085 | 1Score **> 32** indicates **identity** Score **> 21** indicates **homology** | U | R.GVDYQPGGSSK.I |
| 2788 | 1 | 556.2623 | 1110.5101 | 1110.5094 | 0.62 | 0 | 42 | 0.0012 | 1Score **> 31** indicates **identity** Score **> 26** indicates **homology** | U | K.GNAFFIGDNR.F + Deamidated (NQ) |
| 3540 | 1 | 588.8400 | 1175.6655 | 1175.6662 | -0.63 | 1 | 20 | 0.19 | 1Score **> 31** indicates **identity** Score **> 26** indicates **homology** | U | K.FKELGINTVR.V |
| 7348 | 1 | 775.8757 | 1549.7368 | 1549.7372 | -0.29 | 0 | 70 | 1.6e-005 | 1Score **> 34** indicates **identity** | U | R.SIPVGYSAADVDSNR.L |

#### 1 subset or intersection (1 subset protein in total)

|  |  | Score | Mass | Subset of |  |
| --- | --- | --- | --- | --- | --- |
|  | gi|347835485|emb|CCD50057.1| | 39 | 48000 | 11.1 |  |
|  | glycoside hydrolase family 72 protein [Botrytis cinerea T4] | | | | |

|  |  | Score | Mass | Subset of |  |
| --- | --- | --- | --- | --- | --- |
|  | gi|347835485|emb|CCD50057.1| | 39 | 48000 | 11.1 | glycoside hydrolase family 72 protein [Botrytis cinerea T4] |
|  |  | | | | |

---

### 12

|  |  | Accession | Score | Description |
| --- | --- | --- | --- | --- |
|  | 1 | gi|1095455875|gb|APA12830.1| | 181 | hypothetical protein sscle\_10g076000 [Sclerotinia sclerotiorum 1980 UF-70] |

|  |  | Score | Mass | Matches | Sequences | emPAI |  |
| --- | --- | --- | --- | --- | --- | --- | --- |
| 12.1 | gi|1095455875|gb|APA12830.1| | 181 | 112854 | 10 (8) | 6 (4) | 0.15 |  |
|  | hypothetical protein sscle\_10g076000 [Sclerotinia sclerotiorum 1980 UF-70] | | | | | | |

|  |  | Score | Mass | Matches | Sequences | emPAI |  |
| --- | --- | --- | --- | --- | --- | --- | --- |
| 12.1 | gi|1095455875|gb|APA12830.1| | 181 | 112854 | 10 (8) | 6 (4) | 0.15 | hypothetical protein sscle\_10g076000 [Sclerotinia sclerotiorum 1980 UF-70] |
|  |  | | | | | | |

#### 10 peptide matches (7 non-duplicate, 3 duplicate)

Auto-fit to window

| Query | Dupes | Observed | Mr(expt) | Mr(calc) | ppm | M | Score | Expect | Rank | U | Peptide |
| --- | --- | --- | --- | --- | --- | --- | --- | --- | --- | --- | --- |

| Query | Dupes | Observed | Mr(expt) | Mr(calc) | ppm | M | Score | Expect | Rank | U | Peptide |
| --- | --- | --- | --- | --- | --- | --- | --- | --- | --- | --- | --- |
| 172 |  | 382.2145 | 762.4143 | 762.4024 | 15.6 | 0 | 7 | 0.38 | 1Score **> 32** indicates **identity** Score **> 15** indicates **homology** | U | M.AQSLFR.L + Acetyl (Protein N-term) |
| 4659 | 1 | 635.8170 | 1269.6195 | 1269.6201 | -0.48 | 0 | 47 | 0.0021 | 1Score **> 32** indicates **identity** | U | R.VLVSGSDPEDPR.Y |
| 4833 |  | 644.3616 | 1286.7087 | 1286.7082 | 0.41 | 0 | 44 | 0.00015 | 1Score **> 33** indicates **identity** Score **> 19** indicates **homology** | U | R.ILDEVTLQTQK.L |
| 8258 | 1 | 833.4787 | 1664.9428 | 1664.9362 | 3.99 | 0 | 39 | 0.00077 | 1Score **> 29** indicates **identity** Score **> 21** indicates **homology** | U | K.LLPNIPAAVNNFLGGR.T |
| 8670 | 1 | 856.4783 | 1710.9421 | 1710.9417 | 0.24 | 0 | 56 | 6.6e-006 | 1Score **> 31** indicates **identity** Score **> 16** indicates **homology** | U | R.QINIGGWSIPSTIGIR.F |
| 8680 |  | 856.9804 | 1711.9463 | 1711.9257 | 12.0 | 0 | 48 | 0.0012 | 1Score **> 31** indicates **identity** | U | R.QINIGGWSIPSTIGIR.F + Deamidated (NQ) |
| 14483 |  | 1292.1826 | 2582.3507 | 2582.3170 | 13.1 | 0 | 6 | 1 | 1Score **> 33** indicates **identity** Score **> 19** indicates **homology** | U | R.LGEWPNFPDFTLPGVGPTVGAGGAVK.L |

#### 1 subset or intersection (2 subset proteins in total)

|  |  | Score | Mass | Subset of |  |
| --- | --- | --- | --- | --- | --- |
|  | gi|154692679|gb|EDN92417.1| | 163 | 74027 | 12.1 |  |
|  | hypothetical protein SS1G\_08280 [Sclerotinia sclerotiorum 1980 UF-70] | | | | |
|  | 1 sameset of gi|154692679|gb|EDN92417.1| | | | | |
|  | gi|156049147|ref|XP\_001590540.1| | 163 | 74027 |  |  |
|  | hypothetical protein SS1G\_08280 [Sclerotinia sclerotiorum 1980 UF-70] | | | | |

|  |  | Score | Mass | Subset of |  |
| --- | --- | --- | --- | --- | --- |
|  | gi|154692679|gb|EDN92417.1| | 163 | 74027 | 12.1 | hypothetical protein SS1G\_08280 [Sclerotinia sclerotiorum 1980 UF-70] |
|  |  | | | | |
|  | 1 sameset of gi|154692679|gb|EDN92417.1| | | | | |
|  | gi|156049147|ref|XP\_001590540.1| | 163 | 74027 |  | hypothetical protein SS1G\_08280 [Sclerotinia sclerotiorum 1980 UF-70] |
|  |  | | | | |

---

### 13

|  |  | Accession | Score | Description |
| --- | --- | --- | --- | --- |
|  | 1 | gi|154693130|gb|EDN92868.1| | 177 | actin [Sclerotinia sclerotiorum 1980 UF-70] |

|  |  | Score | Mass | Matches | Sequences | emPAI |  |
| --- | --- | --- | --- | --- | --- | --- | --- |
| 13.1 | gi|154693130|gb|EDN92868.1| | 177 | 41841 | 15 (8) | 9 (5) | 0.46 |  |
|  | actin [Sclerotinia sclerotiorum 1980 UF-70] | | | | | | |
|  | 3 samesets of gi|154693130|gb|EDN92868.1| | | | | | | |
|  | gi|156048004|ref|XP\_001589969.1| | 177 | 41841 | 15 (8) | 9 (5) | 0.46 |  |
|  | actin [Sclerotinia sclerotiorum 1980 UF-70] | | | | | | |
|  | gi|347833050|emb|CCD48747.1| | 177 | 48353 | 15 (8) | 9 (5) | 0.39 |  |
|  | actA, actin [Botrytis cinerea T4] | | | | | | |
|  | gi|1095458188|gb|APA15139.1| | 177 | 41841 | 15 (8) | 9 (5) | 0.46 |  |
|  | hypothetical protein sscle\_14g099090 [Sclerotinia sclerotiorum 1980 UF-70] | | | | | | |

|  |  | Score | Mass | Matches | Sequences | emPAI |  |
| --- | --- | --- | --- | --- | --- | --- | --- |
| 13.1 | gi|154693130|gb|EDN92868.1| | 177 | 41841 | 15 (8) | 9 (5) | 0.46 | actin [Sclerotinia sclerotiorum 1980 UF-70] |
|  |  | | | | | | |
|  | 3 samesets of gi|154693130|gb|EDN92868.1| | | | | | | |
|  | gi|156048004|ref|XP\_001589969.1| | 177 | 41841 | 15 (8) | 9 (5) | 0.46 | actin [Sclerotinia sclerotiorum 1980 UF-70] |
|  |  | | | | | | |
|  | gi|347833050|emb|CCD48747.1| | 177 | 48353 | 15 (8) | 9 (5) | 0.39 | actA, actin [Botrytis cinerea T4] |
|  |  | | | | | | |
|  | gi|1095458188|gb|APA15139.1| | 177 | 41841 | 15 (8) | 9 (5) | 0.46 | hypothetical protein sscle\_14g099090 [Sclerotinia sclerotiorum 1980 UF-70] |
|  |  | | | | | | |

#### 15 peptide matches (10 non-duplicate, 5 duplicate)

Auto-fit to window

| Query | Dupes | Observed | Mr(expt) | Mr(calc) | ppm | M | Score | Expect | Rank | U | Peptide |
| --- | --- | --- | --- | --- | --- | --- | --- | --- | --- | --- | --- |

| Query | Dupes | Observed | Mr(expt) | Mr(calc) | ppm | M | Score | Expect | Rank | U | Peptide |
| --- | --- | --- | --- | --- | --- | --- | --- | --- | --- | --- | --- |
| 308 |  | 398.2399 | 794.4653 | 794.4650 | 0.37 | 0 | 18 | 0.092 | 1Score **> 26** indicates **identity** Score **> 20** indicates **homology** | U | K.IIAPPER.K |
| 1144 |  | 462.2871 | 922.5596 | 922.5600 | -0.39 | 1 | 22 | 0.022 | 1Score **> 25** indicates **identity** Score **> 18** indicates **homology** | U | K.IIAPPERK.Y |
| 1549 |  | 488.7279 | 975.4412 | 975.4410 | 0.18 | 0 | 68 | 1.2e-005 | 1Score **> 31** indicates **identity** | U | K.AGFAGDDAPR.A |
| 3809 |  | 400.2398 | 1197.6974 | 1197.6982 | -0.67 | 0 | 5 | 0.93 | 1Score **> 31** indicates **identity** Score **> 17** indicates **homology** |  | R.AVFPSIVGRPR.H |
| 3811 | 1 | 599.8568 | 1197.6991 | 1197.6982 | 0.70 | 0 | 17 | 1 | 1Score **> 31** indicates **identity** Score **> 29** indicates **homology** |  | R.AVFPSIVGRPR.H |
| 5523 |  | 677.8146 | 1353.6146 | 1353.6161 | -1.07 | 1 | 27 | 0.18 | 1Score **> 32** indicates **identity** | U | K.DSYVGDEAQSKR.G |
| 7014 | 1 | 758.8544 | 1515.6943 | 1515.6954 | -0.70 | 0 | 31 | 0.078 | 1Score **> 33** indicates **identity** | U | K.QEYDESGPSIVHR.K |
| 9302 | 1 | 895.9506 | 1789.8866 | 1789.8846 | 1.08 | 0 | 54 | 9.4e-005 | 1Score **> 35** indicates **identity** Score **> 26** indicates **homology** | U | K.SYELPDGQVITIGNER.F |
| 10608 | 2 | 977.5352 | 1953.0558 | 1953.0571 | -0.68 | 0 | 56 | 0.0002 | 1Score **> 32** indicates **identity** | U | R.VAPEEHPVLLTEAPINPK.S |
| 14866 |  | 883.1101 | 2646.3085 | 2646.2370 | 27.0 | 0 | 1 | 1.8 | 9Score **> 35** indicates **identity** Score **> 16** indicates **homology** | U | K.LCYVALDFEQEIQTASQSSSLEK.S + Deamidated (NQ) |

#### 2 subsets and intersections (3 subset proteins in total)

|  |  | Score | Mass | Subset of |  |
| --- | --- | --- | --- | --- | --- |
|  | gi|224459065|gb|ACN43302.1| | 75 | 28105 | 13.1 |  |
|  | actin, partial [Paraphaeosphaeria minitans] | | | | |
|  | gi|569532343|gb|AHE41160.1| | 68 | 3966 | 13.1 |  |
|  | actin, partial [Sclerotinia sclerotiorum] | | | | |
|  | 1 sameset of gi|569532343|gb|AHE41160.1| | | | | |
|  | gi|569532395|gb|AHE41186.1| | 68 | 3966 |  |  |
|  | actin, partial [Sclerotinia sclerotiorum] | | | | |

|  |  | Score | Mass | Subset of |  |
| --- | --- | --- | --- | --- | --- |
|  | gi|224459065|gb|ACN43302.1| | 75 | 28105 | 13.1 | actin, partial [Paraphaeosphaeria minitans] |
|  |  | | | | |
|  | gi|569532343|gb|AHE41160.1| | 68 | 3966 | 13.1 | actin, partial [Sclerotinia sclerotiorum] |
|  |  | | | | |
|  | 1 sameset of gi|569532343|gb|AHE41160.1| | | | | |
|  | gi|569532395|gb|AHE41186.1| | 68 | 3966 |  | actin, partial [Sclerotinia sclerotiorum] |
|  |  | | | | |

---

### 14

|  |  | Accession | Score | Description |
| --- | --- | --- | --- | --- |
|  | 1 | gi|154696190|gb|EDN95928.1| | 170 | predicted protein [Sclerotinia sclerotiorum 1980 UF-70] |

|  |  | Score | Mass | Matches | Sequences | emPAI |  |
| --- | --- | --- | --- | --- | --- | --- | --- |
| 14.1 | gi|154696190|gb|EDN95928.1| | 170 | 37138 | 16 (7) | 7 (5) | 0.53 |  |
|  | predicted protein [Sclerotinia sclerotiorum 1980 UF-70] | | | | | | |
|  | 3 samesets of gi|154696190|gb|EDN95928.1| | | | | | | |
|  | gi|156040235|ref|XP\_001587104.1| | 170 | 37138 | 16 (7) | 7 (5) | 0.53 |  |
|  | predicted protein [Sclerotinia sclerotiorum 1980 UF-70] | | | | | | |
|  | gi|238477231|gb|ACR43468.1| | 170 | 37138 | 16 (7) | 7 (5) | 0.53 |  |
|  | developmental-specific protein Ssp2 [Sclerotinia sclerotiorum] | | | | | | |
|  | gi|1095452383|gb|APA09343.1| | 170 | 37138 | 16 (7) | 7 (5) | 0.53 |  |
|  | hypothetical protein sscle\_05g041130 [Sclerotinia sclerotiorum 1980 UF-70] | | | | | | |

|  |  | Score | Mass | Matches | Sequences | emPAI |  |
| --- | --- | --- | --- | --- | --- | --- | --- |
| 14.1 | gi|154696190|gb|EDN95928.1| | 170 | 37138 | 16 (7) | 7 (5) | 0.53 | predicted protein [Sclerotinia sclerotiorum 1980 UF-70] |
|  |  | | | | | | |
|  | 3 samesets of gi|154696190|gb|EDN95928.1| | | | | | | |
|  | gi|156040235|ref|XP\_001587104.1| | 170 | 37138 | 16 (7) | 7 (5) | 0.53 | predicted protein [Sclerotinia sclerotiorum 1980 UF-70] |
|  |  | | | | | | |
|  | gi|238477231|gb|ACR43468.1| | 170 | 37138 | 16 (7) | 7 (5) | 0.53 | developmental-specific protein Ssp2 [Sclerotinia sclerotiorum] |
|  |  | | | | | | |
|  | gi|1095452383|gb|APA09343.1| | 170 | 37138 | 16 (7) | 7 (5) | 0.53 | hypothetical protein sscle\_05g041130 [Sclerotinia sclerotiorum 1980 UF-70] |
|  |  | | | | | | |

#### 16 peptide matches (9 non-duplicate, 7 duplicate)

Auto-fit to window

| Query | Dupes | Observed | Mr(expt) | Mr(calc) | ppm | M | Score | Expect | Rank | U | Peptide |
| --- | --- | --- | --- | --- | --- | --- | --- | --- | --- | --- | --- |

| Query | Dupes | Observed | Mr(expt) | Mr(calc) | ppm | M | Score | Expect | Rank | U | Peptide |
| --- | --- | --- | --- | --- | --- | --- | --- | --- | --- | --- | --- |
| 653 | 3 | 428.7535 | 855.4925 | 855.4926 | -0.12 | 0 | 36 | 0.013 | 1Score **> 29** indicates **identity** | U | K.RPDLISR.L |
| 1070 | 1 | 457.7119 | 913.4091 | 913.4082 | 0.98 | 0 | 19 | 0.4 | 1Score **> 29** indicates **identity** Score **> 27** indicates **homology** | U | K.DWYFQR.V |
| 1619 |  | 492.8011 | 983.5876 | 983.5876 | 0.044 | 1 | 30 | 0.01 | 1Score **> 28** indicates **identity** Score **> 23** indicates **homology** | U | K.KRPDLISR.L |
| 8192 | 2 | 828.3629 | 1654.7113 | 1654.7145 | -1.93 | 0 | 53 | 0.00031 | 1Score **> 30** indicates **identity** | U | K.QPENDVGTMVYSDGK.W + Oxidation (M) |
| 9242 |  | 595.2764 | 1782.8075 | 1782.8094 | -1.11 | 1 | 23 | 0.57 | 1Score **> 33** indicates **identity** | U | R.KQPENDVGTMVYSDGK.W + Oxidation (M) |
| 9244 |  | 892.4122 | 1782.8098 | 1782.8094 | 0.20 | 1 | 68 | 9.6e-007 | 1Score **> 33** indicates **identity** Score **> 21** indicates **homology** | U | R.KQPENDVGTMVYSDGK.W + Oxidation (M) |
| 12603 | 1 | 1110.5179 | 2219.0213 | 2219.0238 | -1.14 | 0 | 52 | 0.00099 | 1Score **> 34** indicates **identity** | U | K.EEVQPPYQNPTPLACTMTK.N + Oxidation (M) |
| 13333 |  | 1174.5653 | 2347.1161 | 2347.1188 | -1.17 | 1 | 16 | 1 | 1Score **> 35** indicates **identity** Score **> 28** indicates **homology** | U | K.KEEVQPPYQNPTPLACTMTK.N + Oxidation (M) |
| 13334 |  | 783.3799 | 2347.1178 | 2347.1188 | -0.42 | 1 | 9 | 1 | 1Score **> 35** indicates **identity** Score **> 21** indicates **homology** | U | K.KEEVQPPYQNPTPLACTMTK.N + Oxidation (M) |

---

### 15

|  |  | Accession | Score | Description |
| --- | --- | --- | --- | --- |
|  | 1 | gi|154705040|gb|EDO04779.1| | 163 | hypothetical protein SS1G\_07262 [Sclerotinia sclerotiorum 1980 UF-70] |

|  |  | Score | Mass | Matches | Sequences | emPAI |  |
| --- | --- | --- | --- | --- | --- | --- | --- |
| 15.1 | gi|154705040|gb|EDO04779.1| | 163 | 23894 | 6 (4) | 2 (2) | 0.30 |  |
|  | hypothetical protein SS1G\_07262 [Sclerotinia sclerotiorum 1980 UF-70] | | | | | | |
|  | 2 samesets of gi|154705040|gb|EDO04779.1| | | | | | | |
|  | gi|156051710|ref|XP\_001591816.1| | 163 | 23894 | 6 (4) | 2 (2) | 0.30 |  |
|  | hypothetical protein SS1G\_07262 [Sclerotinia sclerotiorum 1980 UF-70] | | | | | | |
|  | gi|1095453329|gb|APA10288.1| | 163 | 26301 | 6 (4) | 2 (2) | 0.27 |  |
|  | hypothetical protein sscle\_06g050580 [Sclerotinia sclerotiorum 1980 UF-70] | | | | | | |

|  |  | Score | Mass | Matches | Sequences | emPAI |  |
| --- | --- | --- | --- | --- | --- | --- | --- |
| 15.1 | gi|154705040|gb|EDO04779.1| | 163 | 23894 | 6 (4) | 2 (2) | 0.30 | hypothetical protein SS1G\_07262 [Sclerotinia sclerotiorum 1980 UF-70] |
|  |  | | | | | | |
|  | 2 samesets of gi|154705040|gb|EDO04779.1| | | | | | | |
|  | gi|156051710|ref|XP\_001591816.1| | 163 | 23894 | 6 (4) | 2 (2) | 0.30 | hypothetical protein SS1G\_07262 [Sclerotinia sclerotiorum 1980 UF-70] |
|  |  | | | | | | |
|  | gi|1095453329|gb|APA10288.1| | 163 | 26301 | 6 (4) | 2 (2) | 0.27 | hypothetical protein sscle\_06g050580 [Sclerotinia sclerotiorum 1980 UF-70] |
|  |  | | | | | | |

#### 6 peptide matches (2 non-duplicate, 4 duplicate)

Auto-fit to window

| Query | Dupes | Observed | Mr(expt) | Mr(calc) | ppm | M | Score | Expect | Rank | U | Peptide |
| --- | --- | --- | --- | --- | --- | --- | --- | --- | --- | --- | --- |

| Query | Dupes | Observed | Mr(expt) | Mr(calc) | ppm | M | Score | Expect | Rank | U | Peptide |
| --- | --- | --- | --- | --- | --- | --- | --- | --- | --- | --- | --- |
| 2673 | 4 | 550.3092 | 1098.6038 | 1098.6033 | 0.48 | 0 | 78 | 1.2e-006 | 1Score **> 31** indicates **identity** | U | R.VDPSVLISNR.Q |
| 4271 |  | 618.8359 | 1235.6572 | 1235.6550 | 1.76 | 0 | 39 | 0.0021 | 1Score **> 32** indicates **identity** Score **> 25** indicates **homology** | U | R.FGGLTPGEFLAK.W |

#### 1 subset or intersection (1 subset protein in total)

|  |  | Score | Mass | Subset of |  |
| --- | --- | --- | --- | --- | --- |
|  | gi|347839683|emb|CCD54255.1| | 149 | 27846 | 15.1 |  |
|  | hypothetical protein BofuT4\_P129550.1 [Botrytis cinerea T4] | | | | |

|  |  | Score | Mass | Subset of |  |
| --- | --- | --- | --- | --- | --- |
|  | gi|347839683|emb|CCD54255.1| | 149 | 27846 | 15.1 | hypothetical protein BofuT4\_P129550.1 [Botrytis cinerea T4] |
|  |  | | | | |

---

### 16

|  |  | Accession | Score | Description |
| --- | --- | --- | --- | --- |
|  | 1 | gi|154694427|gb|EDN94165.1| | 161 | hypothetical protein SS1G\_10038 [Sclerotinia sclerotiorum 1980 UF-70] |

|  |  | Score | Mass | Matches | Sequences | emPAI |  |
| --- | --- | --- | --- | --- | --- | --- | --- |
| 16.1 | gi|154694427|gb|EDN94165.1| | 161 | 64156 | 13 (6) | 6 (4) | 0.22 |  |
|  | hypothetical protein SS1G\_10038 [Sclerotinia sclerotiorum 1980 UF-70] | | | | | | |
|  | 2 samesets of gi|154694427|gb|EDN94165.1| | | | | | | |
|  | gi|156045687|ref|XP\_001589399.1| | 161 | 64156 | 13 (6) | 6 (4) | 0.22 |  |
|  | hypothetical protein SS1G\_10038 [Sclerotinia sclerotiorum 1980 UF-70] | | | | | | |
|  | gi|1095448599|gb|APA05563.1| | 161 | 64156 | 13 (6) | 6 (4) | 0.22 |  |
|  | hypothetical protein sscle\_01g003330 [Sclerotinia sclerotiorum 1980 UF-70] | | | | | | |

|  |  | Score | Mass | Matches | Sequences | emPAI |  |
| --- | --- | --- | --- | --- | --- | --- | --- |
| 16.1 | gi|154694427|gb|EDN94165.1| | 161 | 64156 | 13 (6) | 6 (4) | 0.22 | hypothetical protein SS1G\_10038 [Sclerotinia sclerotiorum 1980 UF-70] |
|  |  | | | | | | |
|  | 2 samesets of gi|154694427|gb|EDN94165.1| | | | | | | |
|  | gi|156045687|ref|XP\_001589399.1| | 161 | 64156 | 13 (6) | 6 (4) | 0.22 | hypothetical protein SS1G\_10038 [Sclerotinia sclerotiorum 1980 UF-70] |
|  |  | | | | | | |
|  | gi|1095448599|gb|APA05563.1| | 161 | 64156 | 13 (6) | 6 (4) | 0.22 | hypothetical protein sscle\_01g003330 [Sclerotinia sclerotiorum 1980 UF-70] |
|  |  | | | | | | |

#### 13 peptide matches (8 non-duplicate, 5 duplicate)

Auto-fit to window

| Query | Dupes | Observed | Mr(expt) | Mr(calc) | ppm | M | Score | Expect | Rank | U | Peptide |
| --- | --- | --- | --- | --- | --- | --- | --- | --- | --- | --- | --- |

| Query | Dupes | Observed | Mr(expt) | Mr(calc) | ppm | M | Score | Expect | Rank | U | Peptide |
| --- | --- | --- | --- | --- | --- | --- | --- | --- | --- | --- | --- |
| 3269 |  | 577.8009 | 1153.5872 | 1153.5880 | -0.64 | 0 | 14 | 0.42 | 1Score **> 32** indicates **identity** Score **> 23** indicates **homology** | U | R.LPEFNEHLR.T |
| 3270 |  | 385.5366 | 1153.5880 | 1153.5880 | 0.043 | 0 | 18 | 0.16 | 1Score **> 32** indicates **identity** Score **> 23** indicates **homology** | U | R.LPEFNEHLR.T |
| 3677 | 1 | 594.3373 | 1186.6601 | 1186.6598 | 0.31 | 0 | 53 | 0.00047 | 1Score **> 33** indicates **identity** | U | K.LAPVTIYDAPK.F |
| 4173 |  | 615.8261 | 1229.6375 | 1229.6404 | -2.33 | 0 | 53 | 0.00012 | 1Score **> 34** indicates **identity** Score **> 26** indicates **homology** | U | R.GAAAAEVLWSGAK.D |
| 4457 | 1 | 626.8410 | 1251.6674 | 1251.6652 | 1.80 | 0 | 58 | 2.3e-005 | 1Score **> 32** indicates **identity** Score **> 24** indicates **homology** | U | K.IFYQGLTPWK.F |
| 5212 | 2 | 440.5629 | 1318.6670 | 1318.6670 |  | 1 | 21 | 0.014 | 1Score **> 34** indicates **identity** Score **> 15** indicates **homology** | U | R.NWYPVEDIKR.T |
| 5214 | 1 | 660.3411 | 1318.6676 | 1318.6670 | 0.46 | 1 | 9 | 1 | 1Score **> 34** indicates **identity** Score **> 21** indicates **homology** | U | R.NWYPVEDIKR.T |
| 8561 |  | 850.9145 | 1699.8144 | 1699.8238 | -5.50 | 1 | 3 | 0.64 | 1Score **> 34** indicates **identity** Score **> 13** indicates **homology** | U | K.DPVTGQNRSQIDAGSR.L |

#### 1 subset or intersection (1 subset protein in total)

|  |  | Score | Mass | Subset of |  |
| --- | --- | --- | --- | --- | --- |
|  | gi|347838271|emb|CCD52843.1| | 95 | 69881 | 16.1 |  |
|  | glycoside hydrolase family 20 protein [Botrytis cinerea T4] | | | | |

|  |  | Score | Mass | Subset of |  |
| --- | --- | --- | --- | --- | --- |
|  | gi|347838271|emb|CCD52843.1| | 95 | 69881 | 16.1 | glycoside hydrolase family 20 protein [Botrytis cinerea T4] |
|  |  | | | | |

---

### 17

|  |  | Accession | Score | Description |
| --- | --- | --- | --- | --- |
|  | 1 | gi|1095450409|gb|APA07372.1| | 160 | hypothetical protein sscle\_02g021420 [Sclerotinia sclerotiorum 1980 UF-70] |

|  |  | Score | Mass | Matches | Sequences | emPAI |  |
| --- | --- | --- | --- | --- | --- | --- | --- |
| 17.1 | gi|1095450409|gb|APA07372.1| | 160 | 64659 | 8 (4) | 6 (3) | 0.16 |  |
|  | hypothetical protein sscle\_02g021420 [Sclerotinia sclerotiorum 1980 UF-70] | | | | | | |

|  |  | Score | Mass | Matches | Sequences | emPAI |  |
| --- | --- | --- | --- | --- | --- | --- | --- |
| 17.1 | gi|1095450409|gb|APA07372.1| | 160 | 64659 | 8 (4) | 6 (3) | 0.16 | hypothetical protein sscle\_02g021420 [Sclerotinia sclerotiorum 1980 UF-70] |
|  |  | | | | | | |

#### 8 peptide matches (6 non-duplicate, 2 duplicate)

Auto-fit to window

| Query | Dupes | Observed | Mr(expt) | Mr(calc) | ppm | M | Score | Expect | Rank | U | Peptide |
| --- | --- | --- | --- | --- | --- | --- | --- | --- | --- | --- | --- |

| Query | Dupes | Observed | Mr(expt) | Mr(calc) | ppm | M | Score | Expect | Rank | U | Peptide |
| --- | --- | --- | --- | --- | --- | --- | --- | --- | --- | --- | --- |
| 8 |  | 351.2133 | 700.4120 | 700.4119 | 0.17 | 0 | 19 | 0.34 | 1Score **> 33** indicates **identity** Score **> 27** indicates **homology** | U | R.LAEAGLK.T |
| 3164 |  | 571.7828 | 1141.5510 | 1141.5516 | -0.51 | 0 | 41 | 0.0017 | 1Score **> 31** indicates **identity** Score **> 25** indicates **homology** | U | R.YLQSGYNAAR.K |
| 3733 |  | 596.8407 | 1191.6668 | 1191.6652 | 1.40 | 1 | 23 | 0.33 | 1Score **> 31** indicates **identity** | U | R.KWLVEGLGYK.D |
| 5190 | 1 | 659.2918 | 1316.5691 | 1316.5680 | 0.79 | 0 | 24 | 0.18 | 1Score **> 29** indicates **identity** | U | R.GQVNHWSSSCR.L |
| 10072 |  | 941.4629 | 1880.9112 | 1880.9116 | -0.19 | 0 | 59 | 4.6e-006 | 1Score **> 35** indicates **identity** Score **> 18** indicates **homology** | U | R.LYDTQPSTSLTSQDGIR.Y |
| 11562 | 1 | 1039.0319 | 2076.0492 | 2076.0488 | 0.20 | 0 | 91 | 3.6e-009 | 1Score **> 35** indicates **identity** Score **> 19** indicates **homology** | U | K.TLLLESGGPSYGVTGGDLNAR.R |

#### 1 subset or intersection (2 subset proteins in total)

|  |  | Score | Mass | Subset of |  |
| --- | --- | --- | --- | --- | --- |
|  | gi|154698456|gb|EDN98194.1| | 78 | 60811 | 17.1 |  |
|  | hypothetical protein SS1G\_13051 [Sclerotinia sclerotiorum 1980 UF-70] | | | | |
|  | 1 sameset of gi|154698456|gb|EDN98194.1| | | | | |
|  | gi|156035695|ref|XP\_001585959.1| | 78 | 60811 |  |  |
|  | hypothetical protein SS1G\_13051 [Sclerotinia sclerotiorum 1980 UF-70] | | | | |

|  |  | Score | Mass | Subset of |  |
| --- | --- | --- | --- | --- | --- |
|  | gi|154698456|gb|EDN98194.1| | 78 | 60811 | 17.1 | hypothetical protein SS1G\_13051 [Sclerotinia sclerotiorum 1980 UF-70] |
|  |  | | | | |
|  | 1 sameset of gi|154698456|gb|EDN98194.1| | | | | |
|  | gi|156035695|ref|XP\_001585959.1| | 78 | 60811 |  | hypothetical protein SS1G\_13051 [Sclerotinia sclerotiorum 1980 UF-70] |
|  |  | | | | |

---

### 18

|  |  | Accession | Score | Description |
| --- | --- | --- | --- | --- |
|  | 1 | gi|154696912|gb|EDN96650.1| | 125 | hypothetical protein SS1G\_01576 [Sclerotinia sclerotiorum 1980 UF-70] |

|  |  | Score | Mass | Matches | Sequences | emPAI |  |
| --- | --- | --- | --- | --- | --- | --- | --- |
| 18.1 | gi|154696912|gb|EDN96650.1| | 125 | 66177 | 8 (4) | 3 (2) | 0.16 |  |
|  | hypothetical protein SS1G\_01576 [Sclerotinia sclerotiorum 1980 UF-70] | | | | | | |
|  | 2 samesets of gi|154696912|gb|EDN96650.1| | | | | | | |
|  | gi|156062920|ref|XP\_001597382.1| | 125 | 66177 | 8 (4) | 3 (2) | 0.16 |  |
|  | hypothetical protein SS1G\_01576 [Sclerotinia sclerotiorum 1980 UF-70] | | | | | | |
|  | gi|1095449044|gb|APA06008.1| | 125 | 63412 | 8 (4) | 3 (2) | 0.16 |  |
|  | hypothetical protein sscle\_01g007780 [Sclerotinia sclerotiorum 1980 UF-70] | | | | | | |

|  |  | Score | Mass | Matches | Sequences | emPAI |  |
| --- | --- | --- | --- | --- | --- | --- | --- |
| 18.1 | gi|154696912|gb|EDN96650.1| | 125 | 66177 | 8 (4) | 3 (2) | 0.16 | hypothetical protein SS1G\_01576 [Sclerotinia sclerotiorum 1980 UF-70] |
|  |  | | | | | | |
|  | 2 samesets of gi|154696912|gb|EDN96650.1| | | | | | | |
|  | gi|156062920|ref|XP\_001597382.1| | 125 | 66177 | 8 (4) | 3 (2) | 0.16 | hypothetical protein SS1G\_01576 [Sclerotinia sclerotiorum 1980 UF-70] |
|  |  | | | | | | |
|  | gi|1095449044|gb|APA06008.1| | 125 | 63412 | 8 (4) | 3 (2) | 0.16 | hypothetical protein sscle\_01g007780 [Sclerotinia sclerotiorum 1980 UF-70] |
|  |  | | | | | | |

#### 8 peptide matches (4 non-duplicate, 4 duplicate)

Auto-fit to window

| Query | Dupes | Observed | Mr(expt) | Mr(calc) | ppm | M | Score | Expect | Rank | U | Peptide |
| --- | --- | --- | --- | --- | --- | --- | --- | --- | --- | --- | --- |

| Query | Dupes | Observed | Mr(expt) | Mr(calc) | ppm | M | Score | Expect | Rank | U | Peptide |
| --- | --- | --- | --- | --- | --- | --- | --- | --- | --- | --- | --- |
| 2557 | 1 | 545.7839 | 1089.5533 | 1089.5567 | -3.10 | 0 | 46 | 0.0042 | 1Score **> 34** indicates **identity** | U | R.QGAATWTISR.G |
| 2566 | 2 | 546.2778 | 1090.5411 | 1090.5407 | 0.36 | 0 | 48 | 0.0022 | 1Score **> 34** indicates **identity** Score **> 34** indicates **homology** | U | R.QGAATWTISR.G + Deamidated (NQ) |
| 3138 |  | 570.7988 | 1139.5830 | 1139.6159 | -28.9 | 1 | 6 | 1.1 | 2Score **> 32** indicates **identity** Score **> 19** indicates **homology** | U | K.RQAATPSRPR.D + Deamidated (NQ) |
| 8944 | 1 | 873.4451 | 1744.8757 | 1744.8744 | 0.73 | 0 | 60 | 4.5e-006 | 1Score **> 35** indicates **identity** Score **> 19** indicates **homology** | U | R.ADTFAITGVQDGGIQPR.L |

---

### 19

|  |  | Accession | Score | Description |
| --- | --- | --- | --- | --- |
|  | 1 | gi|154702253|gb|EDO01992.1| | 123 | hypothetical protein SS1G\_04468 [Sclerotinia sclerotiorum 1980 UF-70] |
|  | 2 | gi\|347829827\|emb\|CCD45524.1\| | 110 | glycoside hydrolase family 47 protein [Botrytis cinerea T4] |
|  |  |  |  |  |
| Cut threshold Threshold (**0**): | | | | |

|  |  | Score | Mass | Matches | Sequences | emPAI |  |
| --- | --- | --- | --- | --- | --- | --- | --- |
| 19.1 | gi|154702253|gb|EDO01992.1| | 123 | 58064 | 6 (5) | 4 (4) | 0.25 |  |
|  | hypothetical protein SS1G\_04468 [Sclerotinia sclerotiorum 1980 UF-70] | | | | | | |
|  | 2 samesets of gi|154702253|gb|EDO01992.1| | | | | | | |
|  | gi|156057473|ref|XP\_001594660.1| | 123 | 58064 | 6 (5) | 4 (4) | 0.25 |  |
|  | hypothetical protein SS1G\_04468 [Sclerotinia sclerotiorum 1980 UF-70] | | | | | | |
|  | gi|1095449920|gb|APA06883.1| | 123 | 58064 | 6 (5) | 4 (4) | 0.25 |  |
|  | hypothetical protein sscle\_02g016530 [Sclerotinia sclerotiorum 1980 UF-70] | | | | | | |
| 19.2 | gi|347829827|emb|CCD45524.1| | 110 | 58211 | 5 (4) | 3 (3) | 0.18 |  |
|  | glycoside hydrolase family 47 protein [Botrytis cinerea T4] | | | | | | |
|  | | | | | | |

|  |  | Score | Mass | Matches | Sequences | emPAI |  |
| --- | --- | --- | --- | --- | --- | --- | --- |
| 19.1 | gi|154702253|gb|EDO01992.1| | 123 | 58064 | 6 (5) | 4 (4) | 0.25 | hypothetical protein SS1G\_04468 [Sclerotinia sclerotiorum 1980 UF-70] |
|  |  | | | | | | |
|  | 2 samesets of gi|154702253|gb|EDO01992.1| | | | | | | |
|  | gi|156057473|ref|XP\_001594660.1| | 123 | 58064 | 6 (5) | 4 (4) | 0.25 | hypothetical protein SS1G\_04468 [Sclerotinia sclerotiorum 1980 UF-70] |
|  |  | | | | | | |
|  | gi|1095449920|gb|APA06883.1| | 123 | 58064 | 6 (5) | 4 (4) | 0.25 | hypothetical protein sscle\_02g016530 [Sclerotinia sclerotiorum 1980 UF-70] |
|  |  | | | | | | |
| 19.2 | gi|347829827|emb|CCD45524.1| | 110 | 58211 | 5 (4) | 3 (3) | 0.18 | glycoside hydrolase family 47 protein [Botrytis cinerea T4] |
|  |  | | | | | | |
|  | | | | | | |

#### 8 peptide matches (6 non-duplicate, 2 duplicate)

Auto-fit to window

| Query | Dupes | Observed | Mr(expt) | Mr(calc) | ppm | M | Score | Expect | Rank | U | 1 | 2 | Peptide |
| --- | --- | --- | --- | --- | --- | --- | --- | --- | --- | --- | --- | --- | --- |

| Query | Dupes | Observed | Mr(expt) | Mr(calc) | ppm | M | Score | Expect | Rank | U | 1 | 2 | Peptide |
| --- | --- | --- | --- | --- | --- | --- | --- | --- | --- | --- | --- | --- | --- |
| 1147 |  | 463.2353 | 924.4561 | 924.4552 | 0.91 | 0 | 16 | 0.035 | 1Score **> 31** indicates **identity** Score **> 14** indicates **homology** | U |  |  | K.TYGELSQK.G |
| 2165 |  | 525.7319 | 1049.4492 | 1049.4488 | 0.38 | 0 | 22 | 0.029 | 1Score **> 29** indicates **identity** Score **> 19** indicates **homology** | U |  |  | K.MYVYDSTR.F + Oxidation (M) |
| 2278 | 1 | 531.2850 | 1060.5555 | 1060.5553 | 0.21 | 0 | 38 | 0.02 | 1Score **> 34** indicates **identity** | U |  |  | R.WIAAADSSIK.H |
| 5404 |  | 671.3511 | 1340.6877 | 1340.6877 | 0.010 | 0 | 41 | 0.00068 | 1Score **> 33** indicates **identity** Score **> 22** indicates **homology** | U |  |  | K.WVFNTEAHPLK.V |
| 5405 |  | 447.9035 | 1340.6886 | 1340.6877 | 0.65 | 0 | 7 | 0.23 | 1Score **> 33** indicates **identity** Score **> 13** indicates **homology** | U |  |  | K.WVFNTEAHPLK.V |
| 5810 | 1 | 691.3909 | 1380.7673 | 1380.7653 | 1.46 | 0 | 65 | 1.8e-006 | 1Score **> 30** indicates **identity** Score **> 20** indicates **homology** | U |  |  | R.YVGGLLAGYDLLK.G |

---

### 20

|  |  | Accession | Score | Description |
| --- | --- | --- | --- | --- |
|  | 1 | gi|154701174|gb|EDO00913.1| | 117 | hypothetical protein SS1G\_03387 [Sclerotinia sclerotiorum 1980 UF-70] |

|  |  | Score | Mass | Matches | Sequences | emPAI |  |
| --- | --- | --- | --- | --- | --- | --- | --- |
| 20.1 | gi|154701174|gb|EDO00913.1| | 117 | 63029 | 3 (3) | 2 (2) | 0.11 |  |
|  | hypothetical protein SS1G\_03387 [Sclerotinia sclerotiorum 1980 UF-70] | | | | | | |
|  | 2 samesets of gi|154701174|gb|EDO00913.1| | | | | | | |
|  | gi|156058750|ref|XP\_001595298.1| | 117 | 63029 | 3 (3) | 2 (2) | 0.11 |  |
|  | hypothetical protein SS1G\_03387 [Sclerotinia sclerotiorum 1980 UF-70] | | | | | | |
|  | gi|1095453967|gb|APA10925.1| | 117 | 69769 | 3 (3) | 2 (2) | 0.10 |  |
|  | hypothetical protein sscle\_07g056950 [Sclerotinia sclerotiorum 1980 UF-70] | | | | | | |

|  |  | Score | Mass | Matches | Sequences | emPAI |  |
| --- | --- | --- | --- | --- | --- | --- | --- |
| 20.1 | gi|154701174|gb|EDO00913.1| | 117 | 63029 | 3 (3) | 2 (2) | 0.11 | hypothetical protein SS1G\_03387 [Sclerotinia sclerotiorum 1980 UF-70] |
|  |  | | | | | | |
|  | 2 samesets of gi|154701174|gb|EDO00913.1| | | | | | | |
|  | gi|156058750|ref|XP\_001595298.1| | 117 | 63029 | 3 (3) | 2 (2) | 0.11 | hypothetical protein SS1G\_03387 [Sclerotinia sclerotiorum 1980 UF-70] |
|  |  | | | | | | |
|  | gi|1095453967|gb|APA10925.1| | 117 | 69769 | 3 (3) | 2 (2) | 0.10 | hypothetical protein sscle\_07g056950 [Sclerotinia sclerotiorum 1980 UF-70] |
|  |  | | | | | | |

#### 3 peptide matches (2 non-duplicate, 1 duplicate)

Auto-fit to window

| Query | Dupes | Observed | Mr(expt) | Mr(calc) | ppm | M | Score | Expect | Rank | U | Peptide |
| --- | --- | --- | --- | --- | --- | --- | --- | --- | --- | --- | --- |

| Query | Dupes | Observed | Mr(expt) | Mr(calc) | ppm | M | Score | Expect | Rank | U | Peptide |
| --- | --- | --- | --- | --- | --- | --- | --- | --- | --- | --- | --- |
| 794 |  | 440.2193 | 878.4240 | 878.4246 | -0.72 | 0 | 30 | 0.017 | 1Score **> 33** indicates **identity** Score **> 25** indicates **homology** | U | K.AEGFNSVR.I |
| 4615 | 1 | 633.3358 | 1264.6571 | 1264.6564 | 0.53 | 0 | 82 | 6.1e-007 | 1Score **> 33** indicates **identity** Score **> 32** indicates **homology** | U | K.FSALWSQIGTR.M |

#### 1 subset or intersection (1 subset protein in total)

|  |  | Score | Mass | Subset of |  |
| --- | --- | --- | --- | --- | --- |
|  | gi|347831056|emb|CCD46753.1| | 30 | 71027 | 20.1 |  |
|  | glycoside hydrolase family 5 protein [Botrytis cinerea T4] | | | | |

|  |  | Score | Mass | Subset of |  |
| --- | --- | --- | --- | --- | --- |
|  | gi|347831056|emb|CCD46753.1| | 30 | 71027 | 20.1 | glycoside hydrolase family 5 protein [Botrytis cinerea T4] |
|  |  | | | | |

---

### 21

|  |  | Accession | Score | Description |
| --- | --- | --- | --- | --- |
|  | 1 | gi|154696764|gb|EDN96502.1| | 105 | hypothetical protein SS1G\_01428 [Sclerotinia sclerotiorum 1980 UF-70] |

|  |  | Score | Mass | Matches | Sequences | emPAI |  |
| --- | --- | --- | --- | --- | --- | --- | --- |
| 21.1 | gi|154696764|gb|EDN96502.1| | 105 | 45622 | 6 (6) | 2 (2) | 0.15 |  |
|  | hypothetical protein SS1G\_01428 [Sclerotinia sclerotiorum 1980 UF-70] | | | | | | |
|  | 2 samesets of gi|154696764|gb|EDN96502.1| | | | | | | |
|  | gi|156062624|ref|XP\_001597234.1| | 105 | 45622 | 6 (6) | 2 (2) | 0.15 |  |
|  | hypothetical protein SS1G\_01428 [Sclerotinia sclerotiorum 1980 UF-70] | | | | | | |
|  | gi|1095449159|gb|APA06123.1| | 105 | 45249 | 6 (6) | 2 (2) | 0.15 |  |
|  | hypothetical protein sscle\_01g008930 [Sclerotinia sclerotiorum 1980 UF-70] | | | | | | |

|  |  | Score | Mass | Matches | Sequences | emPAI |  |
| --- | --- | --- | --- | --- | --- | --- | --- |
| 21.1 | gi|154696764|gb|EDN96502.1| | 105 | 45622 | 6 (6) | 2 (2) | 0.15 | hypothetical protein SS1G\_01428 [Sclerotinia sclerotiorum 1980 UF-70] |
|  |  | | | | | | |
|  | 2 samesets of gi|154696764|gb|EDN96502.1| | | | | | | |
|  | gi|156062624|ref|XP\_001597234.1| | 105 | 45622 | 6 (6) | 2 (2) | 0.15 | hypothetical protein SS1G\_01428 [Sclerotinia sclerotiorum 1980 UF-70] |
|  |  | | | | | | |
|  | gi|1095449159|gb|APA06123.1| | 105 | 45249 | 6 (6) | 2 (2) | 0.15 | hypothetical protein sscle\_01g008930 [Sclerotinia sclerotiorum 1980 UF-70] |
|  |  | | | | | | |

#### 6 peptide matches (2 non-duplicate, 4 duplicate)

Auto-fit to window

| Query | Dupes | Observed | Mr(expt) | Mr(calc) | ppm | M | Score | Expect | Rank | U | Peptide |
| --- | --- | --- | --- | --- | --- | --- | --- | --- | --- | --- | --- |

| Query | Dupes | Observed | Mr(expt) | Mr(calc) | ppm | M | Score | Expect | Rank | U | Peptide |
| --- | --- | --- | --- | --- | --- | --- | --- | --- | --- | --- | --- |
| 4490 | 4 | 628.3484 | 1254.6822 | 1254.6820 | 0.21 | 0 | 48 | 0.00029 | 1Score **> 32** indicates **identity** Score **> 25** indicates **homology** | U | K.ASAVVIDPPTSAK.K |
| 7254 |  | 771.4236 | 1540.8326 | 1540.8290 | 2.36 | 0 | 38 | 0.0024 | 1Score **> 32** indicates **identity** Score **> 25** indicates **homology** | U | K.IFLTPQGTYFQVK.C |

#### 1 subset or intersection (1 subset protein in total)

|  |  | Score | Mass | Subset of |  |
| --- | --- | --- | --- | --- | --- |
|  | gi|347838629|emb|CCD53201.1| | 91 | 45879 | 21.1 |  |
|  | hypothetical protein BofuT4\_P122200.1 [Botrytis cinerea T4] | | | | |

|  |  | Score | Mass | Subset of |  |
| --- | --- | --- | --- | --- | --- |
|  | gi|347838629|emb|CCD53201.1| | 91 | 45879 | 21.1 | hypothetical protein BofuT4\_P122200.1 [Botrytis cinerea T4] |
|  |  | | | | |

---

### 22

|  |  | Accession | Score | Description |
| --- | --- | --- | --- | --- |
|  | 1 | gi|347832626|emb|CCD48323.1| | 102 | glycoside hydrolase family 71 protein [Botrytis cinerea T4] |

|  |  | Score | Mass | Matches | Sequences | emPAI |  |
| --- | --- | --- | --- | --- | --- | --- | --- |
| 22.1 | gi|347832626|emb|CCD48323.1| | 102 | 81772 | 2 (1) | 2 (1) | 0.04 |  |
|  | glycoside hydrolase family 71 protein [Botrytis cinerea T4] | | | | | | |

|  |  | Score | Mass | Matches | Sequences | emPAI |  |
| --- | --- | --- | --- | --- | --- | --- | --- |
| 22.1 | gi|347832626|emb|CCD48323.1| | 102 | 81772 | 2 (1) | 2 (1) | 0.04 | glycoside hydrolase family 71 protein [Botrytis cinerea T4] |
|  |  | | | | | | |

#### 2 peptide matches (2 non-duplicate, 0 duplicate)

Auto-fit to window

| Query | Dupes | Observed | Mr(expt) | Mr(calc) | ppm | M | Score | Expect | Rank | U | Peptide |
| --- | --- | --- | --- | --- | --- | --- | --- | --- | --- | --- | --- |

| Query | Dupes | Observed | Mr(expt) | Mr(calc) | ppm | M | Score | Expect | Rank | U | Peptide |
| --- | --- | --- | --- | --- | --- | --- | --- | --- | --- | --- | --- |
| 1464 |  | 483.7612 | 965.5078 | 965.5083 | -0.53 | 0 | 16 | 0.8 | 1Score **> 31** indicates **identity** Score **> 28** indicates **homology** |  | K.QQFFLQR.G |
| 9019 |  | 876.9512 | 1751.8878 | 1751.8842 | 2.03 | 0 | 102 | 2.9e-009 | 1Score **> 34** indicates **identity** Score **> 29** indicates **homology** | U | K.AFASSFAGDQLDIAALR.A |

---

### 23

|  |  | Accession | Score | Description |
| --- | --- | --- | --- | --- |
|  | 1 | gi|154704206|gb|EDO03945.1| | 95 | hypothetical protein SS1G\_06426 [Sclerotinia sclerotiorum 1980 UF-70] |

|  |  | Score | Mass | Matches | Sequences | emPAI |  |
| --- | --- | --- | --- | --- | --- | --- | --- |
| 23.1 | gi|154704206|gb|EDO03945.1| | 95 | 95078 | 6 (4) | 4 (2) | 0.07 |  |
|  | hypothetical protein SS1G\_06426 [Sclerotinia sclerotiorum 1980 UF-70] | | | | | | |
|  | 2 samesets of gi|154704206|gb|EDO03945.1| | | | | | | |
|  | gi|156052521|ref|XP\_001592187.1| | 95 | 95078 | 6 (4) | 4 (2) | 0.07 |  |
|  | hypothetical protein SS1G\_06426 [Sclerotinia sclerotiorum 1980 UF-70] | | | | | | |
|  | gi|1095457549|gb|APA14501.1| | 95 | 95078 | 6 (4) | 4 (2) | 0.07 |  |
|  | hypothetical protein sscle\_13g092710 [Sclerotinia sclerotiorum 1980 UF-70] | | | | | | |

|  |  | Score | Mass | Matches | Sequences | emPAI |  |
| --- | --- | --- | --- | --- | --- | --- | --- |
| 23.1 | gi|154704206|gb|EDO03945.1| | 95 | 95078 | 6 (4) | 4 (2) | 0.07 | hypothetical protein SS1G\_06426 [Sclerotinia sclerotiorum 1980 UF-70] |
|  |  | | | | | | |
|  | 2 samesets of gi|154704206|gb|EDO03945.1| | | | | | | |
|  | gi|156052521|ref|XP\_001592187.1| | 95 | 95078 | 6 (4) | 4 (2) | 0.07 | hypothetical protein SS1G\_06426 [Sclerotinia sclerotiorum 1980 UF-70] |
|  |  | | | | | | |
|  | gi|1095457549|gb|APA14501.1| | 95 | 95078 | 6 (4) | 4 (2) | 0.07 | hypothetical protein sscle\_13g092710 [Sclerotinia sclerotiorum 1980 UF-70] |
|  |  | | | | | | |

#### 6 peptide matches (4 non-duplicate, 2 duplicate)

Auto-fit to window

| Query | Dupes | Observed | Mr(expt) | Mr(calc) | ppm | M | Score | Expect | Rank | U | Peptide |
| --- | --- | --- | --- | --- | --- | --- | --- | --- | --- | --- | --- |

| Query | Dupes | Observed | Mr(expt) | Mr(calc) | ppm | M | Score | Expect | Rank | U | Peptide |
| --- | --- | --- | --- | --- | --- | --- | --- | --- | --- | --- | --- |
| 2424 | 2 | 538.7919 | 1075.5692 | 1075.5695 | -0.34 | 0 | 51 | 5.5e-005 | 1Score **> 34** indicates **identity** Score **> 21** indicates **homology** | U | K.TAANAVIAAMK.T + Oxidation (M) |
| 4355 |  | 415.2344 | 1242.6815 | 1242.6455 | 28.9 | 0 | 3 | 2.2 | 5Score **> 33** indicates **identity** Score **> 19** indicates **homology** | U | K.QAISPNAAEITK.L + Deamidated (NQ) |
| 8059 |  | 819.3740 | 1636.7335 | 1636.7329 | 0.36 | 0 | 38 | 0.00043 | 1Score **> 32** indicates **identity** Score **> 17** indicates **homology** | U | R.AVGDSSWTSVDSAAER.A |
| 10072 |  | 941.4629 | 1880.9112 | 1880.9380 | -14.3 | 2 | 1 | 2.8 | 4Score **> 35** indicates **identity** Score **> 18** indicates **homology** | U | K.NKYTLTPSSSPWGRVR.V + Deamidated (NQ); Dioxidation (W) |

---

### 24

|  |  | Accession | Score | Description |
| --- | --- | --- | --- | --- |
|  | 1 | gi|1095455126|gb|APA12083.1| | 91 | hypothetical protein sscle\_08g068530 [Sclerotinia sclerotiorum 1980 UF-70] |

|  |  | Score | Mass | Matches | Sequences | emPAI |  |
| --- | --- | --- | --- | --- | --- | --- | --- |
| 24.1 | gi|1095455126|gb|APA12083.1| | 91 | 62168 | 9 (3) | 6 (2) | 0.11 |  |
|  | hypothetical protein sscle\_08g068530 [Sclerotinia sclerotiorum 1980 UF-70] | | | | | | |

|  |  | Score | Mass | Matches | Sequences | emPAI |  |
| --- | --- | --- | --- | --- | --- | --- | --- |
| 24.1 | gi|1095455126|gb|APA12083.1| | 91 | 62168 | 9 (3) | 6 (2) | 0.11 | hypothetical protein sscle\_08g068530 [Sclerotinia sclerotiorum 1980 UF-70] |
|  |  | | | | | | |

#### 9 peptide matches (6 non-duplicate, 3 duplicate)

Auto-fit to window

| Query | Dupes | Observed | Mr(expt) | Mr(calc) | ppm | M | Score | Expect | Rank | U | Peptide |
| --- | --- | --- | --- | --- | --- | --- | --- | --- | --- | --- | --- |

| Query | Dupes | Observed | Mr(expt) | Mr(calc) | ppm | M | Score | Expect | Rank | U | Peptide |
| --- | --- | --- | --- | --- | --- | --- | --- | --- | --- | --- | --- |
| 36 |  | 359.2004 | 716.3861 | 716.3817 | 6.23 | 0 | 10 | 0.65 | 1Score **> 36** indicates **identity** Score **> 21** indicates **homology** |  | K.ATAAEVR.S |
| 108 | 1 | 372.2185 | 742.4224 | 742.4225 | -0.13 | 0 | 17 | 0.45 | 1Score **> 29** indicates **identity** Score **> 26** indicates **homology** |  | R.TLLGDPK.F |
| 266 | 1 | 394.2363 | 786.4580 | 786.4599 | -2.41 | 0 | 64 | 5.3e-005 | 1Score **> 34** indicates **identity** | U | K.LGDLITR.K |
| 368 | 1 | 405.2237 | 808.4329 | 808.4555 | -28.0 | 0 | 13 | 2.6 | 3Score **> 30** indicates **identity** |  | M.PSLHLSR.V |
| 1219 |  | 467.7600 | 933.5055 | 933.5032 | 2.43 | 0 | 27 | 0.027 | 1Score **> 33** indicates **identity** Score **> 24** indicates **homology** | U | R.GLEHIHTK.Y |
| 3222 |  | 575.7861 | 1149.5577 | 1149.5700 | -10.7 | 0 | 0 | 25 | 10Score **> 34** indicates **identity** Score **> 27** indicates **homology** |  | K.ANGTMTLSDLK.N |

#### 1 subset or intersection (1 subset protein in total)

|  |  | Score | Mass | Subset of |  |
| --- | --- | --- | --- | --- | --- |
|  | gi|347835611|emb|CCD50183.1| | 27 | 62429 | 24.1 |  |
|  | similar to gamma-glutamyltransferase 2 (secreted protein) [Botrytis cinerea T4] | | | | |

|  |  | Score | Mass | Subset of |  |
| --- | --- | --- | --- | --- | --- |
|  | gi|347835611|emb|CCD50183.1| | 27 | 62429 | 24.1 | similar to gamma-glutamyltransferase 2 (secreted protein) [Botrytis cinerea T4] |
|  |  | | | | |

---

### 25

|  |  | Accession | Score | Description |
| --- | --- | --- | --- | --- |
|  | 1 | gi|154702326|gb|EDO02065.1| | 84 | hypothetical protein SS1G\_04541 [Sclerotinia sclerotiorum 1980 UF-70] |

|  |  | Score | Mass | Matches | Sequences | emPAI |  |
| --- | --- | --- | --- | --- | --- | --- | --- |
| 25.1 | gi|154702326|gb|EDO02065.1| | 84 | 78092 | 11 (5) | 7 (4) | 0.18 |  |
|  | hypothetical protein SS1G\_04541 [Sclerotinia sclerotiorum 1980 UF-70] | | | | | | |
|  | 2 samesets of gi|154702326|gb|EDO02065.1| | | | | | | |
|  | gi|156057619|ref|XP\_001594733.1| | 84 | 78092 | 11 (5) | 7 (4) | 0.18 |  |
|  | hypothetical protein SS1G\_04541 [Sclerotinia sclerotiorum 1980 UF-70] | | | | | | |
|  | gi|1095449867|gb|APA06830.1| | 84 | 80327 | 11 (5) | 7 (4) | 0.17 |  |
|  | hypothetical protein sscle\_02g016000 [Sclerotinia sclerotiorum 1980 UF-70] | | | | | | |

|  |  | Score | Mass | Matches | Sequences | emPAI |  |
| --- | --- | --- | --- | --- | --- | --- | --- |
| 25.1 | gi|154702326|gb|EDO02065.1| | 84 | 78092 | 11 (5) | 7 (4) | 0.18 | hypothetical protein SS1G\_04541 [Sclerotinia sclerotiorum 1980 UF-70] |
|  |  | | | | | | |
|  | 2 samesets of gi|154702326|gb|EDO02065.1| | | | | | | |
|  | gi|156057619|ref|XP\_001594733.1| | 84 | 78092 | 11 (5) | 7 (4) | 0.18 | hypothetical protein SS1G\_04541 [Sclerotinia sclerotiorum 1980 UF-70] |
|  |  | | | | | | |
|  | gi|1095449867|gb|APA06830.1| | 84 | 80327 | 11 (5) | 7 (4) | 0.17 | hypothetical protein sscle\_02g016000 [Sclerotinia sclerotiorum 1980 UF-70] |
|  |  | | | | | | |

#### 11 peptide matches (8 non-duplicate, 3 duplicate)

Auto-fit to window

| Query | Dupes | Observed | Mr(expt) | Mr(calc) | ppm | M | Score | Expect | Rank | U | Peptide |
| --- | --- | --- | --- | --- | --- | --- | --- | --- | --- | --- | --- |

| Query | Dupes | Observed | Mr(expt) | Mr(calc) | ppm | M | Score | Expect | Rank | U | Peptide |
| --- | --- | --- | --- | --- | --- | --- | --- | --- | --- | --- | --- |
| 47 | 1 | 361.6950 | 721.3753 | 721.3759 | -0.72 | 0 | 26 | 0.12 | 1Score **> 31** indicates **identity** Score **> 29** indicates **homology** | U | R.LAFSER.L |
| 1209 |  | 466.7616 | 931.5086 | 931.5087 | -0.070 | 0 | 18 | 0.052 | 1Score **> 33** indicates **identity** Score **> 17** indicates **homology** | U | R.GGQVTTLTR.A |
| 3870 |  | 602.8161 | 1203.6176 | 1203.6136 | 3.40 | 0 | 2 | 1 | 3Score **> 34** indicates **identity** Score **> 15** indicates **homology** | U | R.DPYVGDIAVAGK.T |
| 4455 | 2 | 626.3268 | 1250.6390 | 1250.6367 | 1.82 | 0 | 37 | 0.00081 | 1Score **> 33** indicates **identity** Score **> 19** indicates **homology** | U | R.NVLADLADHQR.A |
| 6456 |  | 726.8770 | 1451.7395 | 1451.7368 | 1.83 | 0 | 45 | 0.0003 | 1Score **> 34** indicates **identity** Score **> 22** indicates **homology** | U | R.STSILNYLNSANR.R |
| 6858 |  | 500.6033 | 1498.7881 | 1498.7892 | -0.75 | 0 | 2 | 0.75 | 1Score **> 33** indicates **identity** Score **> 13** indicates **homology** | U | K.TLFLSHNKPEASR.N |
| 6859 |  | 750.4016 | 1498.7885 | 1498.7892 | -0.44 | 0 | 29 | 0.0027 | 1Score **> 33** indicates **identity** Score **> 16** indicates **homology** | U | K.TLFLSHNKPEASR.N |
| 7513 |  | 783.8747 | 1565.7348 | 1565.7070 | 17.8 | 1 | 2 | 10 | 5Score **> 34** indicates **identity** Score **> 25** indicates **homology** | U | K.SQGQSADASRWLAR.A + 2 Deamidated (NQ); Dioxidation (W) |

---

### 26

|  |  | Accession | Score | Description |
| --- | --- | --- | --- | --- |
|  | 1 | gi|154698322|gb|EDN98060.1| | 83 | hypothetical protein SS1G\_12917 [Sclerotinia sclerotiorum 1980 UF-70] |

|  |  | Score | Mass | Matches | Sequences | emPAI |  |
| --- | --- | --- | --- | --- | --- | --- | --- |
| 26.1 | gi|154698322|gb|EDN98060.1| | 83 | 43069 | 5 (2) | 2 (2) | 0.16 |  |
|  | hypothetical protein SS1G\_12917 [Sclerotinia sclerotiorum 1980 UF-70] | | | | | | |
|  | 2 samesets of gi|154698322|gb|EDN98060.1| | | | | | | |
|  | gi|156036456|ref|XP\_001586339.1| | 83 | 43069 | 5 (2) | 2 (2) | 0.16 |  |
|  | hypothetical protein SS1G\_12917 [Sclerotinia sclerotiorum 1980 UF-70] | | | | | | |
|  | gi|1095449456|gb|APA06419.1| | 83 | 43069 | 5 (2) | 2 (2) | 0.16 |  |
|  | hypothetical protein sscle\_02g011890 [Sclerotinia sclerotiorum 1980 UF-70] | | | | | | |

|  |  | Score | Mass | Matches | Sequences | emPAI |  |
| --- | --- | --- | --- | --- | --- | --- | --- |
| 26.1 | gi|154698322|gb|EDN98060.1| | 83 | 43069 | 5 (2) | 2 (2) | 0.16 | hypothetical protein SS1G\_12917 [Sclerotinia sclerotiorum 1980 UF-70] |
|  |  | | | | | | |
|  | 2 samesets of gi|154698322|gb|EDN98060.1| | | | | | | |
|  | gi|156036456|ref|XP\_001586339.1| | 83 | 43069 | 5 (2) | 2 (2) | 0.16 | hypothetical protein SS1G\_12917 [Sclerotinia sclerotiorum 1980 UF-70] |
|  |  | | | | | | |
|  | gi|1095449456|gb|APA06419.1| | 83 | 43069 | 5 (2) | 2 (2) | 0.16 | hypothetical protein sscle\_02g011890 [Sclerotinia sclerotiorum 1980 UF-70] |
|  |  | | | | | | |

#### 5 peptide matches (4 non-duplicate, 1 duplicate)

Auto-fit to window

| Query | Dupes | Observed | Mr(expt) | Mr(calc) | ppm | M | Score | Expect | Rank | U | Peptide |
| --- | --- | --- | --- | --- | --- | --- | --- | --- | --- | --- | --- |

| Query | Dupes | Observed | Mr(expt) | Mr(calc) | ppm | M | Score | Expect | Rank | U | Peptide |
| --- | --- | --- | --- | --- | --- | --- | --- | --- | --- | --- | --- |
| 4845 | 1 | 644.8442 | 1287.6738 | 1287.6724 | 1.09 | 0 | 56 | 0.00037 | 1Score **> 34** indicates **identity** Score **> 34** indicates **homology** | U | K.SWIQPFAGQVR.I |
| 18512 |  | 1035.1588 | 3102.4546 | 3102.4492 | 1.75 | 0 | 27 | 0.43 | 1Score **> 35** indicates **identity** | U | K.SVLGFNEPDLTYEQSSNMLPEVAAQGYK.S + Oxidation (M) |
| 18514 |  | 1552.2425 | 3102.4705 | 3102.4492 | 6.89 | 0 | 63 | 0.00011 | 1Score **> 36** indicates **identity** | U | K.SVLGFNEPDLTYEQSSNMLPEVAAQGYK.S + Oxidation (M) |
| 18516 |  | 1552.7203 | 3103.4261 | 3103.4332 | -2.27 | 0 | 32 | 0.11 | 1Score **> 35** indicates **identity** | U | K.SVLGFNEPDLTYEQSSNMLPEVAAQGYK.S + Deamidated (NQ); Oxidation (M) |

---

### 27

|  |  | Accession | Score | Description |
| --- | --- | --- | --- | --- |
|  | 1 | gi|347840672|emb|CCD55244.1| | 79 | similar to extracellular dihydrogeodin oxidase/laccase [Botrytis cinerea T4] |

|  |  | Score | Mass | Matches | Sequences | emPAI |  |
| --- | --- | --- | --- | --- | --- | --- | --- |
| 27.1 | gi|347840672|emb|CCD55244.1| | 79 | 65066 | 11 (7) | 4 (1) | 0.16 |  |
|  | similar to extracellular dihydrogeodin oxidase/laccase [Botrytis cinerea T4] | | | | | | |

|  |  | Score | Mass | Matches | Sequences | emPAI |  |
| --- | --- | --- | --- | --- | --- | --- | --- |
| 27.1 | gi|347840672|emb|CCD55244.1| | 79 | 65066 | 11 (7) | 4 (1) | 0.16 | similar to extracellular dihydrogeodin oxidase/laccase [Botrytis cinerea T4] |
|  |  | | | | | | |

#### 11 peptide matches (6 non-duplicate, 5 duplicate)

Auto-fit to window

| Query | Dupes | Observed | Mr(expt) | Mr(calc) | ppm | M | Score | Expect | Rank | U | Peptide |
| --- | --- | --- | --- | --- | --- | --- | --- | --- | --- | --- | --- |

| Query | Dupes | Observed | Mr(expt) | Mr(calc) | ppm | M | Score | Expect | Rank | U | Peptide |
| --- | --- | --- | --- | --- | --- | --- | --- | --- | --- | --- | --- |
| 3354 | 1 | 581.3000 | 1160.5854 | 1160.5859 | -0.44 | 0 | 22 | 0.038 | 1Score **> 34** indicates **identity** Score **> 20** indicates **homology** | U | R.LINAGTEGMQK.F |
| 3547 | 3 | 589.2977 | 1176.5809 | 1176.5809 | 0.044 | 0 | 35 | 0.0082 | 1Score **> 34** indicates **identity** Score **> 26** indicates **homology** | U | R.LINAGTEGMQK.F + Oxidation (M) |
| 3554 | 1 | 589.7905 | 1177.5665 | 1177.5649 | 1.40 | 0 | 35 | 0.0016 | 1Score **> 34** indicates **identity** Score **> 20** indicates **homology** | U | R.LINAGTEGMQK.F + Deamidated (NQ); Oxidation (M) |
| 5599 |  | 680.3733 | 1358.7320 | 1358.7405 | -6.26 | 0 | 2 | 5.1 | 10Score **> 34** indicates **identity** Score **> 22** indicates **homology** | U | R.VLVSSDSNLIQGK.G |
| 7301 |  | 773.8876 | 1545.7607 | 1545.7821 | -13.8 | 1 | 2 | 1.2 | 4Score **> 34** indicates **identity** Score **> 16** indicates **homology** | U | R.STIAGCSLAKNPEAK.A |
| 20592 |  | 878.2086 | 3508.8051 | 3508.7249 | 22.9 | 0 | 3 | 1.5 | 4Score **> 34** indicates **identity** Score **> 17** indicates **homology** | U | R.DVILVNDVFPGPLIEANWGDTIEVTVHNEIR.G + 3 Deamidated (NQ); Dioxidation (W) |

---

### 28

|  |  | Accession | Score | Description |
| --- | --- | --- | --- | --- |
|  | 1 | gi|154691589|gb|EDN91327.1| | 78 | hypothetical protein SS1G\_00730 [Sclerotinia sclerotiorum 1980 UF-70] |

|  |  | Score | Mass | Matches | Sequences | emPAI |  |
| --- | --- | --- | --- | --- | --- | --- | --- |
| 28.1 | gi|154691589|gb|EDN91327.1| | 78 | 66638 | 10 (6) | 4 (2) | 0.10 |  |
|  | hypothetical protein SS1G\_00730 [Sclerotinia sclerotiorum 1980 UF-70] | | | | | | |
|  | 2 samesets of gi|154691589|gb|EDN91327.1| | | | | | | |
|  | gi|156065439|ref|XP\_001598641.1| | 78 | 66638 | 10 (6) | 4 (2) | 0.10 |  |
|  | hypothetical protein SS1G\_00730 [Sclerotinia sclerotiorum 1980 UF-70] | | | | | | |
|  | gi|1095450750|gb|APA07712.1| | 78 | 66638 | 10 (6) | 4 (2) | 0.10 |  |
|  | hypothetical protein sscle\_03g024820 [Sclerotinia sclerotiorum 1980 UF-70] | | | | | | |

|  |  | Score | Mass | Matches | Sequences | emPAI |  |
| --- | --- | --- | --- | --- | --- | --- | --- |
| 28.1 | gi|154691589|gb|EDN91327.1| | 78 | 66638 | 10 (6) | 4 (2) | 0.10 | hypothetical protein SS1G\_00730 [Sclerotinia sclerotiorum 1980 UF-70] |
|  |  | | | | | | |
|  | 2 samesets of gi|154691589|gb|EDN91327.1| | | | | | | |
|  | gi|156065439|ref|XP\_001598641.1| | 78 | 66638 | 10 (6) | 4 (2) | 0.10 | hypothetical protein SS1G\_00730 [Sclerotinia sclerotiorum 1980 UF-70] |
|  |  | | | | | | |
|  | gi|1095450750|gb|APA07712.1| | 78 | 66638 | 10 (6) | 4 (2) | 0.10 | hypothetical protein sscle\_03g024820 [Sclerotinia sclerotiorum 1980 UF-70] |
|  |  | | | | | | |

#### 10 peptide matches (4 non-duplicate, 6 duplicate)

Auto-fit to window

| Query | Dupes | Observed | Mr(expt) | Mr(calc) | ppm | M | Score | Expect | Rank | U | Peptide |
| --- | --- | --- | --- | --- | --- | --- | --- | --- | --- | --- | --- |

| Query | Dupes | Observed | Mr(expt) | Mr(calc) | ppm | M | Score | Expect | Rank | U | Peptide |
| --- | --- | --- | --- | --- | --- | --- | --- | --- | --- | --- | --- |
| 334 |  | 400.7528 | 799.4910 | 799.4916 | -0.73 | 0 | 43 | 0.0034 | 1Score **> 31** indicates **identity** | U | R.VLGVSGLR.V |
| 471 |  | 415.2429 | 828.4712 | 828.4705 | 0.80 | 0 | 19 | 0.2 | 1Score **> 32** indicates **identity** Score **> 25** indicates **homology** | U | R.VNAPTISK.L |
| 1522 |  | 487.2313 | 972.4481 | 972.4487 | -0.66 | 0 | 7 | 1 | 1Score **> 29** indicates **identity** Score **> 19** indicates **homology** | U | R.NFMVYQR.G + Oxidation (M) |
| 2224 | 6 | 528.2540 | 1054.4935 | 1054.4931 | 0.40 | 0 | 42 | 0.00035 | 1Score **> 31** indicates **identity** Score **> 20** indicates **homology** | U | R.ESSETSFLR.A |

#### 1 subset or intersection (1 subset protein in total)

|  |  | Score | Mass | Subset of |  |
| --- | --- | --- | --- | --- | --- |
|  | gi|347828260|emb|CCD43957.1| | 64 | 67068 | 28.1 |  |
|  | similar to GMC oxidoreductase [Botrytis cinerea T4] | | | | |

|  |  | Score | Mass | Subset of |  |
| --- | --- | --- | --- | --- | --- |
|  | gi|347828260|emb|CCD43957.1| | 64 | 67068 | 28.1 | similar to GMC oxidoreductase [Botrytis cinerea T4] |
|  |  | | | | |

---

### 29

|  |  | Accession | Score | Description |
| --- | --- | --- | --- | --- |
|  | 1 | gi|154704145|gb|EDO03884.1| | 72 | hypothetical protein SS1G\_06365 [Sclerotinia sclerotiorum 1980 UF-70] |

|  |  | Score | Mass | Matches | Sequences | emPAI |  |
| --- | --- | --- | --- | --- | --- | --- | --- |
| 29.1 | gi|154704145|gb|EDO03884.1| | 72 | 63251 | 7 (2) | 3 (2) | 0.11 |  |
|  | hypothetical protein SS1G\_06365 [Sclerotinia sclerotiorum 1980 UF-70] | | | | | | |
|  | 2 samesets of gi|154704145|gb|EDO03884.1| | | | | | | |
|  | gi|156052399|ref|XP\_001592126.1| | 72 | 63251 | 7 (2) | 3 (2) | 0.11 |  |
|  | hypothetical protein SS1G\_06365 [Sclerotinia sclerotiorum 1980 UF-70] | | | | | | |
|  | gi|1095457515|gb|APA14467.1| | 72 | 62600 | 7 (2) | 3 (2) | 0.11 |  |
|  | hypothetical protein sscle\_13g092370 [Sclerotinia sclerotiorum 1980 UF-70] | | | | | | |

|  |  | Score | Mass | Matches | Sequences | emPAI |  |
| --- | --- | --- | --- | --- | --- | --- | --- |
| 29.1 | gi|154704145|gb|EDO03884.1| | 72 | 63251 | 7 (2) | 3 (2) | 0.11 | hypothetical protein SS1G\_06365 [Sclerotinia sclerotiorum 1980 UF-70] |
|  |  | | | | | | |
|  | 2 samesets of gi|154704145|gb|EDO03884.1| | | | | | | |
|  | gi|156052399|ref|XP\_001592126.1| | 72 | 63251 | 7 (2) | 3 (2) | 0.11 | hypothetical protein SS1G\_06365 [Sclerotinia sclerotiorum 1980 UF-70] |
|  |  | | | | | | |
|  | gi|1095457515|gb|APA14467.1| | 72 | 62600 | 7 (2) | 3 (2) | 0.11 | hypothetical protein sscle\_13g092370 [Sclerotinia sclerotiorum 1980 UF-70] |
|  |  | | | | | | |

#### 7 peptide matches (3 non-duplicate, 4 duplicate)

Auto-fit to window

| Query | Dupes | Observed | Mr(expt) | Mr(calc) | ppm | M | Score | Expect | Rank | U | Peptide |
| --- | --- | --- | --- | --- | --- | --- | --- | --- | --- | --- | --- |

| Query | Dupes | Observed | Mr(expt) | Mr(calc) | ppm | M | Score | Expect | Rank | U | Peptide |
| --- | --- | --- | --- | --- | --- | --- | --- | --- | --- | --- | --- |
| 983 |  | 451.6951 | 901.3756 | 901.3752 | 0.40 | 0 | 40 | 0.00073 | 1Score **> 24** indicates **identity** Score **> 21** indicates **homology** | U | K.SGSYWMR.S + Oxidation (M) |
| 1852 | 4 | 508.2460 | 1014.4775 | 1014.4771 | 0.44 | 0 | 57 | 0.00011 | 1Score **> 30** indicates **identity** | U | R.SYDFTVQR.G |
| 3056 |  | 566.8016 | 1131.5887 | 1131.5771 | 10.2 | 0 | 6 | 1.6 | 4Score **> 34** indicates **identity** Score **> 21** indicates **homology** | U | R.LINAGTEGTQK.F + Deamidated (NQ) |

---

### 30

|  |  | Accession | Score | Description |
| --- | --- | --- | --- | --- |
|  | 1 | gi|154697112|gb|EDN96850.1| | 71 | hypothetical protein SS1G\_01776 [Sclerotinia sclerotiorum 1980 UF-70] |

|  |  | Score | Mass | Matches | Sequences | emPAI |  |
| --- | --- | --- | --- | --- | --- | --- | --- |
| 30.1 | gi|154697112|gb|EDN96850.1| | 71 | 49311 | 3 (3) | 2 (2) | 0.14 |  |
|  | hypothetical protein SS1G\_01776 [Sclerotinia sclerotiorum 1980 UF-70] | | | | | | |
|  | 3 samesets of gi|154697112|gb|EDN96850.1| | | | | | | |
|  | gi|156063320|ref|XP\_001597582.1| | 71 | 49311 | 3 (3) | 2 (2) | 0.14 |  |
|  | hypothetical protein SS1G\_01776 [Sclerotinia sclerotiorum 1980 UF-70] | | | | | | |
|  | gi|225200260|gb|ACN82436.1| | 71 | 55055 | 3 (3) | 2 (2) | 0.12 |  |
|  | alpha-amylase [Sclerotinia sclerotiorum] | | | | | | |
|  | gi|1095448879|gb|APA05843.1| | 71 | 55055 | 3 (3) | 2 (2) | 0.12 |  |
|  | hypothetical protein sscle\_01g006130 [Sclerotinia sclerotiorum 1980 UF-70] | | | | | | |

|  |  | Score | Mass | Matches | Sequences | emPAI |  |
| --- | --- | --- | --- | --- | --- | --- | --- |
| 30.1 | gi|154697112|gb|EDN96850.1| | 71 | 49311 | 3 (3) | 2 (2) | 0.14 | hypothetical protein SS1G\_01776 [Sclerotinia sclerotiorum 1980 UF-70] |
|  |  | | | | | | |
|  | 3 samesets of gi|154697112|gb|EDN96850.1| | | | | | | |
|  | gi|156063320|ref|XP\_001597582.1| | 71 | 49311 | 3 (3) | 2 (2) | 0.14 | hypothetical protein SS1G\_01776 [Sclerotinia sclerotiorum 1980 UF-70] |
|  |  | | | | | | |
|  | gi|225200260|gb|ACN82436.1| | 71 | 55055 | 3 (3) | 2 (2) | 0.12 | alpha-amylase [Sclerotinia sclerotiorum] |
|  |  | | | | | | |
|  | gi|1095448879|gb|APA05843.1| | 71 | 55055 | 3 (3) | 2 (2) | 0.12 | hypothetical protein sscle\_01g006130 [Sclerotinia sclerotiorum 1980 UF-70] |
|  |  | | | | | | |

#### 3 peptide matches (2 non-duplicate, 1 duplicate)

Auto-fit to window

| Query | Dupes | Observed | Mr(expt) | Mr(calc) | ppm | M | Score | Expect | Rank | U | Peptide |
| --- | --- | --- | --- | --- | --- | --- | --- | --- | --- | --- | --- |

| Query | Dupes | Observed | Mr(expt) | Mr(calc) | ppm | M | Score | Expect | Rank | U | Peptide |
| --- | --- | --- | --- | --- | --- | --- | --- | --- | --- | --- | --- |
| 2142 |  | 524.3008 | 1046.5870 | 1046.5873 | -0.25 | 0 | 37 | 0.01 | 1Score **> 32** indicates **identity** Score **> 30** indicates **homology** | U | R.FIASVNQIR.N |
| 5102 | 1 | 655.3424 | 1308.6701 | 1308.6674 | 2.12 | 0 | 47 | 0.00027 | 1Score **> 33** indicates **identity** Score **> 23** indicates **homology** | U | R.SQSIYQVITDR.F |

---

### 31

|  |  | Accession | Score | Description |
| --- | --- | --- | --- | --- |
|  | 1 | gi|347831150|emb|CCD46847.1| | 67 | hypothetical protein BofuT4\_P115530.1 [Botrytis cinerea T4] |

|  |  | Score | Mass | Matches | Sequences | emPAI |  |
| --- | --- | --- | --- | --- | --- | --- | --- |
| 31.1 | gi|347831150|emb|CCD46847.1| | 67 | 41337 | 1 (1) | 1 (1) | 0.08 |  |
|  | hypothetical protein BofuT4\_P115530.1 [Botrytis cinerea T4] | | | | | | |

|  |  | Score | Mass | Matches | Sequences | emPAI |  |
| --- | --- | --- | --- | --- | --- | --- | --- |
| 31.1 | gi|347831150|emb|CCD46847.1| | 67 | 41337 | 1 (1) | 1 (1) | 0.08 | hypothetical protein BofuT4\_P115530.1 [Botrytis cinerea T4] |
|  |  | | | | | | |

#### 1 peptide matches (1 non-duplicate, 0 duplicate)

Auto-fit to window

| Query | Dupes | Observed | Mr(expt) | Mr(calc) | ppm | M | Score | Expect | Rank | U | Peptide |
| --- | --- | --- | --- | --- | --- | --- | --- | --- | --- | --- | --- |

| Query | Dupes | Observed | Mr(expt) | Mr(calc) | ppm | M | Score | Expect | Rank | U | Peptide |
| --- | --- | --- | --- | --- | --- | --- | --- | --- | --- | --- | --- |
| 9839 |  | 926.8968 | 1851.7790 | 1851.7768 | 1.21 | 0 | 67 | 9.9e-006 | 1Score **> 30** indicates **identity** | U | K.GGFNMQSTTSIDCSGFK.S + Oxidation (M) |

---

### 32

|  |  | Accession | Score | Description |
| --- | --- | --- | --- | --- |
|  | 1 | gi|154698335|gb|EDN98073.1| | 63 | hypothetical protein SS1G\_12930 [Sclerotinia sclerotiorum 1980 UF-70] |

|  |  | Score | Mass | Matches | Sequences | emPAI |  |
| --- | --- | --- | --- | --- | --- | --- | --- |
| 32.1 | gi|154698335|gb|EDN98073.1| | 63 | 31996 | 3 (2) | 2 (2) | 0.22 |  |
|  | hypothetical protein SS1G\_12930 [Sclerotinia sclerotiorum 1980 UF-70] | | | | | | |
|  | 2 samesets of gi|154698335|gb|EDN98073.1| | | | | | | |
|  | gi|156036482|ref|XP\_001586352.1| | 63 | 31996 | 3 (2) | 2 (2) | 0.22 |  |
|  | hypothetical protein SS1G\_12930 [Sclerotinia sclerotiorum 1980 UF-70] | | | | | | |
|  | gi|1095449447|gb|APA06410.1| | 63 | 31996 | 3 (2) | 2 (2) | 0.22 |  |
|  | hypothetical protein sscle\_02g011800 [Sclerotinia sclerotiorum 1980 UF-70] | | | | | | |

|  |  | Score | Mass | Matches | Sequences | emPAI |  |
| --- | --- | --- | --- | --- | --- | --- | --- |
| 32.1 | gi|154698335|gb|EDN98073.1| | 63 | 31996 | 3 (2) | 2 (2) | 0.22 | hypothetical protein SS1G\_12930 [Sclerotinia sclerotiorum 1980 UF-70] |
|  |  | | | | | | |
|  | 2 samesets of gi|154698335|gb|EDN98073.1| | | | | | | |
|  | gi|156036482|ref|XP\_001586352.1| | 63 | 31996 | 3 (2) | 2 (2) | 0.22 | hypothetical protein SS1G\_12930 [Sclerotinia sclerotiorum 1980 UF-70] |
|  |  | | | | | | |
|  | gi|1095449447|gb|APA06410.1| | 63 | 31996 | 3 (2) | 2 (2) | 0.22 | hypothetical protein sscle\_02g011800 [Sclerotinia sclerotiorum 1980 UF-70] |
|  |  | | | | | | |

#### 3 peptide matches (2 non-duplicate, 1 duplicate)

Auto-fit to window

| Query | Dupes | Observed | Mr(expt) | Mr(calc) | ppm | M | Score | Expect | Rank | U | Peptide |
| --- | --- | --- | --- | --- | --- | --- | --- | --- | --- | --- | --- |

| Query | Dupes | Observed | Mr(expt) | Mr(calc) | ppm | M | Score | Expect | Rank | U | Peptide |
| --- | --- | --- | --- | --- | --- | --- | --- | --- | --- | --- | --- |
| 567 |  | 422.2554 | 842.4963 | 842.4974 | -1.27 | 0 | 57 | 0.00017 | 1Score **> 32** indicates **identity** | U | K.QALLAATR.T |
| 4764 | 1 | 641.2784 | 1280.5423 | 1280.5422 | 0.12 | 0 | 35 | 0.0066 | 1Score **> 29** indicates **identity** Score **> 26** indicates **homology** | U | K.TQADWENDFR.M |

---

### 33

|  |  | Accession | Score | Description |
| --- | --- | --- | --- | --- |
|  | 1 | gi|347841076|emb|CCD55648.1| | 61 | similar to cellobiose dehydrogenase [Botrytis cinerea T4] |

|  |  | Score | Mass | Matches | Sequences | emPAI |  |
| --- | --- | --- | --- | --- | --- | --- | --- |
| 33.1 | gi|347841076|emb|CCD55648.1| | 61 | 61015 | 3 (2) | 1 (1) | 0.05 |  |
|  | similar to cellobiose dehydrogenase [Botrytis cinerea T4] | | | | | | |

|  |  | Score | Mass | Matches | Sequences | emPAI |  |
| --- | --- | --- | --- | --- | --- | --- | --- |
| 33.1 | gi|347841076|emb|CCD55648.1| | 61 | 61015 | 3 (2) | 1 (1) | 0.05 | similar to cellobiose dehydrogenase [Botrytis cinerea T4] |
|  |  | | | | | | |

#### 3 peptide matches (1 non-duplicate, 2 duplicate)

Auto-fit to window

| Query | Dupes | Observed | Mr(expt) | Mr(calc) | ppm | M | Score | Expect | Rank | U | Peptide |
| --- | --- | --- | --- | --- | --- | --- | --- | --- | --- | --- | --- |

| Query | Dupes | Observed | Mr(expt) | Mr(calc) | ppm | M | Score | Expect | Rank | U | Peptide |
| --- | --- | --- | --- | --- | --- | --- | --- | --- | --- | --- | --- |
| 6762 | 2 | 745.9007 | 1489.7868 | 1489.7889 | -1.37 | 0 | 49 | 6.2e-005 | 1Score **> 34** indicates **identity** Score **> 20** indicates **homology** | U | R.GGPVTTYLQSALQR.S |

---

### 34

|  |  | Accession | Score | Description |
| --- | --- | --- | --- | --- |
|  | 1 | gi|154700524|gb|EDO00263.1| | 60 | hypothetical protein SS1G\_14133 [Sclerotinia sclerotiorum 1980 UF-70] |

|  |  | Score | Mass | Matches | Sequences | emPAI |  |
| --- | --- | --- | --- | --- | --- | --- | --- |
| 34.1 | gi|154700524|gb|EDO00263.1| | 60 | 32906 | 6 (3) | 3 (1) | 0.10 |  |
|  | hypothetical protein SS1G\_14133 [Sclerotinia sclerotiorum 1980 UF-70] | | | | | | |
|  | 3 samesets of gi|154700524|gb|EDO00263.1| | | | | | | |
|  | gi|156031050|ref|XP\_001584850.1| | 60 | 32906 | 6 (3) | 3 (1) | 0.10 |  |
|  | hypothetical protein SS1G\_14133 [Sclerotinia sclerotiorum 1980 UF-70] | | | | | | |
|  | gi|1095455123|gb|APA12080.1| | 60 | 32906 | 6 (3) | 3 (1) | 0.10 |  |
|  | hypothetical protein sscle\_08g068500 [Sclerotinia sclerotiorum 1980 UF-70] | | | | | | |
|  | gi|347832563|emb|CCD48260.1| | 60 | 66617 | 3 (3) | 1 (1) | 0.05 |  |
|  | hypothetical protein BofuT4\_P106440.1 [Botrytis cinerea T4] | | | | | | |

|  |  | Score | Mass | Matches | Sequences | emPAI |  |
| --- | --- | --- | --- | --- | --- | --- | --- |
| 34.1 | gi|154700524|gb|EDO00263.1| | 60 | 32906 | 6 (3) | 3 (1) | 0.10 | hypothetical protein SS1G\_14133 [Sclerotinia sclerotiorum 1980 UF-70] |
|  |  | | | | | | |
|  | 3 samesets of gi|154700524|gb|EDO00263.1| | | | | | | |
|  | gi|156031050|ref|XP\_001584850.1| | 60 | 32906 | 6 (3) | 3 (1) | 0.10 | hypothetical protein SS1G\_14133 [Sclerotinia sclerotiorum 1980 UF-70] |
|  |  | | | | | | |
|  | gi|1095455123|gb|APA12080.1| | 60 | 32906 | 6 (3) | 3 (1) | 0.10 | hypothetical protein sscle\_08g068500 [Sclerotinia sclerotiorum 1980 UF-70] |
|  |  | | | | | | |
|  | gi|347832563|emb|CCD48260.1| | 60 | 66617 | 3 (3) | 1 (1) | 0.05 | hypothetical protein BofuT4\_P106440.1 [Botrytis cinerea T4] |
|  |  | | | | | | |

#### 6 peptide matches (3 non-duplicate, 3 duplicate)

Auto-fit to window

| Query | Dupes | Observed | Mr(expt) | Mr(calc) | ppm | M | Score | Expect | Rank | U | Peptide |
| --- | --- | --- | --- | --- | --- | --- | --- | --- | --- | --- | --- |

| Query | Dupes | Observed | Mr(expt) | Mr(calc) | ppm | M | Score | Expect | Rank | U | Peptide |
| --- | --- | --- | --- | --- | --- | --- | --- | --- | --- | --- | --- |
| 714 | 2 | 433.2555 | 864.4965 | 864.4957 | 0.97 | 0 | 42 | 0.0022 | 1Score **> 28** indicates **identity** Score **> 28** indicates **homology** | U | K.LDLTYIK.Y |
| 6288 | 1 | 717.8968 | 1433.7790 | 1433.7766 | 1.71 | 0 | 9 | 1 | 1Score **> 32** indicates **identity** Score **> 22** indicates **homology** | U | K.SSNSVLPDLVYIK.T |
| 22796 |  | 808.3901 | 4036.9143 | 4036.9443 | -7.42 | 1 | 2 | 1 | 6Score **> 35** indicates **identity** Score **> 14** indicates **homology** | U | R.ILDTGTTFVEEHNGWWQLIDATGDGRPDLAYIKNK.N + 2 Dioxidation (W) |

---

### 35

|  |  | Accession | Score | Description |
| --- | --- | --- | --- | --- |
|  | 1 | gi|347836311|emb|CCD50883.1| | 56 | glycoside hydrolase family 3 protein [Botrytis cinerea T4] |

|  |  | Score | Mass | Matches | Sequences | emPAI |  |
| --- | --- | --- | --- | --- | --- | --- | --- |
| 35.1 | gi|347836311|emb|CCD50883.1| | 56 | 94046 | 7 (3) | 5 (3) | 0.11 |  |
|  | glycoside hydrolase family 3 protein [Botrytis cinerea T4] | | | | | | |

|  |  | Score | Mass | Matches | Sequences | emPAI |  |
| --- | --- | --- | --- | --- | --- | --- | --- |
| 35.1 | gi|347836311|emb|CCD50883.1| | 56 | 94046 | 7 (3) | 5 (3) | 0.11 | glycoside hydrolase family 3 protein [Botrytis cinerea T4] |
|  |  | | | | | | |

#### 7 peptide matches (6 non-duplicate, 1 duplicate)

Auto-fit to window

| Query | Dupes | Observed | Mr(expt) | Mr(calc) | ppm | M | Score | Expect | Rank | U | Peptide |
| --- | --- | --- | --- | --- | --- | --- | --- | --- | --- | --- | --- |

| Query | Dupes | Observed | Mr(expt) | Mr(calc) | ppm | M | Score | Expect | Rank | U | Peptide |
| --- | --- | --- | --- | --- | --- | --- | --- | --- | --- | --- | --- |
| 588 |  | 424.7183 | 847.4220 | 847.4222 | -0.25 | 0 | 6 | 1.1 | 2Score **> 34** indicates **identity** Score **> 19** indicates **homology** | U | R.LNDMVTR.I |
| 698 |  | 432.7164 | 863.4183 | 863.4171 | 1.40 | 0 | 36 | 0.0029 | 1Score **> 33** indicates **identity** Score **> 23** indicates **homology** | U | R.LNDMVTR.I + Oxidation (M) |
| 1042 | 1 | 456.2694 | 910.5243 | 910.5236 | 0.79 | 0 | 4 | 2.3 | 6Score **> 28** indicates **identity** Score **> 21** indicates **homology** | U | K.AITSVPPAR.A |
| 1520 |  | 486.7953 | 971.5760 | 971.5764 | -0.37 | 0 | 39 | 0.0048 | 1Score **> 32** indicates **identity** Score **> 28** indicates **homology** | U | K.QVVSLAVTR.K |
| 1676 |  | 495.7459 | 989.4772 | 989.4778 | -0.57 | 0 | 31 | 0.013 | 1Score **> 33** indicates **identity** Score **> 24** indicates **homology** | U | K.ALSSNIDDR.T |
| 24324 |  | 1652.4207 | 4954.2401 | 4954.3469 | -21.6 | 1 | 3 | 1.1 | 7Score **> 34** indicates **identity** Score **> 16** indicates **homology** | U | R.TLHEFYMWPFAEGIKAGVGAVMTSYNDVNGSAASQNSYLINNLIK.D + 2 Deamidated (NQ); 2 Oxidation (M) |

#### 2 subsets and intersections (7 subset proteins in total)

|  |  | Score | Mass | Subset of |  |
| --- | --- | --- | --- | --- | --- |
|  | gi|1095453406|gb|APA10365.1| | 43 | 94193 | 35.1 |  |
|  | hypothetical protein sscle\_06g051350 [Sclerotinia sclerotiorum 1980 UF-70] | | | | |
|  | 2 samesets of gi|1095453406|gb|APA10365.1| | | | | |
|  | gi|154704940|gb|EDO04679.1| | 43 | 94193 |  |  |
|  | hypothetical protein SS1G\_07162 [Sclerotinia sclerotiorum 1980 UF-70] | | | | |
|  | gi|156051510|ref|XP\_001591716.1| | 43 | 94193 |  |  |
|  | hypothetical protein SS1G\_07162 [Sclerotinia sclerotiorum 1980 UF-70] | | | | |
|  | gi|1095454631|gb|APA11588.1| | 36 | 105063 | 35.1 |  |
|  | hypothetical protein sscle\_08g063580 [Sclerotinia sclerotiorum 1980 UF-70] | | | | |
|  | 3 samesets of gi|1095454631|gb|APA11588.1| | | | | |
|  | gi|154702815|gb|EDO02554.1| | 36 | 105063 |  |  |
|  | hypothetical protein SS1G\_05030 [Sclerotinia sclerotiorum 1980 UF-70] | | | | |
|  | gi|156055358|ref|XP\_001593603.1| | 36 | 105063 |  |  |
|  | hypothetical protein SS1G\_05030 [Sclerotinia sclerotiorum 1980 UF-70] | | | | |
|  | gi|347827528|emb|CCD43225.1| | 36 | 105076 |  |  |
|  | glycoside hydrolase family 3 protein [Botrytis cinerea T4] | | | | |

|  |  | Score | Mass | Subset of |  |
| --- | --- | --- | --- | --- | --- |
|  | gi|1095453406|gb|APA10365.1| | 43 | 94193 | 35.1 | hypothetical protein sscle\_06g051350 [Sclerotinia sclerotiorum 1980 UF-70] |
|  |  | | | | |
|  | 2 samesets of gi|1095453406|gb|APA10365.1| | | | | |
|  | gi|154704940|gb|EDO04679.1| | 43 | 94193 |  | hypothetical protein SS1G\_07162 [Sclerotinia sclerotiorum 1980 UF-70] |
|  |  | | | | |
|  | gi|156051510|ref|XP\_001591716.1| | 43 | 94193 |  | hypothetical protein SS1G\_07162 [Sclerotinia sclerotiorum 1980 UF-70] |
|  |  | | | | |
|  | gi|1095454631|gb|APA11588.1| | 36 | 105063 | 35.1 | hypothetical protein sscle\_08g063580 [Sclerotinia sclerotiorum 1980 UF-70] |
|  |  | | | | |
|  | 3 samesets of gi|1095454631|gb|APA11588.1| | | | | |
|  | gi|154702815|gb|EDO02554.1| | 36 | 105063 |  | hypothetical protein SS1G\_05030 [Sclerotinia sclerotiorum 1980 UF-70] |
|  |  | | | | |
|  | gi|156055358|ref|XP\_001593603.1| | 36 | 105063 |  | hypothetical protein SS1G\_05030 [Sclerotinia sclerotiorum 1980 UF-70] |
|  |  | | | | |
|  | gi|347827528|emb|CCD43225.1| | 36 | 105076 |  | glycoside hydrolase family 3 protein [Botrytis cinerea T4] |
|  |  | | | | |

---

### 36

|  |  | Accession | Score | Description |
| --- | --- | --- | --- | --- |
|  | 1 | gi|154696502|gb|EDN96240.1| | 54 | predicted protein [Sclerotinia sclerotiorum 1980 UF-70] |

|  |  | Score | Mass | Matches | Sequences | emPAI |  |
| --- | --- | --- | --- | --- | --- | --- | --- |
| 36.1 | gi|154696502|gb|EDN96240.1| | 54 | 17311 | 10 (7) | 2 (1) | 0.20 |  |
|  | predicted protein [Sclerotinia sclerotiorum 1980 UF-70] | | | | | | |
|  | 2 samesets of gi|154696502|gb|EDN96240.1| | | | | | | |
|  | gi|156062100|ref|XP\_001596972.1| | 54 | 17311 | 10 (7) | 2 (1) | 0.20 |  |
|  | predicted protein [Sclerotinia sclerotiorum 1980 UF-70] | | | | | | |
|  | gi|1095449358|gb|APA06322.1| | 54 | 27475 | 10 (7) | 2 (1) | 0.12 |  |
|  | hypothetical protein sscle\_01g010920 [Sclerotinia sclerotiorum 1980 UF-70] | | | | | | |

|  |  | Score | Mass | Matches | Sequences | emPAI |  |
| --- | --- | --- | --- | --- | --- | --- | --- |
| 36.1 | gi|154696502|gb|EDN96240.1| | 54 | 17311 | 10 (7) | 2 (1) | 0.20 | predicted protein [Sclerotinia sclerotiorum 1980 UF-70] |
|  |  | | | | | | |
|  | 2 samesets of gi|154696502|gb|EDN96240.1| | | | | | | |
|  | gi|156062100|ref|XP\_001596972.1| | 54 | 17311 | 10 (7) | 2 (1) | 0.20 | predicted protein [Sclerotinia sclerotiorum 1980 UF-70] |
|  |  | | | | | | |
|  | gi|1095449358|gb|APA06322.1| | 54 | 27475 | 10 (7) | 2 (1) | 0.12 | hypothetical protein sscle\_01g010920 [Sclerotinia sclerotiorum 1980 UF-70] |
|  |  | | | | | | |

#### 10 peptide matches (2 non-duplicate, 8 duplicate)

Auto-fit to window

| Query | Dupes | Observed | Mr(expt) | Mr(calc) | ppm | M | Score | Expect | Rank | U | Peptide |
| --- | --- | --- | --- | --- | --- | --- | --- | --- | --- | --- | --- |

| Query | Dupes | Observed | Mr(expt) | Mr(calc) | ppm | M | Score | Expect | Rank | U | Peptide |
| --- | --- | --- | --- | --- | --- | --- | --- | --- | --- | --- | --- |
| 228 | 8 | 390.2415 | 778.4685 | 778.4701 | -2.08 | 0 | 24 | 0.0099 | 1Score **> 24** indicates **identity** Score **> 16** indicates **homology** | U | M.PLPLSPR.S |
| 7094 |  | 765.3759 | 1528.7373 | 1528.7310 | 4.10 | 0 | 0 | 1.1 | 3Score **> 34** indicates **identity** Score **> 13** indicates **homology** |  | R.TSYYDLHYQIAR.T |

---

### 37

|  |  | Accession | Score | Description |
| --- | --- | --- | --- | --- |
|  | 1 | gi|154691568|gb|EDN91306.1| | 53 | hypothetical protein SS1G\_00709 [Sclerotinia sclerotiorum 1980 UF-70] |

|  |  | Score | Mass | Matches | Sequences | emPAI |  |
| --- | --- | --- | --- | --- | --- | --- | --- |
| 37.1 | gi|154691568|gb|EDN91306.1| | 53 | 31398 | 1 (1) | 1 (1) | 0.11 |  |
|  | hypothetical protein SS1G\_00709 [Sclerotinia sclerotiorum 1980 UF-70] | | | | | | |
|  | 3 samesets of gi|154691568|gb|EDN91306.1| | | | | | | |
|  | gi|156065397|ref|XP\_001598620.1| | 53 | 31398 | 1 (1) | 1 (1) | 0.11 |  |
|  | hypothetical protein SS1G\_00709 [Sclerotinia sclerotiorum 1980 UF-70] | | | | | | |
|  | gi|347442007|emb|CCD34928.1| | 53 | 35551 | 1 (1) | 1 (1) | 0.09 |  |
|  | similar to transaldolase [Botrytis cinerea T4] | | | | | | |
|  | gi|1095450763|gb|APA07725.1| | 53 | 35506 | 1 (1) | 1 (1) | 0.09 |  |
|  | hypothetical protein sscle\_03g024950 [Sclerotinia sclerotiorum 1980 UF-70] | | | | | | |

|  |  | Score | Mass | Matches | Sequences | emPAI |  |
| --- | --- | --- | --- | --- | --- | --- | --- |
| 37.1 | gi|154691568|gb|EDN91306.1| | 53 | 31398 | 1 (1) | 1 (1) | 0.11 | hypothetical protein SS1G\_00709 [Sclerotinia sclerotiorum 1980 UF-70] |
|  |  | | | | | | |
|  | 3 samesets of gi|154691568|gb|EDN91306.1| | | | | | | |
|  | gi|156065397|ref|XP\_001598620.1| | 53 | 31398 | 1 (1) | 1 (1) | 0.11 | hypothetical protein SS1G\_00709 [Sclerotinia sclerotiorum 1980 UF-70] |
|  |  | | | | | | |
|  | gi|347442007|emb|CCD34928.1| | 53 | 35551 | 1 (1) | 1 (1) | 0.09 | similar to transaldolase [Botrytis cinerea T4] |
|  |  | | | | | | |
|  | gi|1095450763|gb|APA07725.1| | 53 | 35506 | 1 (1) | 1 (1) | 0.09 | hypothetical protein sscle\_03g024950 [Sclerotinia sclerotiorum 1980 UF-70] |
|  |  | | | | | | |

#### 1 peptide matches (1 non-duplicate, 0 duplicate)

Auto-fit to window

| Query | Dupes | Observed | Mr(expt) | Mr(calc) | ppm | M | Score | Expect | Rank | U | Peptide |
| --- | --- | --- | --- | --- | --- | --- | --- | --- | --- | --- | --- |

| Query | Dupes | Observed | Mr(expt) | Mr(calc) | ppm | M | Score | Expect | Rank | U | Peptide |
| --- | --- | --- | --- | --- | --- | --- | --- | --- | --- | --- | --- |
| 5014 |  | 651.8437 | 1301.6728 | 1301.6728 | 0.041 | 0 | 53 | 0.00081 | 1Score **> 34** indicates **identity** | U | K.IASTWEGIQAAR.I |

---

### 38

|  |  | Accession | Score | Description |
| --- | --- | --- | --- | --- |
|  | 1 | gi|347838742|emb|CCD53314.1| | 52 | hypothetical protein BofuT4\_P123330.1 [Botrytis cinerea T4] |

|  |  | Score | Mass | Matches | Sequences | emPAI |  |
| --- | --- | --- | --- | --- | --- | --- | --- |
| 38.1 | gi|347838742|emb|CCD53314.1| | 52 | 27295 | 7 (5) | 2 (1) | 0.12 |  |
|  | hypothetical protein BofuT4\_P123330.1 [Botrytis cinerea T4] | | | | | | |
|  | 4 samesets of gi|347838742|emb|CCD53314.1| | | | | | | |
|  | gi|154698228|gb|EDN97966.1| | 52 | 30880 | 6 (5) | 1 (1) | 0.11 |  |
|  | hypothetical protein SS1G\_12822 [Sclerotinia sclerotiorum 1980 UF-70] | | | | | | |
|  | gi|156036268|ref|XP\_001586245.1| | 52 | 30880 | 6 (5) | 1 (1) | 0.11 |  |
|  | hypothetical protein SS1G\_12822 [Sclerotinia sclerotiorum 1980 UF-70] | | | | | | |
|  | gi|347826560|emb|CCD42257.1| | 52 | 30748 | 6 (5) | 1 (1) | 0.11 |  |
|  | hypothetical protein BofuT4\_P013850.1 [Botrytis cinerea T4] | | | | | | |
|  | gi|1095449525|gb|APA06488.1| | 52 | 30880 | 6 (5) | 1 (1) | 0.11 |  |
|  | hypothetical protein sscle\_02g012580 [Sclerotinia sclerotiorum 1980 UF-70] | | | | | | |

|  |  | Score | Mass | Matches | Sequences | emPAI |  |
| --- | --- | --- | --- | --- | --- | --- | --- |
| 38.1 | gi|347838742|emb|CCD53314.1| | 52 | 27295 | 7 (5) | 2 (1) | 0.12 | hypothetical protein BofuT4\_P123330.1 [Botrytis cinerea T4] |
|  |  | | | | | | |
|  | 4 samesets of gi|347838742|emb|CCD53314.1| | | | | | | |
|  | gi|154698228|gb|EDN97966.1| | 52 | 30880 | 6 (5) | 1 (1) | 0.11 | hypothetical protein SS1G\_12822 [Sclerotinia sclerotiorum 1980 UF-70] |
|  |  | | | | | | |
|  | gi|156036268|ref|XP\_001586245.1| | 52 | 30880 | 6 (5) | 1 (1) | 0.11 | hypothetical protein SS1G\_12822 [Sclerotinia sclerotiorum 1980 UF-70] |
|  |  | | | | | | |
|  | gi|347826560|emb|CCD42257.1| | 52 | 30748 | 6 (5) | 1 (1) | 0.11 | hypothetical protein BofuT4\_P013850.1 [Botrytis cinerea T4] |
|  |  | | | | | | |
|  | gi|1095449525|gb|APA06488.1| | 52 | 30880 | 6 (5) | 1 (1) | 0.11 | hypothetical protein sscle\_02g012580 [Sclerotinia sclerotiorum 1980 UF-70] |
|  |  | | | | | | |

#### 7 peptide matches (2 non-duplicate, 5 duplicate)

Auto-fit to window

| Query | Dupes | Observed | Mr(expt) | Mr(calc) | ppm | M | Score | Expect | Rank | U | Peptide |
| --- | --- | --- | --- | --- | --- | --- | --- | --- | --- | --- | --- |

| Query | Dupes | Observed | Mr(expt) | Mr(calc) | ppm | M | Score | Expect | Rank | U | Peptide |
| --- | --- | --- | --- | --- | --- | --- | --- | --- | --- | --- | --- |
| 469 | 5 | 415.2425 | 828.4704 | 828.4705 | -0.15 | 0 | 39 | 0.011 | 1Score **> 32** indicates **identity** | U | K.IIELEGR.G |
| 5837 |  | 693.3595 | 1384.7044 | 1384.7351 | -22.1 | 0 | 9 | 2.5 | 3Score **> 33** indicates **identity** Score **> 25** indicates **homology** | U | K.WTITIPSNPTVR.F + Deamidated (NQ) |

#### 1 subset or intersection (4 subset proteins in total)

|  |  | Score | Mass | Subset of |  |
| --- | --- | --- | --- | --- | --- |
|  | gi|1095458230|gb|APA15181.1| | 47 | 111385 | 38.1 |  |
|  | hypothetical protein sscle\_14g099510 [Sclerotinia sclerotiorum 1980 UF-70] | | | | |
|  | 3 samesets of gi|1095458230|gb|APA15181.1| | | | | |
|  | gi|154693184|gb|EDN92922.1| | 47 | 113387 |  |  |
|  | hypothetical protein SS1G\_08787 [Sclerotinia sclerotiorum 1980 UF-70] | | | | |
|  | gi|156048112|ref|XP\_001590023.1| | 47 | 113387 |  |  |
|  | hypothetical protein SS1G\_08787 [Sclerotinia sclerotiorum 1980 UF-70] | | | | |
|  | gi|347832999|emb|CCD48696.1| | 47 | 111904 |  |  |
|  | hypothetical protein BofuT4\_P033380.1 [Botrytis cinerea T4] | | | | |

|  |  | Score | Mass | Subset of |  |
| --- | --- | --- | --- | --- | --- |
|  | gi|1095458230|gb|APA15181.1| | 47 | 111385 | 38.1 | hypothetical protein sscle\_14g099510 [Sclerotinia sclerotiorum 1980 UF-70] |
|  |  | | | | |
|  | 3 samesets of gi|1095458230|gb|APA15181.1| | | | | |
|  | gi|154693184|gb|EDN92922.1| | 47 | 113387 |  | hypothetical protein SS1G\_08787 [Sclerotinia sclerotiorum 1980 UF-70] |
|  |  | | | | |
|  | gi|156048112|ref|XP\_001590023.1| | 47 | 113387 |  | hypothetical protein SS1G\_08787 [Sclerotinia sclerotiorum 1980 UF-70] |
|  |  | | | | |
|  | gi|347832999|emb|CCD48696.1| | 47 | 111904 |  | hypothetical protein BofuT4\_P033380.1 [Botrytis cinerea T4] |
|  |  | | | | |

---

### 39

|  |  | Accession | Score | Description |
| --- | --- | --- | --- | --- |
|  | 1 | gi|154698875|gb|EDN98613.1| | 48 | hypothetical protein SS1G\_13472 [Sclerotinia sclerotiorum 1980 UF-70] |

|  |  | Score | Mass | Matches | Sequences | emPAI |  |
| --- | --- | --- | --- | --- | --- | --- | --- |
| 39.1 | gi|154698875|gb|EDN98613.1| | 48 | 60572 | 2 (1) | 2 (1) | 0.05 |  |
|  | hypothetical protein SS1G\_13472 [Sclerotinia sclerotiorum 1980 UF-70] | | | | | | |
|  | 2 samesets of gi|154698875|gb|EDN98613.1| | | | | | | |
|  | gi|156034338|ref|XP\_001585588.1| | 48 | 60572 | 2 (1) | 2 (1) | 0.05 |  |
|  | hypothetical protein SS1G\_13472 [Sclerotinia sclerotiorum 1980 UF-70] | | | | | | |
|  | gi|1095458571|gb|APA15521.1| | 48 | 60572 | 2 (1) | 2 (1) | 0.05 |  |
|  | hypothetical protein sscle\_15g102910 [Sclerotinia sclerotiorum 1980 UF-70] | | | | | | |

|  |  | Score | Mass | Matches | Sequences | emPAI |  |
| --- | --- | --- | --- | --- | --- | --- | --- |
| 39.1 | gi|154698875|gb|EDN98613.1| | 48 | 60572 | 2 (1) | 2 (1) | 0.05 | hypothetical protein SS1G\_13472 [Sclerotinia sclerotiorum 1980 UF-70] |
|  |  | | | | | | |
|  | 2 samesets of gi|154698875|gb|EDN98613.1| | | | | | | |
|  | gi|156034338|ref|XP\_001585588.1| | 48 | 60572 | 2 (1) | 2 (1) | 0.05 | hypothetical protein SS1G\_13472 [Sclerotinia sclerotiorum 1980 UF-70] |
|  |  | | | | | | |
|  | gi|1095458571|gb|APA15521.1| | 48 | 60572 | 2 (1) | 2 (1) | 0.05 | hypothetical protein sscle\_15g102910 [Sclerotinia sclerotiorum 1980 UF-70] |
|  |  | | | | | | |

#### 2 peptide matches (2 non-duplicate, 0 duplicate)

Auto-fit to window

| Query | Dupes | Observed | Mr(expt) | Mr(calc) | ppm | M | Score | Expect | Rank | U | Peptide |
| --- | --- | --- | --- | --- | --- | --- | --- | --- | --- | --- | --- |

| Query | Dupes | Observed | Mr(expt) | Mr(calc) | ppm | M | Score | Expect | Rank | U | Peptide |
| --- | --- | --- | --- | --- | --- | --- | --- | --- | --- | --- | --- |
| 1149 |  | 463.2674 | 924.5203 | 924.5392 | -20.5 | 1 | 2 | 2.3 | 7Score **> 29** indicates **identity** Score **> 18** indicates **homology** | U | K.KLSAELHK.R |
| 2765 |  | 554.8043 | 1107.5941 | 1107.5924 | 1.52 | 0 | 48 | 0.00064 | 1Score **> 32** indicates **identity** Score **> 29** indicates **homology** | U | R.SIYQIITDR.Y |

---

### 40

|  |  | Accession | Score | Description |
| --- | --- | --- | --- | --- |
|  | 1 | gi|347827005|emb|CCD42702.1| | 47 | hypothetical protein BofuT4\_P073140.1 [Botrytis cinerea T4] |

|  |  | Score | Mass | Matches | Sequences | emPAI |  |
| --- | --- | --- | --- | --- | --- | --- | --- |
| 40.1 | gi|347827005|emb|CCD42702.1| | 47 | 66587 | 2 (2) | 2 (2) | 0.10 |  |
|  | hypothetical protein BofuT4\_P073140.1 [Botrytis cinerea T4] | | | | | | |

|  |  | Score | Mass | Matches | Sequences | emPAI |  |
| --- | --- | --- | --- | --- | --- | --- | --- |
| 40.1 | gi|347827005|emb|CCD42702.1| | 47 | 66587 | 2 (2) | 2 (2) | 0.10 | hypothetical protein BofuT4\_P073140.1 [Botrytis cinerea T4] |
|  |  | | | | | | |

#### 2 peptide matches (2 non-duplicate, 0 duplicate)

Auto-fit to window

| Query | Dupes | Observed | Mr(expt) | Mr(calc) | ppm | M | Score | Expect | Rank | U | Peptide |
| --- | --- | --- | --- | --- | --- | --- | --- | --- | --- | --- | --- |

| Query | Dupes | Observed | Mr(expt) | Mr(calc) | ppm | M | Score | Expect | Rank | U | Peptide |
| --- | --- | --- | --- | --- | --- | --- | --- | --- | --- | --- | --- |
| 1516 |  | 486.7683 | 971.5219 | 971.5222 | -0.29 | 0 | 39 | 0.0023 | 1Score **> 32** indicates **identity** Score **> 25** indicates **homology** | U | R.MVVGNPALR.T + Oxidation (M) |
| 1961 |  | 513.7776 | 1025.5406 | 1025.5407 | -0.036 | 0 | 32 | 0.0062 | 1Score **> 30** indicates **identity** Score **> 22** indicates **homology** | U | K.ITAFQPGHR.M |

---

### 41

|  |  | Accession | Score | Description |
| --- | --- | --- | --- | --- |
|  | 1 | gi|507414638|emb|CCD56877.2| | 46 | carbohydrate-Binding Module family 20 protein [Botrytis cinerea T4] |

|  |  | Score | Mass | Matches | Sequences | emPAI |  |
| --- | --- | --- | --- | --- | --- | --- | --- |
| 41.1 | gi|507414638|emb|CCD56877.2| | 46 | 43095 | 3 (1) | 2 (1) | 0.08 |  |
|  | carbohydrate-Binding Module family 20 protein [Botrytis cinerea T4] | | | | | | |

|  |  | Score | Mass | Matches | Sequences | emPAI |  |
| --- | --- | --- | --- | --- | --- | --- | --- |
| 41.1 | gi|507414638|emb|CCD56877.2| | 46 | 43095 | 3 (1) | 2 (1) | 0.08 | carbohydrate-Binding Module family 20 protein [Botrytis cinerea T4] |
|  |  | | | | | | |

#### 3 peptide matches (2 non-duplicate, 1 duplicate)

Auto-fit to window

| Query | Dupes | Observed | Mr(expt) | Mr(calc) | ppm | M | Score | Expect | Rank | U | Peptide |
| --- | --- | --- | --- | --- | --- | --- | --- | --- | --- | --- | --- |

| Query | Dupes | Observed | Mr(expt) | Mr(calc) | ppm | M | Score | Expect | Rank | U | Peptide |
| --- | --- | --- | --- | --- | --- | --- | --- | --- | --- | --- | --- |
| 1580 | 1 | 491.2144 | 980.4142 | 980.4134 | 0.84 | 0 | 17 | 0.21 | 1Score **> 29** indicates **identity** Score **> 23** indicates **homology** |  | R.SGPCGYNAR.V |
| 3876 |  | 603.2950 | 1204.5755 | 1204.5758 | -0.22 | 0 | 46 | 0.00092 | 1Score **> 34** indicates **identity** Score **> 28** indicates **homology** | U | R.ICQDQSIVDK.L |

---

### 42

|  |  | Accession | Score | Description |
| --- | --- | --- | --- | --- |
|  | 1 | gi|154702896|gb|EDO02635.1| | 44 | laccase precursor [Sclerotinia sclerotiorum 1980 UF-70] |

|  |  | Score | Mass | Matches | Sequences | emPAI |  |
| --- | --- | --- | --- | --- | --- | --- | --- |
| 42.1 | gi|154702896|gb|EDO02635.1| | 44 | 66133 | 9 (3) | 4 (3) | 0.16 |  |
|  | laccase precursor [Sclerotinia sclerotiorum 1980 UF-70] | | | | | | |
|  | 2 samesets of gi|154702896|gb|EDO02635.1| | | | | | | |
|  | gi|156055520|ref|XP\_001593684.1| | 44 | 66133 | 9 (3) | 4 (3) | 0.16 |  |
|  | laccase precursor [Sclerotinia sclerotiorum 1980 UF-70] | | | | | | |
|  | gi|1095454699|gb|APA11656.1| | 44 | 66005 | 9 (3) | 4 (3) | 0.16 |  |
|  | hypothetical protein sscle\_08g064260 [Sclerotinia sclerotiorum 1980 UF-70] | | | | | | |

|  |  | Score | Mass | Matches | Sequences | emPAI |  |
| --- | --- | --- | --- | --- | --- | --- | --- |
| 42.1 | gi|154702896|gb|EDO02635.1| | 44 | 66133 | 9 (3) | 4 (3) | 0.16 | laccase precursor [Sclerotinia sclerotiorum 1980 UF-70] |
|  |  | | | | | | |
|  | 2 samesets of gi|154702896|gb|EDO02635.1| | | | | | | |
|  | gi|156055520|ref|XP\_001593684.1| | 44 | 66133 | 9 (3) | 4 (3) | 0.16 | laccase precursor [Sclerotinia sclerotiorum 1980 UF-70] |
|  |  | | | | | | |
|  | gi|1095454699|gb|APA11656.1| | 44 | 66005 | 9 (3) | 4 (3) | 0.16 | hypothetical protein sscle\_08g064260 [Sclerotinia sclerotiorum 1980 UF-70] |
|  |  | | | | | | |

#### 9 peptide matches (6 non-duplicate, 3 duplicate)

Auto-fit to window

| Query | Dupes | Observed | Mr(expt) | Mr(calc) | ppm | M | Score | Expect | Rank | U | Peptide |
| --- | --- | --- | --- | --- | --- | --- | --- | --- | --- | --- | --- |

| Query | Dupes | Observed | Mr(expt) | Mr(calc) | ppm | M | Score | Expect | Rank | U | Peptide |
| --- | --- | --- | --- | --- | --- | --- | --- | --- | --- | --- | --- |
| 1869 |  | 508.7556 | 1015.4965 | 1015.4975 | -0.89 | 0 | 42 | 0.0013 | 1Score **> 32** indicates **identity** Score **> 25** indicates **homology** | U | R.YYDLTVSR.S |
| 2414 | 1 | 538.2913 | 1074.5680 | 1074.5669 | 0.97 | 0 | 21 | 0.14 | 1Score **> 34** indicates **identity** Score **> 25** indicates **homology** | U | R.LINAGSSGTQK.F |
| 2419 | 1 | 538.7833 | 1075.5520 | 1075.5509 | 0.96 | 0 | 11 | 0.8 | 3Score **> 34** indicates **identity** Score **> 22** indicates **homology** | U | R.LINAGSSGTQK.F + Deamidated (NQ) |
| 2760 | 1 | 554.7416 | 1107.4687 | 1107.4689 | -0.14 | 0 | 17 | 0.2 | 1Score **> 30** indicates **identity** Score **> 22** indicates **homology** | U | R.SDLDVPCMR.L + Oxidation (M) |
| 10332 |  | 954.9949 | 1907.9752 | 1907.9741 | 0.56 | 0 | 36 | 0.037 | 1Score **> 35** indicates **identity** | U | K.VPNGPVFPNVDNNLINGK.G + Deamidated (NQ) |
| 10343 |  | 955.4890 | 1908.9633 | 1908.9581 | 2.73 | 0 | 7 | 1 | 1Score **> 35** indicates **identity** Score **> 19** indicates **homology** | U | K.VPNGPVFPNVDNNLINGK.G + 2 Deamidated (NQ) |

#### 1 subset or intersection (1 subset protein in total)

|  |  | Score | Mass | Subset of |  |
| --- | --- | --- | --- | --- | --- |
|  | gi|347840621|emb|CCD55193.1| | 42 | 66446 | 42.1 |  |
|  | similar to extracellular dihydrogeodin oxidase/laccase [Botrytis cinerea T4] | | | | |

|  |  | Score | Mass | Subset of |  |
| --- | --- | --- | --- | --- | --- |
|  | gi|347840621|emb|CCD55193.1| | 42 | 66446 | 42.1 | similar to extracellular dihydrogeodin oxidase/laccase [Botrytis cinerea T4] |
|  |  | | | | |

---

### 43

|  |  | Accession | Score | Description |
| --- | --- | --- | --- | --- |
|  | 1 | gi|347827354|emb|CCD43051.1| | 43 | similar to phosphatidylserine decarboxylase [Botrytis cinerea T4] |

|  |  | Score | Mass | Matches | Sequences | emPAI |  |
| --- | --- | --- | --- | --- | --- | --- | --- |
| 43.1 | gi|347827354|emb|CCD43051.1| | 43 | 46590 | 8 (1) | 6 (1) | 0.07 |  |
|  | similar to phosphatidylserine decarboxylase [Botrytis cinerea T4] | | | | | | |
|  | 3 samesets of gi|347827354|emb|CCD43051.1| | | | | | | |
|  | gi|154702348|gb|EDO02087.1| | 43 | 47479 | 3 (1) | 2 (1) | 0.07 |  |
|  | phosphatidylserine decarboxylase [Sclerotinia sclerotiorum 1980 UF-70] | | | | | | |
|  | gi|156057663|ref|XP\_001594755.1| | 43 | 47479 | 3 (1) | 2 (1) | 0.07 |  |
|  | phosphatidylserine decarboxylase [Sclerotinia sclerotiorum 1980 UF-70] | | | | | | |
|  | gi|1095449848|gb|APA06811.1| | 43 | 47479 | 3 (1) | 2 (1) | 0.07 |  |
|  | hypothetical protein sscle\_02g015810 [Sclerotinia sclerotiorum 1980 UF-70] | | | | | | |

|  |  | Score | Mass | Matches | Sequences | emPAI |  |
| --- | --- | --- | --- | --- | --- | --- | --- |
| 43.1 | gi|347827354|emb|CCD43051.1| | 43 | 46590 | 8 (1) | 6 (1) | 0.07 | similar to phosphatidylserine decarboxylase [Botrytis cinerea T4] |
|  |  | | | | | | |
|  | 3 samesets of gi|347827354|emb|CCD43051.1| | | | | | | |
|  | gi|154702348|gb|EDO02087.1| | 43 | 47479 | 3 (1) | 2 (1) | 0.07 | phosphatidylserine decarboxylase [Sclerotinia sclerotiorum 1980 UF-70] |
|  |  | | | | | | |
|  | gi|156057663|ref|XP\_001594755.1| | 43 | 47479 | 3 (1) | 2 (1) | 0.07 | phosphatidylserine decarboxylase [Sclerotinia sclerotiorum 1980 UF-70] |
|  |  | | | | | | |
|  | gi|1095449848|gb|APA06811.1| | 43 | 47479 | 3 (1) | 2 (1) | 0.07 | hypothetical protein sscle\_02g015810 [Sclerotinia sclerotiorum 1980 UF-70] |
|  |  | | | | | | |

#### 8 peptide matches (7 non-duplicate, 1 duplicate)

Auto-fit to window

| Query | Dupes | Observed | Mr(expt) | Mr(calc) | ppm | M | Score | Expect | Rank | U | Peptide |
| --- | --- | --- | --- | --- | --- | --- | --- | --- | --- | --- | --- |

| Query | Dupes | Observed | Mr(expt) | Mr(calc) | ppm | M | Score | Expect | Rank | U | Peptide |
| --- | --- | --- | --- | --- | --- | --- | --- | --- | --- | --- | --- |
| 2563 |  | 546.2730 | 1090.5315 | 1090.5295 | 1.84 | 1 | 5 | 1.5 | 4Score **> 34** indicates **identity** Score **> 19** indicates **homology** | U | K.EKVPNFDNK.T + Deamidated (NQ) |
| 3051 |  | 566.7774 | 1131.5402 | 1131.5408 | -0.48 | 0 | 43 | 0.00049 | 1Score **> 33** indicates **identity** Score **> 23** indicates **homology** | U | R.EVDDLDTAVR.N |
| 3444 | 1 | 584.8052 | 1167.5958 | 1167.6149 | -16.3 | 1 | 2 | 1 | 1Score **> 32** indicates **identity** Score **> 14** indicates **homology** | U | K.KAGGHNFLAPR.R + Deamidated (NQ) |
| 5617 |  | 682.3353 | 1362.6560 | 1362.6456 | 7.66 | 1 | 13 | 1.2 | 2Score **> 34** indicates **identity** Score **> 26** indicates **homology** | U | R.VFEAEDPKNWK.T + Deamidated (NQ) |
| 9025 |  | 585.0006 | 1751.9800 | 1751.9570 | 13.1 | 0 | 10 | 1 | 1Score **> 30** indicates **identity** Score **> 22** indicates **homology** | U | K.NVNKPLTPLSDWLVR.F + Deamidated (NQ) |
| 22783 |  | 808.1889 | 4035.9081 | 4035.8591 | 12.1 | 2 | 2 | 1.1 | 7Score **> 35** indicates **identity** Score **> 15** indicates **homology** | U | K.EKVPNFDNKTTYGELFQEGIWTHSFLNTFDYHR.Q + 3 Deamidated (NQ) |
| 22794 |  | 808.3888 | 4036.9076 | 4036.8432 | 16.0 | 2 | 4 | 0.72 | 1Score **> 35** indicates **identity** Score **> 16** indicates **homology** | U | K.EKVPNFDNKTTYGELFQEGIWTHSFLNTFDYHR.Q + 4 Deamidated (NQ) |

---

### 44

|  |  | Accession | Score | Description |
| --- | --- | --- | --- | --- |
|  | 1 | gi|1095454060|gb|APA11018.1| | 42 | hypothetical protein sscle\_07g057880 [Sclerotinia sclerotiorum 1980 UF-70] |

|  |  | Score | Mass | Matches | Sequences | emPAI |  |
| --- | --- | --- | --- | --- | --- | --- | --- |
| 44.1 | gi|1095454060|gb|APA11018.1| | 42 | 42363 | 2 (1) | 1 (1) | 0.08 |  |
|  | hypothetical protein sscle\_07g057880 [Sclerotinia sclerotiorum 1980 UF-70] | | | | | | |

|  |  | Score | Mass | Matches | Sequences | emPAI |  |
| --- | --- | --- | --- | --- | --- | --- | --- |
| 44.1 | gi|1095454060|gb|APA11018.1| | 42 | 42363 | 2 (1) | 1 (1) | 0.08 | hypothetical protein sscle\_07g057880 [Sclerotinia sclerotiorum 1980 UF-70] |
|  |  | | | | | | |

#### 2 peptide matches (1 non-duplicate, 1 duplicate)

Auto-fit to window

| Query | Dupes | Observed | Mr(expt) | Mr(calc) | ppm | M | Score | Expect | Rank | U | Peptide |
| --- | --- | --- | --- | --- | --- | --- | --- | --- | --- | --- | --- |

| Query | Dupes | Observed | Mr(expt) | Mr(calc) | ppm | M | Score | Expect | Rank | U | Peptide |
| --- | --- | --- | --- | --- | --- | --- | --- | --- | --- | --- | --- |
| 2584 | 1 | 547.2519 | 1092.4893 | 1092.4910 | -1.50 | 0 | 42 | 0.00054 | 1Score **> 32** indicates **identity** Score **> 22** indicates **homology** | U | R.EYAPMDVPR.H + Oxidation (M) |

---

### 45

|  |  | Accession | Score | Description |
| --- | --- | --- | --- | --- |
|  | 1 | gi|1095457170|gb|APA14123.1| | 41 | hypothetical protein sscle\_12g088930 [Sclerotinia sclerotiorum 1980 UF-70] |

|  |  | Score | Mass | Matches | Sequences | emPAI |  |
| --- | --- | --- | --- | --- | --- | --- | --- |
| 45.1 | gi|1095457170|gb|APA14123.1| | 41 | 24866 | 4 (1) | 3 (1) | 0.13 |  |
|  | hypothetical protein sscle\_12g088930 [Sclerotinia sclerotiorum 1980 UF-70] | | | | | | |
|  | 11 samesets of gi|1095457170|gb|APA14123.1| | | | | | | |
|  | gi|154695422|gb|EDN95160.1| | 41 | 17973 | 3 (1) | 2 (1) | 0.19 |  |
|  | ubiquitin fusion protein [Sclerotinia sclerotiorum 1980 UF-70] | | | | | | |
|  | gi|154697207|gb|EDN96945.1| | 41 | 14842 | 3 (1) | 2 (1) | 0.23 |  |
|  | ubiquitin fusion protein [Sclerotinia sclerotiorum 1980 UF-70] | | | | | | |
|  | gi|156042476|ref|XP\_001587795.1| | 41 | 17973 | 3 (1) | 2 (1) | 0.19 |  |
|  | ubiquitin-40S ribosomal protein S31 fusion protein [Sclerotinia sclerotiorum 1980 UF-70] | | | | | | |
|  | gi|156063510|ref|XP\_001597677.1| | 41 | 14842 | 3 (1) | 2 (1) | 0.23 |  |
|  | ubiquitin-60S ribosomal protein L40 fusion protein [Sclerotinia sclerotiorum 1980 UF-70] | | | | | | |
|  | gi|347841176|emb|CCD55748.1| | 41 | 17973 | 3 (1) | 2 (1) | 0.19 |  |
|  | similar to ubiquitin/40S ribosomal protein S27a [Botrytis cinerea T4] | | | | | | |
|  | gi|154691033|gb|EDN90771.1| | 41 | 34242 | 4 (1) | 3 (1) | 0.10 |  |
|  | polyubiquitin [Sclerotinia sclerotiorum 1980 UF-70] | | | | | | |
|  | gi|156064327|ref|XP\_001598085.1| | 41 | 34242 | 4 (1) | 3 (1) | 0.10 |  |
|  | polyubiquitin [Sclerotinia sclerotiorum 1980 UF-70] | | | | | | |
|  | gi|347836850|emb|CCD51422.1| | 41 | 34216 | 4 (1) | 3 (1) | 0.10 |  |
|  | similar to polyubiquitin protein [Botrytis cinerea T4] | | | | | | |
|  | gi|1095451191|gb|APA08153.1| | 41 | 34242 | 4 (1) | 3 (1) | 0.10 |  |
|  | hypothetical protein sscle\_03g029230 [Sclerotinia sclerotiorum 1980 UF-70] | | | | | | |
|  | gi|347830393|emb|CCD46090.1| | 41 | 19033 | 2 (1) | 1 (1) | 0.18 |  |
|  | hypothetical protein BofuT4\_P116400.1 [Botrytis cinerea T4] | | | | | | |
|  | gi|1095448800|gb|APA05764.1| | 41 | 15308 | 2 (1) | 1 (1) | 0.22 |  |
|  | hypothetical protein sscle\_01g005340 [Sclerotinia sclerotiorum 1980 UF-70] | | | | | | |

|  |  | Score | Mass | Matches | Sequences | emPAI |  |
| --- | --- | --- | --- | --- | --- | --- | --- |
| 45.1 | gi|1095457170|gb|APA14123.1| | 41 | 24866 | 4 (1) | 3 (1) | 0.13 | hypothetical protein sscle\_12g088930 [Sclerotinia sclerotiorum 1980 UF-70] |
|  |  | | | | | | |
|  | 11 samesets of gi|1095457170|gb|APA14123.1| | | | | | | |
|  | gi|154695422|gb|EDN95160.1| | 41 | 17973 | 3 (1) | 2 (1) | 0.19 | ubiquitin fusion protein [Sclerotinia sclerotiorum 1980 UF-70] |
|  |  | | | | | | |
|  | gi|154697207|gb|EDN96945.1| | 41 | 14842 | 3 (1) | 2 (1) | 0.23 | ubiquitin fusion protein [Sclerotinia sclerotiorum 1980 UF-70] |
|  |  | | | | | | |
|  | gi|156042476|ref|XP\_001587795.1| | 41 | 17973 | 3 (1) | 2 (1) | 0.19 | ubiquitin-40S ribosomal protein S31 fusion protein [Sclerotinia sclerotiorum 1980 UF-70] |
|  |  | | | | | | |
|  | gi|156063510|ref|XP\_001597677.1| | 41 | 14842 | 3 (1) | 2 (1) | 0.23 | ubiquitin-60S ribosomal protein L40 fusion protein [Sclerotinia sclerotiorum 1980 UF-70] |
|  |  | | | | | | |
|  | gi|347841176|emb|CCD55748.1| | 41 | 17973 | 3 (1) | 2 (1) | 0.19 | similar to ubiquitin/40S ribosomal protein S27a [Botrytis cinerea T4] |
|  |  | | | | | | |
|  | gi|154691033|gb|EDN90771.1| | 41 | 34242 | 4 (1) | 3 (1) | 0.10 | polyubiquitin [Sclerotinia sclerotiorum 1980 UF-70] |
|  |  | | | | | | |
|  | gi|156064327|ref|XP\_001598085.1| | 41 | 34242 | 4 (1) | 3 (1) | 0.10 | polyubiquitin [Sclerotinia sclerotiorum 1980 UF-70] |
|  |  | | | | | | |
|  | gi|347836850|emb|CCD51422.1| | 41 | 34216 | 4 (1) | 3 (1) | 0.10 | similar to polyubiquitin protein [Botrytis cinerea T4] |
|  |  | | | | | | |
|  | gi|1095451191|gb|APA08153.1| | 41 | 34242 | 4 (1) | 3 (1) | 0.10 | hypothetical protein sscle\_03g029230 [Sclerotinia sclerotiorum 1980 UF-70] |
|  |  | | | | | | |
|  | gi|347830393|emb|CCD46090.1| | 41 | 19033 | 2 (1) | 1 (1) | 0.18 | hypothetical protein BofuT4\_P116400.1 [Botrytis cinerea T4] |
|  |  | | | | | | |
|  | gi|1095448800|gb|APA05764.1| | 41 | 15308 | 2 (1) | 1 (1) | 0.22 | hypothetical protein sscle\_01g005340 [Sclerotinia sclerotiorum 1980 UF-70] |
|  |  | | | | | | |

#### 4 peptide matches (3 non-duplicate, 1 duplicate)

Auto-fit to window

| Query | Dupes | Observed | Mr(expt) | Mr(calc) | ppm | M | Score | Expect | Rank | U | Peptide |
| --- | --- | --- | --- | --- | --- | --- | --- | --- | --- | --- | --- |

| Query | Dupes | Observed | Mr(expt) | Mr(calc) | ppm | M | Score | Expect | Rank | U | Peptide |
| --- | --- | --- | --- | --- | --- | --- | --- | --- | --- | --- | --- |
| 2325 | 1 | 534.3144 | 1066.6143 | 1066.6135 | 0.81 | 0 | 41 | 0.0019 | 1Score **> 28** indicates **identity** Score **> 26** indicates **homology** | U | K.ESTLHLVLR.L |
| 2675 |  | 550.3094 | 1098.6042 | 1098.5822 | 20.1 | 0 | 5 | 2.4 | 3Score **> 31** indicates **identity** Score **> 22** indicates **homology** | U | M.VSPNLLNWR.V + Deamidated (NQ) |
| 22588 |  | 796.6243 | 3978.0850 | 3978.1374 | -13.2 | 2 | 3 | 0.98 | 1Score **> 32** indicates **identity** Score **> 15** indicates **homology** | U | K.TIQIIVQAVMQIFVKTLTGKTITLEVESSDTIDNVK.A + 3 Deamidated (NQ) |

---

### 46

|  |  | Accession | Score | Description |
| --- | --- | --- | --- | --- |
|  | 1 | gi|347838722|emb|CCD53294.1| | 40 | hypothetical protein BofuT4\_P123130.1 [Botrytis cinerea T4] |

|  |  | Score | Mass | Matches | Sequences | emPAI |  |
| --- | --- | --- | --- | --- | --- | --- | --- |
| 46.1 | gi|347838722|emb|CCD53294.1| | 40 | 21674 | 2 (2) | 1 (1) | 0.16 |  |
|  | hypothetical protein BofuT4\_P123130.1 [Botrytis cinerea T4] | | | | | | |

|  |  | Score | Mass | Matches | Sequences | emPAI |  |
| --- | --- | --- | --- | --- | --- | --- | --- |
| 46.1 | gi|347838722|emb|CCD53294.1| | 40 | 21674 | 2 (2) | 1 (1) | 0.16 | hypothetical protein BofuT4\_P123130.1 [Botrytis cinerea T4] |
|  |  | | | | | | |

#### 2 peptide matches (1 non-duplicate, 1 duplicate)

Auto-fit to window

| Query | Dupes | Observed | Mr(expt) | Mr(calc) | ppm | M | Score | Expect | Rank | U | Peptide |
| --- | --- | --- | --- | --- | --- | --- | --- | --- | --- | --- | --- |

| Query | Dupes | Observed | Mr(expt) | Mr(calc) | ppm | M | Score | Expect | Rank | U | Peptide |
| --- | --- | --- | --- | --- | --- | --- | --- | --- | --- | --- | --- |
| 1612 | 1 | 492.7467 | 983.4788 | 983.4753 | 3.64 | 0 | 35 | 0.02 | 1Score **> 31** indicates **identity** | U | K.FWDFELK.Q |

---

### 47

|  |  | Accession | Score | Description |
| --- | --- | --- | --- | --- |
|  | 1 | gi|154693234|gb|EDN92972.1| | 40 | hypothetical protein SS1G\_08837 [Sclerotinia sclerotiorum 1980 UF-70] |

|  |  | Score | Mass | Matches | Sequences | emPAI |  |
| --- | --- | --- | --- | --- | --- | --- | --- |
| 47.1 | gi|154693234|gb|EDN92972.1| | 40 | 44896 | 1 (1) | 1 (1) | 0.07 |  |
|  | hypothetical protein SS1G\_08837 [Sclerotinia sclerotiorum 1980 UF-70] | | | | | | |
|  | 2 samesets of gi|154693234|gb|EDN92972.1| | | | | | | |
|  | gi|156048212|ref|XP\_001590073.1| | 40 | 44896 | 1 (1) | 1 (1) | 0.07 |  |
|  | hypothetical protein SS1G\_08837 [Sclerotinia sclerotiorum 1980 UF-70] | | | | | | |
|  | gi|1095458271|gb|APA15222.1| | 40 | 44896 | 1 (1) | 1 (1) | 0.07 |  |
|  | hypothetical protein sscle\_14g099920 [Sclerotinia sclerotiorum 1980 UF-70] | | | | | | |

|  |  | Score | Mass | Matches | Sequences | emPAI |  |
| --- | --- | --- | --- | --- | --- | --- | --- |
| 47.1 | gi|154693234|gb|EDN92972.1| | 40 | 44896 | 1 (1) | 1 (1) | 0.07 | hypothetical protein SS1G\_08837 [Sclerotinia sclerotiorum 1980 UF-70] |
|  |  | | | | | | |
|  | 2 samesets of gi|154693234|gb|EDN92972.1| | | | | | | |
|  | gi|156048212|ref|XP\_001590073.1| | 40 | 44896 | 1 (1) | 1 (1) | 0.07 | hypothetical protein SS1G\_08837 [Sclerotinia sclerotiorum 1980 UF-70] |
|  |  | | | | | | |
|  | gi|1095458271|gb|APA15222.1| | 40 | 44896 | 1 (1) | 1 (1) | 0.07 | hypothetical protein sscle\_14g099920 [Sclerotinia sclerotiorum 1980 UF-70] |
|  |  | | | | | | |

#### 1 peptide matches (1 non-duplicate, 0 duplicate)

Auto-fit to window

| Query | Dupes | Observed | Mr(expt) | Mr(calc) | ppm | M | Score | Expect | Rank | U | Peptide |
| --- | --- | --- | --- | --- | --- | --- | --- | --- | --- | --- | --- |

| Query | Dupes | Observed | Mr(expt) | Mr(calc) | ppm | M | Score | Expect | Rank | U | Peptide |
| --- | --- | --- | --- | --- | --- | --- | --- | --- | --- | --- | --- |
| 2361 |  | 536.2989 | 1070.5833 | 1070.5832 | 0.12 | 0 | 40 | 0.004 | 1Score **> 32** indicates **identity** Score **> 28** indicates **homology** | U | K.NNQAAITAIR.K |

---

### 48

|  |  | Accession | Score | Description |
| --- | --- | --- | --- | --- |
|  | 1 | gi|154703307|gb|EDO03046.1| | 39 | hypothetical protein SS1G\_05524 [Sclerotinia sclerotiorum 1980 UF-70] |

|  |  | Score | Mass | Matches | Sequences | emPAI |  |
| --- | --- | --- | --- | --- | --- | --- | --- |
| 48.1 | gi|154703307|gb|EDO03046.1| | 39 | 141121 | 8 (3) | 5 (1) | 0.02 |  |
|  | hypothetical protein SS1G\_05524 [Sclerotinia sclerotiorum 1980 UF-70] | | | | | | |
|  | 2 samesets of gi|154703307|gb|EDO03046.1| | | | | | | |
|  | gi|156056342|ref|XP\_001594095.1| | 39 | 141121 | 8 (3) | 5 (1) | 0.02 |  |
|  | hypothetical protein SS1G\_05524 [Sclerotinia sclerotiorum 1980 UF-70] | | | | | | |
|  | gi|1095455005|gb|APA11962.1| | 39 | 141121 | 8 (3) | 5 (1) | 0.02 |  |
|  | hypothetical protein sscle\_08g067320 [Sclerotinia sclerotiorum 1980 UF-70] | | | | | | |

|  |  | Score | Mass | Matches | Sequences | emPAI |  |
| --- | --- | --- | --- | --- | --- | --- | --- |
| 48.1 | gi|154703307|gb|EDO03046.1| | 39 | 141121 | 8 (3) | 5 (1) | 0.02 | hypothetical protein SS1G\_05524 [Sclerotinia sclerotiorum 1980 UF-70] |
|  |  | | | | | | |
|  | 2 samesets of gi|154703307|gb|EDO03046.1| | | | | | | |
|  | gi|156056342|ref|XP\_001594095.1| | 39 | 141121 | 8 (3) | 5 (1) | 0.02 | hypothetical protein SS1G\_05524 [Sclerotinia sclerotiorum 1980 UF-70] |
|  |  | | | | | | |
|  | gi|1095455005|gb|APA11962.1| | 39 | 141121 | 8 (3) | 5 (1) | 0.02 | hypothetical protein sscle\_08g067320 [Sclerotinia sclerotiorum 1980 UF-70] |
|  |  | | | | | | |

#### 8 peptide matches (6 non-duplicate, 2 duplicate)

Auto-fit to window

| Query | Dupes | Observed | Mr(expt) | Mr(calc) | ppm | M | Score | Expect | Rank | U | Peptide |
| --- | --- | --- | --- | --- | --- | --- | --- | --- | --- | --- | --- |

| Query | Dupes | Observed | Mr(expt) | Mr(calc) | ppm | M | Score | Expect | Rank | U | Peptide |
| --- | --- | --- | --- | --- | --- | --- | --- | --- | --- | --- | --- |
| 251 |  | 392.7192 | 783.4238 | 783.4351 | -14.4 | 0 | 9 | 1.2 | 2Score **> 27** indicates **identity** Score **> 23** indicates **homology** |  | R.LRPDQR.A |
| 1549 |  | 488.7279 | 975.4412 | 975.4257 | 15.8 | 0 | 10 | 7.7 | 6Score **> 31** indicates **identity** |  | R.VNDANGQTR.F + 2 Deamidated (NQ) |
| 1728 |  | 500.2830 | 998.5514 | 998.5509 | 0.57 | 1 | 9 | 0.23 | 1Score **> 31** indicates **identity** Score **> 15** indicates **homology** |  | K.KSPAAVAAQR.G + Deamidated (NQ) |
| 9049 | 2 | 879.4186 | 1756.8227 | 1756.8479 | -14.3 | 1 | 32 | 0.002 | 1Score **> 34** indicates **identity** Score **> 18** indicates **homology** | U | R.LELLQQAKDHSSNGSK.E + 3 Deamidated (NQ) |
| 15526 |  | 1377.2124 | 2752.4102 | 2752.3879 | 8.13 | 1 | 1 | 1 | 3Score **> 34** indicates **identity** Score **> 13** indicates **homology** | U | R.GIQEKAVLGNASQQGIVNANPSDALAGK.E + 3 Deamidated (NQ) |
| 15543 |  | 1377.7113 | 2753.4080 | 2753.3719 | 13.1 | 1 | 0 | 1 | 2Score **> 34** indicates **identity** Score **> 13** indicates **homology** | U | R.GIQEKAVLGNASQQGIVNANPSDALAGK.E + 4 Deamidated (NQ) |

---

### 49

|  |  | Accession | Score | Description |
| --- | --- | --- | --- | --- |
|  | 1 | gi|154691708|gb|EDN91446.1| | 38 | hypothetical protein SS1G\_00849 [Sclerotinia sclerotiorum 1980 UF-70] |

|  |  | Score | Mass | Matches | Sequences | emPAI |  |
| --- | --- | --- | --- | --- | --- | --- | --- |
| 49.1 | gi|154691708|gb|EDN91446.1| | 38 | 16672 | 2 (2) | 1 (1) | 0.20 |  |
|  | hypothetical protein SS1G\_00849 [Sclerotinia sclerotiorum 1980 UF-70] | | | | | | |
|  | 2 samesets of gi|154691708|gb|EDN91446.1| | | | | | | |
|  | gi|156065677|ref|XP\_001598760.1| | 38 | 16672 | 2 (2) | 1 (1) | 0.20 |  |
|  | hypothetical protein SS1G\_00849 [Sclerotinia sclerotiorum 1980 UF-70] | | | | | | |
|  | gi|1095450668|gb|APA07630.1| | 38 | 16672 | 2 (2) | 1 (1) | 0.20 |  |
|  | hypothetical protein sscle\_03g024000 [Sclerotinia sclerotiorum 1980 UF-70] | | | | | | |

|  |  | Score | Mass | Matches | Sequences | emPAI |  |
| --- | --- | --- | --- | --- | --- | --- | --- |
| 49.1 | gi|154691708|gb|EDN91446.1| | 38 | 16672 | 2 (2) | 1 (1) | 0.20 | hypothetical protein SS1G\_00849 [Sclerotinia sclerotiorum 1980 UF-70] |
|  |  | | | | | | |
|  | 2 samesets of gi|154691708|gb|EDN91446.1| | | | | | | |
|  | gi|156065677|ref|XP\_001598760.1| | 38 | 16672 | 2 (2) | 1 (1) | 0.20 | hypothetical protein SS1G\_00849 [Sclerotinia sclerotiorum 1980 UF-70] |
|  |  | | | | | | |
|  | gi|1095450668|gb|APA07630.1| | 38 | 16672 | 2 (2) | 1 (1) | 0.20 | hypothetical protein sscle\_03g024000 [Sclerotinia sclerotiorum 1980 UF-70] |
|  |  | | | | | | |

#### 2 peptide matches (1 non-duplicate, 1 duplicate)

Auto-fit to window

| Query | Dupes | Observed | Mr(expt) | Mr(calc) | ppm | M | Score | Expect | Rank | U | Peptide |
| --- | --- | --- | --- | --- | --- | --- | --- | --- | --- | --- | --- |

| Query | Dupes | Observed | Mr(expt) | Mr(calc) | ppm | M | Score | Expect | Rank | U | Peptide |
| --- | --- | --- | --- | --- | --- | --- | --- | --- | --- | --- | --- |
| 358 | 1 | 404.2295 | 806.4444 | 806.4439 | 0.55 | 0 | 34 | 0.024 | 1Score **> 32** indicates **identity** Score **> 30** indicates **homology** | U | R.TFWVVR.Q |

---

### 50

|  |  | Accession | Score | Description |
| --- | --- | --- | --- | --- |
|  | 1 | gi|154693514|gb|EDN93252.1| | 38 | hypothetical protein SS1G\_09118 [Sclerotinia sclerotiorum 1980 UF-70] |

|  |  | Score | Mass | Matches | Sequences | emPAI |  |
| --- | --- | --- | --- | --- | --- | --- | --- |
| 50.1 | gi|154693514|gb|EDN93252.1| | 38 | 33973 | 1 (1) | 1 (1) | 0.10 |  |
|  | hypothetical protein SS1G\_09118 [Sclerotinia sclerotiorum 1980 UF-70] | | | | | | |
|  | 2 samesets of gi|154693514|gb|EDN93252.1| | | | | | | |
|  | gi|156048772|ref|XP\_001590353.1| | 38 | 33973 | 1 (1) | 1 (1) | 0.10 |  |
|  | hypothetical protein SS1G\_09118 [Sclerotinia sclerotiorum 1980 UF-70] | | | | | | |
|  | gi|1095458486|gb|APA15437.1| | 38 | 33973 | 1 (1) | 1 (1) | 0.10 |  |
|  | hypothetical protein sscle\_14g102070 [Sclerotinia sclerotiorum 1980 UF-70] | | | | | | |

|  |  | Score | Mass | Matches | Sequences | emPAI |  |
| --- | --- | --- | --- | --- | --- | --- | --- |
| 50.1 | gi|154693514|gb|EDN93252.1| | 38 | 33973 | 1 (1) | 1 (1) | 0.10 | hypothetical protein SS1G\_09118 [Sclerotinia sclerotiorum 1980 UF-70] |
|  |  | | | | | | |
|  | 2 samesets of gi|154693514|gb|EDN93252.1| | | | | | | |
|  | gi|156048772|ref|XP\_001590353.1| | 38 | 33973 | 1 (1) | 1 (1) | 0.10 | hypothetical protein SS1G\_09118 [Sclerotinia sclerotiorum 1980 UF-70] |
|  |  | | | | | | |
|  | gi|1095458486|gb|APA15437.1| | 38 | 33973 | 1 (1) | 1 (1) | 0.10 | hypothetical protein sscle\_14g102070 [Sclerotinia sclerotiorum 1980 UF-70] |
|  |  | | | | | | |

#### 1 peptide matches (1 non-duplicate, 0 duplicate)

Auto-fit to window

| Query | Dupes | Observed | Mr(expt) | Mr(calc) | ppm | M | Score | Expect | Rank | U | Peptide |
| --- | --- | --- | --- | --- | --- | --- | --- | --- | --- | --- | --- |

| Query | Dupes | Observed | Mr(expt) | Mr(calc) | ppm | M | Score | Expect | Rank | U | Peptide |
| --- | --- | --- | --- | --- | --- | --- | --- | --- | --- | --- | --- |
| 1908 |  | 510.7601 | 1019.5056 | 1019.5036 | 2.02 | 0 | 38 | 0.0033 | 1Score **> 34** indicates **identity** Score **> 26** indicates **homology** | U | R.LSSNQAWSK.G |

---

### 51

|  |  | Accession | Score | Description |
| --- | --- | --- | --- | --- |
|  | 1 | gi|347830059|emb|CCD45756.1| | 38 | glycoside hydrolase family 28 protein [Botrytis cinerea T4] |

|  |  | Score | Mass | Matches | Sequences | emPAI |  |
| --- | --- | --- | --- | --- | --- | --- | --- |
| 51.1 | gi|347830059|emb|CCD45756.1| | 38 | 39845 | 2 (2) | 1 (1) | 0.08 |  |
|  | glycoside hydrolase family 28 protein [Botrytis cinerea T4] | | | | | | |

|  |  | Score | Mass | Matches | Sequences | emPAI |  |
| --- | --- | --- | --- | --- | --- | --- | --- |
| 51.1 | gi|347830059|emb|CCD45756.1| | 38 | 39845 | 2 (2) | 1 (1) | 0.08 | glycoside hydrolase family 28 protein [Botrytis cinerea T4] |
|  |  | | | | | | |

#### 2 peptide matches (1 non-duplicate, 1 duplicate)

Auto-fit to window

| Query | Dupes | Observed | Mr(expt) | Mr(calc) | ppm | M | Score | Expect | Rank | U | Peptide |
| --- | --- | --- | --- | --- | --- | --- | --- | --- | --- | --- | --- |

| Query | Dupes | Observed | Mr(expt) | Mr(calc) | ppm | M | Score | Expect | Rank | U | Peptide |
| --- | --- | --- | --- | --- | --- | --- | --- | --- | --- | --- | --- |
| 3344 | 1 | 580.7777 | 1159.5409 | 1159.5404 | 0.39 | 0 | 35 | 0.0024 | 1Score **> 32** indicates **identity** Score **> 21** indicates **homology** | U | R.TCHVSSLGSGR.D |

---

### 52

|  |  | Accession | Score | Description |
| --- | --- | --- | --- | --- |
|  | 1 | gi|347837081|emb|CCD51653.1| | 37 | similar to ubiquitin-protein ligase [Botrytis cinerea T4] |

|  |  | Score | Mass | Matches | Sequences | emPAI |  |
| --- | --- | --- | --- | --- | --- | --- | --- |
| 52.1 | gi|347837081|emb|CCD51653.1| | 37 | 134855 | 3 (1) | 3 (1) | 0.02 |  |
|  | similar to ubiquitin-protein ligase [Botrytis cinerea T4] | | | | | | |
|  | 3 samesets of gi|347837081|emb|CCD51653.1| | | | | | | |
|  | gi|154691227|gb|EDN90965.1| | 37 | 134295 | 2 (1) | 2 (1) | 0.02 |  |
|  | hypothetical protein SS1G\_00365 [Sclerotinia sclerotiorum 1980 UF-70] | | | | | | |
|  | gi|156064715|ref|XP\_001598279.1| | 37 | 134295 | 2 (1) | 2 (1) | 0.02 |  |
|  | hypothetical protein SS1G\_00365 [Sclerotinia sclerotiorum 1980 UF-70] | | | | | | |
|  | gi|1095451036|gb|APA07998.1| | 37 | 134827 | 2 (1) | 2 (1) | 0.02 |  |
|  | hypothetical protein sscle\_03g027680 [Sclerotinia sclerotiorum 1980 UF-70] | | | | | | |

|  |  | Score | Mass | Matches | Sequences | emPAI |  |
| --- | --- | --- | --- | --- | --- | --- | --- |
| 52.1 | gi|347837081|emb|CCD51653.1| | 37 | 134855 | 3 (1) | 3 (1) | 0.02 | similar to ubiquitin-protein ligase [Botrytis cinerea T4] |
|  |  | | | | | | |
|  | 3 samesets of gi|347837081|emb|CCD51653.1| | | | | | | |
|  | gi|154691227|gb|EDN90965.1| | 37 | 134295 | 2 (1) | 2 (1) | 0.02 | hypothetical protein SS1G\_00365 [Sclerotinia sclerotiorum 1980 UF-70] |
|  |  | | | | | | |
|  | gi|156064715|ref|XP\_001598279.1| | 37 | 134295 | 2 (1) | 2 (1) | 0.02 | hypothetical protein SS1G\_00365 [Sclerotinia sclerotiorum 1980 UF-70] |
|  |  | | | | | | |
|  | gi|1095451036|gb|APA07998.1| | 37 | 134827 | 2 (1) | 2 (1) | 0.02 | hypothetical protein sscle\_03g027680 [Sclerotinia sclerotiorum 1980 UF-70] |
|  |  | | | | | | |

#### 3 peptide matches (3 non-duplicate, 0 duplicate)

Auto-fit to window

| Query | Dupes | Observed | Mr(expt) | Mr(calc) | ppm | M | Score | Expect | Rank | U | Peptide |
| --- | --- | --- | --- | --- | --- | --- | --- | --- | --- | --- | --- |

| Query | Dupes | Observed | Mr(expt) | Mr(calc) | ppm | M | Score | Expect | Rank | U | Peptide |
| --- | --- | --- | --- | --- | --- | --- | --- | --- | --- | --- | --- |
| 264 |  | 393.2465 | 784.4784 | 784.4807 | -2.91 | 0 | 37 | 0.011 | 1Score **> 30** indicates **identity** | U | R.AALEILR.Y |
| 2851 |  | 557.8144 | 1113.6142 | 1113.6441 | -26.8 | 2 | 3 | 5.2 | 8Score **> 32** indicates **identity** Score **> 23** indicates **homology** | U | K.RKIMAAVGPR.I + Oxidation (M) |
| 4179 |  | 411.2203 | 1230.6392 | 1230.6357 | 2.87 | 1 | 13 | 0.55 | 1Score **> 34** indicates **identity** Score **> 23** indicates **homology** | U | R.IFPDRADEIR.M |

---

### 53

|  |  | Accession | Score | Description |
| --- | --- | --- | --- | --- |
|  | 1 | gi|154693707|gb|EDN93445.1| | 37 | hypothetical protein SS1G\_09311 [Sclerotinia sclerotiorum 1980 UF-70] |

|  |  | Score | Mass | Matches | Sequences | emPAI |  |
| --- | --- | --- | --- | --- | --- | --- | --- |
| 53.1 | gi|154693707|gb|EDN93445.1| | 37 | 78216 | 2 (1) | 2 (1) | 0.04 |  |
|  | hypothetical protein SS1G\_09311 [Sclerotinia sclerotiorum 1980 UF-70] | | | | | | |
|  | 2 samesets of gi|154693707|gb|EDN93445.1| | | | | | | |
|  | gi|156046070|ref|XP\_001589590.1| | 37 | 78216 | 2 (1) | 2 (1) | 0.04 |  |
|  | hypothetical protein SS1G\_09311 [Sclerotinia sclerotiorum 1980 UF-70] | | | | | | |
|  | gi|1095458871|gb|APA15821.1| | 37 | 82436 | 2 (1) | 2 (1) | 0.04 |  |
|  | hypothetical protein sscle\_15g105910 [Sclerotinia sclerotiorum 1980 UF-70] | | | | | | |

|  |  | Score | Mass | Matches | Sequences | emPAI |  |
| --- | --- | --- | --- | --- | --- | --- | --- |
| 53.1 | gi|154693707|gb|EDN93445.1| | 37 | 78216 | 2 (1) | 2 (1) | 0.04 | hypothetical protein SS1G\_09311 [Sclerotinia sclerotiorum 1980 UF-70] |
|  |  | | | | | | |
|  | 2 samesets of gi|154693707|gb|EDN93445.1| | | | | | | |
|  | gi|156046070|ref|XP\_001589590.1| | 37 | 78216 | 2 (1) | 2 (1) | 0.04 | hypothetical protein SS1G\_09311 [Sclerotinia sclerotiorum 1980 UF-70] |
|  |  | | | | | | |
|  | gi|1095458871|gb|APA15821.1| | 37 | 82436 | 2 (1) | 2 (1) | 0.04 | hypothetical protein sscle\_15g105910 [Sclerotinia sclerotiorum 1980 UF-70] |
|  |  | | | | | | |

#### 2 peptide matches (2 non-duplicate, 0 duplicate)

Auto-fit to window

| Query | Dupes | Observed | Mr(expt) | Mr(calc) | ppm | M | Score | Expect | Rank | U | Peptide |
| --- | --- | --- | --- | --- | --- | --- | --- | --- | --- | --- | --- |

| Query | Dupes | Observed | Mr(expt) | Mr(calc) | ppm | M | Score | Expect | Rank | U | Peptide |
| --- | --- | --- | --- | --- | --- | --- | --- | --- | --- | --- | --- |
| 1204 |  | 466.7430 | 931.4714 | 931.4835 | -13.0 | 2 | 37 | 0.03 | 1Score **> 34** indicates **identity** | U | R.SSSPRRDK.R |
| 4709 |  | 638.8137 | 1275.6129 | 1275.6306 | -13.9 | 1 | 7 | 0.81 | 1Score **> 34** indicates **identity** Score **> 18** indicates **homology** |  | R.DNTKNEELVSK.A |

---

### 54

|  |  | Accession | Score | Description |
| --- | --- | --- | --- | --- |
|  | 1 | gi|154691632|gb|EDN91370.1| | 36 | hypothetical protein SS1G\_00773 [Sclerotinia sclerotiorum 1980 UF-70] |

|  |  | Score | Mass | Matches | Sequences | emPAI |  |
| --- | --- | --- | --- | --- | --- | --- | --- |
| 54.1 | gi|154691632|gb|EDN91370.1| | 36 | 190114 | 6 (1) | 4 (1) | 0.02 |  |
|  | hypothetical protein SS1G\_00773 [Sclerotinia sclerotiorum 1980 UF-70] | | | | | | |
|  | 2 samesets of gi|154691632|gb|EDN91370.1| | | | | | | |
|  | gi|156065525|ref|XP\_001598684.1| | 36 | 190114 | 6 (1) | 4 (1) | 0.02 |  |
|  | hypothetical protein SS1G\_00773 [Sclerotinia sclerotiorum 1980 UF-70] | | | | | | |
|  | gi|1095450715|gb|APA07677.1| | 36 | 129514 | 5 (1) | 3 (1) | 0.03 |  |
|  | hypothetical protein sscle\_03g024470 [Sclerotinia sclerotiorum 1980 UF-70] | | | | | | |

|  |  | Score | Mass | Matches | Sequences | emPAI |  |
| --- | --- | --- | --- | --- | --- | --- | --- |
| 54.1 | gi|154691632|gb|EDN91370.1| | 36 | 190114 | 6 (1) | 4 (1) | 0.02 | hypothetical protein SS1G\_00773 [Sclerotinia sclerotiorum 1980 UF-70] |
|  |  | | | | | | |
|  | 2 samesets of gi|154691632|gb|EDN91370.1| | | | | | | |
|  | gi|156065525|ref|XP\_001598684.1| | 36 | 190114 | 6 (1) | 4 (1) | 0.02 | hypothetical protein SS1G\_00773 [Sclerotinia sclerotiorum 1980 UF-70] |
|  |  | | | | | | |
|  | gi|1095450715|gb|APA07677.1| | 36 | 129514 | 5 (1) | 3 (1) | 0.03 | hypothetical protein sscle\_03g024470 [Sclerotinia sclerotiorum 1980 UF-70] |
|  |  | | | | | | |

#### 6 peptide matches (5 non-duplicate, 1 duplicate)

Auto-fit to window

| Query | Dupes | Observed | Mr(expt) | Mr(calc) | ppm | M | Score | Expect | Rank | U | Peptide |
| --- | --- | --- | --- | --- | --- | --- | --- | --- | --- | --- | --- |

| Query | Dupes | Observed | Mr(expt) | Mr(calc) | ppm | M | Score | Expect | Rank | U | Peptide |
| --- | --- | --- | --- | --- | --- | --- | --- | --- | --- | --- | --- |
| 273 | 1 | 394.7351 | 787.4555 | 787.4552 | 0.46 | 0 | 8 | 1.9 | 4Score **> 33** indicates **identity** Score **> 23** indicates **homology** | U | K.LSALQTR.D |
| 387 |  | 408.2252 | 814.4358 | 814.4409 | -6.34 | 1 | 4 | 3.1 | 8Score **> 32** indicates **identity** Score **> 22** indicates **homology** |  | R.DQLRAGR.G |
| 481 |  | 416.7477 | 831.4809 | 831.4814 | -0.55 | 1 | 36 | 0.02 | 1Score **> 32** indicates **identity** | U | R.SISLEKR.A |
| 8383 |  | 837.9076 | 1673.8007 | 1673.8043 | -2.12 | 0 | 2 | 1 | 2Score **> 34** indicates **identity** Score **> 15** indicates **homology** | U | R.TQALSCPIGDDIQTR.M |
| 8405 |  | 838.4071 | 1674.7996 | 1674.7883 | 6.78 | 0 | 0 | 1 | 3Score **> 34** indicates **identity** Score **> 13** indicates **homology** | U | R.TQALSCPIGDDIQTR.M + Deamidated (NQ) |

---

### 55

|  |  | Accession | Score | Description |
| --- | --- | --- | --- | --- |
|  | 1 | gi|154703678|gb|EDO03417.1| | 36 | hypothetical protein SS1G\_05898 [Sclerotinia sclerotiorum 1980 UF-70] |

|  |  | Score | Mass | Matches | Sequences | emPAI |  |
| --- | --- | --- | --- | --- | --- | --- | --- |
| 55.1 | gi|154703678|gb|EDO03417.1| | 36 | 89774 | 4 (1) | 1 (1) | 0.04 |  |
|  | hypothetical protein SS1G\_05898 [Sclerotinia sclerotiorum 1980 UF-70] | | | | | | |
|  | 2 samesets of gi|154703678|gb|EDO03417.1| | | | | | | |
|  | gi|156054100|ref|XP\_001592976.1| | 36 | 89774 | 4 (1) | 1 (1) | 0.04 |  |
|  | hypothetical protein SS1G\_05898 [Sclerotinia sclerotiorum 1980 UF-70] | | | | | | |
|  | gi|1095452910|gb|APA09870.1| | 36 | 92511 | 4 (1) | 1 (1) | 0.04 |  |
|  | hypothetical protein sscle\_05g046400 [Sclerotinia sclerotiorum 1980 UF-70] | | | | | | |

|  |  | Score | Mass | Matches | Sequences | emPAI |  |
| --- | --- | --- | --- | --- | --- | --- | --- |
| 55.1 | gi|154703678|gb|EDO03417.1| | 36 | 89774 | 4 (1) | 1 (1) | 0.04 | hypothetical protein SS1G\_05898 [Sclerotinia sclerotiorum 1980 UF-70] |
|  |  | | | | | | |
|  | 2 samesets of gi|154703678|gb|EDO03417.1| | | | | | | |
|  | gi|156054100|ref|XP\_001592976.1| | 36 | 89774 | 4 (1) | 1 (1) | 0.04 | hypothetical protein SS1G\_05898 [Sclerotinia sclerotiorum 1980 UF-70] |
|  |  | | | | | | |
|  | gi|1095452910|gb|APA09870.1| | 36 | 92511 | 4 (1) | 1 (1) | 0.04 | hypothetical protein sscle\_05g046400 [Sclerotinia sclerotiorum 1980 UF-70] |
|  |  | | | | | | |

#### 4 peptide matches (1 non-duplicate, 3 duplicate)

Auto-fit to window

| Query | Dupes | Observed | Mr(expt) | Mr(calc) | ppm | M | Score | Expect | Rank | U | Peptide |
| --- | --- | --- | --- | --- | --- | --- | --- | --- | --- | --- | --- |

| Query | Dupes | Observed | Mr(expt) | Mr(calc) | ppm | M | Score | Expect | Rank | U | Peptide |
| --- | --- | --- | --- | --- | --- | --- | --- | --- | --- | --- | --- |
| 767 | 3 | 436.7364 | 871.4583 | 871.4837 | -29.1 | 0 | 36 | 0.016 | 1Score **> 31** indicates **identity** | U | K.QLAAMIPK.Y + Deamidated (NQ) |

---

### 56

|  |  | Accession | Score | Description |
| --- | --- | --- | --- | --- |
|  | 1 | gi|347830591|emb|CCD46288.1| | 35 | similar to calcium-transporting P-type ATPase [Botrytis cinerea T4] |

|  |  | Score | Mass | Matches | Sequences | emPAI |  |
| --- | --- | --- | --- | --- | --- | --- | --- |
| 56.1 | gi|347830591|emb|CCD46288.1| | 35 | 119114 | 8 (1) | 7 (1) | 0.03 |  |
|  | similar to calcium-transporting P-type ATPase [Botrytis cinerea T4] | | | | | | |

|  |  | Score | Mass | Matches | Sequences | emPAI |  |
| --- | --- | --- | --- | --- | --- | --- | --- |
| 56.1 | gi|347830591|emb|CCD46288.1| | 35 | 119114 | 8 (1) | 7 (1) | 0.03 | similar to calcium-transporting P-type ATPase [Botrytis cinerea T4] |
|  |  | | | | | | |

#### 8 peptide matches (7 non-duplicate, 1 duplicate)

Auto-fit to window

| Query | Dupes | Observed | Mr(expt) | Mr(calc) | ppm | M | Score | Expect | Rank | U | Peptide |
| --- | --- | --- | --- | --- | --- | --- | --- | --- | --- | --- | --- |

| Query | Dupes | Observed | Mr(expt) | Mr(calc) | ppm | M | Score | Expect | Rank | U | Peptide |
| --- | --- | --- | --- | --- | --- | --- | --- | --- | --- | --- | --- |
| 584 |  | 424.2479 | 846.4813 | 846.4923 | -13.0 | 1 | 2 | 1.9 | 5Score **> 32** indicates **identity** Score **> 17** indicates **homology** | U | R.TGVSKSIR.R |
| 1214 |  | 467.2355 | 932.4564 | 932.4788 | -24.0 | 2 | 35 | 0.041 | 1Score **> 34** indicates **identity** | U | R.DRANSKSR.H |
| 1286 |  | 473.2217 | 944.4288 | 944.4563 | -29.1 | 0 | 2 | 4.4 | 8Score **> 29** indicates **identity** Score **> 21** indicates **homology** |  | R.IGNIANNAR.L + 3 Deamidated (NQ) |
| 2335 |  | 534.8060 | 1067.5974 | 1067.5658 | 29.6 | 1 | 3 | 1.8 | 9Score **> 29** indicates **identity** Score **> 18** indicates **homology** |  | R.MARHNAIVR.K + Deamidated (NQ) |
| 2997 |  | 564.3187 | 1126.6229 | 1126.6168 | 5.39 | 1 | 6 | 1.3 | 3Score **> 31** indicates **identity** Score **> 19** indicates **homology** | U | K.KLGMPIAEPR.A + Oxidation (M) |
| 5275 |  | 664.3519 | 1326.6892 | 1326.7255 | -27.4 | 1 | 1 | 2.4 | 3Score **> 33** indicates **identity** Score **> 18** indicates **homology** |  | R.ILRIGNIANNAR.L + 3 Deamidated (NQ) |
| 21688 | 1 | 930.1608 | 3716.6140 | 3716.7046 | -24.4 | 1 | 2 | 1 | 1Score **> 33** indicates **identity** Score **> 15** indicates **homology** | U | R.TPAWPPSVRDRPEAEALMAGSGSDADSSMAEAVSQK.T + Oxidation (M) |

---

### 57

|  |  | Accession | Score | Description |
| --- | --- | --- | --- | --- |
|  | 1 | gi|347836319|emb|CCD50891.1| | 35 | glycoside hydrolase family 3 protein [Botrytis cinerea T4] |

|  |  | Score | Mass | Matches | Sequences | emPAI |  |
| --- | --- | --- | --- | --- | --- | --- | --- |
| 57.1 | gi|347836319|emb|CCD50891.1| | 35 | 95468 | 3 (1) | 2 (1) | 0.03 |  |
|  | glycoside hydrolase family 3 protein [Botrytis cinerea T4] | | | | | | |
|  | 3 samesets of gi|347836319|emb|CCD50891.1| | | | | | | |
|  | gi|154704924|gb|EDO04663.1| | 35 | 96368 | 2 (1) | 2 (1) | 0.03 |  |
|  | beta-glucosidase 1 precursor [Sclerotinia sclerotiorum 1980 UF-70] | | | | | | |
|  | gi|156051478|ref|XP\_001591700.1| | 35 | 96368 | 2 (1) | 2 (1) | 0.03 |  |
|  | beta-glucosidase 1 precursor [Sclerotinia sclerotiorum 1980 UF-70] | | | | | | |
|  | gi|1095453421|gb|APA10380.1| | 35 | 96368 | 2 (1) | 2 (1) | 0.03 |  |
|  | hypothetical protein sscle\_06g051500 [Sclerotinia sclerotiorum 1980 UF-70] | | | | | | |

|  |  | Score | Mass | Matches | Sequences | emPAI |  |
| --- | --- | --- | --- | --- | --- | --- | --- |
| 57.1 | gi|347836319|emb|CCD50891.1| | 35 | 95468 | 3 (1) | 2 (1) | 0.03 | glycoside hydrolase family 3 protein [Botrytis cinerea T4] |
|  |  | | | | | | |
|  | 3 samesets of gi|347836319|emb|CCD50891.1| | | | | | | |
|  | gi|154704924|gb|EDO04663.1| | 35 | 96368 | 2 (1) | 2 (1) | 0.03 | beta-glucosidase 1 precursor [Sclerotinia sclerotiorum 1980 UF-70] |
|  |  | | | | | | |
|  | gi|156051478|ref|XP\_001591700.1| | 35 | 96368 | 2 (1) | 2 (1) | 0.03 | beta-glucosidase 1 precursor [Sclerotinia sclerotiorum 1980 UF-70] |
|  |  | | | | | | |
|  | gi|1095453421|gb|APA10380.1| | 35 | 96368 | 2 (1) | 2 (1) | 0.03 | hypothetical protein sscle\_06g051500 [Sclerotinia sclerotiorum 1980 UF-70] |
|  |  | | | | | | |

#### 3 peptide matches (3 non-duplicate, 0 duplicate)

Auto-fit to window

| Query | Dupes | Observed | Mr(expt) | Mr(calc) | ppm | M | Score | Expect | Rank | U | Peptide |
| --- | --- | --- | --- | --- | --- | --- | --- | --- | --- | --- | --- |

| Query | Dupes | Observed | Mr(expt) | Mr(calc) | ppm | M | Score | Expect | Rank | U | Peptide |
| --- | --- | --- | --- | --- | --- | --- | --- | --- | --- | --- | --- |
| 2280 |  | 531.7726 | 1061.5306 | 1061.5294 | 1.09 | 0 | 35 | 0.0043 | 1Score **> 34** indicates **identity** Score **> 24** indicates **homology** | U | R.TPFTWGPTR.E |
| 4946 |  | 648.8666 | 1295.7186 | 1295.7197 | -0.85 | 0 | 21 | 0.074 | 1Score **> 30** indicates **identity** Score **> 22** indicates **homology** | U | K.NTNNALPLSKPK.F |
| 4954 |  | 649.3519 | 1296.6893 | 1296.7037 | -11.1 | 0 | 5 | 1 | 1Score **> 32** indicates **identity** Score **> 17** indicates **homology** | U | K.NTNNALPLSKPK.F + Deamidated (NQ) |

---

### 58

|  |  | Accession | Score | Description |
| --- | --- | --- | --- | --- |
|  | 1 | gi|154703202|gb|EDO02941.1| | 34 | hypothetical protein SS1G\_05418 [Sclerotinia sclerotiorum 1980 UF-70] |

|  |  | Score | Mass | Matches | Sequences | emPAI |  |
| --- | --- | --- | --- | --- | --- | --- | --- |
| 58.1 | gi|154703202|gb|EDO02941.1| | 34 | 10468 | 2 (1) | 1 (1) | 0.33 |  |
|  | hypothetical protein SS1G\_05418 [Sclerotinia sclerotiorum 1980 UF-70] | | | | | | |
|  | 3 samesets of gi|154703202|gb|EDO02941.1| | | | | | | |
|  | gi|156056132|ref|XP\_001593990.1| | 34 | 10468 | 2 (1) | 1 (1) | 0.33 |  |
|  | hypothetical protein SS1G\_05418 [Sclerotinia sclerotiorum 1980 UF-70] | | | | | | |
|  | gi|347828992|emb|CCD44689.1| | 34 | 10438 | 2 (1) | 1 (1) | 0.33 |  |
|  | similar to NADH-ubiquinone oxidoreductase subunit [Botrytis cinerea T4] | | | | | | |
|  | gi|1095454933|gb|APA11890.1| | 34 | 10468 | 2 (1) | 1 (1) | 0.33 |  |
|  | hypothetical protein sscle\_08g066600 [Sclerotinia sclerotiorum 1980 UF-70] | | | | | | |

|  |  | Score | Mass | Matches | Sequences | emPAI |  |
| --- | --- | --- | --- | --- | --- | --- | --- |
| 58.1 | gi|154703202|gb|EDO02941.1| | 34 | 10468 | 2 (1) | 1 (1) | 0.33 | hypothetical protein SS1G\_05418 [Sclerotinia sclerotiorum 1980 UF-70] |
|  |  | | | | | | |
|  | 3 samesets of gi|154703202|gb|EDO02941.1| | | | | | | |
|  | gi|156056132|ref|XP\_001593990.1| | 34 | 10468 | 2 (1) | 1 (1) | 0.33 | hypothetical protein SS1G\_05418 [Sclerotinia sclerotiorum 1980 UF-70] |
|  |  | | | | | | |
|  | gi|347828992|emb|CCD44689.1| | 34 | 10438 | 2 (1) | 1 (1) | 0.33 | similar to NADH-ubiquinone oxidoreductase subunit [Botrytis cinerea T4] |
|  |  | | | | | | |
|  | gi|1095454933|gb|APA11890.1| | 34 | 10468 | 2 (1) | 1 (1) | 0.33 | hypothetical protein sscle\_08g066600 [Sclerotinia sclerotiorum 1980 UF-70] |
|  |  | | | | | | |

#### 2 peptide matches (1 non-duplicate, 1 duplicate)

Auto-fit to window

| Query | Dupes | Observed | Mr(expt) | Mr(calc) | ppm | M | Score | Expect | Rank | U | Peptide |
| --- | --- | --- | --- | --- | --- | --- | --- | --- | --- | --- | --- |

| Query | Dupes | Observed | Mr(expt) | Mr(calc) | ppm | M | Score | Expect | Rank | U | Peptide |
| --- | --- | --- | --- | --- | --- | --- | --- | --- | --- | --- | --- |
| 4575 | 1 | 631.8217 | 1261.6288 | 1261.6038 | 19.8 | 0 | 34 | 0.0096 | 1Score **> 34** indicates **identity** Score **> 27** indicates **homology** | U | R.EVSEDLAGLSDK.E |

---

### 59

|  |  | Accession | Score | Description |
| --- | --- | --- | --- | --- |
|  | 1 | gi|154693400|gb|EDN93138.1| | 33 | predicted protein [Sclerotinia sclerotiorum 1980 UF-70] |

|  |  | Score | Mass | Matches | Sequences | emPAI |  |
| --- | --- | --- | --- | --- | --- | --- | --- |
| 59.1 | gi|154693400|gb|EDN93138.1| | 33 | 7259 | 3 (1) | 1 (1) | 0.49 |  |
|  | predicted protein [Sclerotinia sclerotiorum 1980 UF-70] | | | | | | |
|  | 1 sameset of gi|154693400|gb|EDN93138.1| | | | | | | |
|  | gi|156048544|ref|XP\_001590239.1| | 33 | 7259 | 3 (1) | 1 (1) | 0.49 |  |
|  | predicted protein [Sclerotinia sclerotiorum 1980 UF-70] | | | | | | |

|  |  | Score | Mass | Matches | Sequences | emPAI |  |
| --- | --- | --- | --- | --- | --- | --- | --- |
| 59.1 | gi|154693400|gb|EDN93138.1| | 33 | 7259 | 3 (1) | 1 (1) | 0.49 | predicted protein [Sclerotinia sclerotiorum 1980 UF-70] |
|  |  | | | | | | |
|  | 1 sameset of gi|154693400|gb|EDN93138.1| | | | | | | |
|  | gi|156048544|ref|XP\_001590239.1| | 33 | 7259 | 3 (1) | 1 (1) | 0.49 | predicted protein [Sclerotinia sclerotiorum 1980 UF-70] |
|  |  | | | | | | |

#### 3 peptide matches (1 non-duplicate, 2 duplicate)

Auto-fit to window

| Query | Dupes | Observed | Mr(expt) | Mr(calc) | ppm | M | Score | Expect | Rank | U | Peptide |
| --- | --- | --- | --- | --- | --- | --- | --- | --- | --- | --- | --- |

| Query | Dupes | Observed | Mr(expt) | Mr(calc) | ppm | M | Score | Expect | Rank | U | Peptide |
| --- | --- | --- | --- | --- | --- | --- | --- | --- | --- | --- | --- |
| 2579 | 2 | 546.7621 | 1091.5096 | 1091.5281 | -16.9 | 0 | 33 | 0.056 | 1Score **> 33** indicates **identity** | U | M.TSSLEMGGLR.S + Acetyl (Protein N-term) |

---

### 60

|  |  | Accession | Score | Description |
| --- | --- | --- | --- | --- |
|  | 1 | gi|347826976|emb|CCD42673.1| | 32 | hypothetical protein BofuT4\_P074750.1 [Botrytis cinerea T4] |

|  |  | Score | Mass | Matches | Sequences | emPAI |  |
| --- | --- | --- | --- | --- | --- | --- | --- |
| 60.1 | gi|347826976|emb|CCD42673.1| | 32 | 49814 | 3 (1) | 2 (1) | 0.07 |  |
|  | hypothetical protein BofuT4\_P074750.1 [Botrytis cinerea T4] | | | | | | |

|  |  | Score | Mass | Matches | Sequences | emPAI |  |
| --- | --- | --- | --- | --- | --- | --- | --- |
| 60.1 | gi|347826976|emb|CCD42673.1| | 32 | 49814 | 3 (1) | 2 (1) | 0.07 | hypothetical protein BofuT4\_P074750.1 [Botrytis cinerea T4] |
|  |  | | | | | | |

#### 3 peptide matches (2 non-duplicate, 1 duplicate)

Auto-fit to window

| Query | Dupes | Observed | Mr(expt) | Mr(calc) | ppm | M | Score | Expect | Rank | U | Peptide |
| --- | --- | --- | --- | --- | --- | --- | --- | --- | --- | --- | --- |

| Query | Dupes | Observed | Mr(expt) | Mr(calc) | ppm | M | Score | Expect | Rank | U | Peptide |
| --- | --- | --- | --- | --- | --- | --- | --- | --- | --- | --- | --- |
| 368 | 1 | 405.2237 | 808.4329 | 808.4443 | -14.1 | 0 | 32 | 0.03 | 1Score **> 30** indicates **identity** | U | R.LASLYSR.E |
| 4916 |  | 431.5726 | 1291.6961 | 1291.6958 | 0.20 | 1 | 5 | 5.9 | 5Score **> 32** indicates **identity** Score **> 25** indicates **homology** | U | K.LLKWMQTAIR.I + Deamidated (NQ); Dioxidation (W) |

---

### 61

|  |  | Accession | Score | Description |
| --- | --- | --- | --- | --- |
|  | 1 | gi|347842042|emb|CCD56614.1| | 32 | similar to MFS multidrug transporter [Botrytis cinerea T4] |

|  |  | Score | Mass | Matches | Sequences | emPAI |  |
| --- | --- | --- | --- | --- | --- | --- | --- |
| 61.1 | gi|347842042|emb|CCD56614.1| | 32 | 61143 | 2 (1) | 2 (1) | 0.05 |  |
|  | similar to MFS multidrug transporter [Botrytis cinerea T4] | | | | | | |

|  |  | Score | Mass | Matches | Sequences | emPAI |  |
| --- | --- | --- | --- | --- | --- | --- | --- |
| 61.1 | gi|347842042|emb|CCD56614.1| | 32 | 61143 | 2 (1) | 2 (1) | 0.05 | similar to MFS multidrug transporter [Botrytis cinerea T4] |
|  |  | | | | | | |

#### 2 peptide matches (2 non-duplicate, 0 duplicate)

Auto-fit to window

| Query | Dupes | Observed | Mr(expt) | Mr(calc) | ppm | M | Score | Expect | Rank | U | Peptide |
| --- | --- | --- | --- | --- | --- | --- | --- | --- | --- | --- | --- |

| Query | Dupes | Observed | Mr(expt) | Mr(calc) | ppm | M | Score | Expect | Rank | U | Peptide |
| --- | --- | --- | --- | --- | --- | --- | --- | --- | --- | --- | --- |
| 5 |  | 351.2005 | 700.3864 | 700.3868 | -0.56 | 0 | 32 | 0.048 | 1Score **> 32** indicates **identity** | U | R.IGDIAGR.K |
| 2518 |  | 543.7873 | 1085.5601 | 1085.5717 | -10.6 | 1 | 8 | 11 | 5Score **> 32** indicates **identity** Score **> 31** indicates **homology** | U | K.KNPVTIEQR.F + 2 Deamidated (NQ) |

---

### 62

|  |  | Accession | Score | Description |
| --- | --- | --- | --- | --- |
|  | 1 | gi|154692782|gb|EDN92520.1| | 32 | hypothetical protein SS1G\_08383 [Sclerotinia sclerotiorum 1980 UF-70] |

|  |  | Score | Mass | Matches | Sequences | emPAI |  |
| --- | --- | --- | --- | --- | --- | --- | --- |
| 62.1 | gi|154692782|gb|EDN92520.1| | 32 | 44011 | 7 (1) | 4 (1) | 0.08 |  |
|  | hypothetical protein SS1G\_08383 [Sclerotinia sclerotiorum 1980 UF-70] | | | | | | |
|  | 2 samesets of gi|154692782|gb|EDN92520.1| | | | | | | |
|  | gi|156049353|ref|XP\_001590643.1| | 32 | 44011 | 7 (1) | 4 (1) | 0.08 |  |
|  | hypothetical protein SS1G\_08383 [Sclerotinia sclerotiorum 1980 UF-70] | | | | | | |
|  | gi|1095455954|gb|APA12909.1| | 32 | 44011 | 7 (1) | 4 (1) | 0.08 |  |
|  | hypothetical protein sscle\_10g076790 [Sclerotinia sclerotiorum 1980 UF-70] | | | | | | |

|  |  | Score | Mass | Matches | Sequences | emPAI |  |
| --- | --- | --- | --- | --- | --- | --- | --- |
| 62.1 | gi|154692782|gb|EDN92520.1| | 32 | 44011 | 7 (1) | 4 (1) | 0.08 | hypothetical protein SS1G\_08383 [Sclerotinia sclerotiorum 1980 UF-70] |
|  |  | | | | | | |
|  | 2 samesets of gi|154692782|gb|EDN92520.1| | | | | | | |
|  | gi|156049353|ref|XP\_001590643.1| | 32 | 44011 | 7 (1) | 4 (1) | 0.08 | hypothetical protein SS1G\_08383 [Sclerotinia sclerotiorum 1980 UF-70] |
|  |  | | | | | | |
|  | gi|1095455954|gb|APA12909.1| | 32 | 44011 | 7 (1) | 4 (1) | 0.08 | hypothetical protein sscle\_10g076790 [Sclerotinia sclerotiorum 1980 UF-70] |
|  |  | | | | | | |

#### 7 peptide matches (4 non-duplicate, 3 duplicate)

Auto-fit to window

| Query | Dupes | Observed | Mr(expt) | Mr(calc) | ppm | M | Score | Expect | Rank | U | Peptide |
| --- | --- | --- | --- | --- | --- | --- | --- | --- | --- | --- | --- |

| Query | Dupes | Observed | Mr(expt) | Mr(calc) | ppm | M | Score | Expect | Rank | U | Peptide |
| --- | --- | --- | --- | --- | --- | --- | --- | --- | --- | --- | --- |
| 199 | 1 | 388.2313 | 774.4481 | 774.4487 | -0.79 | 0 | 20 | 0.83 | 1Score **> 32** indicates **identity** | U | K.TALLTEK.T |
| 345 | 2 | 403.2263 | 804.4380 | 804.4382 | -0.16 | 0 | 32 | 0.0064 | 1Score **> 34** indicates **identity** Score **> 23** indicates **homology** | U | R.VFSIDPK.T |
| 1520 |  | 486.7953 | 971.5760 | 971.5876 | -11.9 | 1 | 13 | 2 | 3Score **> 32** indicates **identity** Score **> 28** indicates **homology** | U | R.SARVAIVTR.D |
| 12218 |  | 1083.0438 | 2164.0731 | 2164.1092 | -16.7 | 2 | 0 | 1.3 | 3Score **> 35** indicates **identity** Score **> 14** indicates **homology** | U | R.QMSMNKAGTLVAVALQRSAR.V + Deamidated (NQ); 2 Oxidation (M) |

---

### 63

|  |  | Accession | Score | Description |
| --- | --- | --- | --- | --- |
|  | 1 | gi|347828896|emb|CCD44593.1| | 32 | hypothetical protein BofuT4\_P054940.1 [Botrytis cinerea T4] |

|  |  | Score | Mass | Matches | Sequences | emPAI |  |
| --- | --- | --- | --- | --- | --- | --- | --- |
| 63.1 | gi|347828896|emb|CCD44593.1| | 32 | 33119 | 2 (1) | 2 (1) | 0.10 |  |
|  | hypothetical protein BofuT4\_P054940.1 [Botrytis cinerea T4] | | | | | | |

|  |  | Score | Mass | Matches | Sequences | emPAI |  |
| --- | --- | --- | --- | --- | --- | --- | --- |
| 63.1 | gi|347828896|emb|CCD44593.1| | 32 | 33119 | 2 (1) | 2 (1) | 0.10 | hypothetical protein BofuT4\_P054940.1 [Botrytis cinerea T4] |
|  |  | | | | | | |

#### 2 peptide matches (2 non-duplicate, 0 duplicate)

Auto-fit to window

| Query | Dupes | Observed | Mr(expt) | Mr(calc) | ppm | M | Score | Expect | Rank | U | Peptide |
| --- | --- | --- | --- | --- | --- | --- | --- | --- | --- | --- | --- |

| Query | Dupes | Observed | Mr(expt) | Mr(calc) | ppm | M | Score | Expect | Rank | U | Peptide |
| --- | --- | --- | --- | --- | --- | --- | --- | --- | --- | --- | --- |
| 831 |  | 441.7743 | 881.5341 | 881.5447 | -12.0 | 0 | 32 | 0.013 | 1Score **> 25** indicates **identity** | U | R.IIEVRPR.T |
| 22892 |  | 1024.2438 | 4092.9460 | 4092.9336 | 3.02 | 1 | 2 | 1 | 4Score **> 35** indicates **identity** Score **> 15** indicates **homology** | U | R.YFDDIWGASESCSLNMNSALLEFKNLDELVIVVER.T + Deamidated (NQ); Oxidation (M) |

---

### 64

|  |  | Accession | Score | Description |
| --- | --- | --- | --- | --- |
|  | 1 | gi|347835016|emb|CCD49588.1| | 32 | similar to leucine rich repeat domain-containing protein [Botrytis cinerea T4] |

|  |  | Score | Mass | Matches | Sequences | emPAI |  |
| --- | --- | --- | --- | --- | --- | --- | --- |
| 64.1 | gi|347835016|emb|CCD49588.1| | 32 | 93774 | 39 (1) | 4 (1) | 0.03 |  |
|  | similar to leucine rich repeat domain-containing protein [Botrytis cinerea T4] | | | | | | |

|  |  | Score | Mass | Matches | Sequences | emPAI |  |
| --- | --- | --- | --- | --- | --- | --- | --- |
| 64.1 | gi|347835016|emb|CCD49588.1| | 32 | 93774 | 39 (1) | 4 (1) | 0.03 | similar to leucine rich repeat domain-containing protein [Botrytis cinerea T4] |
|  |  | | | | | | |

#### 39 peptide matches (5 non-duplicate, 34 duplicate)

Auto-fit to window

| Query | Dupes | Observed | Mr(expt) | Mr(calc) | ppm | M | Score | Expect | Rank | U | Peptide |
| --- | --- | --- | --- | --- | --- | --- | --- | --- | --- | --- | --- |

| Query | Dupes | Observed | Mr(expt) | Mr(calc) | ppm | M | Score | Expect | Rank | U | Peptide |
| --- | --- | --- | --- | --- | --- | --- | --- | --- | --- | --- | --- |
| 972 | 33 | 450.2731 | 898.5316 | 898.5236 | 8.88 | 0 | 32 | 0.031 | 1Score **> 29** indicates **identity** | U | R.INTSVLPR.L |
| 978 | 1 | 450.7609 | 899.5073 | 899.5076 | -0.34 | 0 | 23 | 0.1 | 2Score **> 32** indicates **identity** Score **> 26** indicates **homology** | U | R.INTSVLPR.L + Deamidated (NQ) |
| 1329 |  | 476.2301 | 950.4456 | 950.4682 | -23.8 | 0 | 3 | 2.4 | 5Score **> 32** indicates **identity** Score **> 19** indicates **homology** |  | R.TGSSHGHLR.G |
| 6512 |  | 729.8805 | 1457.7465 | 1457.7725 | -17.8 | 0 | 1 | 3.5 | 8Score **> 34** indicates **identity** Score **> 19** indicates **homology** |  | K.NISALEISDIDIR.Q |
| 8067 |  | 546.9432 | 1637.8077 | 1637.8202 | -7.61 | 1 | 6 | 2 | 5Score **> 34** indicates **identity** Score **> 21** indicates **homology** | U | R.QFFGWDKLAEQLR.S + Deamidated (NQ) |

---

### 65

|  |  | Accession | Score | Description |
| --- | --- | --- | --- | --- |
|  | 1 | gi|154705300|gb|EDO05039.1| | 31 | hypothetical protein SS1G\_07524 [Sclerotinia sclerotiorum 1980 UF-70] |

|  |  | Score | Mass | Matches | Sequences | emPAI |  |
| --- | --- | --- | --- | --- | --- | --- | --- |
| 65.1 | gi|154705300|gb|EDO05039.1| | 31 | 34691 | 4 (1) | 3 (1) | 0.10 |  |
|  | hypothetical protein SS1G\_07524 [Sclerotinia sclerotiorum 1980 UF-70] | | | | | | |
|  | 3 samesets of gi|154705300|gb|EDO05039.1| | | | | | | |
|  | gi|156052230|ref|XP\_001592076.1| | 31 | 34691 | 4 (1) | 3 (1) | 0.10 |  |
|  | hypothetical protein SS1G\_07524 [Sclerotinia sclerotiorum 1980 UF-70] | | | | | | |
|  | gi|347839725|emb|CCD54297.1| | 31 | 34662 | 4 (1) | 3 (1) | 0.10 |  |
|  | similar to succinyl-CoA ligase subunit alpha [Botrytis cinerea T4] | | | | | | |
|  | gi|1095453137|gb|APA10096.1| | 31 | 34691 | 4 (1) | 3 (1) | 0.10 |  |
|  | hypothetical protein sscle\_06g048660 [Sclerotinia sclerotiorum 1980 UF-70] | | | | | | |

|  |  | Score | Mass | Matches | Sequences | emPAI |  |
| --- | --- | --- | --- | --- | --- | --- | --- |
| 65.1 | gi|154705300|gb|EDO05039.1| | 31 | 34691 | 4 (1) | 3 (1) | 0.10 | hypothetical protein SS1G\_07524 [Sclerotinia sclerotiorum 1980 UF-70] |
|  |  | | | | | | |
|  | 3 samesets of gi|154705300|gb|EDO05039.1| | | | | | | |
|  | gi|156052230|ref|XP\_001592076.1| | 31 | 34691 | 4 (1) | 3 (1) | 0.10 | hypothetical protein SS1G\_07524 [Sclerotinia sclerotiorum 1980 UF-70] |
|  |  | | | | | | |
|  | gi|347839725|emb|CCD54297.1| | 31 | 34662 | 4 (1) | 3 (1) | 0.10 | similar to succinyl-CoA ligase subunit alpha [Botrytis cinerea T4] |
|  |  | | | | | | |
|  | gi|1095453137|gb|APA10096.1| | 31 | 34691 | 4 (1) | 3 (1) | 0.10 | hypothetical protein sscle\_06g048660 [Sclerotinia sclerotiorum 1980 UF-70] |
|  |  | | | | | | |

#### 4 peptide matches (3 non-duplicate, 1 duplicate)

Auto-fit to window

| Query | Dupes | Observed | Mr(expt) | Mr(calc) | ppm | M | Score | Expect | Rank | U | Peptide |
| --- | --- | --- | --- | --- | --- | --- | --- | --- | --- | --- | --- |

| Query | Dupes | Observed | Mr(expt) | Mr(calc) | ppm | M | Score | Expect | Rank | U | Peptide |
| --- | --- | --- | --- | --- | --- | --- | --- | --- | --- | --- | --- |
| 3009 | 1 | 564.8259 | 1127.6372 | 1127.6161 | 18.7 | 0 | 31 | 0.031 | 1Score **> 32** indicates **identity** Score **> 29** indicates **homology** | U | K.IGIMPGFIHK.R + Oxidation (M) |
| 4312 |  | 620.8414 | 1239.6683 | 1239.6506 | 14.3 | 1 | 2 | 1.2 | 3Score **> 31** indicates **identity** Score **> 15** indicates **homology** | U | R.RMGHAGAIVSGGK.G |
| 9842 |  | 926.9145 | 1851.8144 | 1851.8654 | -27.5 | 1 | 0 | 4.5 | 4Score **> 32** indicates **identity** Score **> 19** indicates **homology** | U | M.QAFSRCARPAMQAAMR.R + Deamidated (NQ) |

---

### 66

|  |  | Accession | Score | Description |
| --- | --- | --- | --- | --- |
|  | 1 | gi|347836616|emb|CCD51188.1| | 31 | similar to plasma membrane ATPase [Botrytis cinerea T4] |

|  |  | Score | Mass | Matches | Sequences | emPAI |  |
| --- | --- | --- | --- | --- | --- | --- | --- |
| 66.1 | gi|347836616|emb|CCD51188.1| | 31 | 96613 | 3 (1) | 2 (1) | 0.03 |  |
|  | similar to plasma membrane ATPase [Botrytis cinerea T4] | | | | | | |

|  |  | Score | Mass | Matches | Sequences | emPAI |  |
| --- | --- | --- | --- | --- | --- | --- | --- |
| 66.1 | gi|347836616|emb|CCD51188.1| | 31 | 96613 | 3 (1) | 2 (1) | 0.03 | similar to plasma membrane ATPase [Botrytis cinerea T4] |
|  |  | | | | | | |

#### 3 peptide matches (2 non-duplicate, 1 duplicate)

Auto-fit to window

| Query | Dupes | Observed | Mr(expt) | Mr(calc) | ppm | M | Score | Expect | Rank | U | Peptide |
| --- | --- | --- | --- | --- | --- | --- | --- | --- | --- | --- | --- |

| Query | Dupes | Observed | Mr(expt) | Mr(calc) | ppm | M | Score | Expect | Rank | U | Peptide |
| --- | --- | --- | --- | --- | --- | --- | --- | --- | --- | --- | --- |
| 4588 |  | 632.3142 | 1262.6137 | 1262.6506 | -29.2 | 0 | 6 | 1.2 | 3Score **> 34** indicates **identity** Score **> 19** indicates **homology** |  | K.YNVVEILQQR.G + 2 Deamidated (NQ) |
| 6606 | 1 | 737.4014 | 1472.7882 | 1472.7947 | -4.41 | 2 | 31 | 0.02 | 1Score **> 33** indicates **identity** Score **> 26** indicates **homology** | U | R.IGLTDSEVQARRK.K + Deamidated (NQ) |

---

### 67

|  |  | Accession | Score | Description |
| --- | --- | --- | --- | --- |
|  | 1 | gi|347839226|emb|CCD53798.1| | 30 | similar to copper radical oxidase [Botrytis cinerea T4] |

|  |  | Score | Mass | Matches | Sequences | emPAI |  |
| --- | --- | --- | --- | --- | --- | --- | --- |
| 67.1 | gi|347839226|emb|CCD53798.1| | 30 | 115437 | 4 (1) | 3 (1) | 0.03 |  |
|  | similar to copper radical oxidase [Botrytis cinerea T4] | | | | | | |

|  |  | Score | Mass | Matches | Sequences | emPAI |  |
| --- | --- | --- | --- | --- | --- | --- | --- |
| 67.1 | gi|347839226|emb|CCD53798.1| | 30 | 115437 | 4 (1) | 3 (1) | 0.03 | similar to copper radical oxidase [Botrytis cinerea T4] |
|  |  | | | | | | |

#### 4 peptide matches (3 non-duplicate, 1 duplicate)

Auto-fit to window

| Query | Dupes | Observed | Mr(expt) | Mr(calc) | ppm | M | Score | Expect | Rank | U | Peptide |
| --- | --- | --- | --- | --- | --- | --- | --- | --- | --- | --- | --- |

| Query | Dupes | Observed | Mr(expt) | Mr(calc) | ppm | M | Score | Expect | Rank | U | Peptide |
| --- | --- | --- | --- | --- | --- | --- | --- | --- | --- | --- | --- |
| 92 | 1 | 368.7155 | 735.4164 | 735.4167 | -0.36 | 0 | 30 | 0.038 | 1Score **> 29** indicates **identity** | U | K.ISFLEK.F |
| 2916 |  | 560.7664 | 1119.5183 | 1119.4979 | 18.2 | 0 | 17 | 0.056 | 1Score **> 33** indicates **identity** Score **> 17** indicates **homology** | U | K.GCLTDDAVNR.V |
| 17470 |  | 984.5258 | 2950.5556 | 2950.5764 | -7.02 | 1 | 0 | 1 | 1Score **> 32** indicates **identity** Score **> 13** indicates **homology** | U | R.ILDEVTLQTQRVLPNIPAAVNNFLGGR.T + 3 Deamidated (NQ) |

---

### 68

|  |  | Accession | Score | Description |
| --- | --- | --- | --- | --- |
|  | 1 | gi|154693787|gb|EDN93525.1| | 30 | hypothetical protein SS1G\_09392 [Sclerotinia sclerotiorum 1980 UF-70] |

|  |  | Score | Mass | Matches | Sequences | emPAI |  |
| --- | --- | --- | --- | --- | --- | --- | --- |
| 68.1 | gi|154693787|gb|EDN93525.1| | 30 | 42557 | 4 (1) | 2 (1) | 0.08 |  |
|  | hypothetical protein SS1G\_09392 [Sclerotinia sclerotiorum 1980 UF-70] | | | | | | |
|  | 2 samesets of gi|154693787|gb|EDN93525.1| | | | | | | |
|  | gi|156046276|ref|XP\_001589670.1| | 30 | 42557 | 4 (1) | 2 (1) | 0.08 |  |
|  | hypothetical protein SS1G\_09392 [Sclerotinia sclerotiorum 1980 UF-70] | | | | | | |
|  | gi|1095458818|gb|APA15768.1| | 30 | 42557 | 4 (1) | 2 (1) | 0.08 |  |
|  | hypothetical protein sscle\_15g105380 [Sclerotinia sclerotiorum 1980 UF-70] | | | | | | |

|  |  | Score | Mass | Matches | Sequences | emPAI |  |
| --- | --- | --- | --- | --- | --- | --- | --- |
| 68.1 | gi|154693787|gb|EDN93525.1| | 30 | 42557 | 4 (1) | 2 (1) | 0.08 | hypothetical protein SS1G\_09392 [Sclerotinia sclerotiorum 1980 UF-70] |
|  |  | | | | | | |
|  | 2 samesets of gi|154693787|gb|EDN93525.1| | | | | | | |
|  | gi|156046276|ref|XP\_001589670.1| | 30 | 42557 | 4 (1) | 2 (1) | 0.08 | hypothetical protein SS1G\_09392 [Sclerotinia sclerotiorum 1980 UF-70] |
|  |  | | | | | | |
|  | gi|1095458818|gb|APA15768.1| | 30 | 42557 | 4 (1) | 2 (1) | 0.08 | hypothetical protein sscle\_15g105380 [Sclerotinia sclerotiorum 1980 UF-70] |
|  |  | | | | | | |

#### 4 peptide matches (2 non-duplicate, 2 duplicate)

Auto-fit to window

| Query | Dupes | Observed | Mr(expt) | Mr(calc) | ppm | M | Score | Expect | Rank | U | Peptide |
| --- | --- | --- | --- | --- | --- | --- | --- | --- | --- | --- | --- |

| Query | Dupes | Observed | Mr(expt) | Mr(calc) | ppm | M | Score | Expect | Rank | U | Peptide |
| --- | --- | --- | --- | --- | --- | --- | --- | --- | --- | --- | --- |
| 1580 | 1 | 491.2144 | 980.4142 | 980.4134 | 0.84 | 0 | 17 | 0.21 | 1Score **> 29** indicates **identity** Score **> 23** indicates **homology** |  | R.SGPCGYNAR.V |
| 8733 | 1 | 860.3590 | 1718.7033 | 1718.6995 | 2.24 | 0 | 30 | 0.029 | 1Score **> 27** indicates **identity** | U | R.NDWFTCNEFNSGTK.C |

---

### 69

|  |  | Accession | Score | Description |
| --- | --- | --- | --- | --- |
|  | 1 | gi|154699709|gb|EDN99447.1| | 30 | hypothetical protein SS1G\_02301 [Sclerotinia sclerotiorum 1980 UF-70] |

|  |  | Score | Mass | Matches | Sequences | emPAI |  |
| --- | --- | --- | --- | --- | --- | --- | --- |
| 69.1 | gi|154699709|gb|EDN99447.1| | 30 | 94157 | 4 (1) | 2 (1) | 0.03 |  |
|  | hypothetical protein SS1G\_02301 [Sclerotinia sclerotiorum 1980 UF-70] | | | | | | |
|  | 2 samesets of gi|154699709|gb|EDN99447.1| | | | | | | |
|  | gi|156060325|ref|XP\_001596085.1| | 30 | 94157 | 4 (1) | 2 (1) | 0.03 |  |
|  | hypothetical protein SS1G\_02301 [Sclerotinia sclerotiorum 1980 UF-70] | | | | | | |
|  | gi|1095451626|gb|APA08587.1| | 30 | 94157 | 4 (1) | 2 (1) | 0.03 |  |
|  | hypothetical protein sscle\_04g033570 [Sclerotinia sclerotiorum 1980 UF-70] | | | | | | |

|  |  | Score | Mass | Matches | Sequences | emPAI |  |
| --- | --- | --- | --- | --- | --- | --- | --- |
| 69.1 | gi|154699709|gb|EDN99447.1| | 30 | 94157 | 4 (1) | 2 (1) | 0.03 | hypothetical protein SS1G\_02301 [Sclerotinia sclerotiorum 1980 UF-70] |
|  |  | | | | | | |
|  | 2 samesets of gi|154699709|gb|EDN99447.1| | | | | | | |
|  | gi|156060325|ref|XP\_001596085.1| | 30 | 94157 | 4 (1) | 2 (1) | 0.03 | hypothetical protein SS1G\_02301 [Sclerotinia sclerotiorum 1980 UF-70] |
|  |  | | | | | | |
|  | gi|1095451626|gb|APA08587.1| | 30 | 94157 | 4 (1) | 2 (1) | 0.03 | hypothetical protein sscle\_04g033570 [Sclerotinia sclerotiorum 1980 UF-70] |
|  |  | | | | | | |

#### 4 peptide matches (2 non-duplicate, 2 duplicate)

Auto-fit to window

| Query | Dupes | Observed | Mr(expt) | Mr(calc) | ppm | M | Score | Expect | Rank | U | Peptide |
| --- | --- | --- | --- | --- | --- | --- | --- | --- | --- | --- | --- |

| Query | Dupes | Observed | Mr(expt) | Mr(calc) | ppm | M | Score | Expect | Rank | U | Peptide |
| --- | --- | --- | --- | --- | --- | --- | --- | --- | --- | --- | --- |
| 2451 |  | 540.7535 | 1079.4925 | 1079.5103 | -16.5 | 2 | 1 | 1.6 | 9Score **> 32** indicates **identity** Score **> 15** indicates **homology** | U | R.EMKEREMK.K |
| 4623 | 2 | 633.7942 | 1265.5738 | 1265.6074 | -26.5 | 0 | 30 | 0.0095 | 1Score **> 32** indicates **identity** Score **> 22** indicates **homology** | U | K.GNQIYMELAAR.Y + Deamidated (NQ) |

---

### 70

|  |  | Accession | Score | Description |
| --- | --- | --- | --- | --- |
|  | 1 | gi|347826739|emb|CCD42436.1| | 30 | similar to nuclear cohesin complex subunit (Psc3) [Botrytis cinerea T4] |

|  |  | Score | Mass | Matches | Sequences | emPAI |  |
| --- | --- | --- | --- | --- | --- | --- | --- |
| 70.1 | gi|347826739|emb|CCD42436.1| | 30 | 131413 | 2 (1) | 2 (1) | 0.02 |  |
|  | similar to nuclear cohesin complex subunit (Psc3) [Botrytis cinerea T4] | | | | | | |

|  |  | Score | Mass | Matches | Sequences | emPAI |  |
| --- | --- | --- | --- | --- | --- | --- | --- |
| 70.1 | gi|347826739|emb|CCD42436.1| | 30 | 131413 | 2 (1) | 2 (1) | 0.02 | similar to nuclear cohesin complex subunit (Psc3) [Botrytis cinerea T4] |
|  |  | | | | | | |

#### 2 peptide matches (2 non-duplicate, 0 duplicate)

Auto-fit to window

| Query | Dupes | Observed | Mr(expt) | Mr(calc) | ppm | M | Score | Expect | Rank | U | Peptide |
| --- | --- | --- | --- | --- | --- | --- | --- | --- | --- | --- | --- |

| Query | Dupes | Observed | Mr(expt) | Mr(calc) | ppm | M | Score | Expect | Rank | U | Peptide |
| --- | --- | --- | --- | --- | --- | --- | --- | --- | --- | --- | --- |
| 146 |  | 379.7115 | 757.4085 | 757.4082 | 0.38 | 1 | 0 | 3.4 | 10Score **> 31** indicates **identity** Score **> 18** indicates **homology** |  | K.NKRPNK.G + 2 Deamidated (NQ) |
| 4133 |  | 614.8360 | 1227.6574 | 1227.6207 | 29.9 | 0 | 30 | 0.0067 | 1Score **> 33** indicates **identity** Score **> 21** indicates **homology** | U | K.AQQAAAEAAAQAK.K |

---

### 71

|  |  | Accession | Score | Description |
| --- | --- | --- | --- | --- |
|  | 1 | gi|154702642|gb|EDO02381.1| | 30 | hypothetical protein SS1G\_04857 [Sclerotinia sclerotiorum 1980 UF-70] |

|  |  | Score | Mass | Matches | Sequences | emPAI |  |
| --- | --- | --- | --- | --- | --- | --- | --- |
| 71.1 | gi|154702642|gb|EDO02381.1| | 30 | 14994 | 1 (1) | 1 (1) | 0.23 |  |
|  | hypothetical protein SS1G\_04857 [Sclerotinia sclerotiorum 1980 UF-70] | | | | | | |
|  | 2 samesets of gi|154702642|gb|EDO02381.1| | | | | | | |
|  | gi|156055012|ref|XP\_001593430.1| | 30 | 14994 | 1 (1) | 1 (1) | 0.23 |  |
|  | hypothetical protein SS1G\_04857 [Sclerotinia sclerotiorum 1980 UF-70] | | | | | | |
|  | gi|1095454502|gb|APA11459.1| | 30 | 14994 | 1 (1) | 1 (1) | 0.23 |  |
|  | hypothetical protein sscle\_08g062290 [Sclerotinia sclerotiorum 1980 UF-70] | | | | | | |

|  |  | Score | Mass | Matches | Sequences | emPAI |  |
| --- | --- | --- | --- | --- | --- | --- | --- |
| 71.1 | gi|154702642|gb|EDO02381.1| | 30 | 14994 | 1 (1) | 1 (1) | 0.23 | hypothetical protein SS1G\_04857 [Sclerotinia sclerotiorum 1980 UF-70] |
|  |  | | | | | | |
|  | 2 samesets of gi|154702642|gb|EDO02381.1| | | | | | | |
|  | gi|156055012|ref|XP\_001593430.1| | 30 | 14994 | 1 (1) | 1 (1) | 0.23 | hypothetical protein SS1G\_04857 [Sclerotinia sclerotiorum 1980 UF-70] |
|  |  | | | | | | |
|  | gi|1095454502|gb|APA11459.1| | 30 | 14994 | 1 (1) | 1 (1) | 0.23 | hypothetical protein sscle\_08g062290 [Sclerotinia sclerotiorum 1980 UF-70] |
|  |  | | | | | | |

#### 1 peptide matches (1 non-duplicate, 0 duplicate)

Auto-fit to window

| Query | Dupes | Observed | Mr(expt) | Mr(calc) | ppm | M | Score | Expect | Rank | U | Peptide |
| --- | --- | --- | --- | --- | --- | --- | --- | --- | --- | --- | --- |

| Query | Dupes | Observed | Mr(expt) | Mr(calc) | ppm | M | Score | Expect | Rank | U | Peptide |
| --- | --- | --- | --- | --- | --- | --- | --- | --- | --- | --- | --- |
| 1397 |  | 479.2285 | 956.4424 | 956.4426 | -0.16 | 0 | 30 | 0.045 | 1Score **> 29** indicates **identity** | U | K.CTYSLWK.G |

---

### 72

|  |  | Accession | Score | Description |
| --- | --- | --- | --- | --- |
|  | 1 | gi|347841454|emb|CCD56026.1| | 29 | hypothetical protein BofuT4\_P151580.1 [Botrytis cinerea T4] |

|  |  | Score | Mass | Matches | Sequences | emPAI |  |
| --- | --- | --- | --- | --- | --- | --- | --- |
| 72.1 | gi|347841454|emb|CCD56026.1| | 29 | 88988 | 9 (1) | 7 (1) | 0.04 |  |
|  | hypothetical protein BofuT4\_P151580.1 [Botrytis cinerea T4] | | | | | | |

|  |  | Score | Mass | Matches | Sequences | emPAI |  |
| --- | --- | --- | --- | --- | --- | --- | --- |
| 72.1 | gi|347841454|emb|CCD56026.1| | 29 | 88988 | 9 (1) | 7 (1) | 0.04 | hypothetical protein BofuT4\_P151580.1 [Botrytis cinerea T4] |
|  |  | | | | | | |

#### 9 peptide matches (7 non-duplicate, 2 duplicate)

Auto-fit to window

| Query | Dupes | Observed | Mr(expt) | Mr(calc) | ppm | M | Score | Expect | Rank | U | Peptide |
| --- | --- | --- | --- | --- | --- | --- | --- | --- | --- | --- | --- |

| Query | Dupes | Observed | Mr(expt) | Mr(calc) | ppm | M | Score | Expect | Rank | U | Peptide |
| --- | --- | --- | --- | --- | --- | --- | --- | --- | --- | --- | --- |
| 135 | 1 | 377.1892 | 752.3638 | 752.3785 | -19.6 | 2 | 9 | 0.4 | 1Score **> 30** indicates **identity** Score **> 17** indicates **homology** |  | K.KRMMR.R + 2 Oxidation (M) |
| 208 |  | 389.2487 | 776.4829 | 776.4657 | 22.1 | 1 | 7 | 1.8 | 3Score **> 25** indicates **identity** Score **> 22** indicates **homology** |  | R.RSLFVR.S |
| 452 |  | 414.2403 | 826.4661 | 826.4548 | 13.6 | 0 | 1 | 4.2 | 5Score **> 27** indicates **identity** Score **> 20** indicates **homology** |  | K.IEIAQPR.S + Deamidated (NQ) |
| 1649 |  | 494.2765 | 986.5384 | 986.5396 | -1.23 | 1 | 3 | 2.2 | 5Score **> 34** indicates **identity** Score **> 19** indicates **homology** |  | K.AVEKDQAVK.L |
| 2426 |  | 538.7922 | 1075.5698 | 1075.5622 | 7.11 | 2 | 1 | 6.2 | 6Score **> 33** indicates **identity** Score **> 21** indicates **homology** | U | R.NRSKEVTDK.I |
| 3853 |  | 602.3019 | 1202.5892 | 1202.6142 | -20.8 | 1 | 1 | 1.5 | 3Score **> 34** indicates **identity** Score **> 15** indicates **homology** | U | R.QQLSKEEIAR.G + 2 Deamidated (NQ) |
| 5566 | 1 | 679.3628 | 1356.7111 | 1356.7361 | -18.4 | 2 | 29 | 0.025 | 1Score **> 33** indicates **identity** Score **> 26** indicates **homology** | U | K.KNAEKALAAINGR.E + 2 Deamidated (NQ) |

---

### 73

|  |  | Accession | Score | Description |
| --- | --- | --- | --- | --- |
|  | 1 | gi|347835919|emb|CCD50491.1| | 29 | similar to myo-inositol-1-phosphate synthase [Botrytis cinerea T4] |

|  |  | Score | Mass | Matches | Sequences | emPAI |  |
| --- | --- | --- | --- | --- | --- | --- | --- |
| 73.1 | gi|347835919|emb|CCD50491.1| | 29 | 59424 | 5 (2) | 3 (1) | 0.06 |  |
|  | similar to myo-inositol-1-phosphate synthase [Botrytis cinerea T4] | | | | | | |
|  | 3 samesets of gi|347835919|emb|CCD50491.1| | | | | | | |
|  | gi|154691870|gb|EDN91608.1| | 29 | 58823 | 4 (2) | 2 (1) | 0.06 |  |
|  | conserved hypothetical protein [Sclerotinia sclerotiorum 1980 UF-70] | | | | | | |
|  | gi|156066001|ref|XP\_001598922.1| | 29 | 58823 | 4 (2) | 2 (1) | 0.06 |  |
|  | conserved hypothetical protein [Sclerotinia sclerotiorum 1980 UF-70] | | | | | | |
|  | gi|1095450540|gb|APA07502.1| | 29 | 58823 | 4 (2) | 2 (1) | 0.06 |  |
|  | hypothetical protein sscle\_03g022720 [Sclerotinia sclerotiorum 1980 UF-70] | | | | | | |

|  |  | Score | Mass | Matches | Sequences | emPAI |  |
| --- | --- | --- | --- | --- | --- | --- | --- |
| 73.1 | gi|347835919|emb|CCD50491.1| | 29 | 59424 | 5 (2) | 3 (1) | 0.06 | similar to myo-inositol-1-phosphate synthase [Botrytis cinerea T4] |
|  |  | | | | | | |
|  | 3 samesets of gi|347835919|emb|CCD50491.1| | | | | | | |
|  | gi|154691870|gb|EDN91608.1| | 29 | 58823 | 4 (2) | 2 (1) | 0.06 | conserved hypothetical protein [Sclerotinia sclerotiorum 1980 UF-70] |
|  |  | | | | | | |
|  | gi|156066001|ref|XP\_001598922.1| | 29 | 58823 | 4 (2) | 2 (1) | 0.06 | conserved hypothetical protein [Sclerotinia sclerotiorum 1980 UF-70] |
|  |  | | | | | | |
|  | gi|1095450540|gb|APA07502.1| | 29 | 58823 | 4 (2) | 2 (1) | 0.06 | hypothetical protein sscle\_03g022720 [Sclerotinia sclerotiorum 1980 UF-70] |
|  |  | | | | | | |

#### 5 peptide matches (3 non-duplicate, 2 duplicate)

Auto-fit to window

| Query | Dupes | Observed | Mr(expt) | Mr(calc) | ppm | M | Score | Expect | Rank | U | Peptide |
| --- | --- | --- | --- | --- | --- | --- | --- | --- | --- | --- | --- |

| Query | Dupes | Observed | Mr(expt) | Mr(calc) | ppm | M | Score | Expect | Rank | U | Peptide |
| --- | --- | --- | --- | --- | --- | --- | --- | --- | --- | --- | --- |
| 1293 | 2 | 473.7718 | 945.5290 | 945.5144 | 15.4 | 1 | 28 | 0.016 | 1Score **> 33** indicates **identity** Score **> 22** indicates **homology** | U | R.RGLSWATR.E |
| 1636 |  | 493.7328 | 985.4511 | 985.4287 | 22.7 | 0 | 2 | 1 | 2Score **> 29** indicates **identity** Score **> 14** indicates **homology** | U | K.ACNEHVEK.I |
| 21773 |  | 1244.9396 | 3731.7969 | 3731.8781 | -21.8 | 2 | 0 | 1 | 2Score **> 36** indicates **identity** Score **> 13** indicates **homology** | U | K.NDLDKVIVLWTANTERFAELIDGVNDTAENLLK.A + 3 Deamidated (NQ) |

---

### 74

|  |  | Accession | Score | Description |
| --- | --- | --- | --- | --- |
|  | 1 | gi|154695368|gb|EDN95106.1| | 29 | hypothetical protein SS1G\_10981 [Sclerotinia sclerotiorum 1980 UF-70] |

|  |  | Score | Mass | Matches | Sequences | emPAI |  |
| --- | --- | --- | --- | --- | --- | --- | --- |
| 74.1 | gi|154695368|gb|EDN95106.1| | 29 | 58550 | 7 (1) | 1 (1) | 0.06 |  |
|  | hypothetical protein SS1G\_10981 [Sclerotinia sclerotiorum 1980 UF-70] | | | | | | |
|  | 2 samesets of gi|154695368|gb|EDN95106.1| | | | | | | |
|  | gi|156042368|ref|XP\_001587741.1| | 29 | 58550 | 7 (1) | 1 (1) | 0.06 |  |
|  | hypothetical protein SS1G\_10981 [Sclerotinia sclerotiorum 1980 UF-70] | | | | | | |
|  | gi|1095457213|gb|APA14166.1| | 29 | 58550 | 7 (1) | 1 (1) | 0.06 |  |
|  | hypothetical protein sscle\_12g089360 [Sclerotinia sclerotiorum 1980 UF-70] | | | | | | |

|  |  | Score | Mass | Matches | Sequences | emPAI |  |
| --- | --- | --- | --- | --- | --- | --- | --- |
| 74.1 | gi|154695368|gb|EDN95106.1| | 29 | 58550 | 7 (1) | 1 (1) | 0.06 | hypothetical protein SS1G\_10981 [Sclerotinia sclerotiorum 1980 UF-70] |
|  |  | | | | | | |
|  | 2 samesets of gi|154695368|gb|EDN95106.1| | | | | | | |
|  | gi|156042368|ref|XP\_001587741.1| | 29 | 58550 | 7 (1) | 1 (1) | 0.06 | hypothetical protein SS1G\_10981 [Sclerotinia sclerotiorum 1980 UF-70] |
|  |  | | | | | | |
|  | gi|1095457213|gb|APA14166.1| | 29 | 58550 | 7 (1) | 1 (1) | 0.06 | hypothetical protein sscle\_12g089360 [Sclerotinia sclerotiorum 1980 UF-70] |
|  |  | | | | | | |

#### 7 peptide matches (2 non-duplicate, 5 duplicate)

Auto-fit to window

| Query | Dupes | Observed | Mr(expt) | Mr(calc) | ppm | M | Score | Expect | Rank | U | Peptide |
| --- | --- | --- | --- | --- | --- | --- | --- | --- | --- | --- | --- |

| Query | Dupes | Observed | Mr(expt) | Mr(calc) | ppm | M | Score | Expect | Rank | U | Peptide |
| --- | --- | --- | --- | --- | --- | --- | --- | --- | --- | --- | --- |
| 6998 | 3 | 757.9123 | 1513.8101 | 1513.8352 | -16.5 | 1 | 29 | 0.033 | 1Score **> 33** indicates **identity** Score **> 26** indicates **homology** | U | K.VLGVSKDADELQIK.S |
| 7009 | 2 | 758.4041 | 1514.7937 | 1514.8192 | -16.8 | 1 | 15 | 0.37 | 1Score **> 34** indicates **identity** Score **> 23** indicates **homology** | U | K.VLGVSKDADELQIK.S + Deamidated (NQ) |

---

### 75

|  |  | Accession | Score | Description |
| --- | --- | --- | --- | --- |
|  | 1 | gi|154693995|gb|EDN93733.1| | 28 | predicted protein [Sclerotinia sclerotiorum 1980 UF-70] |

|  |  | Score | Mass | Matches | Sequences | emPAI |  |
| --- | --- | --- | --- | --- | --- | --- | --- |
| 75.1 | gi|154693995|gb|EDN93733.1| | 28 | 41581 | 5 (2) | 2 (1) | 0.08 |  |
|  | predicted protein [Sclerotinia sclerotiorum 1980 UF-70] | | | | | | |
|  | 6 samesets of gi|154693995|gb|EDN93733.1| | | | | | | |
|  | gi|156047821|ref|XP\_001589878.1| | 28 | 41581 | 5 (2) | 2 (1) | 0.08 |  |
|  | predicted protein [Sclerotinia sclerotiorum 1980 UF-70] | | | | | | |
|  | gi|1095458647|gb|APA15597.1| | 28 | 44772 | 5 (2) | 2 (1) | 0.07 |  |
|  | hypothetical protein sscle\_15g103670 [Sclerotinia sclerotiorum 1980 UF-70] | | | | | | |
|  | gi|154701096|gb|EDO00835.1| | 28 | 12377 | 3 (2) | 1 (1) | 0.28 |  |
|  | hypothetical protein SS1G\_03309 [Sclerotinia sclerotiorum 1980 UF-70] | | | | | | |
|  | gi|156058594|ref|XP\_001595220.1| | 28 | 12377 | 3 (2) | 1 (1) | 0.28 |  |
|  | hypothetical protein SS1G\_03309 [Sclerotinia sclerotiorum 1980 UF-70] | | | | | | |
|  | gi|347839374|emb|CCD53946.1| | 28 | 8780 | 3 (2) | 1 (1) | 0.40 |  |
|  | similar to NADH-ubiquinone oxidoreductase 9.5 kDa subunit [Botrytis cinerea T4] | | | | | | |
|  | gi|1095454035|gb|APA10993.1| | 28 | 12377 | 3 (2) | 1 (1) | 0.28 |  |
|  | hypothetical protein sscle\_07g057630 [Sclerotinia sclerotiorum 1980 UF-70] | | | | | | |

|  |  | Score | Mass | Matches | Sequences | emPAI |  |
| --- | --- | --- | --- | --- | --- | --- | --- |
| 75.1 | gi|154693995|gb|EDN93733.1| | 28 | 41581 | 5 (2) | 2 (1) | 0.08 | predicted protein [Sclerotinia sclerotiorum 1980 UF-70] |
|  |  | | | | | | |
|  | 6 samesets of gi|154693995|gb|EDN93733.1| | | | | | | |
|  | gi|156047821|ref|XP\_001589878.1| | 28 | 41581 | 5 (2) | 2 (1) | 0.08 | predicted protein [Sclerotinia sclerotiorum 1980 UF-70] |
|  |  | | | | | | |
|  | gi|1095458647|gb|APA15597.1| | 28 | 44772 | 5 (2) | 2 (1) | 0.07 | hypothetical protein sscle\_15g103670 [Sclerotinia sclerotiorum 1980 UF-70] |
|  |  | | | | | | |
|  | gi|154701096|gb|EDO00835.1| | 28 | 12377 | 3 (2) | 1 (1) | 0.28 | hypothetical protein SS1G\_03309 [Sclerotinia sclerotiorum 1980 UF-70] |
|  |  | | | | | | |
|  | gi|156058594|ref|XP\_001595220.1| | 28 | 12377 | 3 (2) | 1 (1) | 0.28 | hypothetical protein SS1G\_03309 [Sclerotinia sclerotiorum 1980 UF-70] |
|  |  | | | | | | |
|  | gi|347839374|emb|CCD53946.1| | 28 | 8780 | 3 (2) | 1 (1) | 0.40 | similar to NADH-ubiquinone oxidoreductase 9.5 kDa subunit [Botrytis cinerea T4] |
|  |  | | | | | | |
|  | gi|1095454035|gb|APA10993.1| | 28 | 12377 | 3 (2) | 1 (1) | 0.28 | hypothetical protein sscle\_07g057630 [Sclerotinia sclerotiorum 1980 UF-70] |
|  |  | | | | | | |

#### 5 peptide matches (3 non-duplicate, 2 duplicate)

Auto-fit to window

| Query | Dupes | Observed | Mr(expt) | Mr(calc) | ppm | M | Score | Expect | Rank | U | Peptide |
| --- | --- | --- | --- | --- | --- | --- | --- | --- | --- | --- | --- |

| Query | Dupes | Observed | Mr(expt) | Mr(calc) | ppm | M | Score | Expect | Rank | U | Peptide |
| --- | --- | --- | --- | --- | --- | --- | --- | --- | --- | --- | --- |
| 1177 | 2 | 464.7301 | 927.4457 | 927.4450 | 0.74 | 1 | 31 | 0.039 | 1Score **> 30** indicates **identity** | U | R.FFSKEDR.K |
| 1234 |  | 468.7429 | 935.4713 | 935.4712 | 0.087 | 0 | 1 | 2.8 | 7Score **> 33** indicates **identity** Score **> 18** indicates **homology** | U | K.NLELYQR.M + Deamidated (NQ) |
| 1236 |  | 468.7480 | 935.4814 | 935.4712 | 10.9 | 0 | 2 | 1 | 2Score **> 33** indicates **identity** Score **> 15** indicates **homology** | U | K.NLELYQR.M + Deamidated (NQ) |

---

### 76

|  |  | Accession | Score | Description |
| --- | --- | --- | --- | --- |
|  | 1 | gi|347441989|emb|CCD34910.1| | 28 | hypothetical protein BofuT4\_P086140.1 [Botrytis cinerea T4] |

|  |  | Score | Mass | Matches | Sequences | emPAI |  |
| --- | --- | --- | --- | --- | --- | --- | --- |
| 76.1 | gi|347441989|emb|CCD34910.1| | 28 | 41026 | 6 (1) | 2 (1) | 0.08 |  |
|  | hypothetical protein BofuT4\_P086140.1 [Botrytis cinerea T4] | | | | | | |

|  |  | Score | Mass | Matches | Sequences | emPAI |  |
| --- | --- | --- | --- | --- | --- | --- | --- |
| 76.1 | gi|347441989|emb|CCD34910.1| | 28 | 41026 | 6 (1) | 2 (1) | 0.08 | hypothetical protein BofuT4\_P086140.1 [Botrytis cinerea T4] |
|  |  | | | | | | |

#### 6 peptide matches (2 non-duplicate, 4 duplicate)

Auto-fit to window

| Query | Dupes | Observed | Mr(expt) | Mr(calc) | ppm | M | Score | Expect | Rank | U | Peptide |
| --- | --- | --- | --- | --- | --- | --- | --- | --- | --- | --- | --- |

| Query | Dupes | Observed | Mr(expt) | Mr(calc) | ppm | M | Score | Expect | Rank | U | Peptide |
| --- | --- | --- | --- | --- | --- | --- | --- | --- | --- | --- | --- |
| 1769 |  | 503.7508 | 1005.4871 | 1005.5066 | -19.4 | 1 | 1 | 1.8 | 5Score **> 34** indicates **identity** Score **> 16** indicates **homology** | U | -.MNFPTPRK.R + Oxidation (M) |
| 6626 | 4 | 738.3958 | 1474.7771 | 1474.7813 | -2.88 | 2 | 28 | 0.061 | 1Score **> 35** indicates **identity** Score **> 28** indicates **homology** | U | R.EVMNRLEAKELK.N + Oxidation (M) |

---

### 77

|  |  | Accession | Score | Description |
| --- | --- | --- | --- | --- |
|  | 1 | gi|347827155|emb|CCD42852.1| | 28 | hypothetical protein BofuT4\_P071480.1 [Botrytis cinerea T4] |

|  |  | Score | Mass | Matches | Sequences | emPAI |  |
| --- | --- | --- | --- | --- | --- | --- | --- |
| 77.1 | gi|347827155|emb|CCD42852.1| | 28 | 44964 | 3 (1) | 2 (1) | 0.07 |  |
|  | hypothetical protein BofuT4\_P071480.1 [Botrytis cinerea T4] | | | | | | |

|  |  | Score | Mass | Matches | Sequences | emPAI |  |
| --- | --- | --- | --- | --- | --- | --- | --- |
| 77.1 | gi|347827155|emb|CCD42852.1| | 28 | 44964 | 3 (1) | 2 (1) | 0.07 | hypothetical protein BofuT4\_P071480.1 [Botrytis cinerea T4] |
|  |  | | | | | | |

#### 3 peptide matches (2 non-duplicate, 1 duplicate)

Auto-fit to window

| Query | Dupes | Observed | Mr(expt) | Mr(calc) | ppm | M | Score | Expect | Rank | U | Peptide |
| --- | --- | --- | --- | --- | --- | --- | --- | --- | --- | --- | --- |

| Query | Dupes | Observed | Mr(expt) | Mr(calc) | ppm | M | Score | Expect | Rank | U | Peptide |
| --- | --- | --- | --- | --- | --- | --- | --- | --- | --- | --- | --- |
| 196 |  | 387.7293 | 773.4441 | 773.4395 | 5.93 | 1 | 6 | 0.99 | 1Score **> 35** indicates **identity** Score **> 18** indicates **homology** | U | K.AKTTPTR.K |
| 7517 | 1 | 784.3771 | 1566.7397 | 1566.7672 | -17.5 | 1 | 28 | 0.012 | 1Score **> 34** indicates **identity** Score **> 21** indicates **homology** | U | -.MTTTRSGLNSASNVK.Q + Deamidated (NQ) |

---

### 78

|  |  | Accession | Score | Description |
| --- | --- | --- | --- | --- |
|  | 1 | gi|154697145|gb|EDN96883.1| | 28 | hypothetical protein SS1G\_01811 [Sclerotinia sclerotiorum 1980 UF-70] |

|  |  | Score | Mass | Matches | Sequences | emPAI |  |
| --- | --- | --- | --- | --- | --- | --- | --- |
| 78.1 | gi|154697145|gb|EDN96883.1| | 28 | 51236 | 3 (1) | 1 (1) | 0.06 |  |
|  | hypothetical protein SS1G\_01811 [Sclerotinia sclerotiorum 1980 UF-70] | | | | | | |
|  | 2 samesets of gi|154697145|gb|EDN96883.1| | | | | | | |
|  | gi|156063386|ref|XP\_001597615.1| | 28 | 51236 | 3 (1) | 1 (1) | 0.06 |  |
|  | hypothetical protein SS1G\_01811 [Sclerotinia sclerotiorum 1980 UF-70] | | | | | | |
|  | gi|1095448853|gb|APA05817.1| | 28 | 66711 | 3 (1) | 1 (1) | 0.05 |  |
|  | hypothetical protein sscle\_01g005870 [Sclerotinia sclerotiorum 1980 UF-70] | | | | | | |

|  |  | Score | Mass | Matches | Sequences | emPAI |  |
| --- | --- | --- | --- | --- | --- | --- | --- |
| 78.1 | gi|154697145|gb|EDN96883.1| | 28 | 51236 | 3 (1) | 1 (1) | 0.06 | hypothetical protein SS1G\_01811 [Sclerotinia sclerotiorum 1980 UF-70] |
|  |  | | | | | | |
|  | 2 samesets of gi|154697145|gb|EDN96883.1| | | | | | | |
|  | gi|156063386|ref|XP\_001597615.1| | 28 | 51236 | 3 (1) | 1 (1) | 0.06 | hypothetical protein SS1G\_01811 [Sclerotinia sclerotiorum 1980 UF-70] |
|  |  | | | | | | |
|  | gi|1095448853|gb|APA05817.1| | 28 | 66711 | 3 (1) | 1 (1) | 0.05 | hypothetical protein sscle\_01g005870 [Sclerotinia sclerotiorum 1980 UF-70] |
|  |  | | | | | | |

#### 3 peptide matches (2 non-duplicate, 1 duplicate)

Auto-fit to window

| Query | Dupes | Observed | Mr(expt) | Mr(calc) | ppm | M | Score | Expect | Rank | U | Peptide |
| --- | --- | --- | --- | --- | --- | --- | --- | --- | --- | --- | --- |

| Query | Dupes | Observed | Mr(expt) | Mr(calc) | ppm | M | Score | Expect | Rank | U | Peptide |
| --- | --- | --- | --- | --- | --- | --- | --- | --- | --- | --- | --- |
| 3387 |  | 582.3062 | 1162.5977 | 1162.5983 | -0.43 | 0 | 28 | 0.0038 | 1Score **> 34** indicates **identity** Score **> 16** indicates **homology** | U | R.QLGGVVDSNFK.V |
| 3412 | 1 | 583.2936 | 1164.5727 | 1164.5663 | 5.55 | 0 | 2 | 3 | 7Score **> 34** indicates **identity** Score **> 20** indicates **homology** | U | R.QLGGVVDSNFK.V + 2 Deamidated (NQ) |

---

### 79

|  |  | Accession | Score | Description |
| --- | --- | --- | --- | --- |
|  | 1 | gi|154696763|gb|EDN96501.1| | 28 | hypothetical protein SS1G\_01427 [Sclerotinia sclerotiorum 1980 UF-70] |

|  |  | Score | Mass | Matches | Sequences | emPAI |  |
| --- | --- | --- | --- | --- | --- | --- | --- |
| 79.1 | gi|154696763|gb|EDN96501.1| | 28 | 40222 | 2 (1) | 1 (1) | 0.08 |  |
|  | hypothetical protein SS1G\_01427 [Sclerotinia sclerotiorum 1980 UF-70] | | | | | | |
|  | 3 samesets of gi|154696763|gb|EDN96501.1| | | | | | | |
|  | gi|156062622|ref|XP\_001597233.1| | 28 | 40222 | 2 (1) | 1 (1) | 0.08 |  |
|  | hypothetical protein SS1G\_01427 [Sclerotinia sclerotiorum 1980 UF-70] | | | | | | |
|  | gi|347838630|emb|CCD53202.1| | 28 | 36416 | 2 (1) | 1 (1) | 0.09 |  |
|  | hypothetical protein BofuT4\_P122210.1 [Botrytis cinerea T4] | | | | | | |
|  | gi|1095449160|gb|APA06124.1| | 28 | 39607 | 2 (1) | 1 (1) | 0.08 |  |
|  | hypothetical protein sscle\_01g008940 [Sclerotinia sclerotiorum 1980 UF-70] | | | | | | |

|  |  | Score | Mass | Matches | Sequences | emPAI |  |
| --- | --- | --- | --- | --- | --- | --- | --- |
| 79.1 | gi|154696763|gb|EDN96501.1| | 28 | 40222 | 2 (1) | 1 (1) | 0.08 | hypothetical protein SS1G\_01427 [Sclerotinia sclerotiorum 1980 UF-70] |
|  |  | | | | | | |
|  | 3 samesets of gi|154696763|gb|EDN96501.1| | | | | | | |
|  | gi|156062622|ref|XP\_001597233.1| | 28 | 40222 | 2 (1) | 1 (1) | 0.08 | hypothetical protein SS1G\_01427 [Sclerotinia sclerotiorum 1980 UF-70] |
|  |  | | | | | | |
|  | gi|347838630|emb|CCD53202.1| | 28 | 36416 | 2 (1) | 1 (1) | 0.09 | hypothetical protein BofuT4\_P122210.1 [Botrytis cinerea T4] |
|  |  | | | | | | |
|  | gi|1095449160|gb|APA06124.1| | 28 | 39607 | 2 (1) | 1 (1) | 0.08 | hypothetical protein sscle\_01g008940 [Sclerotinia sclerotiorum 1980 UF-70] |
|  |  | | | | | | |

#### 2 peptide matches (1 non-duplicate, 1 duplicate)

Auto-fit to window

| Query | Dupes | Observed | Mr(expt) | Mr(calc) | ppm | M | Score | Expect | Rank | U | Peptide |
| --- | --- | --- | --- | --- | --- | --- | --- | --- | --- | --- | --- |

| Query | Dupes | Observed | Mr(expt) | Mr(calc) | ppm | M | Score | Expect | Rank | U | Peptide |
| --- | --- | --- | --- | --- | --- | --- | --- | --- | --- | --- | --- |
| 7330 | 1 | 774.8092 | 1547.6038 | 1547.6054 | -1.03 | 1 | 28 | 0.023 | 1Score **> 24** indicates **identity** | U | K.DFKECMEQCSAK.A + Oxidation (M) |

---

### 80

|  |  | Accession | Score | Description |
| --- | --- | --- | --- | --- |
|  | 1 | gi|154696419|gb|EDN96157.1| | 27 | hypothetical protein SS1G\_01081 [Sclerotinia sclerotiorum 1980 UF-70] |

|  |  | Score | Mass | Matches | Sequences | emPAI |  |
| --- | --- | --- | --- | --- | --- | --- | --- |
| 80.1 | gi|154696419|gb|EDN96157.1| | 27 | 78216 | 10 (2) | 7 (2) | 0.09 |  |
|  | hypothetical protein SS1G\_01081 [Sclerotinia sclerotiorum 1980 UF-70] | | | | | | |
|  | 3 samesets of gi|154696419|gb|EDN96157.1| | | | | | | |
|  | gi|156061934|ref|XP\_001596889.1| | 27 | 78216 | 10 (2) | 7 (2) | 0.09 |  |
|  | hypothetical protein SS1G\_01081 [Sclerotinia sclerotiorum 1980 UF-70] | | | | | | |
|  | gi|1095449422|gb|APA06386.1| | 27 | 78216 | 10 (2) | 7 (2) | 0.09 |  |
|  | hypothetical protein sscle\_01g011560 [Sclerotinia sclerotiorum 1980 UF-70] | | | | | | |
|  | gi|347835715|emb|CCD50287.1| | 27 | 78599 | 6 (2) | 4 (2) | 0.09 |  |
|  | cat2, catalase [Botrytis cinerea T4] | | | | | | |

|  |  | Score | Mass | Matches | Sequences | emPAI |  |
| --- | --- | --- | --- | --- | --- | --- | --- |
| 80.1 | gi|154696419|gb|EDN96157.1| | 27 | 78216 | 10 (2) | 7 (2) | 0.09 | hypothetical protein SS1G\_01081 [Sclerotinia sclerotiorum 1980 UF-70] |
|  |  | | | | | | |
|  | 3 samesets of gi|154696419|gb|EDN96157.1| | | | | | | |
|  | gi|156061934|ref|XP\_001596889.1| | 27 | 78216 | 10 (2) | 7 (2) | 0.09 | hypothetical protein SS1G\_01081 [Sclerotinia sclerotiorum 1980 UF-70] |
|  |  | | | | | | |
|  | gi|1095449422|gb|APA06386.1| | 27 | 78216 | 10 (2) | 7 (2) | 0.09 | hypothetical protein sscle\_01g011560 [Sclerotinia sclerotiorum 1980 UF-70] |
|  |  | | | | | | |
|  | gi|347835715|emb|CCD50287.1| | 27 | 78599 | 6 (2) | 4 (2) | 0.09 | cat2, catalase [Botrytis cinerea T4] |
|  |  | | | | | | |

#### 10 peptide matches (7 non-duplicate, 3 duplicate)

Auto-fit to window

| Query | Dupes | Observed | Mr(expt) | Mr(calc) | ppm | M | Score | Expect | Rank | U | Peptide |
| --- | --- | --- | --- | --- | --- | --- | --- | --- | --- | --- | --- |

| Query | Dupes | Observed | Mr(expt) | Mr(calc) | ppm | M | Score | Expect | Rank | U | Peptide |
| --- | --- | --- | --- | --- | --- | --- | --- | --- | --- | --- | --- |
| 431 | 1 | 412.7166 | 823.4187 | 823.4188 | -0.12 | 0 | 31 | 0.04 | 1Score **> 30** indicates **identity** | U | R.FSTVAGSR.G |
| 579 | 1 | 423.2457 | 844.4769 | 844.4767 | 0.28 | 0 | 14 | 0.17 | 1Score **> 34** indicates **identity** Score **> 19** indicates **homology** | U | R.TVSGNLVR.T |
| 984 |  | 451.7275 | 901.4405 | 901.4406 | -0.11 | 0 | 21 | 0.048 | 1Score **> 32** indicates **identity** Score **> 20** indicates **homology** | U | R.DVHGFATR.F |
| 3935 | 1 | 605.3046 | 1208.5947 | 1208.5938 | 0.74 | 0 | 7 | 0.51 | 1Score **> 34** indicates **identity** Score **> 16** indicates **homology** | U | K.STYFPADRPR.Q |
| 6269 |  | 717.3395 | 1432.6644 | 1432.6834 | -13.3 | 0 | 0 | 1 | 2Score **> 33** indicates **identity** Score **> 13** indicates **homology** | U | R.GVDFTDDPLLQGR.I + Deamidated (NQ) |
| 14863 |  | 883.1092 | 2646.3057 | 2646.2687 | 14.0 | 0 | 2 | 1.1 | 9Score **> 35** indicates **identity** Score **> 15** indicates **homology** | U | R.HGGPNFEQLPINRPHVPVHNNNR.D + 4 Deamidated (NQ) |
| 16534 |  | 1432.1685 | 2862.3224 | 2862.3096 | 4.44 | 0 | 0 | 0.96 | 1Score **> 35** indicates **identity** Score **> 13** indicates **homology** | U | R.GAGAHGVFTSYGNYSNITAASFLNEQGK.E + 2 Deamidated (NQ) |

---

### 81

|  |  | Accession | Score | Description |
| --- | --- | --- | --- | --- |
|  | 1 | gi|154701069|gb|EDO00808.1| | 27 | serine protease [Sclerotinia sclerotiorum 1980 UF-70] |

|  |  | Score | Mass | Matches | Sequences | emPAI |  |
| --- | --- | --- | --- | --- | --- | --- | --- |
| 81.1 | gi|154701069|gb|EDO00808.1| | 27 | 55574 | 2 (2) | 1 (1) | 0.06 |  |
|  | serine protease [Sclerotinia sclerotiorum 1980 UF-70] | | | | | | |
|  | 2 samesets of gi|154701069|gb|EDO00808.1| | | | | | | |
|  | gi|156058540|ref|XP\_001595193.1| | 27 | 55574 | 2 (2) | 1 (1) | 0.06 |  |
|  | serine protease [Sclerotinia sclerotiorum 1980 UF-70] | | | | | | |
|  | gi|1095454054|gb|APA11012.1| | 27 | 55574 | 2 (2) | 1 (1) | 0.06 |  |
|  | hypothetical protein sscle\_07g057820 [Sclerotinia sclerotiorum 1980 UF-70] | | | | | | |

|  |  | Score | Mass | Matches | Sequences | emPAI |  |
| --- | --- | --- | --- | --- | --- | --- | --- |
| 81.1 | gi|154701069|gb|EDO00808.1| | 27 | 55574 | 2 (2) | 1 (1) | 0.06 | serine protease [Sclerotinia sclerotiorum 1980 UF-70] |
|  |  | | | | | | |
|  | 2 samesets of gi|154701069|gb|EDO00808.1| | | | | | | |
|  | gi|156058540|ref|XP\_001595193.1| | 27 | 55574 | 2 (2) | 1 (1) | 0.06 | serine protease [Sclerotinia sclerotiorum 1980 UF-70] |
|  |  | | | | | | |
|  | gi|1095454054|gb|APA11012.1| | 27 | 55574 | 2 (2) | 1 (1) | 0.06 | hypothetical protein sscle\_07g057820 [Sclerotinia sclerotiorum 1980 UF-70] |
|  |  | | | | | | |

#### 2 peptide matches (1 non-duplicate, 1 duplicate)

Auto-fit to window

| Query | Dupes | Observed | Mr(expt) | Mr(calc) | ppm | M | Score | Expect | Rank | U | Peptide |
| --- | --- | --- | --- | --- | --- | --- | --- | --- | --- | --- | --- |

| Query | Dupes | Observed | Mr(expt) | Mr(calc) | ppm | M | Score | Expect | Rank | U | Peptide |
| --- | --- | --- | --- | --- | --- | --- | --- | --- | --- | --- | --- |
| 5277 | 1 | 664.8142 | 1327.6137 | 1327.6520 | -28.8 | 1 | 26 | 0.021 | 1Score **> 32** indicates **identity** Score **> 22** indicates **homology** | U | K.GVEWAANSHTKK.V + Deamidated (NQ) |

---

### 82

|  |  | Accession | Score | Description |
| --- | --- | --- | --- | --- |
|  | 1 | gi|347829529|emb|CCD45226.1| | 27 | similar to CHL4 family chromosome segregation protein [Botrytis cinerea T4] |

|  |  | Score | Mass | Matches | Sequences | emPAI |  |
| --- | --- | --- | --- | --- | --- | --- | --- |
| 82.1 | gi|347829529|emb|CCD45226.1| | 27 | 57666 | 2 (1) | 2 (1) | 0.06 |  |
|  | similar to CHL4 family chromosome segregation protein [Botrytis cinerea T4] | | | | | | |
|  | 3 samesets of gi|347829529|emb|CCD45226.1| | | | | | | |
|  | gi|154695332|gb|EDN95070.1| | 27 | 57501 | 2 (1) | 2 (1) | 0.06 |  |
|  | hypothetical protein SS1G\_10945 [Sclerotinia sclerotiorum 1980 UF-70] | | | | | | |
|  | gi|156043883|ref|XP\_001588498.1| | 27 | 57501 | 2 (1) | 2 (1) | 0.06 |  |
|  | hypothetical protein SS1G\_10945 [Sclerotinia sclerotiorum 1980 UF-70] | | | | | | |
|  | gi|1095455143|gb|APA12099.1| | 27 | 57501 | 2 (1) | 2 (1) | 0.06 |  |
|  | hypothetical protein sscle\_09g068690 [Sclerotinia sclerotiorum 1980 UF-70] | | | | | | |

|  |  | Score | Mass | Matches | Sequences | emPAI |  |
| --- | --- | --- | --- | --- | --- | --- | --- |
| 82.1 | gi|347829529|emb|CCD45226.1| | 27 | 57666 | 2 (1) | 2 (1) | 0.06 | similar to CHL4 family chromosome segregation protein [Botrytis cinerea T4] |
|  |  | | | | | | |
|  | 3 samesets of gi|347829529|emb|CCD45226.1| | | | | | | |
|  | gi|154695332|gb|EDN95070.1| | 27 | 57501 | 2 (1) | 2 (1) | 0.06 | hypothetical protein SS1G\_10945 [Sclerotinia sclerotiorum 1980 UF-70] |
|  |  | | | | | | |
|  | gi|156043883|ref|XP\_001588498.1| | 27 | 57501 | 2 (1) | 2 (1) | 0.06 | hypothetical protein SS1G\_10945 [Sclerotinia sclerotiorum 1980 UF-70] |
|  |  | | | | | | |
|  | gi|1095455143|gb|APA12099.1| | 27 | 57501 | 2 (1) | 2 (1) | 0.06 | hypothetical protein sscle\_09g068690 [Sclerotinia sclerotiorum 1980 UF-70] |
|  |  | | | | | | |

#### 2 peptide matches (2 non-duplicate, 0 duplicate)

Auto-fit to window

| Query | Dupes | Observed | Mr(expt) | Mr(calc) | ppm | M | Score | Expect | Rank | U | Peptide |
| --- | --- | --- | --- | --- | --- | --- | --- | --- | --- | --- | --- |

| Query | Dupes | Observed | Mr(expt) | Mr(calc) | ppm | M | Score | Expect | Rank | U | Peptide |
| --- | --- | --- | --- | --- | --- | --- | --- | --- | --- | --- | --- |
| 332 |  | 400.7295 | 799.4444 | 799.4552 | -13.5 | 1 | 27 | 0.051 | 1Score **> 31** indicates **identity** Score **> 26** indicates **homology** | U | K.KDTAIPR.F |
| 24240 |  | 1647.0947 | 4938.2624 | 4938.2771 | -2.98 | 0 | 5 | 1.1 | 3Score **> 34** indicates **identity** Score **> 18** indicates **homology** | U | R.NQINTAPYLLETDDSASDYAQDLYPPHATLDSLSELYTSFTTQK.G + 2 Deamidated (NQ) |

---

### 83

|  |  | Accession | Score | Description |
| --- | --- | --- | --- | --- |
|  | 1 | gi|154698344|gb|EDN98082.1| | 27 | hypothetical protein SS1G\_12939 [Sclerotinia sclerotiorum 1980 UF-70] |

|  |  | Score | Mass | Matches | Sequences | emPAI |  |
| --- | --- | --- | --- | --- | --- | --- | --- |
| 83.1 | gi|154698344|gb|EDN98082.1| | 27 | 65158 | 4 (1) | 4 (1) | 0.05 |  |
|  | hypothetical protein SS1G\_12939 [Sclerotinia sclerotiorum 1980 UF-70] | | | | | | |
|  | 2 samesets of gi|154698344|gb|EDN98082.1| | | | | | | |
|  | gi|156036500|ref|XP\_001586361.1| | 27 | 65158 | 4 (1) | 4 (1) | 0.05 |  |
|  | hypothetical protein SS1G\_12939 [Sclerotinia sclerotiorum 1980 UF-70] | | | | | | |
|  | gi|1095449438|gb|APA06401.1| | 27 | 64701 | 4 (1) | 4 (1) | 0.05 |  |
|  | hypothetical protein sscle\_02g011710 [Sclerotinia sclerotiorum 1980 UF-70] | | | | | | |

|  |  | Score | Mass | Matches | Sequences | emPAI |  |
| --- | --- | --- | --- | --- | --- | --- | --- |
| 83.1 | gi|154698344|gb|EDN98082.1| | 27 | 65158 | 4 (1) | 4 (1) | 0.05 | hypothetical protein SS1G\_12939 [Sclerotinia sclerotiorum 1980 UF-70] |
|  |  | | | | | | |
|  | 2 samesets of gi|154698344|gb|EDN98082.1| | | | | | | |
|  | gi|156036500|ref|XP\_001586361.1| | 27 | 65158 | 4 (1) | 4 (1) | 0.05 | hypothetical protein SS1G\_12939 [Sclerotinia sclerotiorum 1980 UF-70] |
|  |  | | | | | | |
|  | gi|1095449438|gb|APA06401.1| | 27 | 64701 | 4 (1) | 4 (1) | 0.05 | hypothetical protein sscle\_02g011710 [Sclerotinia sclerotiorum 1980 UF-70] |
|  |  | | | | | | |

#### 4 peptide matches (4 non-duplicate, 0 duplicate)

Auto-fit to window

| Query | Dupes | Observed | Mr(expt) | Mr(calc) | ppm | M | Score | Expect | Rank | U | Peptide |
| --- | --- | --- | --- | --- | --- | --- | --- | --- | --- | --- | --- |

| Query | Dupes | Observed | Mr(expt) | Mr(calc) | ppm | M | Score | Expect | Rank | U | Peptide |
| --- | --- | --- | --- | --- | --- | --- | --- | --- | --- | --- | --- |
| 807 |  | 441.2273 | 880.4400 | 880.4654 | -28.9 | 1 | 1 | 3.3 | 6Score **> 31** indicates **identity** Score **> 18** indicates **homology** | U | R.TVKYNAGK.A + Deamidated (NQ) |
| 2418 |  | 538.7830 | 1075.5515 | 1075.5510 | 0.49 | 0 | 27 | 0.032 | 1Score **> 34** indicates **identity** Score **> 24** indicates **homology** | U | R.TSGGVVDSNLK.V |
| 4912 |  | 646.8261 | 1291.6375 | 1291.6707 | -25.6 | 1 | 3 | 1 | 3Score **> 34** indicates **identity** Score **> 16** indicates **homology** | U | -.MLWIRNSSLR.S + Deamidated (NQ); Oxidation (M) |
| 7442 |  | 779.3691 | 1556.7236 | 1556.7505 | -17.2 | 0 | 6 | 1 | 1Score **> 33** indicates **identity** Score **> 19** indicates **homology** |  | K.ALGGTSTINGMTYVR.A + Deamidated (NQ); Oxidation (M) |

---

### 84

|  |  | Accession | Score | Description |
| --- | --- | --- | --- | --- |
|  | 1 | gi|154693043|gb|EDN92781.1| | 26 | hypothetical protein SS1G\_08645 [Sclerotinia sclerotiorum 1980 UF-70] |

|  |  | Score | Mass | Matches | Sequences | emPAI |  |
| --- | --- | --- | --- | --- | --- | --- | --- |
| 84.1 | gi|154693043|gb|EDN92781.1| | 26 | 52373 | 2 (1) | 1 (1) | 0.06 |  |
|  | hypothetical protein SS1G\_08645 [Sclerotinia sclerotiorum 1980 UF-70] | | | | | | |
|  | 2 samesets of gi|154693043|gb|EDN92781.1| | | | | | | |
|  | gi|156049875|ref|XP\_001590904.1| | 26 | 52373 | 2 (1) | 1 (1) | 0.06 |  |
|  | hypothetical protein SS1G\_08645 [Sclerotinia sclerotiorum 1980 UF-70] | | | | | | |
|  | gi|1095456168|gb|APA13123.1| | 26 | 52373 | 2 (1) | 1 (1) | 0.06 |  |
|  | hypothetical protein sscle\_10g078930 [Sclerotinia sclerotiorum 1980 UF-70] | | | | | | |

|  |  | Score | Mass | Matches | Sequences | emPAI |  |
| --- | --- | --- | --- | --- | --- | --- | --- |
| 84.1 | gi|154693043|gb|EDN92781.1| | 26 | 52373 | 2 (1) | 1 (1) | 0.06 | hypothetical protein SS1G\_08645 [Sclerotinia sclerotiorum 1980 UF-70] |
|  |  | | | | | | |
|  | 2 samesets of gi|154693043|gb|EDN92781.1| | | | | | | |
|  | gi|156049875|ref|XP\_001590904.1| | 26 | 52373 | 2 (1) | 1 (1) | 0.06 | hypothetical protein SS1G\_08645 [Sclerotinia sclerotiorum 1980 UF-70] |
|  |  | | | | | | |
|  | gi|1095456168|gb|APA13123.1| | 26 | 52373 | 2 (1) | 1 (1) | 0.06 | hypothetical protein sscle\_10g078930 [Sclerotinia sclerotiorum 1980 UF-70] |
|  |  | | | | | | |

#### 2 peptide matches (1 non-duplicate, 1 duplicate)

Auto-fit to window

| Query | Dupes | Observed | Mr(expt) | Mr(calc) | ppm | M | Score | Expect | Rank | U | Peptide |
| --- | --- | --- | --- | --- | --- | --- | --- | --- | --- | --- | --- |

| Query | Dupes | Observed | Mr(expt) | Mr(calc) | ppm | M | Score | Expect | Rank | U | Peptide |
| --- | --- | --- | --- | --- | --- | --- | --- | --- | --- | --- | --- |
| 2574 | 1 | 546.7516 | 1091.4886 | 1091.4883 | 0.26 | 0 | 26 | 0.046 | 1Score **> 31** indicates **identity** Score **> 26** indicates **homology** | U | K.FQNENTDPK.A |

---

### 85

|  |  | Accession | Score | Description |
| --- | --- | --- | --- | --- |
|  | 1 | gi|154693434|gb|EDN93172.1| | 26 | formate dehydrogenase [Sclerotinia sclerotiorum 1980 UF-70] |

|  |  | Score | Mass | Matches | Sequences | emPAI |  |
| --- | --- | --- | --- | --- | --- | --- | --- |
| 85.1 | gi|154693434|gb|EDN93172.1| | 26 | 48163 | 3 (1) | 2 (1) | 0.07 |  |
|  | formate dehydrogenase [Sclerotinia sclerotiorum 1980 UF-70] | | | | | | |
|  | 5 samesets of gi|154693434|gb|EDN93172.1| | | | | | | |
|  | gi|156048612|ref|XP\_001590273.1| | 26 | 48163 | 3 (1) | 2 (1) | 0.07 |  |
|  | formate dehydrogenase [Sclerotinia sclerotiorum 1980 UF-70] | | | | | | |
|  | gi|347841449|emb|CCD56021.1| | 26 | 48205 | 3 (1) | 2 (1) | 0.07 |  |
|  | similar to NAD-dependent formate dehydrogenase [Botrytis cinerea T4] | | | | | | |
|  | gi|406863832|gb|EKD16879.1| | 26 | 86394 | 3 (1) | 2 (1) | 0.04 |  |
|  | formate dehydrogenase [Marssonina brunnea f. sp. 'multigermtubi' MB\_m1] | | | | | | |
|  | gi|597580361|ref|XP\_007293237.1| | 26 | 86394 | 3 (1) | 2 (1) | 0.04 |  |
|  | formate dehydrogenase [Marssonina brunnea f. sp. 'multigermtubi' MB\_m1] | | | | | | |
|  | gi|1095458429|gb|APA15380.1| | 26 | 41111 | 3 (1) | 2 (1) | 0.08 |  |
|  | hypothetical protein sscle\_14g101500 [Sclerotinia sclerotiorum 1980 UF-70] | | | | | | |

|  |  | Score | Mass | Matches | Sequences | emPAI |  |
| --- | --- | --- | --- | --- | --- | --- | --- |
| 85.1 | gi|154693434|gb|EDN93172.1| | 26 | 48163 | 3 (1) | 2 (1) | 0.07 | formate dehydrogenase [Sclerotinia sclerotiorum 1980 UF-70] |
|  |  | | | | | | |
|  | 5 samesets of gi|154693434|gb|EDN93172.1| | | | | | | |
|  | gi|156048612|ref|XP\_001590273.1| | 26 | 48163 | 3 (1) | 2 (1) | 0.07 | formate dehydrogenase [Sclerotinia sclerotiorum 1980 UF-70] |
|  |  | | | | | | |
|  | gi|347841449|emb|CCD56021.1| | 26 | 48205 | 3 (1) | 2 (1) | 0.07 | similar to NAD-dependent formate dehydrogenase [Botrytis cinerea T4] |
|  |  | | | | | | |
|  | gi|406863832|gb|EKD16879.1| | 26 | 86394 | 3 (1) | 2 (1) | 0.04 | formate dehydrogenase [Marssonina brunnea f. sp. 'multigermtubi' MB\_m1] |
|  |  | | | | | | |
|  | gi|597580361|ref|XP\_007293237.1| | 26 | 86394 | 3 (1) | 2 (1) | 0.04 | formate dehydrogenase [Marssonina brunnea f. sp. 'multigermtubi' MB\_m1] |
|  |  | | | | | | |
|  | gi|1095458429|gb|APA15380.1| | 26 | 41111 | 3 (1) | 2 (1) | 0.08 | hypothetical protein sscle\_14g101500 [Sclerotinia sclerotiorum 1980 UF-70] |
|  |  | | | | | | |

#### 3 peptide matches (2 non-duplicate, 1 duplicate)

Auto-fit to window

| Query | Dupes | Observed | Mr(expt) | Mr(calc) | ppm | M | Score | Expect | Rank | U | Peptide |
| --- | --- | --- | --- | --- | --- | --- | --- | --- | --- | --- | --- |

| Query | Dupes | Observed | Mr(expt) | Mr(calc) | ppm | M | Score | Expect | Rank | U | Peptide |
| --- | --- | --- | --- | --- | --- | --- | --- | --- | --- | --- | --- |
| 552 | 1 | 421.7585 | 841.5025 | 841.5134 | -13.0 | 1 | 7 | 2 | 10Score **> 30** indicates **identity** Score **> 23** indicates **homology** | U | R.IGERVLR.R |
| 669 |  | 429.2635 | 856.5124 | 856.5131 | -0.76 | 0 | 26 | 0.045 | 1Score **> 32** indicates **identity** Score **> 25** indicates **homology** | U | K.VVGTVAVGR.I |

---

### 86

|  |  | Accession | Score | Description |
| --- | --- | --- | --- | --- |
|  | 1 | gi|154694870|gb|EDN94608.1| | 26 | hypothetical protein SS1G\_10482 [Sclerotinia sclerotiorum 1980 UF-70] |

|  |  | Score | Mass | Matches | Sequences | emPAI |  |
| --- | --- | --- | --- | --- | --- | --- | --- |
| 86.1 | gi|154694870|gb|EDN94608.1| | 26 | 38128 | 2 (1) | 1 (1) | 0.09 |  |
|  | hypothetical protein SS1G\_10482 [Sclerotinia sclerotiorum 1980 UF-70] | | | | | | |
|  | 2 samesets of gi|154694870|gb|EDN94608.1| | | | | | | |
|  | gi|156044756|ref|XP\_001588934.1| | 26 | 38128 | 2 (1) | 1 (1) | 0.09 |  |
|  | hypothetical protein SS1G\_10482 [Sclerotinia sclerotiorum 1980 UF-70] | | | | | | |
|  | gi|1095459324|gb|APA16273.1| | 26 | 38128 | 2 (1) | 1 (1) | 0.09 |  |
|  | hypothetical protein sscle\_16g110430 [Sclerotinia sclerotiorum 1980 UF-70] | | | | | | |

|  |  | Score | Mass | Matches | Sequences | emPAI |  |
| --- | --- | --- | --- | --- | --- | --- | --- |
| 86.1 | gi|154694870|gb|EDN94608.1| | 26 | 38128 | 2 (1) | 1 (1) | 0.09 | hypothetical protein SS1G\_10482 [Sclerotinia sclerotiorum 1980 UF-70] |
|  |  | | | | | | |
|  | 2 samesets of gi|154694870|gb|EDN94608.1| | | | | | | |
|  | gi|156044756|ref|XP\_001588934.1| | 26 | 38128 | 2 (1) | 1 (1) | 0.09 | hypothetical protein SS1G\_10482 [Sclerotinia sclerotiorum 1980 UF-70] |
|  |  | | | | | | |
|  | gi|1095459324|gb|APA16273.1| | 26 | 38128 | 2 (1) | 1 (1) | 0.09 | hypothetical protein sscle\_16g110430 [Sclerotinia sclerotiorum 1980 UF-70] |
|  |  | | | | | | |

#### 2 peptide matches (1 non-duplicate, 1 duplicate)

Auto-fit to window

| Query | Dupes | Observed | Mr(expt) | Mr(calc) | ppm | M | Score | Expect | Rank | U | Peptide |
| --- | --- | --- | --- | --- | --- | --- | --- | --- | --- | --- | --- |

| Query | Dupes | Observed | Mr(expt) | Mr(calc) | ppm | M | Score | Expect | Rank | U | Peptide |
| --- | --- | --- | --- | --- | --- | --- | --- | --- | --- | --- | --- |
| 372 | 1 | 405.7452 | 809.4759 | 809.4759 | -0.067 | 0 | 26 | 0.0091 | 1Score **> 26** indicates **identity** Score **> 18** indicates **homology** | U | R.TPPNLIR.A |

---

### 87

|  |  | Accession | Score | Description |
| --- | --- | --- | --- | --- |
|  | 1 | gi|347830192|emb|CCD45889.1| | 25 | glycoside hydrolase family 27 protein [Botrytis cinerea T4] |

|  |  | Score | Mass | Matches | Sequences | emPAI |  |
| --- | --- | --- | --- | --- | --- | --- | --- |
| 87.1 | gi|347830192|emb|CCD45889.1| | 25 | 59101 | 4 (1) | 3 (1) | 0.06 |  |
|  | glycoside hydrolase family 27 protein [Botrytis cinerea T4] | | | | | | |

|  |  | Score | Mass | Matches | Sequences | emPAI |  |
| --- | --- | --- | --- | --- | --- | --- | --- |
| 87.1 | gi|347830192|emb|CCD45889.1| | 25 | 59101 | 4 (1) | 3 (1) | 0.06 | glycoside hydrolase family 27 protein [Botrytis cinerea T4] |
|  |  | | | | | | |

#### 4 peptide matches (3 non-duplicate, 1 duplicate)

Auto-fit to window

| Query | Dupes | Observed | Mr(expt) | Mr(calc) | ppm | M | Score | Expect | Rank | U | Peptide |
| --- | --- | --- | --- | --- | --- | --- | --- | --- | --- | --- | --- |

| Query | Dupes | Observed | Mr(expt) | Mr(calc) | ppm | M | Score | Expect | Rank | U | Peptide |
| --- | --- | --- | --- | --- | --- | --- | --- | --- | --- | --- | --- |
| 459 | 1 | 414.7686 | 827.5225 | 827.5229 | -0.39 | 0 | 25 | 0.044 | 1Score **> 29** indicates **identity** Score **> 24** indicates **homology** | U | R.LLTVINR.G |
| 1224 |  | 468.2505 | 934.4865 | 934.4872 | -0.76 | 0 | 11 | 8.2 | 4Score **> 33** indicates **identity** Score **> 33** indicates **homology** |  | K.GLLAAGYNR.L + Deamidated (NQ) |
| 21794 |  | 935.1849 | 3736.7104 | 3736.7427 | -8.64 | 2 | 1 | 0.92 | 1Score **> 35** indicates **identity** Score **> 13** indicates **homology** | U | K.GLLAAGYNRLNLDDCWSQGSREPNGSLLWNTEK.F + 3 Deamidated (NQ) |

---

### 88

|  |  | Accession | Score | Description |
| --- | --- | --- | --- | --- |
|  | 1 | gi|347837155|emb|CCD51727.1| | 25 | similar to MFS multidrug transporter [Botrytis cinerea T4] |

|  |  | Score | Mass | Matches | Sequences | emPAI |  |
| --- | --- | --- | --- | --- | --- | --- | --- |
| 88.1 | gi|347837155|emb|CCD51727.1| | 25 | 54711 | 2 (1) | 1 (1) | 0.06 |  |
|  | similar to MFS multidrug transporter [Botrytis cinerea T4] | | | | | | |

|  |  | Score | Mass | Matches | Sequences | emPAI |  |
| --- | --- | --- | --- | --- | --- | --- | --- |
| 88.1 | gi|347837155|emb|CCD51727.1| | 25 | 54711 | 2 (1) | 1 (1) | 0.06 | similar to MFS multidrug transporter [Botrytis cinerea T4] |
|  |  | | | | | | |

#### 2 peptide matches (1 non-duplicate, 1 duplicate)

Auto-fit to window

| Query | Dupes | Observed | Mr(expt) | Mr(calc) | ppm | M | Score | Expect | Rank | U | Peptide |
| --- | --- | --- | --- | --- | --- | --- | --- | --- | --- | --- | --- |

| Query | Dupes | Observed | Mr(expt) | Mr(calc) | ppm | M | Score | Expect | Rank | U | Peptide |
| --- | --- | --- | --- | --- | --- | --- | --- | --- | --- | --- | --- |
| 462 | 1 | 414.7690 | 827.5235 | 827.5229 | 0.70 | 0 | 25 | 0.037 | 1Score **> 29** indicates **identity** Score **> 24** indicates **homology** | U | R.IGTVGILR.A |

---

### 89

|  |  | Accession | Score | Description |
| --- | --- | --- | --- | --- |
|  | 1 | gi|347835242|emb|CCD49814.1| | 25 | hypothetical protein BofuT4\_P095220.1 [Botrytis cinerea T4] |

|  |  | Score | Mass | Matches | Sequences | emPAI |  |
| --- | --- | --- | --- | --- | --- | --- | --- |
| 89.1 | gi|347835242|emb|CCD49814.1| | 25 | 58496 | 2 (1) | 1 (1) | 0.06 |  |
|  | hypothetical protein BofuT4\_P095220.1 [Botrytis cinerea T4] | | | | | | |

|  |  | Score | Mass | Matches | Sequences | emPAI |  |
| --- | --- | --- | --- | --- | --- | --- | --- |
| 89.1 | gi|347835242|emb|CCD49814.1| | 25 | 58496 | 2 (1) | 1 (1) | 0.06 | hypothetical protein BofuT4\_P095220.1 [Botrytis cinerea T4] |
|  |  | | | | | | |

#### 2 peptide matches (1 non-duplicate, 1 duplicate)

Auto-fit to window

| Query | Dupes | Observed | Mr(expt) | Mr(calc) | ppm | M | Score | Expect | Rank | U | Peptide |
| --- | --- | --- | --- | --- | --- | --- | --- | --- | --- | --- | --- |

| Query | Dupes | Observed | Mr(expt) | Mr(calc) | ppm | M | Score | Expect | Rank | U | Peptide |
| --- | --- | --- | --- | --- | --- | --- | --- | --- | --- | --- | --- |
| 105 | 1 | 371.2516 | 740.4886 | 740.5021 | -18.2 | 1 | 25 | 0.029 | 1Score **> 22** indicates **identity** | U | R.RILLAR.L |

---

### 90

|  |  | Accession | Score | Description |
| --- | --- | --- | --- | --- |
|  | 1 | gi|154695175|gb|EDN94913.1| | 25 | hypothetical protein SS1G\_10788 [Sclerotinia sclerotiorum 1980 UF-70] |

|  |  | Score | Mass | Matches | Sequences | emPAI |  |
| --- | --- | --- | --- | --- | --- | --- | --- |
| 90.1 | gi|154695175|gb|EDN94913.1| | 25 | 112161 | 3 (1) | 3 (1) | 0.03 |  |
|  | hypothetical protein SS1G\_10788 [Sclerotinia sclerotiorum 1980 UF-70] | | | | | | |
|  | 3 samesets of gi|154695175|gb|EDN94913.1| | | | | | | |
|  | gi|156043569|ref|XP\_001588341.1| | 25 | 112161 | 3 (1) | 3 (1) | 0.03 |  |
|  | hypothetical protein SS1G\_10788 [Sclerotinia sclerotiorum 1980 UF-70] | | | | | | |
|  | gi|1095455264|gb|APA12220.1| | 25 | 111085 | 3 (1) | 3 (1) | 0.03 |  |
|  | hypothetical protein sscle\_09g069900 [Sclerotinia sclerotiorum 1980 UF-70] | | | | | | |
|  | gi|347829353|emb|CCD45050.1| | 25 | 111127 | 2 (1) | 2 (1) | 0.03 |  |
|  | similar to oxysterol-binding protein [Botrytis cinerea T4] | | | | | | |

|  |  | Score | Mass | Matches | Sequences | emPAI |  |
| --- | --- | --- | --- | --- | --- | --- | --- |
| 90.1 | gi|154695175|gb|EDN94913.1| | 25 | 112161 | 3 (1) | 3 (1) | 0.03 | hypothetical protein SS1G\_10788 [Sclerotinia sclerotiorum 1980 UF-70] |
|  |  | | | | | | |
|  | 3 samesets of gi|154695175|gb|EDN94913.1| | | | | | | |
|  | gi|156043569|ref|XP\_001588341.1| | 25 | 112161 | 3 (1) | 3 (1) | 0.03 | hypothetical protein SS1G\_10788 [Sclerotinia sclerotiorum 1980 UF-70] |
|  |  | | | | | | |
|  | gi|1095455264|gb|APA12220.1| | 25 | 111085 | 3 (1) | 3 (1) | 0.03 | hypothetical protein sscle\_09g069900 [Sclerotinia sclerotiorum 1980 UF-70] |
|  |  | | | | | | |
|  | gi|347829353|emb|CCD45050.1| | 25 | 111127 | 2 (1) | 2 (1) | 0.03 | similar to oxysterol-binding protein [Botrytis cinerea T4] |
|  |  | | | | | | |

#### 3 peptide matches (3 non-duplicate, 0 duplicate)

Auto-fit to window

| Query | Dupes | Observed | Mr(expt) | Mr(calc) | ppm | M | Score | Expect | Rank | U | Peptide |
| --- | --- | --- | --- | --- | --- | --- | --- | --- | --- | --- | --- |

| Query | Dupes | Observed | Mr(expt) | Mr(calc) | ppm | M | Score | Expect | Rank | U | Peptide |
| --- | --- | --- | --- | --- | --- | --- | --- | --- | --- | --- | --- |
| 251 |  | 392.7192 | 783.4238 | 783.4351 | -14.4 | 0 | 9 | 1.2 | 2Score **> 27** indicates **identity** Score **> 23** indicates **homology** |  | R.LRPDQR.A |
| 5876 |  | 695.3306 | 1388.6466 | 1388.6428 | 2.73 | 0 | 25 | 0.018 | 1Score **> 33** indicates **identity** Score **> 20** indicates **homology** | U | -.MAGMEQLEIHSK.S + Oxidation (M) |
| 6164 |  | 474.5683 | 1420.6831 | 1420.6681 | 10.5 | 1 | 0 | 1.1 | 2Score **> 34** indicates **identity** Score **> 13** indicates **homology** | U | R.NDSSTAQEQLKAK.G + 2 Deamidated (NQ) |

---

### 91

|  |  | Accession | Score | Description |
| --- | --- | --- | --- | --- |
|  | 1 | gi|347828924|emb|CCD44621.1| | 25 | hypothetical protein BofuT4\_P055220.1 [Botrytis cinerea T4] |

|  |  | Score | Mass | Matches | Sequences | emPAI |  |
| --- | --- | --- | --- | --- | --- | --- | --- |
| 91.1 | gi|347828924|emb|CCD44621.1| | 25 | 140173 | 7 (1) | 5 (1) | 0.02 |  |
|  | hypothetical protein BofuT4\_P055220.1 [Botrytis cinerea T4] | | | | | | |

|  |  | Score | Mass | Matches | Sequences | emPAI |  |
| --- | --- | --- | --- | --- | --- | --- | --- |
| 91.1 | gi|347828924|emb|CCD44621.1| | 25 | 140173 | 7 (1) | 5 (1) | 0.02 | hypothetical protein BofuT4\_P055220.1 [Botrytis cinerea T4] |
|  |  | | | | | | |

#### 7 peptide matches (7 non-duplicate, 0 duplicate)

Auto-fit to window

| Query | Dupes | Observed | Mr(expt) | Mr(calc) | ppm | M | Score | Expect | Rank | U | Peptide |
| --- | --- | --- | --- | --- | --- | --- | --- | --- | --- | --- | --- |

| Query | Dupes | Observed | Mr(expt) | Mr(calc) | ppm | M | Score | Expect | Rank | U | Peptide |
| --- | --- | --- | --- | --- | --- | --- | --- | --- | --- | --- | --- |
| 770 |  | 436.7453 | 871.4760 | 871.5014 | -29.2 | 0 | 13 | 0.32 | 1Score **> 33** indicates **identity** Score **> 21** indicates **homology** | U | K.QLEILQK.S + Deamidated (NQ) |
| 3175 |  | 572.3174 | 1142.6202 | 1142.6156 | 4.06 | 2 | 9 | 1.1 | 2Score **> 33** indicates **identity** Score **> 22** indicates **homology** | U | R.RQLNQQRAK.A + 2 Deamidated (NQ) |
| 3229 |  | 575.7993 | 1149.5840 | 1149.5699 | 12.2 | 1 | 6 | 1.5 | 6Score **> 34** indicates **identity** Score **> 20** indicates **homology** |  | K.EAMAKVDAATK.D + Oxidation (M) |
| 5165 |  | 658.3696 | 1314.7246 | 1314.7394 | -11.3 | 1 | 25 | 0.032 | 1Score **> 33** indicates **identity** Score **> 22** indicates **homology** | U | R.ETILKENVELK.K |
| 5166 |  | 439.2494 | 1314.7263 | 1314.7394 | -10.0 | 1 | 4 | 1 | 1Score **> 33** indicates **identity** Score **> 17** indicates **homology** | U | R.ETILKENVELK.K |
| 5185 |  | 658.8622 | 1315.7098 | 1315.7235 | -10.4 | 1 | 4 | 3.8 | 8Score **> 34** indicates **identity** Score **> 23** indicates **homology** | U | R.ETILKENVELK.K + Deamidated (NQ) |
| 17243 |  | 975.8124 | 2924.4155 | 2924.4558 | -13.8 | 2 | 0 | 0.96 | 1Score **> 35** indicates **identity** Score **> 13** indicates **homology** | U | R.QLNQQRAKASAQTIQMLMGFMILQR.A + 4 Deamidated (NQ); Oxidation (M) |

---

### 92

|  |  | Accession | Score | Description |
| --- | --- | --- | --- | --- |
|  | 1 | gi|154697379|gb|EDN97117.1| | 25 | hypothetical protein SS1G\_02045 [Sclerotinia sclerotiorum 1980 UF-70] |

|  |  | Score | Mass | Matches | Sequences | emPAI |  |
| --- | --- | --- | --- | --- | --- | --- | --- |
| 92.1 | gi|154697379|gb|EDN97117.1| | 25 | 9864 | 6 (2) | 3 (1) | 0.35 |  |
|  | hypothetical protein SS1G\_02045 [Sclerotinia sclerotiorum 1980 UF-70] | | | | | | |
|  | 1 sameset of gi|154697379|gb|EDN97117.1| | | | | | | |
|  | gi|156063854|ref|XP\_001597849.1| | 25 | 9864 | 6 (2) | 3 (1) | 0.35 |  |
|  | hypothetical protein SS1G\_02045 [Sclerotinia sclerotiorum 1980 UF-70] | | | | | | |

|  |  | Score | Mass | Matches | Sequences | emPAI |  |
| --- | --- | --- | --- | --- | --- | --- | --- |
| 92.1 | gi|154697379|gb|EDN97117.1| | 25 | 9864 | 6 (2) | 3 (1) | 0.35 | hypothetical protein SS1G\_02045 [Sclerotinia sclerotiorum 1980 UF-70] |
|  |  | | | | | | |
|  | 1 sameset of gi|154697379|gb|EDN97117.1| | | | | | | |
|  | gi|156063854|ref|XP\_001597849.1| | 25 | 9864 | 6 (2) | 3 (1) | 0.35 | hypothetical protein SS1G\_02045 [Sclerotinia sclerotiorum 1980 UF-70] |
|  |  | | | | | | |

#### 6 peptide matches (3 non-duplicate, 3 duplicate)

Auto-fit to window

| Query | Dupes | Observed | Mr(expt) | Mr(calc) | ppm | M | Score | Expect | Rank | U | Peptide |
| --- | --- | --- | --- | --- | --- | --- | --- | --- | --- | --- | --- |

| Query | Dupes | Observed | Mr(expt) | Mr(calc) | ppm | M | Score | Expect | Rank | U | Peptide |
| --- | --- | --- | --- | --- | --- | --- | --- | --- | --- | --- | --- |
| 1434 |  | 481.7563 | 961.4981 | 961.4828 | 15.8 | 1 | 3 | 3 | 9Score **> 34** indicates **identity** Score **> 21** indicates **homology** |  | M.QTTSKEAR.I + Acetyl (Protein N-term) |
| 1466 |  | 483.7779 | 965.5412 | 965.5559 | -15.3 | 1 | 2 | 4.1 | 6Score **> 30** indicates **identity** Score **> 21** indicates **homology** |  | K.VGKLWAHR.F |
| 4727 | 3 | 639.3592 | 1276.7039 | 1276.6914 | 9.82 | 0 | 27 | 0.065 | 1Score **> 32** indicates **identity** Score **> 28** indicates **homology** | U | R.INLAIEAIYEK.G + Deamidated (NQ) |

---

### 93

|  |  | Accession | Score | Description |
| --- | --- | --- | --- | --- |
|  | 1 | gi|154701688|gb|EDO01427.1| | 24 | predicted protein [Sclerotinia sclerotiorum 1980 UF-70] |

|  |  | Score | Mass | Matches | Sequences | emPAI |  |
| --- | --- | --- | --- | --- | --- | --- | --- |
| 93.1 | gi|154701688|gb|EDO01427.1| | 24 | 78212 | 8 (1) | 3 (1) | 0.04 |  |
|  | predicted protein [Sclerotinia sclerotiorum 1980 UF-70] | | | | | | |
|  | 2 samesets of gi|154701688|gb|EDO01427.1| | | | | | | |
|  | gi|156059778|ref|XP\_001595812.1| | 24 | 78212 | 8 (1) | 3 (1) | 0.04 |  |
|  | predicted protein [Sclerotinia sclerotiorum 1980 UF-70] | | | | | | |
|  | gi|1095455532|gb|APA12488.1| | 24 | 78212 | 8 (1) | 3 (1) | 0.04 |  |
|  | hypothetical protein sscle\_09g072580 [Sclerotinia sclerotiorum 1980 UF-70] | | | | | | |

|  |  | Score | Mass | Matches | Sequences | emPAI |  |
| --- | --- | --- | --- | --- | --- | --- | --- |
| 93.1 | gi|154701688|gb|EDO01427.1| | 24 | 78212 | 8 (1) | 3 (1) | 0.04 | predicted protein [Sclerotinia sclerotiorum 1980 UF-70] |
|  |  | | | | | | |
|  | 2 samesets of gi|154701688|gb|EDO01427.1| | | | | | | |
|  | gi|156059778|ref|XP\_001595812.1| | 24 | 78212 | 8 (1) | 3 (1) | 0.04 | predicted protein [Sclerotinia sclerotiorum 1980 UF-70] |
|  |  | | | | | | |
|  | gi|1095455532|gb|APA12488.1| | 24 | 78212 | 8 (1) | 3 (1) | 0.04 | hypothetical protein sscle\_09g072580 [Sclerotinia sclerotiorum 1980 UF-70] |
|  |  | | | | | | |

#### 8 peptide matches (3 non-duplicate, 5 duplicate)

Auto-fit to window

| Query | Dupes | Observed | Mr(expt) | Mr(calc) | ppm | M | Score | Expect | Rank | U | Peptide |
| --- | --- | --- | --- | --- | --- | --- | --- | --- | --- | --- | --- |

| Query | Dupes | Observed | Mr(expt) | Mr(calc) | ppm | M | Score | Expect | Rank | U | Peptide |
| --- | --- | --- | --- | --- | --- | --- | --- | --- | --- | --- | --- |
| 509 | 5 | 418.7527 | 835.4909 | 835.4916 | -0.84 | 0 | 24 | 0.023 | 1Score **> 27** indicates **identity** Score **> 20** indicates **homology** | U | R.VPAPVTPR.L |
| 713 |  | 433.2555 | 864.4964 | 864.4705 | 30.0 | 0 | 13 | 1.7 | 2Score **> 29** indicates **identity** Score **> 28** indicates **homology** | U | K.ISIQYNK.T |
| 16245 |  | 945.4890 | 2833.4452 | 2833.3758 | 24.5 | 1 | 2 | 1 | 1Score **> 35** indicates **identity** Score **> 14** indicates **homology** | U | K.WPPLTSENSLYHPIQGRGDPMPWR.K |

---

### 94

|  |  | Accession | Score | Description |
| --- | --- | --- | --- | --- |
|  | 1 | gi|154693594|gb|EDN93332.1| | 24 | predicted protein [Sclerotinia sclerotiorum 1980 UF-70] |

|  |  | Score | Mass | Matches | Sequences | emPAI |  |
| --- | --- | --- | --- | --- | --- | --- | --- |
| 94.1 | gi|154693594|gb|EDN93332.1| | 24 | 13747 | 1 (1) | 1 (1) | 0.25 |  |
|  | predicted protein [Sclerotinia sclerotiorum 1980 UF-70] | | | | | | |
|  | 1 sameset of gi|154693594|gb|EDN93332.1| | | | | | | |
|  | gi|156045844|ref|XP\_001589477.1| | 24 | 13747 | 1 (1) | 1 (1) | 0.25 |  |
|  | predicted protein [Sclerotinia sclerotiorum 1980 UF-70] | | | | | | |

|  |  | Score | Mass | Matches | Sequences | emPAI |  |
| --- | --- | --- | --- | --- | --- | --- | --- |
| 94.1 | gi|154693594|gb|EDN93332.1| | 24 | 13747 | 1 (1) | 1 (1) | 0.25 | predicted protein [Sclerotinia sclerotiorum 1980 UF-70] |
|  |  | | | | | | |
|  | 1 sameset of gi|154693594|gb|EDN93332.1| | | | | | | |
|  | gi|156045844|ref|XP\_001589477.1| | 24 | 13747 | 1 (1) | 1 (1) | 0.25 | predicted protein [Sclerotinia sclerotiorum 1980 UF-70] |
|  |  | | | | | | |

#### 1 peptide matches (1 non-duplicate, 0 duplicate)

Auto-fit to window

| Query | Dupes | Observed | Mr(expt) | Mr(calc) | ppm | M | Score | Expect | Rank | U | Peptide |
| --- | --- | --- | --- | --- | --- | --- | --- | --- | --- | --- | --- |

| Query | Dupes | Observed | Mr(expt) | Mr(calc) | ppm | M | Score | Expect | Rank | U | Peptide |
| --- | --- | --- | --- | --- | --- | --- | --- | --- | --- | --- | --- |
| 8413 |  | 838.9204 | 1675.8263 | 1675.8199 | 3.79 | 1 | 24 | 0.0072 | 1Score **> 35** indicates **identity** Score **> 15** indicates **homology** | U | R.MGSQNPTTIKNQVNK.L + Deamidated (NQ); Oxidation (M) |

---

### 95

|  |  | Accession | Score | Description |
| --- | --- | --- | --- | --- |
|  | 1 | gi|205829274|sp|A6S9N4.1|PAN1\_BOTFB | 23 | RecName: Full=Actin cytoskeleton-regulatory complex protein pan1 |

|  |  | Score | Mass | Matches | Sequences | emPAI |  |
| --- | --- | --- | --- | --- | --- | --- | --- |
| 95.1 | gi|205829274|sp|A6S9N4.1|PAN1\_BOTFB | 23 | 155861 | 10 (1) | 4 (1) | 0.02 |  |
|  | RecName: Full=Actin cytoskeleton-regulatory complex protein pan1 | | | | | | |
|  | 1 sameset of gi|205829274|sp|A6S9N4.1|PAN1\_BOTFB | | | | | | |
|  | gi|347841674|emb|CCD56246.1| | 23 | 155843 | 10 (1) | 4 (1) | 0.02 |  |
|  | hypothetical protein BofuT4\_P148560.1 [Botrytis cinerea T4] | | | | | | |

|  |  | Score | Mass | Matches | Sequences | emPAI |  |
| --- | --- | --- | --- | --- | --- | --- | --- |
| 95.1 | gi|205829274|sp|A6S9N4.1|PAN1\_BOTFB | 23 | 155861 | 10 (1) | 4 (1) | 0.02 | RecName: Full=Actin cytoskeleton-regulatory complex protein pan1 |
|  |  | | | | | | |
|  | 1 sameset of gi|205829274|sp|A6S9N4.1|PAN1\_BOTFB | | | | | | |
|  | gi|347841674|emb|CCD56246.1| | 23 | 155843 | 10 (1) | 4 (1) | 0.02 | hypothetical protein BofuT4\_P148560.1 [Botrytis cinerea T4] |
|  |  | | | | | | |

#### 10 peptide matches (4 non-duplicate, 6 duplicate)

Auto-fit to window

| Query | Dupes | Observed | Mr(expt) | Mr(calc) | ppm | M | Score | Expect | Rank | U | Peptide |
| --- | --- | --- | --- | --- | --- | --- | --- | --- | --- | --- | --- |

| Query | Dupes | Observed | Mr(expt) | Mr(calc) | ppm | M | Score | Expect | Rank | U | Peptide |
| --- | --- | --- | --- | --- | --- | --- | --- | --- | --- | --- | --- |
| 403 |  | 408.7505 | 815.4865 | 815.4726 | 17.1 | 2 | 8 | 2.3 | 7Score **> 32** indicates **identity** Score **> 24** indicates **homology** |  | R.VRRTER.S |
| 2396 |  | 537.2827 | 1072.5509 | 1072.5512 | -0.35 | 1 | 23 | 0.051 | 1Score **> 33** indicates **identity** Score **> 23** indicates **homology** | U | R.QEAELAKER.E |
| 3229 | 2 | 575.7993 | 1149.5840 | 1149.6029 | -16.5 | 1 | 7 | 1.1 | 2Score **> 34** indicates **identity** Score **> 20** indicates **homology** | U | R.AAYIKQQAEK.R + Deamidated (NQ) |
| 3495 | 4 | 586.2965 | 1170.5785 | 1170.6105 | -27.4 | 1 | 8 | 0.2 | 1Score **> 32** indicates **identity** Score **> 14** indicates **homology** |  | K.QQEARLAAQR.A + Deamidated (NQ) |

---

### 96

|  |  | Accession | Score | Description |
| --- | --- | --- | --- | --- |
|  | 1 | gi|8515841|gb|AAF76202.1|AF271387\_1 | 23 | aspartyl protease [Sclerotinia sclerotiorum] |

|  |  | Score | Mass | Matches | Sequences | emPAI |  |
| --- | --- | --- | --- | --- | --- | --- | --- |
| 96.1 | gi|8515841|gb|AAF76202.1|AF271387\_1 | 23 | 45417 | 4 (2) | 3 (2) | 0.15 |  |
|  | aspartyl protease [Sclerotinia sclerotiorum] | | | | | | |
|  | 7 samesets of gi|8515841|gb|AAF76202.1|AF271387\_1 | | | | | | |
|  | gi|154701416|gb|EDO01155.1| | 23 | 44889 | 3 (2) | 2 (2) | 0.15 |  |
|  | aspartyl protease [Sclerotinia sclerotiorum 1980 UF-70] | | | | | | |
|  | gi|156059234|ref|XP\_001595540.1| | 23 | 44889 | 3 (2) | 2 (2) | 0.15 |  |
|  | aspartyl protease [Sclerotinia sclerotiorum 1980 UF-70] | | | | | | |
|  | gi|374094078|gb|AEY84361.1| | 23 | 36850 | 3 (2) | 2 (2) | 0.19 |  |
|  | aspartyl protease, partial [Sclerotinia sclerotiorum] | | | | | | |
|  | gi|374094080|gb|AEY84362.1| | 23 | 36850 | 3 (2) | 2 (2) | 0.19 |  |
|  | aspartyl protease, partial [Sclerotinia sclerotiorum] | | | | | | |
|  | gi|374094082|gb|AEY84363.1| | 23 | 36850 | 3 (2) | 2 (2) | 0.19 |  |
|  | aspartyl protease, partial [Sclerotinia sclerotiorum] | | | | | | |
|  | gi|374094084|gb|AEY84364.1| | 23 | 36850 | 3 (2) | 2 (2) | 0.19 |  |
|  | aspartyl protease, partial [Sclerotinia sclerotiorum] | | | | | | |
|  | gi|1095455745|gb|APA12701.1| | 23 | 44889 | 3 (2) | 2 (2) | 0.15 |  |
|  | hypothetical protein sscle\_09g074710 [Sclerotinia sclerotiorum 1980 UF-70] | | | | | | |

|  |  | Score | Mass | Matches | Sequences | emPAI |  |
| --- | --- | --- | --- | --- | --- | --- | --- |
| 96.1 | gi|8515841|gb|AAF76202.1|AF271387\_1 | 23 | 45417 | 4 (2) | 3 (2) | 0.15 | aspartyl protease [Sclerotinia sclerotiorum] |
|  |  | | | | | | |
|  | 7 samesets of gi|8515841|gb|AAF76202.1|AF271387\_1 | | | | | | |
|  | gi|154701416|gb|EDO01155.1| | 23 | 44889 | 3 (2) | 2 (2) | 0.15 | aspartyl protease [Sclerotinia sclerotiorum 1980 UF-70] |
|  |  | | | | | | |
|  | gi|156059234|ref|XP\_001595540.1| | 23 | 44889 | 3 (2) | 2 (2) | 0.15 | aspartyl protease [Sclerotinia sclerotiorum 1980 UF-70] |
|  |  | | | | | | |
|  | gi|374094078|gb|AEY84361.1| | 23 | 36850 | 3 (2) | 2 (2) | 0.19 | aspartyl protease, partial [Sclerotinia sclerotiorum] |
|  |  | | | | | | |
|  | gi|374094080|gb|AEY84362.1| | 23 | 36850 | 3 (2) | 2 (2) | 0.19 | aspartyl protease, partial [Sclerotinia sclerotiorum] |
|  |  | | | | | | |
|  | gi|374094082|gb|AEY84363.1| | 23 | 36850 | 3 (2) | 2 (2) | 0.19 | aspartyl protease, partial [Sclerotinia sclerotiorum] |
|  |  | | | | | | |
|  | gi|374094084|gb|AEY84364.1| | 23 | 36850 | 3 (2) | 2 (2) | 0.19 | aspartyl protease, partial [Sclerotinia sclerotiorum] |
|  |  | | | | | | |
|  | gi|1095455745|gb|APA12701.1| | 23 | 44889 | 3 (2) | 2 (2) | 0.15 | hypothetical protein sscle\_09g074710 [Sclerotinia sclerotiorum 1980 UF-70] |
|  |  | | | | | | |

#### 4 peptide matches (4 non-duplicate, 0 duplicate)

Auto-fit to window

| Query | Dupes | Observed | Mr(expt) | Mr(calc) | ppm | M | Score | Expect | Rank | U | Peptide |
| --- | --- | --- | --- | --- | --- | --- | --- | --- | --- | --- | --- |

| Query | Dupes | Observed | Mr(expt) | Mr(calc) | ppm | M | Score | Expect | Rank | U | Peptide |
| --- | --- | --- | --- | --- | --- | --- | --- | --- | --- | --- | --- |
| 2984 |  | 563.8333 | 1125.6519 | 1125.6506 | 1.21 | 0 | 22 | 0.047 | 1Score **> 29** indicates **identity** Score **> 21** indicates **homology** | U | K.LNTVKPAQQK.T |
| 13411 |  | 1182.5546 | 2363.0946 | 2363.0926 | 0.83 | 0 | 10 | 1 | 1Score **> 35** indicates **identity** Score **> 23** indicates **homology** | U | K.TFFDNAMSQGLAMPVFTADLR.K + 2 Oxidation (M) |
| 13412 |  | 788.7092 | 2363.1057 | 2363.0926 | 5.53 | 0 | 19 | 0.021 | 1Score **> 35** indicates **identity** Score **> 14** indicates **homology** | U | K.TFFDNAMSQGLAMPVFTADLR.K + 2 Oxidation (M) |
| 23337 |  | 1514.3477 | 4540.0212 | 4539.9952 | 5.71 | 0 | 2 | 1 | 3Score **> 33** indicates **identity** Score **> 14** indicates **homology** | U | K.MMQGANWSISYGDGSGAAGNVGTDTVNIGGATVTGQAIEMATAVSR.S + 3 Deamidated (NQ); Dioxidation (W); 2 Oxidation (M) |

---

### 97

|  |  | Accession | Score | Description |
| --- | --- | --- | --- | --- |
|  | 1 | gi|347837413|emb|CCD51985.1| | 23 | hypothetical protein BofuT4\_P081440.1 [Botrytis cinerea T4] |

|  |  | Score | Mass | Matches | Sequences | emPAI |  |
| --- | --- | --- | --- | --- | --- | --- | --- |
| 97.1 | gi|347837413|emb|CCD51985.1| | 23 | 74020 | 3 (1) | 2 (1) | 0.04 |  |
|  | hypothetical protein BofuT4\_P081440.1 [Botrytis cinerea T4] | | | | | | |
|  | 3 samesets of gi|347837413|emb|CCD51985.1| | | | | | | |
|  | gi|154692815|gb|EDN92553.1| | 23 | 74172 | 2 (1) | 2 (1) | 0.04 |  |
|  | hypothetical protein SS1G\_08416 [Sclerotinia sclerotiorum 1980 UF-70] | | | | | | |
|  | gi|156049419|ref|XP\_001590676.1| | 23 | 74172 | 2 (1) | 2 (1) | 0.04 |  |
|  | hypothetical protein SS1G\_08416 [Sclerotinia sclerotiorum 1980 UF-70] | | | | | | |
|  | gi|1095455979|gb|APA12934.1| | 23 | 72915 | 2 (1) | 2 (1) | 0.05 |  |
|  | hypothetical protein sscle\_10g077040 [Sclerotinia sclerotiorum 1980 UF-70] | | | | | | |

|  |  | Score | Mass | Matches | Sequences | emPAI |  |
| --- | --- | --- | --- | --- | --- | --- | --- |
| 97.1 | gi|347837413|emb|CCD51985.1| | 23 | 74020 | 3 (1) | 2 (1) | 0.04 | hypothetical protein BofuT4\_P081440.1 [Botrytis cinerea T4] |
|  |  | | | | | | |
|  | 3 samesets of gi|347837413|emb|CCD51985.1| | | | | | | |
|  | gi|154692815|gb|EDN92553.1| | 23 | 74172 | 2 (1) | 2 (1) | 0.04 | hypothetical protein SS1G\_08416 [Sclerotinia sclerotiorum 1980 UF-70] |
|  |  | | | | | | |
|  | gi|156049419|ref|XP\_001590676.1| | 23 | 74172 | 2 (1) | 2 (1) | 0.04 | hypothetical protein SS1G\_08416 [Sclerotinia sclerotiorum 1980 UF-70] |
|  |  | | | | | | |
|  | gi|1095455979|gb|APA12934.1| | 23 | 72915 | 2 (1) | 2 (1) | 0.05 | hypothetical protein sscle\_10g077040 [Sclerotinia sclerotiorum 1980 UF-70] |
|  |  | | | | | | |

#### 3 peptide matches (2 non-duplicate, 1 duplicate)

Auto-fit to window

| Query | Dupes | Observed | Mr(expt) | Mr(calc) | ppm | M | Score | Expect | Rank | U | Peptide |
| --- | --- | --- | --- | --- | --- | --- | --- | --- | --- | --- | --- |

| Query | Dupes | Observed | Mr(expt) | Mr(calc) | ppm | M | Score | Expect | Rank | U | Peptide |
| --- | --- | --- | --- | --- | --- | --- | --- | --- | --- | --- | --- |
| 4596 |  | 632.3330 | 1262.6515 | 1262.6731 | -17.2 | 0 | 23 | 0.015 | 1Score **> 34** indicates **identity** Score **> 17** indicates **homology** | U | K.GEAVVPHQALSR.V |
| 5562 | 1 | 679.3596 | 1356.7047 | 1356.7249 | -14.9 | 1 | 6 | 1.5 | 7Score **> 33** indicates **identity** Score **> 20** indicates **homology** | U | R.GAQEVVQRVLEK.L + 2 Deamidated (NQ) |

---

### 98

|  |  | Accession | Score | Description |
| --- | --- | --- | --- | --- |
|  | 1 | gi|154701812|gb|EDO01551.1| | 22 | hypothetical protein SS1G\_04026 [Sclerotinia sclerotiorum 1980 UF-70] |

|  |  | Score | Mass | Matches | Sequences | emPAI |  |
| --- | --- | --- | --- | --- | --- | --- | --- |
| 98.1 | gi|154701812|gb|EDO01551.1| | 22 | 14598 | 3 (1) | 1 (1) | 0.23 |  |
|  | hypothetical protein SS1G\_04026 [Sclerotinia sclerotiorum 1980 UF-70] | | | | | | |
|  | 2 samesets of gi|154701812|gb|EDO01551.1| | | | | | | |
|  | gi|156056591|ref|XP\_001594219.1| | 22 | 14598 | 3 (1) | 1 (1) | 0.23 |  |
|  | hypothetical protein SS1G\_04026 [Sclerotinia sclerotiorum 1980 UF-70] | | | | | | |
|  | gi|1095450275|gb|APA07238.1| | 22 | 14598 | 3 (1) | 1 (1) | 0.23 |  |
|  | hypothetical protein sscle\_02g020080 [Sclerotinia sclerotiorum 1980 UF-70] | | | | | | |

|  |  | Score | Mass | Matches | Sequences | emPAI |  |
| --- | --- | --- | --- | --- | --- | --- | --- |
| 98.1 | gi|154701812|gb|EDO01551.1| | 22 | 14598 | 3 (1) | 1 (1) | 0.23 | hypothetical protein SS1G\_04026 [Sclerotinia sclerotiorum 1980 UF-70] |
|  |  | | | | | | |
|  | 2 samesets of gi|154701812|gb|EDO01551.1| | | | | | | |
|  | gi|156056591|ref|XP\_001594219.1| | 22 | 14598 | 3 (1) | 1 (1) | 0.23 | hypothetical protein SS1G\_04026 [Sclerotinia sclerotiorum 1980 UF-70] |
|  |  | | | | | | |
|  | gi|1095450275|gb|APA07238.1| | 22 | 14598 | 3 (1) | 1 (1) | 0.23 | hypothetical protein sscle\_02g020080 [Sclerotinia sclerotiorum 1980 UF-70] |
|  |  | | | | | | |

#### 3 peptide matches (1 non-duplicate, 2 duplicate)

Auto-fit to window

| Query | Dupes | Observed | Mr(expt) | Mr(calc) | ppm | M | Score | Expect | Rank | U | Peptide |
| --- | --- | --- | --- | --- | --- | --- | --- | --- | --- | --- | --- |

| Query | Dupes | Observed | Mr(expt) | Mr(calc) | ppm | M | Score | Expect | Rank | U | Peptide |
| --- | --- | --- | --- | --- | --- | --- | --- | --- | --- | --- | --- |
| 5489 | 2 | 674.8296 | 1347.6446 | 1347.6684 | -17.6 | 1 | 22 | 0.053 | 1Score **> 34** indicates **identity** Score **> 22** indicates **homology** | U | K.YHPAGDPRNPPK.E |

---

### 99

|  |  | Accession | Score | Description |
| --- | --- | --- | --- | --- |
|  | 1 | gi|189082538|sp|A6RIS1.2|MPH1\_BOTFB | 22 | RecName: Full=ATP-dependent DNA helicase MPH1; AltName: Full=FANCM-like protein 1 |

|  |  | Score | Mass | Matches | Sequences | emPAI |  |
| --- | --- | --- | --- | --- | --- | --- | --- |
| 99.1 | gi|189082538|sp|A6RIS1.2|MPH1\_BOTFB | 22 | 137188 | 4 (1) | 4 (1) | 0.02 |  |
|  | RecName: Full=ATP-dependent DNA helicase MPH1; AltName: Full=FANCM-like protein 1 | | | | | | |
|  | 1 sameset of gi|189082538|sp|A6RIS1.2|MPH1\_BOTFB | | | | | | |
|  | gi|347829953|emb|CCD45650.1| | 22 | 137188 | 4 (1) | 4 (1) | 0.02 |  |
|  | similar to gi|189082538|sp|A6RIS1.2|MPH1\_BOTFB RecName: Full=ATP-dependent DNA helicase mph1 [Botrytis cinerea T4] | | | | | | |

|  |  | Score | Mass | Matches | Sequences | emPAI |  |
| --- | --- | --- | --- | --- | --- | --- | --- |
| 99.1 | gi|189082538|sp|A6RIS1.2|MPH1\_BOTFB | 22 | 137188 | 4 (1) | 4 (1) | 0.02 | RecName: Full=ATP-dependent DNA helicase MPH1; AltName: Full=FANCM-like protein 1 |
|  |  | | | | | | |
|  | 1 sameset of gi|189082538|sp|A6RIS1.2|MPH1\_BOTFB | | | | | | |
|  | gi|347829953|emb|CCD45650.1| | 22 | 137188 | 4 (1) | 4 (1) | 0.02 | similar to gi|189082538|sp|A6RIS1.2|MPH1\_BOTFB RecName: Full=ATP-dependent DNA helicase mph1 [Botrytis cinerea T4] |
|  |  | | | | | | |

#### 4 peptide matches (4 non-duplicate, 0 duplicate)

Auto-fit to window

| Query | Dupes | Observed | Mr(expt) | Mr(calc) | ppm | M | Score | Expect | Rank | U | Peptide |
| --- | --- | --- | --- | --- | --- | --- | --- | --- | --- | --- | --- |

| Query | Dupes | Observed | Mr(expt) | Mr(calc) | ppm | M | Score | Expect | Rank | U | Peptide |
| --- | --- | --- | --- | --- | --- | --- | --- | --- | --- | --- | --- |
| 157 |  | 380.2216 | 758.4287 | 758.4286 | 0.070 | 0 | 29 | 0.19 | 1Score **> 34** indicates **identity** |  | K.QLETIR.K |
| 328 |  | 399.7690 | 797.5233 | 797.5235 | -0.21 | 2 | 2 | 7.6 | 10Score **> 24** indicates **identity** Score **> 23** indicates **homology** | U | K.ARKKPAK.K |
| 2486 |  | 542.2886 | 1082.5627 | 1082.5356 | 25.0 | 0 | 1 | 5.4 | 7Score **> 31** indicates **identity** Score **> 21** indicates **homology** | U | K.LNGNVAGPPSR.I + 2 Deamidated (NQ) |
| 7854 |  | 806.3768 | 1610.7391 | 1610.7545 | -9.55 | 1 | 22 | 0.012 | 1Score **> 33** indicates **identity** Score **> 15** indicates **homology** | U | K.TWMASGAGKSANMGVK.G + Oxidation (M) |

---

### 100

|  |  | Accession | Score | Description |
| --- | --- | --- | --- | --- |
|  | 1 | gi|154702860|gb|EDO02599.1| | 22 | predicted protein [Sclerotinia sclerotiorum 1980 UF-70] |

|  |  | Score | Mass | Matches | Sequences | emPAI |  |
| --- | --- | --- | --- | --- | --- | --- | --- |
| 100.1 | gi|154702860|gb|EDO02599.1| | 22 | 6219 | 4 (1) | 2 (1) | 0.58 |  |
|  | predicted protein [Sclerotinia sclerotiorum 1980 UF-70] | | | | | | |
|  | 1 sameset of gi|154702860|gb|EDO02599.1| | | | | | | |
|  | gi|156055448|ref|XP\_001593648.1| | 22 | 6219 | 4 (1) | 2 (1) | 0.58 |  |
|  | predicted protein [Sclerotinia sclerotiorum 1980 UF-70] | | | | | | |

|  |  | Score | Mass | Matches | Sequences | emPAI |  |
| --- | --- | --- | --- | --- | --- | --- | --- |
| 100.1 | gi|154702860|gb|EDO02599.1| | 22 | 6219 | 4 (1) | 2 (1) | 0.58 | predicted protein [Sclerotinia sclerotiorum 1980 UF-70] |
|  |  | | | | | | |
|  | 1 sameset of gi|154702860|gb|EDO02599.1| | | | | | | |
|  | gi|156055448|ref|XP\_001593648.1| | 22 | 6219 | 4 (1) | 2 (1) | 0.58 | predicted protein [Sclerotinia sclerotiorum 1980 UF-70] |
|  |  | | | | | | |

#### 4 peptide matches (2 non-duplicate, 2 duplicate)

Auto-fit to window

| Query | Dupes | Observed | Mr(expt) | Mr(calc) | ppm | M | Score | Expect | Rank | U | Peptide |
| --- | --- | --- | --- | --- | --- | --- | --- | --- | --- | --- | --- |

| Query | Dupes | Observed | Mr(expt) | Mr(calc) | ppm | M | Score | Expect | Rank | U | Peptide |
| --- | --- | --- | --- | --- | --- | --- | --- | --- | --- | --- | --- |
| 136 | 1 | 377.1894 | 752.3642 | 752.3752 | -14.5 | 1 | 7 | 0.27 | 1Score **> 30** indicates **identity** Score **> 14** indicates **homology** | U | K.QRYMR.T |
| 2185 | 1 | 526.7744 | 1051.5341 | 1051.5596 | -24.2 | 2 | 22 | 0.022 | 1Score **> 32** indicates **identity** Score **> 18** indicates **homology** | U | R.IGKQRYMR.T + Deamidated (NQ) |

---

### 101

|  |  | Accession | Score | Description |
| --- | --- | --- | --- | --- |
|  | 1 | gi|347830803|emb|CCD46500.1| | 22 | similar to transcription factor Zn, C2H2 [Botrytis cinerea T4] |

|  |  | Score | Mass | Matches | Sequences | emPAI |  |
| --- | --- | --- | --- | --- | --- | --- | --- |
| 101.1 | gi|347830803|emb|CCD46500.1| | 22 | 47463 | 3 (1) | 2 (1) | 0.07 |  |
|  | similar to transcription factor Zn, C2H2 [Botrytis cinerea T4] | | | | | | |

|  |  | Score | Mass | Matches | Sequences | emPAI |  |
| --- | --- | --- | --- | --- | --- | --- | --- |
| 101.1 | gi|347830803|emb|CCD46500.1| | 22 | 47463 | 3 (1) | 2 (1) | 0.07 | similar to transcription factor Zn, C2H2 [Botrytis cinerea T4] |
|  |  | | | | | | |

#### 3 peptide matches (2 non-duplicate, 1 duplicate)

Auto-fit to window

| Query | Dupes | Observed | Mr(expt) | Mr(calc) | ppm | M | Score | Expect | Rank | U | Peptide |
| --- | --- | --- | --- | --- | --- | --- | --- | --- | --- | --- | --- |

| Query | Dupes | Observed | Mr(expt) | Mr(calc) | ppm | M | Score | Expect | Rank | U | Peptide |
| --- | --- | --- | --- | --- | --- | --- | --- | --- | --- | --- | --- |
| 1836 |  | 507.2307 | 1012.4467 | 1012.4760 | -28.9 | 1 | 22 | 0.024 | 1Score **> 29** indicates **identity** Score **> 18** indicates **homology** | U | K.TCTKSFNR.K |
| 4427 | 1 | 625.8223 | 1249.6300 | 1249.6527 | -18.2 | 2 | 5 | 1.6 | 7Score **> 34** indicates **identity** Score **> 20** indicates **homology** |  | K.SFNRKSDLQR.H |

---

### 102

|  |  | Accession | Score | Description |
| --- | --- | --- | --- | --- |
|  | 1 | gi|154699920|gb|EDN99658.1| | 22 | hypothetical protein SS1G\_02516 [Sclerotinia sclerotiorum 1980 UF-70] |

|  |  | Score | Mass | Matches | Sequences | emPAI |  |
| --- | --- | --- | --- | --- | --- | --- | --- |
| 102.1 | gi|154699920|gb|EDN99658.1| | 22 | 17208 | 3 (1) | 2 (1) | 0.20 |  |
|  | hypothetical protein SS1G\_02516 [Sclerotinia sclerotiorum 1980 UF-70] | | | | | | |
|  | 1 sameset of gi|154699920|gb|EDN99658.1| | | | | | | |
|  | gi|156060747|ref|XP\_001596296.1| | 22 | 17208 | 3 (1) | 2 (1) | 0.20 |  |
|  | hypothetical protein SS1G\_02516 [Sclerotinia sclerotiorum 1980 UF-70] | | | | | | |

|  |  | Score | Mass | Matches | Sequences | emPAI |  |
| --- | --- | --- | --- | --- | --- | --- | --- |
| 102.1 | gi|154699920|gb|EDN99658.1| | 22 | 17208 | 3 (1) | 2 (1) | 0.20 | hypothetical protein SS1G\_02516 [Sclerotinia sclerotiorum 1980 UF-70] |
|  |  | | | | | | |
|  | 1 sameset of gi|154699920|gb|EDN99658.1| | | | | | | |
|  | gi|156060747|ref|XP\_001596296.1| | 22 | 17208 | 3 (1) | 2 (1) | 0.20 | hypothetical protein SS1G\_02516 [Sclerotinia sclerotiorum 1980 UF-70] |
|  |  | | | | | | |

#### 3 peptide matches (2 non-duplicate, 1 duplicate)

Auto-fit to window

| Query | Dupes | Observed | Mr(expt) | Mr(calc) | ppm | M | Score | Expect | Rank | U | Peptide |
| --- | --- | --- | --- | --- | --- | --- | --- | --- | --- | --- | --- |

| Query | Dupes | Observed | Mr(expt) | Mr(calc) | ppm | M | Score | Expect | Rank | U | Peptide |
| --- | --- | --- | --- | --- | --- | --- | --- | --- | --- | --- | --- |
| 1707 | 1 | 497.7654 | 993.5161 | 993.4913 | 25.0 | 1 | 26 | 0.082 | 1Score **> 32** indicates **identity** Score **> 28** indicates **homology** | U | K.SEAGLKSMR.A + Oxidation (M) |
| 3879 |  | 603.3091 | 1204.6036 | 1204.6346 | -25.7 | 2 | 4 | 4 | 6Score **> 34** indicates **identity** Score **> 22** indicates **homology** | U | K.SEAGLKSMRAR.A |

---

### 103

|  |  | Accession | Score | Description |
| --- | --- | --- | --- | --- |
|  | 1 | gi|347839278|emb|CCD53850.1| | 21 | similar to aspartic protease precursor (secreted protein) [Botrytis cinerea T4] |

|  |  | Score | Mass | Matches | Sequences | emPAI |  |
| --- | --- | --- | --- | --- | --- | --- | --- |
| 103.1 | gi|347839278|emb|CCD53850.1| | 21 | 39820 | 4 (1) | 1 (1) | 0.08 |  |
|  | similar to aspartic protease precursor (secreted protein) [Botrytis cinerea T4] | | | | | | |

|  |  | Score | Mass | Matches | Sequences | emPAI |  |
| --- | --- | --- | --- | --- | --- | --- | --- |
| 103.1 | gi|347839278|emb|CCD53850.1| | 21 | 39820 | 4 (1) | 1 (1) | 0.08 | similar to aspartic protease precursor (secreted protein) [Botrytis cinerea T4] |
|  |  | | | | | | |

#### 4 peptide matches (1 non-duplicate, 3 duplicate)

Auto-fit to window

| Query | Dupes | Observed | Mr(expt) | Mr(calc) | ppm | M | Score | Expect | Rank | U | Peptide |
| --- | --- | --- | --- | --- | --- | --- | --- | --- | --- | --- | --- |

| Query | Dupes | Observed | Mr(expt) | Mr(calc) | ppm | M | Score | Expect | Rank | U | Peptide |
| --- | --- | --- | --- | --- | --- | --- | --- | --- | --- | --- | --- |
| 324 | 3 | 399.7572 | 797.4999 | 797.5011 | -1.45 | 0 | 21 | 0.042 | 1Score **> 26** indicates **identity** Score **> 20** indicates **homology** | U | K.SAVLPLAK.H |

---

### 104

|  |  | Accession | Score | Description |
| --- | --- | --- | --- | --- |
|  | 1 | gi|1095450321|gb|APA07284.1| | 21 | hypothetical protein sscle\_02g020540 [Sclerotinia sclerotiorum 1980 UF-70] |

|  |  | Score | Mass | Matches | Sequences | emPAI |  |
| --- | --- | --- | --- | --- | --- | --- | --- |
| 104.1 | gi|1095450321|gb|APA07284.1| | 21 | 42432 | 7 (1) | 4 (1) | 0.08 |  |
|  | hypothetical protein sscle\_02g020540 [Sclerotinia sclerotiorum 1980 UF-70] | | | | | | |
|  | 3 samesets of gi|1095450321|gb|APA07284.1| | | | | | | |
|  | gi|154698573|gb|EDN98311.1| | 21 | 32759 | 5 (1) | 2 (1) | 0.10 |  |
|  | hypothetical protein SS1G\_13169 [Sclerotinia sclerotiorum 1980 UF-70] | | | | | | |
|  | gi|156035929|ref|XP\_001586076.1| | 21 | 32759 | 5 (1) | 2 (1) | 0.10 |  |
|  | hypothetical protein SS1G\_13169 [Sclerotinia sclerotiorum 1980 UF-70] | | | | | | |
|  | gi|347841961|emb|CCD56533.1| | 21 | 42928 | 4 (1) | 1 (1) | 0.08 |  |
|  | similar to SET domain-containing protein [Botrytis cinerea T4] | | | | | | |

|  |  | Score | Mass | Matches | Sequences | emPAI |  |
| --- | --- | --- | --- | --- | --- | --- | --- |
| 104.1 | gi|1095450321|gb|APA07284.1| | 21 | 42432 | 7 (1) | 4 (1) | 0.08 | hypothetical protein sscle\_02g020540 [Sclerotinia sclerotiorum 1980 UF-70] |
|  |  | | | | | | |
|  | 3 samesets of gi|1095450321|gb|APA07284.1| | | | | | | |
|  | gi|154698573|gb|EDN98311.1| | 21 | 32759 | 5 (1) | 2 (1) | 0.10 | hypothetical protein SS1G\_13169 [Sclerotinia sclerotiorum 1980 UF-70] |
|  |  | | | | | | |
|  | gi|156035929|ref|XP\_001586076.1| | 21 | 32759 | 5 (1) | 2 (1) | 0.10 | hypothetical protein SS1G\_13169 [Sclerotinia sclerotiorum 1980 UF-70] |
|  |  | | | | | | |
|  | gi|347841961|emb|CCD56533.1| | 21 | 42928 | 4 (1) | 1 (1) | 0.08 | similar to SET domain-containing protein [Botrytis cinerea T4] |
|  |  | | | | | | |

#### 7 peptide matches (4 non-duplicate, 3 duplicate)

Auto-fit to window

| Query | Dupes | Observed | Mr(expt) | Mr(calc) | ppm | M | Score | Expect | Rank | U | Peptide |
| --- | --- | --- | --- | --- | --- | --- | --- | --- | --- | --- | --- |

| Query | Dupes | Observed | Mr(expt) | Mr(calc) | ppm | M | Score | Expect | Rank | U | Peptide |
| --- | --- | --- | --- | --- | --- | --- | --- | --- | --- | --- | --- |
| 848 |  | 443.2432 | 884.4718 | 884.4967 | -28.2 | 1 | 3 | 9.3 | 8Score **> 29** indicates **identity** Score **> 25** indicates **homology** | U | K.SSKLQPPK.E + Deamidated (NQ) |
| 1518 |  | 486.7776 | 971.5406 | 971.5261 | 14.9 | 1 | 5 | 1.1 | 2Score **> 33** indicates **identity** Score **> 18** indicates **homology** | U | K.TRAGLNGQR.D |
| 4432 | 3 | 625.8409 | 1249.6673 | 1249.6554 | 9.55 | 0 | 25 | 0.089 | 1Score **> 33** indicates **identity** Score **> 27** indicates **homology** | U | R.AGYTLTASQPIK.E + Deamidated (NQ) |
| 5280 |  | 664.8812 | 1327.7479 | 1327.7320 | 12.0 | 2 | 1 | 2.9 | 7Score **> 32** indicates **identity** Score **> 18** indicates **homology** | U | R.INKTRAGLNGQR.D + Deamidated (NQ) |

---

### 105

|  |  | Accession | Score | Description |
| --- | --- | --- | --- | --- |
|  | 1 | gi|347829515|emb|CCD45212.1| | 19 | similar to serine/threonine-protein kinase ssp1 [Botrytis cinerea T4] |

|  |  | Score | Mass | Matches | Sequences | emPAI |  |
| --- | --- | --- | --- | --- | --- | --- | --- |
| 105.1 | gi|347829515|emb|CCD45212.1| | 19 | 135275 | 8 (1) | 4 (1) | 0.02 |  |
|  | similar to serine/threonine-protein kinase ssp1 [Botrytis cinerea T4] | | | | | | |

|  |  | Score | Mass | Matches | Sequences | emPAI |  |
| --- | --- | --- | --- | --- | --- | --- | --- |
| 105.1 | gi|347829515|emb|CCD45212.1| | 19 | 135275 | 8 (1) | 4 (1) | 0.02 | similar to serine/threonine-protein kinase ssp1 [Botrytis cinerea T4] |
|  |  | | | | | | |

#### 8 peptide matches (4 non-duplicate, 4 duplicate)

Auto-fit to window

| Query | Dupes | Observed | Mr(expt) | Mr(calc) | ppm | M | Score | Expect | Rank | U | Peptide |
| --- | --- | --- | --- | --- | --- | --- | --- | --- | --- | --- | --- |

| Query | Dupes | Observed | Mr(expt) | Mr(calc) | ppm | M | Score | Expect | Rank | U | Peptide |
| --- | --- | --- | --- | --- | --- | --- | --- | --- | --- | --- | --- |
| 275 |  | 395.2268 | 788.4391 | 788.4253 | 17.5 | 2 | 4 | 1.1 | 2Score **> 33** indicates **identity** Score **> 17** indicates **homology** | U | K.GEGSRKR.A |
| 720 | 4 | 434.2556 | 866.4966 | 866.4722 | 28.1 | 0 | 19 | 0.033 | 1Score **> 28** indicates **identity** Score **> 17** indicates **homology** | U | K.LAAVGSHGR.R |
| 1188 |  | 465.7482 | 929.4818 | 929.5043 | -24.1 | 1 | 5 | 7.1 | 6Score **> 33** indicates **identity** Score **> 26** indicates **homology** | U | R.ATSRAPTAR.S |
| 2377 |  | 358.2081 | 1071.6026 | 1071.5746 | 26.1 | 1 | 1 | 1.7 | 5Score **> 33** indicates **identity** Score **> 16** indicates **homology** |  | R.MLIKDPAER.I |

---

### 106

|  |  | Accession | Score | Description |
| --- | --- | --- | --- | --- |
|  | 1 | gi|154701425|gb|EDO01164.1| | 19 | predicted protein [Sclerotinia sclerotiorum 1980 UF-70] |

|  |  | Score | Mass | Matches | Sequences | emPAI |  |
| --- | --- | --- | --- | --- | --- | --- | --- |
| 106.1 | gi|154701425|gb|EDO01164.1| | 19 | 7646 | 4 (1) | 1 (1) | 0.46 |  |
|  | predicted protein [Sclerotinia sclerotiorum 1980 UF-70] | | | | | | |
|  | 1 sameset of gi|154701425|gb|EDO01164.1| | | | | | | |
|  | gi|156059252|ref|XP\_001595549.1| | 19 | 7646 | 4 (1) | 1 (1) | 0.46 |  |
|  | predicted protein [Sclerotinia sclerotiorum 1980 UF-70] | | | | | | |

|  |  | Score | Mass | Matches | Sequences | emPAI |  |
| --- | --- | --- | --- | --- | --- | --- | --- |
| 106.1 | gi|154701425|gb|EDO01164.1| | 19 | 7646 | 4 (1) | 1 (1) | 0.46 | predicted protein [Sclerotinia sclerotiorum 1980 UF-70] |
|  |  | | | | | | |
|  | 1 sameset of gi|154701425|gb|EDO01164.1| | | | | | | |
|  | gi|156059252|ref|XP\_001595549.1| | 19 | 7646 | 4 (1) | 1 (1) | 0.46 | predicted protein [Sclerotinia sclerotiorum 1980 UF-70] |
|  |  | | | | | | |

#### 4 peptide matches (1 non-duplicate, 3 duplicate)

Auto-fit to window

| Query | Dupes | Observed | Mr(expt) | Mr(calc) | ppm | M | Score | Expect | Rank | U | Peptide |
| --- | --- | --- | --- | --- | --- | --- | --- | --- | --- | --- | --- |

| Query | Dupes | Observed | Mr(expt) | Mr(calc) | ppm | M | Score | Expect | Rank | U | Peptide |
| --- | --- | --- | --- | --- | --- | --- | --- | --- | --- | --- | --- |
| 5589 | 3 | 680.3554 | 1358.6961 | 1358.7228 | -19.6 | 1 | 19 | 0.019 | 1Score **> 34** indicates **identity** Score **> 14** indicates **homology** | U | -.MVILQVKNDTR.E + Acetyl (Protein N-term); Deamidated (NQ) |

---

### 107

|  |  | Accession | Score | Description |
| --- | --- | --- | --- | --- |
|  | 1 | gi|347840830|emb|CCD55402.1| | 19 | similar to NADH-cytochrome b5 reductase [Botrytis cinerea T4] |

|  |  | Score | Mass | Matches | Sequences | emPAI |  |
| --- | --- | --- | --- | --- | --- | --- | --- |
| 107.1 | gi|347840830|emb|CCD55402.1| | 19 | 34166 | 1 (1) | 1 (1) | 0.10 |  |
|  | similar to NADH-cytochrome b5 reductase [Botrytis cinerea T4] | | | | | | |

|  |  | Score | Mass | Matches | Sequences | emPAI |  |
| --- | --- | --- | --- | --- | --- | --- | --- |
| 107.1 | gi|347840830|emb|CCD55402.1| | 19 | 34166 | 1 (1) | 1 (1) | 0.10 | similar to NADH-cytochrome b5 reductase [Botrytis cinerea T4] |
|  |  | | | | | | |

#### 1 peptide matches (1 non-duplicate, 0 duplicate)

Auto-fit to window

| Query | Dupes | Observed | Mr(expt) | Mr(calc) | ppm | M | Score | Expect | Rank | U | Peptide |
| --- | --- | --- | --- | --- | --- | --- | --- | --- | --- | --- | --- |

| Query | Dupes | Observed | Mr(expt) | Mr(calc) | ppm | M | Score | Expect | Rank | U | Peptide |
| --- | --- | --- | --- | --- | --- | --- | --- | --- | --- | --- | --- |
| 5514 |  | 676.8569 | 1351.6992 | 1351.6806 | 13.8 | 1 | 19 | 0.056 | 1Score **> 33** indicates **identity** Score **> 19** indicates **homology** | U | -.MSTAPANKFLSK.A + Acetyl (Protein N-term); Oxidation (M) |

---

### 108

|  |  | Accession | Score | Description |
| --- | --- | --- | --- | --- |
|  | 1 | gi|1095450298|gb|APA07261.1| | 18 | hypothetical protein sscle\_02g020310 [Sclerotinia sclerotiorum 1980 UF-70] |

|  |  | Score | Mass | Matches | Sequences | emPAI |  |
| --- | --- | --- | --- | --- | --- | --- | --- |
| 108.1 | gi|1095450298|gb|APA07261.1| | 18 | 87266 | 9 (1) | 6 (1) | 0.04 |  |
|  | hypothetical protein sscle\_02g020310 [Sclerotinia sclerotiorum 1980 UF-70] | | | | | | |
|  | 1 sameset of gi|1095450298|gb|APA07261.1| | | | | | | |
|  | gi|347840168|emb|CCD54740.1| | 18 | 87293 | 8 (1) | 5 (1) | 0.04 |  |
|  | similar to anaphase-promoting complex subunit Apc5 [Botrytis cinerea T4] | | | | | | |

|  |  | Score | Mass | Matches | Sequences | emPAI |  |
| --- | --- | --- | --- | --- | --- | --- | --- |
| 108.1 | gi|1095450298|gb|APA07261.1| | 18 | 87266 | 9 (1) | 6 (1) | 0.04 | hypothetical protein sscle\_02g020310 [Sclerotinia sclerotiorum 1980 UF-70] |
|  |  | | | | | | |
|  | 1 sameset of gi|1095450298|gb|APA07261.1| | | | | | | |
|  | gi|347840168|emb|CCD54740.1| | 18 | 87293 | 8 (1) | 5 (1) | 0.04 | similar to anaphase-promoting complex subunit Apc5 [Botrytis cinerea T4] |
|  |  | | | | | | |

#### 9 peptide matches (6 non-duplicate, 3 duplicate)

Auto-fit to window

| Query | Dupes | Observed | Mr(expt) | Mr(calc) | ppm | M | Score | Expect | Rank | U | Peptide |
| --- | --- | --- | --- | --- | --- | --- | --- | --- | --- | --- | --- |

| Query | Dupes | Observed | Mr(expt) | Mr(calc) | ppm | M | Score | Expect | Rank | U | Peptide |
| --- | --- | --- | --- | --- | --- | --- | --- | --- | --- | --- | --- |
| 799 |  | 440.2386 | 878.4626 | 878.4498 | 14.6 | 0 | 6 | 1.9 | 3Score **> 32** indicates **identity** Score **> 21** indicates **homology** |  | R.ADFGAALSK.L |
| 3768 | 1 | 598.7989 | 1195.5833 | 1195.5763 | 5.89 | 1 | 14 | 1.2 | 2Score **> 33** indicates **identity** Score **> 27** indicates **homology** |  | K.KAMVMMAVGDK.T + Oxidation (M) |
| 6932 |  | 753.3713 | 1504.7280 | 1504.7521 | -16.0 | 1 | 1 | 1.1 | 6Score **> 34** indicates **identity** Score **> 14** indicates **homology** |  | K.LEGLDSNSLRSWK.A + Deamidated (NQ) |
| 6999 | 1 | 757.9162 | 1513.8178 | 1513.8463 | -18.8 | 2 | 18 | 0.043 | 1Score **> 33** indicates **identity** Score **> 17** indicates **homology** | U | R.LNNLLAKSREDIK.K + Deamidated (NQ) |
| 11988 | 1 | 1074.4508 | 2146.8871 | 2146.9268 | -18.5 | 1 | 1 | 1 | 1Score **> 29** indicates **identity** Score **> 13** indicates **homology** |  | K.GMKSMMGPQTAMQSSLWGR.L + Dioxidation (W); 2 Oxidation (M) |
| 23746 |  | 1204.6296 | 4814.4895 | 4814.5437 | -11.3 | 2 | 1 | 1 | 8Score **> 32** indicates **identity** Score **> 13** indicates **homology** |  | R.AASIAWRARLMPALWAAMAAVANILVALSEFQAACQIVIAILPR.A + Deamidated (NQ); 2 Dioxidation (W); 2 Oxidation (M) |

---

### 109

|  |  | Accession | Score | Description |
| --- | --- | --- | --- | --- |
|  | 1 | gi|154703296|gb|EDO03035.1| | 18 | hypothetical protein SS1G\_05513 [Sclerotinia sclerotiorum 1980 UF-70] |

|  |  | Score | Mass | Matches | Sequences | emPAI |  |
| --- | --- | --- | --- | --- | --- | --- | --- |
| 109.1 | gi|154703296|gb|EDO03035.1| | 18 | 69187 | 4 (1) | 2 (1) | 0.05 |  |
|  | hypothetical protein SS1G\_05513 [Sclerotinia sclerotiorum 1980 UF-70] | | | | | | |
|  | 3 samesets of gi|154703296|gb|EDO03035.1| | | | | | | |
|  | gi|156056320|ref|XP\_001594084.1| | 18 | 69187 | 4 (1) | 2 (1) | 0.05 |  |
|  | hypothetical protein SS1G\_05513 [Sclerotinia sclerotiorum 1980 UF-70] | | | | | | |
|  | gi|171704394|sp|A7EJL9.1|LKHA4\_SCLS1 | 18 | 69187 | 4 (1) | 2 (1) | 0.05 |  |
|  | RecName: Full=Leukotriene A-4 hydrolase homolog; Short=LTA-4 hydrolase; AltName: Full=Leukotriene A(4) hydrolase | | | | | | |
|  | gi|1095454999|gb|APA11956.1| | 18 | 69187 | 4 (1) | 2 (1) | 0.05 |  |
|  | hypothetical protein sscle\_08g067260 [Sclerotinia sclerotiorum 1980 UF-70] | | | | | | |

|  |  | Score | Mass | Matches | Sequences | emPAI |  |
| --- | --- | --- | --- | --- | --- | --- | --- |
| 109.1 | gi|154703296|gb|EDO03035.1| | 18 | 69187 | 4 (1) | 2 (1) | 0.05 | hypothetical protein SS1G\_05513 [Sclerotinia sclerotiorum 1980 UF-70] |
|  |  | | | | | | |
|  | 3 samesets of gi|154703296|gb|EDO03035.1| | | | | | | |
|  | gi|156056320|ref|XP\_001594084.1| | 18 | 69187 | 4 (1) | 2 (1) | 0.05 | hypothetical protein SS1G\_05513 [Sclerotinia sclerotiorum 1980 UF-70] |
|  |  | | | | | | |
|  | gi|171704394|sp|A7EJL9.1|LKHA4\_SCLS1 | 18 | 69187 | 4 (1) | 2 (1) | 0.05 | RecName: Full=Leukotriene A-4 hydrolase homolog; Short=LTA-4 hydrolase; AltName: Full=Leukotriene A(4) hydrolase |
|  |  | | | | | | |
|  | gi|1095454999|gb|APA11956.1| | 18 | 69187 | 4 (1) | 2 (1) | 0.05 | hypothetical protein sscle\_08g067260 [Sclerotinia sclerotiorum 1980 UF-70] |
|  |  | | | | | | |

#### 4 peptide matches (2 non-duplicate, 2 duplicate)

Auto-fit to window

| Query | Dupes | Observed | Mr(expt) | Mr(calc) | ppm | M | Score | Expect | Rank | U | Peptide |
| --- | --- | --- | --- | --- | --- | --- | --- | --- | --- | --- | --- |

| Query | Dupes | Observed | Mr(expt) | Mr(calc) | ppm | M | Score | Expect | Rank | U | Peptide |
| --- | --- | --- | --- | --- | --- | --- | --- | --- | --- | --- | --- |
| 1660 | 1 | 494.7727 | 987.5309 | 987.5237 | 7.37 | 1 | 8 | 1.2 | 4Score **> 34** indicates **identity** Score **> 21** indicates **homology** | U | R.EQVEKDLK.E |
| 4079 | 1 | 611.7908 | 1221.5671 | 1221.5666 | 0.41 | 0 | 18 | 0.025 | 1Score **> 33** indicates **identity** Score **> 14** indicates **homology** | U | K.LFGEDHEFTK.L |

---

### 110

|  |  | Accession | Score | Description |
| --- | --- | --- | --- | --- |
|  | 1 | gi|347441684|emb|CCD34605.1| | 18 | similar to N-alpha-acetyltransferase 15 [Botrytis cinerea T4] |

|  |  | Score | Mass | Matches | Sequences | emPAI |  |
| --- | --- | --- | --- | --- | --- | --- | --- |
| 110.1 | gi|347441684|emb|CCD34605.1| | 18 | 84600 | 4 (1) | 3 (1) | 0.04 |  |
|  | similar to N-alpha-acetyltransferase 15 [Botrytis cinerea T4] | | | | | | |

|  |  | Score | Mass | Matches | Sequences | emPAI |  |
| --- | --- | --- | --- | --- | --- | --- | --- |
| 110.1 | gi|347441684|emb|CCD34605.1| | 18 | 84600 | 4 (1) | 3 (1) | 0.04 | similar to N-alpha-acetyltransferase 15 [Botrytis cinerea T4] |
|  |  | | | | | | |

#### 4 peptide matches (3 non-duplicate, 1 duplicate)

Auto-fit to window

| Query | Dupes | Observed | Mr(expt) | Mr(calc) | ppm | M | Score | Expect | Rank | U | Peptide |
| --- | --- | --- | --- | --- | --- | --- | --- | --- | --- | --- | --- |

| Query | Dupes | Observed | Mr(expt) | Mr(calc) | ppm | M | Score | Expect | Rank | U | Peptide |
| --- | --- | --- | --- | --- | --- | --- | --- | --- | --- | --- | --- |
| 682 |  | 430.2388 | 858.4630 | 858.4671 | -4.74 | 2 | 8 | 11 | 7Score **> 34** indicates **identity** Score **> 31** indicates **homology** |  | K.ARKEAER.L |
| 4114 |  | 409.2397 | 1224.6972 | 1224.7190 | -17.7 | 2 | 1 | 1.1 | 2Score **> 29** indicates **identity** Score **> 14** indicates **homology** |  | K.AADQILKKNPK.H |
| 9231 | 1 | 891.9085 | 1781.8023 | 1781.8175 | -8.51 | 1 | 18 | 0.03 | 1Score **> 33** indicates **identity** Score **> 15** indicates **homology** | U | K.CLMAASAIDKENESVK.E + Deamidated (NQ); Oxidation (M) |

---

### 111

|  |  | Accession | Score | Description |
| --- | --- | --- | --- | --- |
|  | 1 | gi|347831843|emb|CCD47540.1| | 18 | similar to NRPS-like enzyme [Botrytis cinerea T4] |

|  |  | Score | Mass | Matches | Sequences | emPAI |  |
| --- | --- | --- | --- | --- | --- | --- | --- |
| 111.1 | gi|347831843|emb|CCD47540.1| | 18 | 122530 | 7 (1) | 4 (1) | 0.03 |  |
|  | similar to NRPS-like enzyme [Botrytis cinerea T4] | | | | | | |

|  |  | Score | Mass | Matches | Sequences | emPAI |  |
| --- | --- | --- | --- | --- | --- | --- | --- |
| 111.1 | gi|347831843|emb|CCD47540.1| | 18 | 122530 | 7 (1) | 4 (1) | 0.03 | similar to NRPS-like enzyme [Botrytis cinerea T4] |
|  |  | | | | | | |

#### 7 peptide matches (4 non-duplicate, 3 duplicate)

Auto-fit to window

| Query | Dupes | Observed | Mr(expt) | Mr(calc) | ppm | M | Score | Expect | Rank | U | Peptide |
| --- | --- | --- | --- | --- | --- | --- | --- | --- | --- | --- | --- |

| Query | Dupes | Observed | Mr(expt) | Mr(calc) | ppm | M | Score | Expect | Rank | U | Peptide |
| --- | --- | --- | --- | --- | --- | --- | --- | --- | --- | --- | --- |
| 2085 |  | 521.3116 | 1040.6087 | 1040.6342 | -24.5 | 1 | 2 | 1 | 2Score **> 29** indicates **identity** Score **> 14** indicates **homology** | U | K.IQVKVNIAR.V + Deamidated (NQ) |
| 3884 | 1 | 603.3138 | 1204.6131 | 1204.6452 | -26.6 | 1 | 18 | 0.037 | 1Score **> 34** indicates **identity** Score **> 16** indicates **homology** | U | R.SRDLLTEFPK.D |
| 15736 | 1 | 926.4822 | 2776.4249 | 2776.3456 | 28.5 | 1 | 1 | 1 | 3Score **> 34** indicates **identity** Score **> 14** indicates **homology** | U | K.TFPDLQEYQTRDLFVPHSSKPNR.W + 2 Deamidated (NQ) |
| 16207 | 1 | 945.1604 | 2832.4594 | 2832.4129 | 16.4 | 2 | 2 | 1.1 | 7Score **> 34** indicates **identity** Score **> 15** indicates **homology** | U | R.RVTYKDFANAINGVAWCLHGNLGAGK.E + Deamidated (NQ) |

---

### 112

|  |  | Accession | Score | Description |
| --- | --- | --- | --- | --- |
|  | 1 | gi|154695791|gb|EDN95529.1| | 17 | hypothetical protein SS1G\_11407 [Sclerotinia sclerotiorum 1980 UF-70] |

|  |  | Score | Mass | Matches | Sequences | emPAI |  |
| --- | --- | --- | --- | --- | --- | --- | --- |
| 112.1 | gi|154695791|gb|EDN95529.1| | 17 | 36549 | 2 (1) | 2 (1) | 0.09 |  |
|  | hypothetical protein SS1G\_11407 [Sclerotinia sclerotiorum 1980 UF-70] | | | | | | |
|  | 1 sameset of gi|154695791|gb|EDN95529.1| | | | | | | |
|  | gi|156040858|ref|XP\_001587415.1| | 17 | 36549 | 2 (1) | 2 (1) | 0.09 |  |
|  | hypothetical protein SS1G\_11407 [Sclerotinia sclerotiorum 1980 UF-70] | | | | | | |

|  |  | Score | Mass | Matches | Sequences | emPAI |  |
| --- | --- | --- | --- | --- | --- | --- | --- |
| 112.1 | gi|154695791|gb|EDN95529.1| | 17 | 36549 | 2 (1) | 2 (1) | 0.09 | hypothetical protein SS1G\_11407 [Sclerotinia sclerotiorum 1980 UF-70] |
|  |  | | | | | | |
|  | 1 sameset of gi|154695791|gb|EDN95529.1| | | | | | | |
|  | gi|156040858|ref|XP\_001587415.1| | 17 | 36549 | 2 (1) | 2 (1) | 0.09 | hypothetical protein SS1G\_11407 [Sclerotinia sclerotiorum 1980 UF-70] |
|  |  | | | | | | |

#### 2 peptide matches (2 non-duplicate, 0 duplicate)

Auto-fit to window

| Query | Dupes | Observed | Mr(expt) | Mr(calc) | ppm | M | Score | Expect | Rank | U | Peptide |
| --- | --- | --- | --- | --- | --- | --- | --- | --- | --- | --- | --- |

| Query | Dupes | Observed | Mr(expt) | Mr(calc) | ppm | M | Score | Expect | Rank | U | Peptide |
| --- | --- | --- | --- | --- | --- | --- | --- | --- | --- | --- | --- |
| 6722 |  | 744.3549 | 1486.6953 | 1486.6576 | 25.4 | 0 | 17 | 0.034 | 1Score **> 33** indicates **identity** Score **> 15** indicates **homology** | U | K.NFGYIDDSISNNK.L + Deamidated (NQ) |
| 16239 |  | 945.4849 | 2833.4328 | 2833.3494 | 29.4 | 2 | 2 | 0.93 | 1Score **> 35** indicates **identity** Score **> 14** indicates **homology** | U | R.GGHSDWLTQFTKMPWSRSKPVPDK.E + Deamidated (NQ); Dioxidation (W); Oxidation (M) |

---

### 113

|  |  | Accession | Score | Description |
| --- | --- | --- | --- | --- |
|  | 1 | gi|154699123|gb|EDN98861.1| | 16 | hypothetical protein SS1G\_13720 [Sclerotinia sclerotiorum 1980 UF-70] |

|  |  | Score | Mass | Matches | Sequences | emPAI |  |
| --- | --- | --- | --- | --- | --- | --- | --- |
| 113.1 | gi|154699123|gb|EDN98861.1| | 16 | 55455 | 3 (1) | 1 (1) | 0.06 |  |
|  | hypothetical protein SS1G\_13720 [Sclerotinia sclerotiorum 1980 UF-70] | | | | | | |
|  | 3 samesets of gi|154699123|gb|EDN98861.1| | | | | | | |
|  | gi|156033289|ref|XP\_001585481.1| | 16 | 55455 | 3 (1) | 1 (1) | 0.06 |  |
|  | hypothetical protein SS1G\_13720 [Sclerotinia sclerotiorum 1980 UF-70] | | | | | | |
|  | gi|1095457997|gb|APA14948.1| | 16 | 59960 | 3 (1) | 1 (1) | 0.05 |  |
|  | hypothetical protein sscle\_14g097180 [Sclerotinia sclerotiorum 1980 UF-70] | | | | | | |
|  | gi|347840155|emb|CCD54727.1| | 16 | 59699 | 3 (1) | 1 (1) | 0.06 |  |
|  | similar to QUTD\_EMENI Quinate permease (Quinate transporter) [Botrytis cinerea T4] | | | | | | |

|  |  | Score | Mass | Matches | Sequences | emPAI |  |
| --- | --- | --- | --- | --- | --- | --- | --- |
| 113.1 | gi|154699123|gb|EDN98861.1| | 16 | 55455 | 3 (1) | 1 (1) | 0.06 | hypothetical protein SS1G\_13720 [Sclerotinia sclerotiorum 1980 UF-70] |
|  |  | | | | | | |
|  | 3 samesets of gi|154699123|gb|EDN98861.1| | | | | | | |
|  | gi|156033289|ref|XP\_001585481.1| | 16 | 55455 | 3 (1) | 1 (1) | 0.06 | hypothetical protein SS1G\_13720 [Sclerotinia sclerotiorum 1980 UF-70] |
|  |  | | | | | | |
|  | gi|1095457997|gb|APA14948.1| | 16 | 59960 | 3 (1) | 1 (1) | 0.05 | hypothetical protein sscle\_14g097180 [Sclerotinia sclerotiorum 1980 UF-70] |
|  |  | | | | | | |
|  | gi|347840155|emb|CCD54727.1| | 16 | 59699 | 3 (1) | 1 (1) | 0.06 | similar to QUTD\_EMENI Quinate permease (Quinate transporter) [Botrytis cinerea T4] |
|  |  | | | | | | |

#### 3 peptide matches (2 non-duplicate, 1 duplicate)

Auto-fit to window

| Query | Dupes | Observed | Mr(expt) | Mr(calc) | ppm | M | Score | Expect | Rank | U | Peptide |
| --- | --- | --- | --- | --- | --- | --- | --- | --- | --- | --- | --- |

| Query | Dupes | Observed | Mr(expt) | Mr(calc) | ppm | M | Score | Expect | Rank | U | Peptide |
| --- | --- | --- | --- | --- | --- | --- | --- | --- | --- | --- | --- |
| 13358 | 1 | 1177.0895 | 2352.1644 | 2352.1763 | -5.06 | 0 | 16 | 0.046 | 1Score **> 35** indicates **identity** Score **> 16** indicates **homology** | U | R.TLAQAHAAAHNWLWNFLISR.F + Deamidated (NQ); Dioxidation (W) |
| 13385 |  | 1177.5867 | 2353.1588 | 2353.1603 | -0.65 | 0 | 7 | 0.62 | 1Score **> 35** indicates **identity** Score **> 17** indicates **homology** | U | R.TLAQAHAAAHNWLWNFLISR.F + 2 Deamidated (NQ); Dioxidation (W) |

---

### 114

|  |  | Accession | Score | Description |
| --- | --- | --- | --- | --- |
|  | 1 | gi|347836371|emb|CCD50943.1| | 16 | hypothetical protein BofuT4\_P022110.1 [Botrytis cinerea T4] |

|  |  | Score | Mass | Matches | Sequences | emPAI |  |
| --- | --- | --- | --- | --- | --- | --- | --- |
| 114.1 | gi|347836371|emb|CCD50943.1| | 16 | 78508 | 26 (1) | 3 (1) | 0.04 |  |
|  | hypothetical protein BofuT4\_P022110.1 [Botrytis cinerea T4] | | | | | | |

|  |  | Score | Mass | Matches | Sequences | emPAI |  |
| --- | --- | --- | --- | --- | --- | --- | --- |
| 114.1 | gi|347836371|emb|CCD50943.1| | 16 | 78508 | 26 (1) | 3 (1) | 0.04 | hypothetical protein BofuT4\_P022110.1 [Botrytis cinerea T4] |
|  |  | | | | | | |

#### 26 peptide matches (5 non-duplicate, 21 duplicate)

Auto-fit to window

| Query | Dupes | Observed | Mr(expt) | Mr(calc) | ppm | M | Score | Expect | Rank | U | Peptide |
| --- | --- | --- | --- | --- | --- | --- | --- | --- | --- | --- | --- |

| Query | Dupes | Observed | Mr(expt) | Mr(calc) | ppm | M | Score | Expect | Rank | U | Peptide |
| --- | --- | --- | --- | --- | --- | --- | --- | --- | --- | --- | --- |
| 3137 | 1 | 570.7986 | 1139.5827 | 1139.5935 | -9.43 | 0 | 6 | 0.6 | 1Score **> 32** indicates **identity** Score **> 17** indicates **homology** | U | R.SDTGGLGAPIPR.S |
| 5108 |  | 655.8202 | 1309.6259 | 1309.6150 | 8.39 | 1 | 16 | 0.17 | 1Score **> 33** indicates **identity** Score **> 21** indicates **homology** |  | K.SGWASANKLNTK.Y + 2 Deamidated (NQ); Dioxidation (W) |
| 12833 |  | 1136.0399 | 2270.0653 | 2270.1138 | -21.4 | 1 | 7 | 1 | 1Score **> 35** indicates **identity** Score **> 20** indicates **homology** | U | R.TASPQIPSRSSSPQVNELQSR.F + 2 Deamidated (NQ) |
| 12834 | 17 | 1136.0399 | 2270.0653 | 2270.1138 | -21.4 | 1 | 16 | 0.046 | 1Score **> 35** indicates **identity** Score **> 15** indicates **homology** | U | R.TASPQIPSRSSSPQVNELQSR.F + 2 Deamidated (NQ) |
| 12836 | 3 | 1136.0403 | 2270.0660 | 2270.1138 | -21.1 | 1 | 10 | 0.14 | 1Score **> 35** indicates **identity** Score **> 14** indicates **homology** | U | R.TASPQIPSRSSSPQVNELQSR.F + 2 Deamidated (NQ) |

---

### 115

|  |  | Accession | Score | Description |
| --- | --- | --- | --- | --- |
|  | 1 | gi|154697025|gb|EDN96763.1| | 16 | hypothetical protein SS1G\_01689 [Sclerotinia sclerotiorum 1980 UF-70] |

|  |  | Score | Mass | Matches | Sequences | emPAI |  |
| --- | --- | --- | --- | --- | --- | --- | --- |
| 115.1 | gi|154697025|gb|EDN96763.1| | 16 | 33414 | 4 (1) | 4 (1) | 0.10 |  |
|  | hypothetical protein SS1G\_01689 [Sclerotinia sclerotiorum 1980 UF-70] | | | | | | |
|  | 3 samesets of gi|154697025|gb|EDN96763.1| | | | | | | |
|  | gi|156063146|ref|XP\_001597495.1| | 16 | 33414 | 4 (1) | 4 (1) | 0.10 |  |
|  | hypothetical protein SS1G\_01689 [Sclerotinia sclerotiorum 1980 UF-70] | | | | | | |
|  | gi|1095448951|gb|APA05915.1| | 16 | 47401 | 4 (1) | 4 (1) | 0.07 |  |
|  | hypothetical protein sscle\_01g006850 [Sclerotinia sclerotiorum 1980 UF-70] | | | | | | |
|  | gi|347838575|emb|CCD53147.1| | 16 | 48169 | 5 (1) | 4 (1) | 0.07 |  |
|  | hypothetical protein BofuT4\_P121660.1 [Botrytis cinerea T4] | | | | | | |

|  |  | Score | Mass | Matches | Sequences | emPAI |  |
| --- | --- | --- | --- | --- | --- | --- | --- |
| 115.1 | gi|154697025|gb|EDN96763.1| | 16 | 33414 | 4 (1) | 4 (1) | 0.10 | hypothetical protein SS1G\_01689 [Sclerotinia sclerotiorum 1980 UF-70] |
|  |  | | | | | | |
|  | 3 samesets of gi|154697025|gb|EDN96763.1| | | | | | | |
|  | gi|156063146|ref|XP\_001597495.1| | 16 | 33414 | 4 (1) | 4 (1) | 0.10 | hypothetical protein SS1G\_01689 [Sclerotinia sclerotiorum 1980 UF-70] |
|  |  | | | | | | |
|  | gi|1095448951|gb|APA05915.1| | 16 | 47401 | 4 (1) | 4 (1) | 0.07 | hypothetical protein sscle\_01g006850 [Sclerotinia sclerotiorum 1980 UF-70] |
|  |  | | | | | | |
|  | gi|347838575|emb|CCD53147.1| | 16 | 48169 | 5 (1) | 4 (1) | 0.07 | hypothetical protein BofuT4\_P121660.1 [Botrytis cinerea T4] |
|  |  | | | | | | |

#### 4 peptide matches (4 non-duplicate, 0 duplicate)

Auto-fit to window

| Query | Dupes | Observed | Mr(expt) | Mr(calc) | ppm | M | Score | Expect | Rank | U | Peptide |
| --- | --- | --- | --- | --- | --- | --- | --- | --- | --- | --- | --- |

| Query | Dupes | Observed | Mr(expt) | Mr(calc) | ppm | M | Score | Expect | Rank | U | Peptide |
| --- | --- | --- | --- | --- | --- | --- | --- | --- | --- | --- | --- |
| 985 |  | 451.7382 | 901.4618 | 901.4869 | -27.8 | 1 | 6 | 1.1 | 2Score **> 34** indicates **identity** Score **> 19** indicates **homology** | U | R.QLADAKEK.I |
| 3171 |  | 572.3160 | 1142.6174 | 1142.5931 | 21.3 | 0 | 14 | 1.3 | 2Score **> 33** indicates **identity** Score **> 28** indicates **homology** | U | R.LAEQQADQLK.A |
| 5212 |  | 440.5629 | 1318.6670 | 1318.6915 | -18.6 | 2 | 1 | 1.3 | 2Score **> 34** indicates **identity** Score **> 15** indicates **homology** | U | R.ADDEKITMLKR.L |
| 8217 |  | 830.3915 | 1658.7685 | 1658.7471 | 12.9 | 1 | 16 | 0.043 | 1Score **> 33** indicates **identity** Score **> 15** indicates **homology** | U | R.NNVMTNHWKDQEK.Q + Oxidation (M) |

---

### 116

|  |  | Accession | Score | Description |
| --- | --- | --- | --- | --- |
|  | 1 | gi|347842220|emb|CCD56792.1| | 16 | similar to carboxy-cis,cis-muconate cyclase [Botrytis cinerea T4] |

|  |  | Score | Mass | Matches | Sequences | emPAI |  |
| --- | --- | --- | --- | --- | --- | --- | --- |
| 116.1 | gi|347842220|emb|CCD56792.1| | 16 | 42864 | 2 (1) | 2 (1) | 0.08 |  |
|  | similar to carboxy-cis,cis-muconate cyclase [Botrytis cinerea T4] | | | | | | |
|  | 3 samesets of gi|347842220|emb|CCD56792.1| | | | | | | |
|  | gi|154700181|gb|EDN99919.1| | 16 | 40609 | 1 (1) | 1 (1) | 0.08 |  |
|  | hypothetical protein SS1G\_02777 [Sclerotinia sclerotiorum 1980 UF-70] | | | | | | |
|  | gi|156061269|ref|XP\_001596557.1| | 16 | 40609 | 1 (1) | 1 (1) | 0.08 |  |
|  | hypothetical protein SS1G\_02777 [Sclerotinia sclerotiorum 1980 UF-70] | | | | | | |
|  | gi|1095451980|gb|APA08941.1| | 16 | 40609 | 1 (1) | 1 (1) | 0.08 |  |
|  | hypothetical protein sscle\_04g037110 [Sclerotinia sclerotiorum 1980 UF-70] | | | | | | |

|  |  | Score | Mass | Matches | Sequences | emPAI |  |
| --- | --- | --- | --- | --- | --- | --- | --- |
| 116.1 | gi|347842220|emb|CCD56792.1| | 16 | 42864 | 2 (1) | 2 (1) | 0.08 | similar to carboxy-cis,cis-muconate cyclase [Botrytis cinerea T4] |
|  |  | | | | | | |
|  | 3 samesets of gi|347842220|emb|CCD56792.1| | | | | | | |
|  | gi|154700181|gb|EDN99919.1| | 16 | 40609 | 1 (1) | 1 (1) | 0.08 | hypothetical protein SS1G\_02777 [Sclerotinia sclerotiorum 1980 UF-70] |
|  |  | | | | | | |
|  | gi|156061269|ref|XP\_001596557.1| | 16 | 40609 | 1 (1) | 1 (1) | 0.08 | hypothetical protein SS1G\_02777 [Sclerotinia sclerotiorum 1980 UF-70] |
|  |  | | | | | | |
|  | gi|1095451980|gb|APA08941.1| | 16 | 40609 | 1 (1) | 1 (1) | 0.08 | hypothetical protein sscle\_04g037110 [Sclerotinia sclerotiorum 1980 UF-70] |
|  |  | | | | | | |

#### 2 peptide matches (2 non-duplicate, 0 duplicate)

Auto-fit to window

| Query | Dupes | Observed | Mr(expt) | Mr(calc) | ppm | M | Score | Expect | Rank | U | Peptide |
| --- | --- | --- | --- | --- | --- | --- | --- | --- | --- | --- | --- |

| Query | Dupes | Observed | Mr(expt) | Mr(calc) | ppm | M | Score | Expect | Rank | U | Peptide |
| --- | --- | --- | --- | --- | --- | --- | --- | --- | --- | --- | --- |
| 2811 |  | 556.2967 | 1110.5789 | 1110.5458 | 29.9 | 0 | 16 | 0.054 | 1Score **> 31** indicates **identity** Score **> 16** indicates **homology** | U | R.WVEIHPNGK.Y + Dioxidation (W) |
| 22803 |  | 808.5872 | 4037.8994 | 4037.8636 | 8.87 | 2 | 1 | 1.1 | 8Score **> 35** indicates **identity** Score **> 14** indicates **homology** | U | K.NIYGASMKKWSSHSVTSPAEIQHTTSHPMSHDPSASK.S + Deamidated (NQ); Oxidation (M) |

---

### 117

|  |  | Accession | Score | Description |
| --- | --- | --- | --- | --- |
|  | 1 | gi|154693945|gb|EDN93683.1| | 16 | hypothetical protein SS1G\_09550 [Sclerotinia sclerotiorum 1980 UF-70] |

|  |  | Score | Mass | Matches | Sequences | emPAI |  |
| --- | --- | --- | --- | --- | --- | --- | --- |
| 117.1 | gi|154693945|gb|EDN93683.1| | 16 | 171102 | 10 (1) | 4 (1) | 0.02 |  |
|  | hypothetical protein SS1G\_09550 [Sclerotinia sclerotiorum 1980 UF-70] | | | | | | |
|  | 2 samesets of gi|154693945|gb|EDN93683.1| | | | | | | |
|  | gi|156047721|ref|XP\_001589828.1| | 16 | 171102 | 10 (1) | 4 (1) | 0.02 |  |
|  | hypothetical protein SS1G\_09550 [Sclerotinia sclerotiorum 1980 UF-70] | | | | | | |
|  | gi|1095458688|gb|APA15638.1| | 16 | 171102 | 10 (1) | 4 (1) | 0.02 |  |
|  | hypothetical protein sscle\_15g104080 [Sclerotinia sclerotiorum 1980 UF-70] | | | | | | |

|  |  | Score | Mass | Matches | Sequences | emPAI |  |
| --- | --- | --- | --- | --- | --- | --- | --- |
| 117.1 | gi|154693945|gb|EDN93683.1| | 16 | 171102 | 10 (1) | 4 (1) | 0.02 | hypothetical protein SS1G\_09550 [Sclerotinia sclerotiorum 1980 UF-70] |
|  |  | | | | | | |
|  | 2 samesets of gi|154693945|gb|EDN93683.1| | | | | | | |
|  | gi|156047721|ref|XP\_001589828.1| | 16 | 171102 | 10 (1) | 4 (1) | 0.02 | hypothetical protein SS1G\_09550 [Sclerotinia sclerotiorum 1980 UF-70] |
|  |  | | | | | | |
|  | gi|1095458688|gb|APA15638.1| | 16 | 171102 | 10 (1) | 4 (1) | 0.02 | hypothetical protein sscle\_15g104080 [Sclerotinia sclerotiorum 1980 UF-70] |
|  |  | | | | | | |

#### 10 peptide matches (6 non-duplicate, 4 duplicate)

Auto-fit to window

| Query | Dupes | Observed | Mr(expt) | Mr(calc) | ppm | M | Score | Expect | Rank | U | Peptide |
| --- | --- | --- | --- | --- | --- | --- | --- | --- | --- | --- | --- |

| Query | Dupes | Observed | Mr(expt) | Mr(calc) | ppm | M | Score | Expect | Rank | U | Peptide |
| --- | --- | --- | --- | --- | --- | --- | --- | --- | --- | --- | --- |
| 795 |  | 440.2377 | 878.4608 | 878.4359 | 28.4 | 0 | 8 | 2.1 | 5Score **> 32** indicates **identity** Score **> 24** indicates **homology** | U | R.GEQGVPHR.S |
| 805 |  | 440.7300 | 879.4455 | 879.4199 | 29.1 | 0 | 1 | 2.6 | 7Score **> 33** indicates **identity** Score **> 17** indicates **homology** | U | R.GEQGVPHR.S + Deamidated (NQ) |
| 2754 |  | 554.2767 | 1106.5388 | 1106.5204 | 16.7 | 2 | 1 | 0.82 | 1Score **> 34** indicates **identity** Score **> 13** indicates **homology** | U | R.GKSQEKSTGAN.- + Deamidated (NQ) |
| 4326 | 4 | 621.3320 | 1240.6495 | 1240.6160 | 27.0 | 1 | 16 | 0.036 | 1Score **> 32** indicates **identity** Score **> 14** indicates **homology** | U | R.KTPGPAGNSNGNK.G |
| 5016 |  | 651.8534 | 1301.6922 | 1301.6762 | 12.3 | 0 | 1 | 7.9 | 6Score **> 35** indicates **identity** Score **> 22** indicates **homology** | U | R.LSQICQQVLGR.F + Deamidated (NQ) |
| 5036 |  | 652.8330 | 1303.6515 | 1303.6442 | 5.59 | 0 | 16 | 0.25 | 1Score **> 34** indicates **identity** Score **> 22** indicates **homology** | U | R.LSQICQQVLGR.F + 3 Deamidated (NQ) |

---

### 118

|  |  | Accession | Score | Description |
| --- | --- | --- | --- | --- |
|  | 1 | gi|347827291|emb|CCD42988.1| | 15 | hypothetical protein BofuT4\_P070640.1 [Botrytis cinerea T4] |

|  |  | Score | Mass | Matches | Sequences | emPAI |  |
| --- | --- | --- | --- | --- | --- | --- | --- |
| 118.1 | gi|347827291|emb|CCD42988.1| | 15 | 36355 | 3 (1) | 1 (1) | 0.09 |  |
|  | hypothetical protein BofuT4\_P070640.1 [Botrytis cinerea T4] | | | | | | |

|  |  | Score | Mass | Matches | Sequences | emPAI |  |
| --- | --- | --- | --- | --- | --- | --- | --- |
| 118.1 | gi|347827291|emb|CCD42988.1| | 15 | 36355 | 3 (1) | 1 (1) | 0.09 | hypothetical protein BofuT4\_P070640.1 [Botrytis cinerea T4] |
|  |  | | | | | | |

#### 3 peptide matches (2 non-duplicate, 1 duplicate)

Auto-fit to window

| Query | Dupes | Observed | Mr(expt) | Mr(calc) | ppm | M | Score | Expect | Rank | U | Peptide |
| --- | --- | --- | --- | --- | --- | --- | --- | --- | --- | --- | --- |

| Query | Dupes | Observed | Mr(expt) | Mr(calc) | ppm | M | Score | Expect | Rank | U | Peptide |
| --- | --- | --- | --- | --- | --- | --- | --- | --- | --- | --- | --- |
| 9586 |  | 911.9427 | 1821.8708 | 1821.8355 | 19.4 | 0 | 15 | 0.054 | 1Score **> 35** indicates **identity** Score **> 15** indicates **homology** | U | K.FANWVNNILNAQMQR.T + 4 Deamidated (NQ) |
| 9691 | 1 | 919.4128 | 1836.8111 | 1836.8464 | -19.2 | 0 | 11 | 0.53 | 1Score **> 32** indicates **identity** Score **> 20** indicates **homology** | U | K.FANWVNNILNAQMQR.T + 3 Deamidated (NQ); Oxidation (M) |

---

### 119

|  |  | Accession | Score | Description |
| --- | --- | --- | --- | --- |
|  | 1 | gi|347836979|emb|CCD51551.1| | 15 | hypothetical protein BofuT4\_P018570.1 [Botrytis cinerea T4] |

|  |  | Score | Mass | Matches | Sequences | emPAI |  |
| --- | --- | --- | --- | --- | --- | --- | --- |
| 119.1 | gi|347836979|emb|CCD51551.1| | 15 | 43057 | 8 (1) | 3 (1) | 0.08 |  |
|  | hypothetical protein BofuT4\_P018570.1 [Botrytis cinerea T4] | | | | | | |

|  |  | Score | Mass | Matches | Sequences | emPAI |  |
| --- | --- | --- | --- | --- | --- | --- | --- |
| 119.1 | gi|347836979|emb|CCD51551.1| | 15 | 43057 | 8 (1) | 3 (1) | 0.08 | hypothetical protein BofuT4\_P018570.1 [Botrytis cinerea T4] |
|  |  | | | | | | |

#### 8 peptide matches (3 non-duplicate, 5 duplicate)

Auto-fit to window

| Query | Dupes | Observed | Mr(expt) | Mr(calc) | ppm | M | Score | Expect | Rank | U | Peptide |
| --- | --- | --- | --- | --- | --- | --- | --- | --- | --- | --- | --- |

| Query | Dupes | Observed | Mr(expt) | Mr(calc) | ppm | M | Score | Expect | Rank | U | Peptide |
| --- | --- | --- | --- | --- | --- | --- | --- | --- | --- | --- | --- |
| 821 | 4 | 441.2639 | 880.5132 | 880.5242 | -12.5 | 1 | 7 | 1.1 | 2Score **> 27** indicates **identity** Score **> 20** indicates **homology** | U | R.RNKPPLR.L + Deamidated (NQ) |
| 1966 | 1 | 514.2468 | 1026.4790 | 1026.4923 | -13.0 | 0 | 4 | 1.1 | 2Score **> 30** indicates **identity** Score **> 16** indicates **homology** | U | K.NTFWNFAK.V |
| 4473 |  | 627.8068 | 1253.5991 | 1253.6186 | -15.6 | 1 | 15 | 0.037 | 1Score **> 33** indicates **identity** Score **> 14** indicates **homology** | U | K.DMHLVKNPQR.T + Deamidated (NQ); Oxidation (M) |

---

### 120

|  |  | Accession | Score | Description |
| --- | --- | --- | --- | --- |
|  | 1 | gi|347839918|emb|CCD54490.1| | 15 | similar to fatty acid desaturase [Botrytis cinerea T4] |

|  |  | Score | Mass | Matches | Sequences | emPAI |  |
| --- | --- | --- | --- | --- | --- | --- | --- |
| 120.1 | gi|347839918|emb|CCD54490.1| | 15 | 63394 | 6 (1) | 1 (1) | 0.05 |  |
|  | similar to fatty acid desaturase [Botrytis cinerea T4] | | | | | | |

|  |  | Score | Mass | Matches | Sequences | emPAI |  |
| --- | --- | --- | --- | --- | --- | --- | --- |
| 120.1 | gi|347839918|emb|CCD54490.1| | 15 | 63394 | 6 (1) | 1 (1) | 0.05 | similar to fatty acid desaturase [Botrytis cinerea T4] |
|  |  | | | | | | |

#### 6 peptide matches (2 non-duplicate, 4 duplicate)

Auto-fit to window

| Query | Dupes | Observed | Mr(expt) | Mr(calc) | ppm | M | Score | Expect | Rank | U | Peptide |
| --- | --- | --- | --- | --- | --- | --- | --- | --- | --- | --- | --- |

| Query | Dupes | Observed | Mr(expt) | Mr(calc) | ppm | M | Score | Expect | Rank | U | Peptide |
| --- | --- | --- | --- | --- | --- | --- | --- | --- | --- | --- | --- |
| 3559 | 2 | 589.8283 | 1177.6419 | 1177.6706 | -24.4 | 0 | 12 | 1.3 | 3Score **> 32** indicates **identity** Score **> 26** indicates **homology** | U | K.LSAYITEILR.C |
| 3565 | 2 | 393.5558 | 1177.6455 | 1177.6706 | -21.3 | 0 | 15 | 0.057 | 1Score **> 32** indicates **identity** Score **> 15** indicates **homology** | U | K.LSAYITEILR.C |

---

### 121

|  |  | Accession | Score | Description |
| --- | --- | --- | --- | --- |
|  | 1 | gi|154702172|gb|EDO01911.1| | 15 | predicted protein [Sclerotinia sclerotiorum 1980 UF-70] |

|  |  | Score | Mass | Matches | Sequences | emPAI |  |
| --- | --- | --- | --- | --- | --- | --- | --- |
| 121.1 | gi|154702172|gb|EDO01911.1| | 15 | 4498 | 2 (1) | 1 (1) | 0.81 |  |
|  | predicted protein [Sclerotinia sclerotiorum 1980 UF-70] | | | | | | |
|  | 1 sameset of gi|154702172|gb|EDO01911.1| | | | | | | |
|  | gi|156057311|ref|XP\_001594579.1| | 15 | 4498 | 2 (1) | 1 (1) | 0.81 |  |
|  | predicted protein [Sclerotinia sclerotiorum 1980 UF-70] | | | | | | |

|  |  | Score | Mass | Matches | Sequences | emPAI |  |
| --- | --- | --- | --- | --- | --- | --- | --- |
| 121.1 | gi|154702172|gb|EDO01911.1| | 15 | 4498 | 2 (1) | 1 (1) | 0.81 | predicted protein [Sclerotinia sclerotiorum 1980 UF-70] |
|  |  | | | | | | |
|  | 1 sameset of gi|154702172|gb|EDO01911.1| | | | | | | |
|  | gi|156057311|ref|XP\_001594579.1| | 15 | 4498 | 2 (1) | 1 (1) | 0.81 | predicted protein [Sclerotinia sclerotiorum 1980 UF-70] |
|  |  | | | | | | |

#### 2 peptide matches (1 non-duplicate, 1 duplicate)

Auto-fit to window

| Query | Dupes | Observed | Mr(expt) | Mr(calc) | ppm | M | Score | Expect | Rank | U | Peptide |
| --- | --- | --- | --- | --- | --- | --- | --- | --- | --- | --- | --- |

| Query | Dupes | Observed | Mr(expt) | Mr(calc) | ppm | M | Score | Expect | Rank | U | Peptide |
| --- | --- | --- | --- | --- | --- | --- | --- | --- | --- | --- | --- |
| 3963 | 1 | 606.3124 | 1210.6103 | 1210.6016 | 7.24 | 1 | 15 | 0.039 | 1Score **> 33** indicates **identity** Score **> 14** indicates **homology** | U | -.MKNNYATALK.E + Acetyl (Protein N-term); Oxidation (M) |

---

### 122

|  |  | Accession | Score | Description |
| --- | --- | --- | --- | --- |
|  | 1 | gi|154703482|gb|EDO03221.1| | 15 | hypothetical protein SS1G\_05701 [Sclerotinia sclerotiorum 1980 UF-70] |

|  |  | Score | Mass | Matches | Sequences | emPAI |  |
| --- | --- | --- | --- | --- | --- | --- | --- |
| 122.1 | gi|154703482|gb|EDO03221.1| | 15 | 86629 | 6 (1) | 3 (1) | 0.04 |  |
|  | hypothetical protein SS1G\_05701 [Sclerotinia sclerotiorum 1980 UF-70] | | | | | | |
|  | 2 samesets of gi|154703482|gb|EDO03221.1| | | | | | | |
|  | gi|156053708|ref|XP\_001592780.1| | 15 | 86629 | 6 (1) | 3 (1) | 0.04 |  |
|  | hypothetical protein SS1G\_05701 [Sclerotinia sclerotiorum 1980 UF-70] | | | | | | |
|  | gi|1095453054|gb|APA10014.1| | 15 | 92453 | 6 (1) | 3 (1) | 0.04 |  |
|  | hypothetical protein sscle\_05g047840 [Sclerotinia sclerotiorum 1980 UF-70] | | | | | | |

|  |  | Score | Mass | Matches | Sequences | emPAI |  |
| --- | --- | --- | --- | --- | --- | --- | --- |
| 122.1 | gi|154703482|gb|EDO03221.1| | 15 | 86629 | 6 (1) | 3 (1) | 0.04 | hypothetical protein SS1G\_05701 [Sclerotinia sclerotiorum 1980 UF-70] |
|  |  | | | | | | |
|  | 2 samesets of gi|154703482|gb|EDO03221.1| | | | | | | |
|  | gi|156053708|ref|XP\_001592780.1| | 15 | 86629 | 6 (1) | 3 (1) | 0.04 | hypothetical protein SS1G\_05701 [Sclerotinia sclerotiorum 1980 UF-70] |
|  |  | | | | | | |
|  | gi|1095453054|gb|APA10014.1| | 15 | 92453 | 6 (1) | 3 (1) | 0.04 | hypothetical protein sscle\_05g047840 [Sclerotinia sclerotiorum 1980 UF-70] |
|  |  | | | | | | |

#### 6 peptide matches (4 non-duplicate, 2 duplicate)

Auto-fit to window

| Query | Dupes | Observed | Mr(expt) | Mr(calc) | ppm | M | Score | Expect | Rank | U | Peptide |
| --- | --- | --- | --- | --- | --- | --- | --- | --- | --- | --- | --- |

| Query | Dupes | Observed | Mr(expt) | Mr(calc) | ppm | M | Score | Expect | Rank | U | Peptide |
| --- | --- | --- | --- | --- | --- | --- | --- | --- | --- | --- | --- |
| 2908 |  | 560.3125 | 1118.6104 | 1118.6308 | -18.2 | 2 | 8 | 1.2 | 2Score **> 32** indicates **identity** Score **> 21** indicates **homology** | U | R.FGSLRNIRR.K + Deamidated (NQ) |
| 6291 |  | 718.3497 | 1434.6848 | 1434.6811 | 2.58 | 2 | 1 | 4.4 | 5Score **> 34** indicates **identity** Score **> 20** indicates **homology** | U | K.RSQGDNASIRSSR.S + 2 Deamidated (NQ) |
| 9816 |  | 617.9505 | 1850.8297 | 1850.8718 | -22.8 | 2 | 11 | 0.27 | 1Score **> 33** indicates **identity** Score **> 18** indicates **homology** | U | R.SQGDNASIRSSRSSQLR.Q + 3 Deamidated (NQ) |
| 9817 | 2 | 926.4226 | 1850.8305 | 1850.8718 | -22.3 | 2 | 15 | 0.047 | 1Score **> 33** indicates **identity** Score **> 14** indicates **homology** | U | R.SQGDNASIRSSRSSQLR.Q + 3 Deamidated (NQ) |

---

15102050100200300400500600700800900100015002000allper page

Page: 1

---

Not what you expected? Try the select summary.

**Mascot:** http://www.matrixscience.com/

<xsl:stylesheet version="1.0" xmlns:xsl="http://www.w3.org/1999/XSL/Transform" xmlns:cc="http://www.matrixscience.com/xmlns/schema/cc" xmlns:tc="http://www.matrixscience.com/xmlns/schema/tc" xmlns:html="http://www.w3.org/1999/xhtml" > <xsl:output method="xml" version="1.0" indent="no" standalone="yes" omit-xml-declaration="yes" cdata-section-elements="script pre style" /> <xsl:param name="xml-base-supported">1</xsl:param> <xsl:template match="@\*|node()"> <xsl:copy> <xsl:apply-templates select="@\*|node()"/> </xsl:copy> </xsl:template> <xsl:param name="state" select="'default'"/> <xsl:template match="/tc:data"> <xsl:copy> <xsl:apply-templates select="@\*|node()"/> </xsl:copy> </xsl:template> <xsl:template match="/tc:data/tc:elements"> <xsl:if test="@state=$state"> <xsl:copy> <xsl:apply-templates select="@\*|node()"/> </xsl:copy> </xsl:if> </xsl:template> <xsl:template match="tc:\*"> <xsl:copy> <xsl:apply-templates select="@\*|node()"/> </xsl:copy> </xsl:template> <xsl:template match="tc:elements/html:\*"> <xsl:copy> <xsl:copy-of select="./ancestor-or-self::\*/@xml:base"/> <xsl:call-template name="massage-html-element"/> </xsl:copy> </xsl:template> <xsl:template match="html:\*"> <xsl:copy> <xsl:call-template name="massage-html-element"/> </xsl:copy> </xsl:template> <xsl:template name="massage-html-element"> <xsl:choose> <xsl:when test="@href or @src or @action or @data"> <xsl:copy-of select="./ancestor-or-self::\*/@xml:base"/> <xsl:call-template name="massage-uri"> <xsl:with-param name="base"><xsl:value-of select="./ancestor::\*/@xml:base"/></xsl:with-param> </xsl:call-template> <xsl:apply-templates select="@\*[local-name() != 'href' and local-name() != 'src' and local-name() != 'action' and local-name() != 'data']|node()"/> </xsl:when> <xsl:when test="local-name()='param' and @name='src'"> <xsl:copy-of select="./ancestor-or-self::\*/@xml:base"/> <xsl:apply-templates select="@\*|node()"/> </xsl:when> <xsl:otherwise> <xsl:apply-templates select="@\*|node()"/> </xsl:otherwise> </xsl:choose> <xsl:if test="not(contains(' area base basefont br col frame hr img input isindex links meta param ', concat(' ', normalize-space(local-name()), ' ')))"> <xsl:if test="count(./\*) = 0 and (string-length(./child::text()) = 0 or normalize-space(./child::text()) = ' ' or normalize-space(./child::text()) = '')"> <xsl:text>&#10;</xsl:text> </xsl:if> </xsl:if> </xsl:template> <xsl:template name="massage-uri"> <xsl:param name="base"/> <xsl:variable name="uri-attribute"> <xsl:choose> <xsl:when test="@href">href</xsl:when> <xsl:when test="@src">src</xsl:when> <xsl:when test="@action">action</xsl:when> <xsl:when test="@data">data</xsl:when> </xsl:choose> </xsl:variable> <xsl:variable name="uri-value"> <xsl:choose> <xsl:when test="@href"><xsl:value-of select="@href"/></xsl:when> <xsl:when test="@src"><xsl:value-of select="@src"/></xsl:when> <xsl:when test="@action"><xsl:value-of select="@action"/></xsl:when> <xsl:when test="@data"><xsl:value-of select="@data"/></xsl:when> </xsl:choose> </xsl:variable> <xsl:choose> <xsl:when test="$base and $xml-base-supported != 1 and starts-with($base, 'http://') and $uri-attribute and starts-with($uri-value, '../')"> <xsl:choose> <xsl:when test="substring($base, string-length($base), 1) = '/'"> <xsl:attribute name="{$uri-attribute}"> <xsl:value-of select="concat($base, $uri-value)"/> </xsl:attribute> </xsl:when> <xsl:otherwise> <xsl:attribute name="{$uri-attribute}"><xsl:value-of select="concat($base, '/', $uri-value)"/></xsl:attribute> </xsl:otherwise> </xsl:choose> </xsl:when> <xsl:otherwise> <xsl:attribute name="{$uri-attribute}"><xsl:value-of select="$uri-value"/></xsl:attribute> </xsl:otherwise> </xsl:choose> </xsl:template> <xsl:template match="@cc:in"/> <xsl:variable name="IE" select="system-property('xsl:vendor')='Microsoft'"/> <xsl:template match="\*[@cc:in='!IE']"> <xsl:if test="not($IE)"> <xsl:copy> <xsl:apply-templates select="@\*|node()"/> </xsl:copy> </xsl:if> </xsl:template> <xsl:template match="\*[@cc:in and not(contains(@cc:in, '!IE'))]"> <xsl:if test="$IE"> <xsl:comment><![CDATA[[if IE]><![if ]]><xsl:value-of select="@cc:in"/><![CDATA[]><![endif]]]></xsl:comment> <xsl:copy> <xsl:apply-templates select="@\*|node()"/> </xsl:copy> <xsl:comment><![CDATA[[if IE]><![endif]><![endif]]]></xsl:comment> </xsl:if> </xsl:template></xsl:stylesheet>
%3Cxsl%3Astylesheet%20version%3D%221.0%22%20%20%20%20xmlns%3Axsl%3D%22http%3A%2F%2Fwww.w3.org%2F1999%2FXSL%2FTransform%22%20%20%20%20xmlns%3Ahtml%3D%22http%3A%2F%2Fwww.w3.org%2F1999%2Fxhtml%22%20%20%20%20xmlns%3D%22http%3A%2F%2Fwww.w3.org%2F1999%2Fxhtml%22%20%20%20%20id%3D%22FloatingTableCells%22%20%20%20%20%3E%20%3Cxsl%3Apreserve-space%20elements%3D%22\*%22%2F%3E%20%3Cxsl%3Atemplate%20match%3D%22%40\*%7Cnode()%22%3E%20%3Cxsl%3Acopy%3E%20%3Cxsl%3Aapply-templates%20select%3D%22%40\*%7Cnode()%22%2F%3E%20%3C%2Fxsl%3Acopy%3E%20%3C%2Fxsl%3Atemplate%3E%20%3Cxsl%3Atemplate%20match%3D%22%40\*%7Cnode()%22%20mode%3D%22screen-form%22%3E%20%3Cxsl%3Acopy%3E%20%3Cxsl%3Aapply-templates%20select%3D%22%40\*%7Cnode()%22%20mode%3D%22screen-form%22%2F%3E%20%3C%2Fxsl%3Acopy%3E%20%3C%2Fxsl%3Atemplate%3E%20%3Cxsl%3Atemplate%20match%3D%22%40\*%7Cnode()%22%20mode%3D%22print-form%22%3E%20%3Cxsl%3Acopy%3E%20%3Cxsl%3Aapply-templates%20select%3D%22%40\*%7Cnode()%22%20mode%3D%22print-form%22%2F%3E%20%3C%2Fxsl%3Acopy%3E%20%3C%2Fxsl%3Atemplate%3E%20%3Cxsl%3Atemplate%20match%3D%22%40\*%7Cnode()%22%20mode%3D%22screen-table%22%3E%20%3Cxsl%3Acopy%3E%20%3Cxsl%3Aapply-templates%20select%3D%22%40\*%7Cnode()%22%20mode%3D%22screen-table%22%2F%3E%20%3C%2Fxsl%3Acopy%3E%20%3C%2Fxsl%3Atemplate%3E%20%3Cxsl%3Akey%20%20%20%20%20%20%20%20%20name%3D%22is-floating-table%22%20%20%20%20%20%20%20%20%20match%3D%22html%3Atable%5Bcontains(concat('%20'%2C%20normalize-space(%40class)%2C%20'%20')%2C%20'%20has-floating-table-cells%20')%5D%22%20%20%20%20%20%20%20%20%20use%3D%22generate-id()%22%20%20%20%20%20%20%20%20%2F%3E%20%3Cxsl%3Atemplate%20match%3D%22html%3Aform%22%3E%20%3Cxsl%3Achoose%3E%20%3Cxsl%3Awhen%20test%3D%22.%2F%2Fhtml%3Atable%5Bkey('is-floating-table'%2C%20generate-id())%5D%22%3E%20%3Cxsl%3Acopy%3E%20%3Cxsl%3Aapply-templates%20select%3D%22%40\*%7Cnode()%22%20mode%3D%22print-form%22%2F%3E%20%3C%2Fxsl%3Acopy%3E%20%3Cxsl%3Acopy%3E%20%3Cxsl%3Aapply-templates%20select%3D%22%40\*%7Cnode()%22%20mode%3D%22screen-form%22%2F%3E%20%3C%2Fxsl%3Acopy%3E%20%3C%2Fxsl%3Awhen%3E%20%3Cxsl%3Aotherwise%3E%20%3Cxsl%3Acopy%3E%20%3Cxsl%3Aapply-templates%20select%3D%22%40\*%7Cnode()%22%20%2F%3E%20%3C%2Fxsl%3Acopy%3E%20%3C%2Fxsl%3Aotherwise%3E%20%3C%2Fxsl%3Achoose%3E%20%3C%2Fxsl%3Atemplate%3E%20%3Cxsl%3Atemplate%20match%3D%22html%3Atable%5Bnot(ancestor%3A%3Ahtml%3Aform)%5D%22%3E%20%3Cxsl%3Achoose%3E%20%3Cxsl%3Awhen%20test%3D%22key('is-floating-table'%2C%20generate-id())%22%3E%20%3Cxsl%3Acall-template%20name%3D%22create-print-table%22%2F%3E%20%3Cxsl%3Acall-template%20name%3D%22create-screen-table%22%2F%3E%20%3C%2Fxsl%3Awhen%3E%20%3Cxsl%3Aotherwise%3E%20%3Cxsl%3Acopy%3E%20%3Cxsl%3Aapply-templates%20select%3D%22%40\*%7Cnode()%22%2F%3E%20%3C%2Fxsl%3Acopy%3E%20%3C%2Fxsl%3Aotherwise%3E%20%3C%2Fxsl%3Achoose%3E%20%3C%2Fxsl%3Atemplate%3E%20%3Cxsl%3Atemplate%20match%3D%22html%3Atable%5Bancestor%3A%3Ahtml%3Aform%5D%22%20mode%3D%22screen-form%22%3E%20%3Cxsl%3Achoose%3E%20%3Cxsl%3Awhen%20test%3D%22key('is-floating-table'%2C%20generate-id())%22%3E%20%3Cxsl%3Acall-template%20name%3D%22create-screen-table%22%2F%3E%20%3C%2Fxsl%3Awhen%3E%20%3Cxsl%3Aotherwise%3E%20%3Cxsl%3Acopy%3E%20%3Cxsl%3Aapply-templates%20select%3D%22%40\*%7Cnode()%22%20mode%3D%22screen-form%22%2F%3E%20%3C%2Fxsl%3Acopy%3E%20%3C%2Fxsl%3Aotherwise%3E%20%3C%2Fxsl%3Achoose%3E%20%3C%2Fxsl%3Atemplate%3E%20%3Cxsl%3Atemplate%20match%3D%22html%3Atable%5Bancestor%3A%3Ahtml%3Aform%5D%22%20mode%3D%22print-form%22%3E%20%3Cxsl%3Achoose%3E%20%3Cxsl%3Awhen%20test%3D%22key('is-floating-table'%2C%20generate-id())%22%3E%20%3Cxsl%3Acall-template%20name%3D%22create-print-table%22%2F%3E%20%3C%2Fxsl%3Awhen%3E%20%3Cxsl%3Aotherwise%3E%20%3Cxsl%3Acopy%3E%20%3Cxsl%3Aapply-templates%20select%3D%22%40\*%7Cnode()%22%20mode%3D%22print-form%22%2F%3E%20%3C%2Fxsl%3Acopy%3E%20%3C%2Fxsl%3Aotherwise%3E%20%3C%2Fxsl%3Achoose%3E%20%3C%2Fxsl%3Atemplate%3E%20%3Cxsl%3Atemplate%20name%3D%22create-screen-table%22%3E%20%3Cxsl%3Acall-template%20name%3D%22create-table%22%3E%20%3Cxsl%3Awith-param%20name%3D%22table-type%22%20select%3D%22'floating-table-cells-screen'%22%2F%3E%20%3C%2Fxsl%3Acall-template%3E%20%3C%2Fxsl%3Atemplate%3E%20%3Cxsl%3Atemplate%20name%3D%22create-print-table%22%3E%20%3Cxsl%3Acall-template%20name%3D%22create-table%22%3E%20%3Cxsl%3Awith-param%20name%3D%22table-type%22%20select%3D%22'floating-table-cells-print'%22%2F%3E%20%3C%2Fxsl%3Acall-template%3E%20%3C%2Fxsl%3Atemplate%3E%20%3Cxsl%3Atemplate%20name%3D%22create-table%22%3E%20%3Cxsl%3Aparam%20name%3D%22table-type%22%2F%3E%20%3Cxsl%3Avariable%20name%3D%22full-class%22%20select%3D%22concat('%20'%2C%20normalize-space(%40class)%2C%20'%20')%22%2F%3E%20%3Cxsl%3Acopy%3E%20%3Cxsl%3Aapply-templates%20select%3D%22%40\*%5Bname()%20!%3D%20'class'%5D%22%2F%3E%20%3Cxsl%3Aattribute%20name%3D%22class%22%3E%20%3Cxsl%3Achoose%3E%20%3Cxsl%3Awhen%20test%3D%22contains(%24full-class%2C%20'%20has-floating-table-cells%20')%22%3E%20%3Cxsl%3Avalue-of%20select%3D%22normalize-space(concat(substring-before(%24full-class%2C%20'%20has-floating-table-cells%20')%2C%20'%20'%2C%20substring-after(%24full-class%2C%20'%20has-floating-table-cells%20')%2C%20'%20'%2C%20%24table-type))%22%2F%3E%20%3C%2Fxsl%3Awhen%3E%20%3Cxsl%3Aotherwise%3E%20%3Cxsl%3Avalue-of%20select%3D%22normalize-space(concat(%24full-class%2C%20'%20'%2C%20%24table-type))%22%2F%3E%20%3C%2Fxsl%3Aotherwise%3E%20%3C%2Fxsl%3Achoose%3E%20%3C%2Fxsl%3Aattribute%3E%20%3Cxsl%3Achoose%3E%20%3Cxsl%3Awhen%20test%3D%22%24table-type%20%3D%20'floating-table-cells-screen'%22%3E%20%3Cxsl%3Aapply-templates%20select%3D%22node()%22%20mode%3D%22screen-table%22%2F%3E%20%3C%2Fxsl%3Awhen%3E%20%3Cxsl%3Aotherwise%3E%20%3Cxsl%3Aapply-templates%20select%3D%22node()%22%2F%3E%20%3C%2Fxsl%3Aotherwise%3E%20%3C%2Fxsl%3Achoose%3E%20%3C%2Fxsl%3Acopy%3E%20%3C%2Fxsl%3Atemplate%3E%20%3Cxsl%3Akey%20%20%20%20%20%20%20%20%20name%3D%22floated-sibling%22%20%20%20%20%20%20%20%20%20match%3D%22html%3Atable%5Bcontains(concat('%20'%2C%20normalize-space(%40class)%2C%20'%20')%2C%20'%20has-floating-table-cells%20')%5D%2Fhtml%3Atbody%2Fhtml%3Atr%5Bhtml%3Atd%5Bcontains(concat('%20'%2C%20normalize-space(%40class)%2C%20'%20')%2C%20'%20floating-table-cell%20')%5D%5D%22%20%20%20%20%20%20%20%20%20use%3D%22generate-id(preceding-sibling%3A%3Ahtml%3Atr%5B1%5D)%22%20%20%20%20%20%20%20%20%2F%3E%20%3Cxsl%3Atemplate%20match%3D%22html%3Atr%22%20mode%3D%22screen-table%22%3E%20%3Cxsl%3Acopy%3E%20%3Cxsl%3Achoose%3E%20%3Cxsl%3Awhen%20test%3D%22count(key('floated-sibling'%2C%20generate-id()))%22%3E%20%3Cxsl%3Aapply-templates%20select%3D%22%40\*%22%20mode%3D%22screen-table%22%2F%3E%20%3Cxsl%3Avariable%20name%3D%22nodes%22%20select%3D%22key('floated-sibling'%2C%20generate-id())%2Fhtml%3Atd%5Bcontains(concat('%20'%2C%20normalize-space(%40class)%2C%20'%20')%2C%20'%20floating-table-cell%20')%5D%2Fnode()%22%20%2F%3E%20%3Cxsl%3Afor-each%20select%3D%22\*%22%3E%20%3Cxsl%3Acopy%3E%20%3Cxsl%3Achoose%3E%20%3Cxsl%3Awhen%20test%3D%22following-sibling%3A%3A\*%22%3E%20%3Cxsl%3Aapply-templates%20select%3D%22%40\*%7Cnode()%22%20mode%3D%22screen-table%22%2F%3E%20%3C%2Fxsl%3Awhen%3E%20%3Cxsl%3Aotherwise%3E%20%3Cxsl%3Aapply-templates%20select%3D%22%40\*%22%20mode%3D%22screen-table%22%2F%3E%20%3Cxsl%3Acopy-of%20select%3D%22%24nodes%22%2F%3E%20%3C%2Fxsl%3Aotherwise%3E%20%3C%2Fxsl%3Achoose%3E%20%3C%2Fxsl%3Acopy%3E%20%3C%2Fxsl%3Afor-each%3E%20%3C%2Fxsl%3Awhen%3E%20%3Cxsl%3Aotherwise%3E%20%3Cxsl%3Aapply-templates%20select%3D%22%40\*%7Cnode()%22%20mode%3D%22screen-table%22%2F%3E%20%3C%2Fxsl%3Aotherwise%3E%20%3C%2Fxsl%3Achoose%3E%20%3C%2Fxsl%3Acopy%3E%20%3C%2Fxsl%3Atemplate%3E%20%3Cxsl%3Atemplate%20match%3D%22html%3Atd%5Bcontains(concat('%20'%2C%20normalize-space(%40class)%2C%20'%20')%2C%20'%20floating-table-cell%20')%5D%22%20mode%3D%22screen-table%22%3E%20%3Cxsl%3Acopy%3E%20%3Cxsl%3Aapply-templates%20select%3D%22%40\*%22%20mode%3D%22screen-table%22%2F%3E%20%3C%2Fxsl%3Acopy%3E%20%3C%2Fxsl%3Atemplate%3E%3C%2Fxsl%3Astylesheet%3E
%3Cxsl%3Astylesheet%20version%3D%221.0%22%20%20%20%20xmlns%3Axsl%3D%22http%3A%2F%2Fwww.w3.org%2F1999%2FXSL%2FTransform%22%20%20%20%20xmlns%3Ahtml%3D%22http%3A%2F%2Fwww.w3.org%2F1999%2Fxhtml%22%20%20%20%20xmlns%3D%22http%3A%2F%2Fwww.w3.org%2F1999%2Fxhtml%22%20%20%20%20id%3D%22ScrollableTable%22%20%20%20%20%3E%20%3Cxsl%3Apreserve-space%20elements%3D%22\*%22%2F%3E%20%3Cxsl%3Atemplate%20match%3D%22%40\*%7Cnode()%22%3E%20%3Cxsl%3Acopy%3E%20%3Cxsl%3Aapply-templates%20select%3D%22%40\*%7Cnode()%22%2F%3E%20%3C%2Fxsl%3Acopy%3E%20%3C%2Fxsl%3Atemplate%3E%20%3Cxsl%3Atemplate%20match%3D%22html%3Atable%5Bcontains(concat('%20'%2C%20normalize-space(%40class)%2C%20'%20')%2C%20'%20scrollable-table%20')%5D%22%3E%20%3Cxsl%3Avariable%20name%3D%22full-class%22%20select%3D%22concat('%20'%2C%20normalize-space(%40class)%2C%20'%20')%22%2F%3E%20%3Cxsl%3Avariable%20name%3D%22reduced-class%22%20select%3D%22normalize-space(concat(substring-before(%24full-class%2C%20'%20scrollable-table%20')%2C%20'%20'%2C%20substring-after(%24full-class%2C%20'%20scrollable-table%20')))%22%2F%3E%20%3Cdiv%3E%20%3Cxsl%3Aapply-templates%20select%3D%22%40\*%5Bname()%20!%3D%20'class'%5D%22%2F%3E%20%3Cxsl%3Aattribute%20name%3D%22class%22%3E%20%3Cxsl%3Avalue-of%20select%3D%22normalize-space(concat(%24reduced-class%2C%20'%20scrollable-table-header%20scrollable-table-header-enabled'))%22%2F%3E%20%3C%2Fxsl%3Aattribute%3E%20%3Cp%20class%3D%22scrollable-toggle%22%3E%20%3Clabel%3E%20%3Cinput%20type%3D%22checkbox%22%20checked%3D%22checked%22%2F%3E%20Auto-fit%20to%20window%20%20%20%20%20%20%20%20%20%20%20%20%20%20%20%20%3C%2Flabel%3E%20%3C%2Fp%3E%20%3Ctable%20class%3D%22%7B%24reduced-class%7D%22%3E%20%3Cxsl%3Aapply-templates%20select%3D%22html%3Acol%7Chtml%3Acolgroup%7Chtml%3Athead%22%2F%3E%20%3C%2Ftable%3E%20%3C%2Fdiv%3E%20%3Cdiv%3E%20%3Cxsl%3Aapply-templates%20select%3D%22%40\*%5Bname()%20!%3D%20'class'%5D%22%2F%3E%20%3Cxsl%3Aattribute%20name%3D%22class%22%3E%20%3Cxsl%3Avalue-of%20select%3D%22normalize-space(concat(%24reduced-class%2C%20'%20scrollable-table-container%20scrollable-table-container-enabled'))%22%2F%3E%20%3C%2Fxsl%3Aattribute%3E%20%3Ctable%20class%3D%22%7B%24reduced-class%7D%22%3E%20%3Cxsl%3Aapply-templates%20select%3D%22node()%22%2F%3E%20%3C%2Ftable%3E%20%3C%2Fdiv%3E%20%3C%2Fxsl%3Atemplate%3E%3C%2Fxsl%3Astylesheet%3E
%3Cxsl%3Astylesheet%20version%3D%221.0%22%20%20%20%20xmlns%3Axsl%3D%22http%3A%2F%2Fwww.w3.org%2F1999%2FXSL%2FTransform%22%20%20%20%20xmlns%3Ahtml%3D%22http%3A%2F%2Fwww.w3.org%2F1999%2Fxhtml%22%20%20%20%20xmlns%3D%22http%3A%2F%2Fwww.w3.org%2F1999%2Fxhtml%22%20%20%20%20id%3D%22ToggledContent.Capture%22%20%20%20%20%3E%20%3Cxsl%3Apreserve-space%20elements%3D%22\*%22%2F%3E%20%3Cxsl%3Atemplate%20match%3D%22%40\*%7Cnode()%22%3E%20%3Cxsl%3Acopy%3E%20%3Cxsl%3Aapply-templates%20select%3D%22%40\*%7Cnode()%22%2F%3E%20%3C%2Fxsl%3Acopy%3E%20%3C%2Fxsl%3Atemplate%3E%20%3Cxsl%3Akey%20%20%20%20%20%20%20%20%20name%3D%22controls%22%20%20%20%20%20%20%20%20%20match%3D%22\*%5B(local-name()%3D'a'%20or%20local-name()%3D'span')%20and%20%40tc-control%20and%20contains(concat('%20'%2C%20normalize-space(%40class)%2C%20'%20')%2C%20'%20tc-static%20')%20and%20not(%40href)%20and%20(%40tc-toggled%3D0%20or%20%40tc-toggled%3D1)%5D%22%20%20%20%20%20%20%20%20%20use%3D%22%40tc-control%22%20%20%20%20%20%20%20%20%2F%3E%20%3Cxsl%3Atemplate%20match%3D%22\*%5Bkey('controls'%2C%20%40tc-control)%5D%22%3E%20%3Cxsl%3Acopy%3E%20%3Cxsl%3Aapply-templates%20select%3D%22%40\*%22%2F%3E%20%3Cimg%20class%3D%22symbol%22%20title%3D%22%22%3E%20%3Cxsl%3Acopy-of%20select%3D%22.%2Fancestor-or-self%3A%3A\*%2F%40xml%3Abase%22%2F%3E%20%3Cxsl%3Achoose%3E%20%3Cxsl%3Awhen%20test%3D%22%40tc-toggled%20%3D%200%22%3E%20%3Cxsl%3Aattribute%20name%3D%22src%22%3E..%2Fimages%2Fnode-closed.gif%3C%2Fxsl%3Aattribute%3E%20%3Cxsl%3Aattribute%20name%3D%22alt%22%3E%2B%3C%2Fxsl%3Aattribute%3E%20%3C%2Fxsl%3Awhen%3E%20%3Cxsl%3Awhen%20test%3D%22%40tc-toggled%20%3D%201%22%3E%20%3Cxsl%3Aattribute%20name%3D%22src%22%3E..%2Fimages%2Fnode-opened.gif%3C%2Fxsl%3Aattribute%3E%20%3Cxsl%3Aattribute%20name%3D%22alt%22%3E-%3C%2Fxsl%3Aattribute%3E%20%3C%2Fxsl%3Awhen%3E%20%3C%2Fxsl%3Achoose%3E%20%3C%2Fimg%3E%20%3Cxsl%3Aapply-templates%20select%3D%22node()%22%2F%3E%20%3C%2Fxsl%3Acopy%3E%20%3C%2Fxsl%3Atemplate%3E%20%3Cxsl%3Atemplate%20match%3D%22\*%5B%40tc-content%5D%22%3E%20%3Cxsl%3Avariable%20name%3D%22toggles%22%20select%3D%22key('controls'%2C%20%40tc-content)%22%2F%3E%20%3Cxsl%3Achoose%3E%20%3Cxsl%3Awhen%20test%3D%22count(%24toggles)%3D0%22%3E%20%3Cxsl%3Acopy%3E%3Cxsl%3Aapply-templates%20select%3D%22%40\*%7Cnode()%22%2F%3E%3C%2Fxsl%3Acopy%3E%20%3C%2Fxsl%3Awhen%3E%20%3Cxsl%3Aotherwise%3E%20%3Cxsl%3Avariable%20name%3D%22togglestate%22%20select%3D%22number(%24toggles%5B1%5D%2F%40tc-toggled)%22%2F%3E%20%3Cxsl%3Avariable%20name%3D%22full-class%22%20select%3D%22concat('%20'%2C%20normalize-space(%40class)%2C%20'%20')%22%2F%3E%20%3Cxsl%3Acopy%3E%20%3Cxsl%3Aapply-templates%20select%3D%22%40\*%5Bname()%20!%3D%20'class'%5D%22%2F%3E%20%3Cxsl%3Aattribute%20name%3D%22class%22%3E%20%3Cxsl%3Achoose%3E%20%3Cxsl%3Awhen%20test%3D%22%24togglestate%20%3D%201%22%3E%20%3Cxsl%3Avalue-of%20select%3D%22normalize-space(%24full-class)%22%2F%3E%20%3C%2Fxsl%3Awhen%3E%20%3Cxsl%3Awhen%20test%3D%22%24togglestate%20%3D%200%22%3E%20%3Cxsl%3Avalue-of%20select%3D%22normalize-space(concat(%24full-class%2C%20'%20tc-invisible'))%22%2F%3E%20%3C%2Fxsl%3Awhen%3E%20%3Cxsl%3Aotherwise%3E%20%3Cxsl%3Avalue-of%20select%3D%22normalize-space(%24full-class)%22%2F%3E%20%3C%2Fxsl%3Aotherwise%3E%20%3C%2Fxsl%3Achoose%3E%20%3C%2Fxsl%3Aattribute%3E%20%3Cxsl%3Aapply-templates%20select%3D%22node()%22%2F%3E%20%3C%2Fxsl%3Acopy%3E%20%3C%2Fxsl%3Aotherwise%3E%20%3C%2Fxsl%3Achoose%3E%20%3C%2Fxsl%3Atemplate%3E%20%3Cxsl%3Atemplate%20match%3D%22html%3Aform%5B%40tc-control%5D%22%3E%20%3Cform%20onsubmit%3D%22return%20ToggledContent.Capture.handle\_onsubmit(this)%3B%22%20tc-captured%3D%221%22%3E%20%3Cxsl%3Aapply-templates%20select%3D%22%40\*%7Cnode()%22%2F%3E%20%3C%2Fform%3E%20%3C%2Fxsl%3Atemplate%3E%3C%2Fxsl%3Astylesheet%3E


Paste link in e-mail or notepad  
Select allNew windowClose

gi|1095456207|gb|APA13162.1|
